# Functional analysis of structural variants in single cells using Strand-seq

**DOI:** 10.1038/s41587-022-01551-4

**Published:** 2022-11-24

**Authors:** Hyobin Jeong, Karen Grimes, Kerstin K. Rauwolf, Peter-Martin Bruch, Tobias Rausch, Patrick Hasenfeld, Eva Benito, Tobias Roider, Radhakrishnan Sabarinathan, David Porubsky, Sophie A. Herbst, Büşra Erarslan-Uysal, Johann-Christoph Jann, Tobias Marschall, Daniel Nowak, Jean-Pierre Bourquin, Andreas E. Kulozik, Sascha Dietrich, Beat Bornhauser, Ashley D. Sanders, Jan O. Korbel

**Affiliations:** 1 Genome Biology Unit, European Molecular Biology Laboratory (EMBL), Heidelberg, Germany; 2 Faculty of Biosciences, EMBL and Heidelberg University, Heidelberg, Germany; 3 Division of Pediatric Oncology, University Children’s Hospital, Zürich, Switzerland; 4 Department of Hematology, Oncology and Rheumatology, Heidelberg University Hospital, Heidelberg, Germany; 5 Molecular Medicine Partnership Unit, European Molecular Biology Laboratory, University of Heidelberg, Heidelberg, Germany; 6 Department of Hematology and Oncology, University Hospital Düsseldorf, Düsseldorf, Germany; 7 National Centre for Biological Sciences, Tata Institute of Fundamental Research, Bangalore, India; 8 Center for Bioinformatics, Saarland University, Saarbrücken, Germany; 9 Max Planck Institute for Informatics, Saarbrücken, Germany; 10 Department of Pediatric Oncology, Hematology, and Immunology, University of Heidelberg and Hopp Children’s Cancer Center, Heidelberg, Germany; 11 Department of Hematology and Oncology, Medical Faculty Mannheim of the Heidelberg University, Heidelberg, Germany; 12 Institute for Medical Biometry and Bioinformatics, Medical Faculty, Heinrich Heine University, Düsseldorf, Germany; 13 Department of Translational Medical Oncology, National Center for Tumor Diseases (NCT) Heidelberg and German Cancer Research Center (DKFZ), Heidelberg, Germany; 14 Berlin Institute for Medical Systems Biology, Max Delbrück Center for Molecular Medicine in the Helmholtz Association (MDC), Berlin, Germany; 15 Berlin Institute of Health (BIH), Berlin, Germany; 16 Charité-Universitätsmedizin, Berlin, Germany; 17 Bridging Research Division on Mechanisms of Genomic Variation and Data Science, German Cancer Research Center (DKFZ), Heidelberg, Germany; 18 Present Address: Hanyang Institute of Bioscience and Biotechnology, Hanyang University, Seoul, Republic of Korea; 19 Present Address: Department of Genome Sciences, University of Washington School of Medicine, Seattle, WA USA

**Keywords:** Cancer genomics, Data integration, Epigenomics

## Abstract

Somatic structural variants (SVs) are widespread in cancer, but their impact on disease evolution is understudied due to a lack of methods to directly characterize their functional consequences. We present a computational method, scNOVA, which uses Strand-seq to perform haplotype-aware integration of SV discovery and molecular phenotyping in single cells by using nucleosome occupancy to infer gene expression as a readout. Application to leukemias and cell lines identifies local effects of copy-balanced rearrangements on gene deregulation, and consequences of SVs on aberrant signaling pathways in subclones. We discovered distinct SV subclones with dysregulated Wnt signaling in a chronic lymphocytic leukemia patient. We further uncovered the consequences of subclonal chromothripsis in T cell acute lymphoblastic leukemia, which revealed c-Myb activation, enrichment of a primitive cell state and informed successful targeting of the subclone in cell culture, using a Notch inhibitor. By directly linking SVs to their functional effects, scNOVA enables systematic single-cell multiomic studies of structural variation in heterogeneous cell populations.

## Abstract (web summary)

Linking structural variants to gene expression information identifies aberrant signaling pathways.

## Main

The mutational landscapes of numerous cancers were recently cataloged^1,2^, revealing that somatic SVs represent around 55% of driver mutations^2,3^. Somatic mutational processes generate a broad spectrum of SVs from simple (for example, deletions and inversions) to complex classes (for example, chromothripsis)^4–8^, and these SVs are important drivers of malignancy, metastasis and relapse^9–12^. However, with the exception of focal deletions and amplifications, somatic SVs have proven difficult to characterize functionally in cancer genomic surveys^1–3,13^. Studies integrating transcriptome and whole genome sequencing (WGS) data have inferred SV functional outcomes^13–16^, but these typically require large cohorts and do not account for intratumor heterogeneity (ITH)^3^. Instead, SV effects can be measured directly by reading both genotype and molecular phenotype in the same cell, using single-cell multiomics^17–21^. Several such methods have been developed^17–20^, but these do not presently account for small (<10 Mb) somatic copy number alterations (SCNAs), balanced SVs and complex rearrangement events like chromothripsis^4,5,7,22^, which has limited efforts to functionally characterize the most common class of driver mutations in cancer.

To address this, we developed scNOVA (single-cell nucleosome occupancy and genetic variation analysis)—a method enabling functional characterization of the full spectrum of somatic SV classes. scNOVA uses Strand-seq^23^ in two ways: (1) it uses the DNA fragmentation pattern resulting from micrococcal nuclease (MNase) digestion^23^ to directly measure nucleosome occupancy (NO) and indirectly infer patterns of gene activity, and (2) it couples this ‘molecular phenotype’ with SVs discovered by single-cell tri-channel processing (scTRIP, which jointly models read-orientation, read depth and haplotype-phase^24^) in the same cell. MNase digests the linker DNA between nucleosomes, leaving nucleosome-protected DNA intact, to enable genome-wide inference of NO by measuring sequence read counts^25–28^. Previous work has shown that active enhancers and transcribed genes exhibit reduced NO^25–30^. However, the relationships between NO and SV landscapes in cancer remain unexplored. scNOVA addresses this by integrating SVs and NO along the genome of a cell, to functionally characterize SVs in heterogeneous samples.

## Results

### NO classifies cell types and predicts gene activity changes

#### Strand-seq data reveals NO

**Fig. 1:**
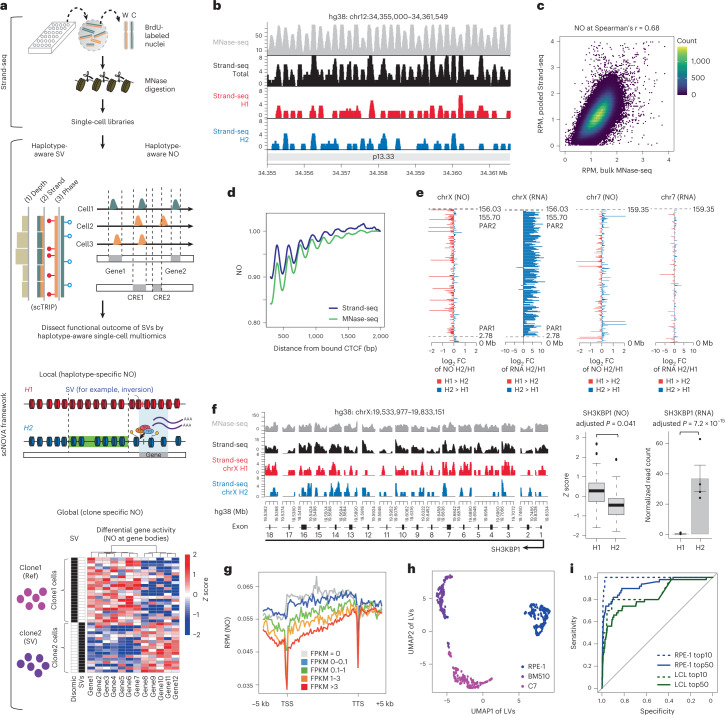
Haplotype-aware single-cell multiomics to functionally characterize SVs. **a**, Leveraging Strand-seq, scNOVA performs SV discovery and then, using phased NO tracks, identifies functional effects of SVs locally (via evaluation of haplotype-specific NO) and globally (clone-specific NO). Orange, Strand-seq reads mapped to the Watson (W) strand; green, reads mapped to the Crick (C) strand. **b**, Strand-seq-based NO tracks in NA12878 reveal nucleosome positions well-concordant with bulk MNase-seq, depicted for a chromosome 12 locus with relatively regular nucleosome positioning^92^. Red, NO tracks mapping to haplotype 1 (H1); blue, H2; black, combining phased and unphased reads; gray, MNase-seq. The *y* axis depicts the mean read counts at each bp in 10 bp bins. **c**, Correlated NO at consensus DNase I hypersensitive sites^33^ for NA12878. **d**, Averaged nucleosome patterns at CTCF binding sites^34^ in NA12878, using pseudobulk Strand-seq and MNase-seq. **e**, FCs of haplotype-resolved NO in gene bodies plotted for chromosome X and chromosome 7 (a representative autosome) in NA12878. FCs of haplotype-resolved RNA expression measurements are shown to the right. **f**, Pseudobulk haplotype-phased NO track of exons of the representative chromosome X gene *SH3KBP1* based on Strand-seq. Boxplots comparing H1 and H2 use two-sided Wilcoxon rank sum tests followed by Benjamini–Hochberg multiple testing (FDR) correction (boxplots defined by minima = 25th percentile – 1.5 × interquartile range (IQR), maxima = 75th percentile + 1.5 × IQR, center = median and bounds of box = 25th and 75th percentile; *n* = 47 single cells). Bar charts show haplotype-specific RNA expression of *SH3KBP1* (two-sided likelihood ratio test followed by FDR correction; *n* = 4 biological replicates; data are presented as mean values ± s.e.m.). **g**, Inverse correlation of NO at gene bodies and gene expression. NO is based on pseudobulk Strand-seq libraries from RPE-1. Gene bodies were scaled to the same length. **h**, Cell-typing based on NO at gene bodies (AUC = 0.96). Cell line codes: Blue, RPE-1; Purple, BM510; Magenta, C7; LV, latent variable. **i**, Receiver operating characteristics for inferring altered gene activity by analyzing NO at gene bodies, using pseudobulk Strand-seq libraries from in silico cell mixing.

**Extended Data Fig. 1:**
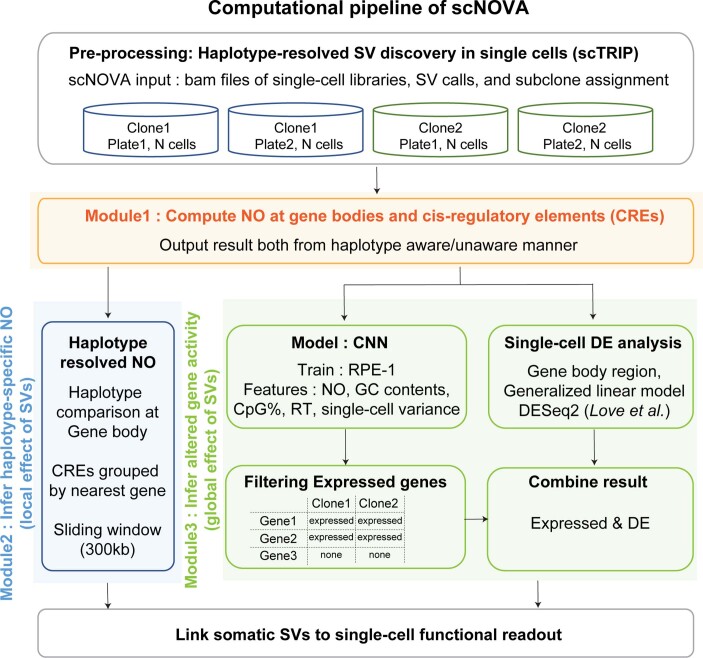
Overview of components of the scNOVA computational workflow. scNOVA employs single cell tri-channel processing (scTRIP) as realized in the MosaiCatcher pipeline to perform haplotype-aware somatic SV discovery^24^. Modules of scNOVA enable single-cell mulitomics of these somatic SVs, including inference of haplotype-specific NO to investigate local (*cis*) effect of SVs, and inference of altered gene/pathway activity to investigate global (*trans*) effect of SVs detectable between geneticlaly distinct subclones. To infer alterations in gene activity, scNOVA integrates deep convolutional neural network (CNN) based machine learning, and negative binomial generalized linear models. The framework dissects intra-sample genetic heterogeneity at single-cell resolution, measures the local haplotype-specific impact of somatic SVs, can be used to explore global gene dysregulation in SV-containing cells, can discriminate between genetically-distinct subclones, and can uncover shared functional consequences of heterogeneous SVs affecting the same chromosomal interval.

**Extended Data Fig. 2:**
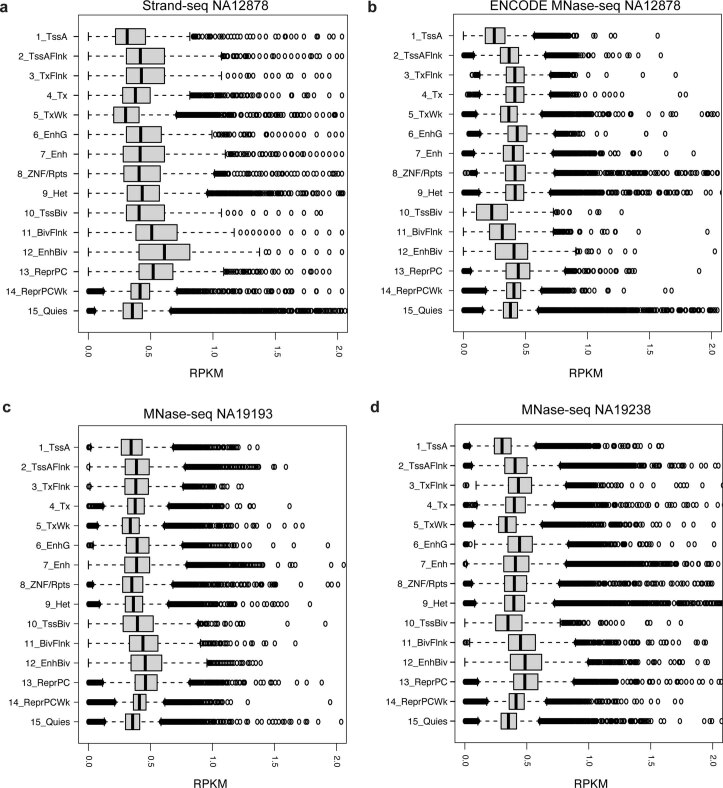
Read depth of Strand-seq and MNase-seq data stratified into 15 chromatin states defined by Roadmap epigenome consortium^33^. 15 chromatin states based on the NA12878 cell line were utilized in this genome-wide analysis. Plots generated represent Strand-seq data from NA12878 (n = 95 cells) **(a)**, and publicly available MNase-seq from NA12878, NA19193, and NA19238 (n = 1 sample each) **(b-d)**. The bulk MNase-seq experiment of NA12878 was pursued using single-end SOLID sequencing reads, and that of NA19193 and NA19238 was done using paired-end Illumina reads. The X-axis in the box plot indicates reads per kilobase per million (RPKM) measured for each genomic segment annotated by one of the 15 chromatin states. Abbreviations for chromatin states^33^ are: TssA-Active TSS, TssAFlnk-Flanking Active TSS, TxFlnk - Transcription at gene 5’and 3’, Tx - Strong transcription, TxWk - Weak transcription, EnhG - Genic enhancers, Enh - Enhancers, ZNV/Rpts - ZNF genes & repeats, Het - Heterochromatin, TssBiv - Bivalent/Poised TSS, BivFlnk - Flanking Bivalent TSS/Enh, EnhBiv - Bivalent Enhancer, ReprPC - Repressed PolyComb, ReprPCWk - Weak Repressed PolyComb, Quies - Quiescent/Low. Boxplots were defined by minima = 25th percentile - 1.5X interquartile range (IQR), maxima = 75th percentile + 1.5X IQR, center = median, and bounds of box = 25th and 75th percentile. Both Strand-seq and MNase-seq assays measured NO in all fifteen chromatin states. Among these chromatin states, Strand-seq and MNase-seq revealed the highest NO signals on average for the polycomb repressed state and the bivalent enhancer state; whereas the lowest average NO signals were consistently seen for the active transcription start site (TSS) state.

We hypothesized that NO patterns derived from MNase fragmentation during Strand-seq library preparation could represent a readout to allow functional characterization of SVs (Fig. 1a and Extended Data Fig. 1). To test this, we evaluated whether Strand-seq data revealed nucleosome positioning through comparison with bulk MNase-seq data. We used the NA12878 lymphoblastoid cell line (LCL), which has both datatypes available, and pooled 95 Strand-seq libraries (sequenced to a median of 540,379 mapped nonduplicate reads per single cell^31^; Supplementary Table 1), into a ‘pseudobulk’ track, allowing direct comparison with the corresponding MNase-seq dataset (sequenced to 19-fold genomic coverage^32^). We measured NO along the genome (Methods) and found Strand-seq and MNase-seq were highly concordant in terms of uniformity of coverage and inferred nucleosome positions at DNase I hypersensitive sites (Spearman’s *r* = 0.68) (Fig. 1b,c). Nucleosome positioning near the binding site of CTCF^26,28^ (a key chromatin organizer) closely matched between both assays (Fig. 1d and Supplementary Fig. 1), and estimated nucleosome repeat lengths^28^ were highly concordant (Supplementary Fig. 1). In addition, both assays measured NO in all 15 chromatin states identified by the Roadmap Epigenome Consortium^33^. Among these chromatin states, Strand-seq and MNase-seq revealed the highest NO signals on average for the polycomb-repressed state and the bivalent enhancer state, whereas the lowest average NO signals were consistently seen for the active transcription start site (TSS) state (Extended Data Fig. 2). This indicates that Strand-seq enables direct measurement of NO to reveal a ‘molecular readout’. We thus developed the scNOVA framework, which harnesses Strand-seq to measure NO genome-wide and couples this with SVs discovered in the same sequenced cell (Fig. 1a).

As Strand-seq resolves its measurements by haplotype^31^, we considered that haplotype-specific differences in NO (haplotype-specific NO) resulting from random monoallelic expression, germline SNPs and local effects of SVs could be harnessed for scNOVA. To assess the utility of haplotype-resolved NO, we phased 24,652,658 of 49,205,197 (50.1%) of the NA12878 Strand-seq read fragments, and pooled these reads to generate pseudobulk NO tracks for each chromosomal haplotype (denoted ‘H1’ and ‘H2’, respectively; Fig. 1b). Using the female-derived NA12878 cell line, we compared haplotype-specific NO to haplotype-resolved gene expression measurements from bulk RNA-seq data^34^ (Methods). We identified a significant increase of NO in gene bodies mapping to H1 compared with H2 across the X chromosome (adjusted *P* = 0.0012; Wilcoxon rank sum test), suggesting that H1 represents the inactive X chromosome. These data were consistent with haplotype-resolved gene expression measurements at loci subject to X-inactivation^35^, whereas genes escaping X-inactivation did not exhibit haplotype-specific NO (Fig. 1e,f and Supplementary Fig. 3). We also investigated whether Strand-seq data is informative of haplotype-specific NO at cis-regulatory elements (CREs), and identified a 1.4-fold enrichment for allele-specific CRE binding on the X chromosome (*P* = 0.015; hypergeometric test; based on 718 CREs with haplotype-specific NO genome-wide; 10% false discovery rate (FDR)) (Supplementary Fig. 2). Moreover, CREs with haplotype-specific NO were significantly over-represented near genes showing allele-specific expression in the genome (*P* < 0.0018, hypergeometric test; Supplementary Fig. 2). These data suggest that haplotype-specific NO, a signal directly obtained from Strand-seq datasets, reflects biological gene regulation patterns in the genome.

#### Cell-typing

**Extended Data Fig. 3:**
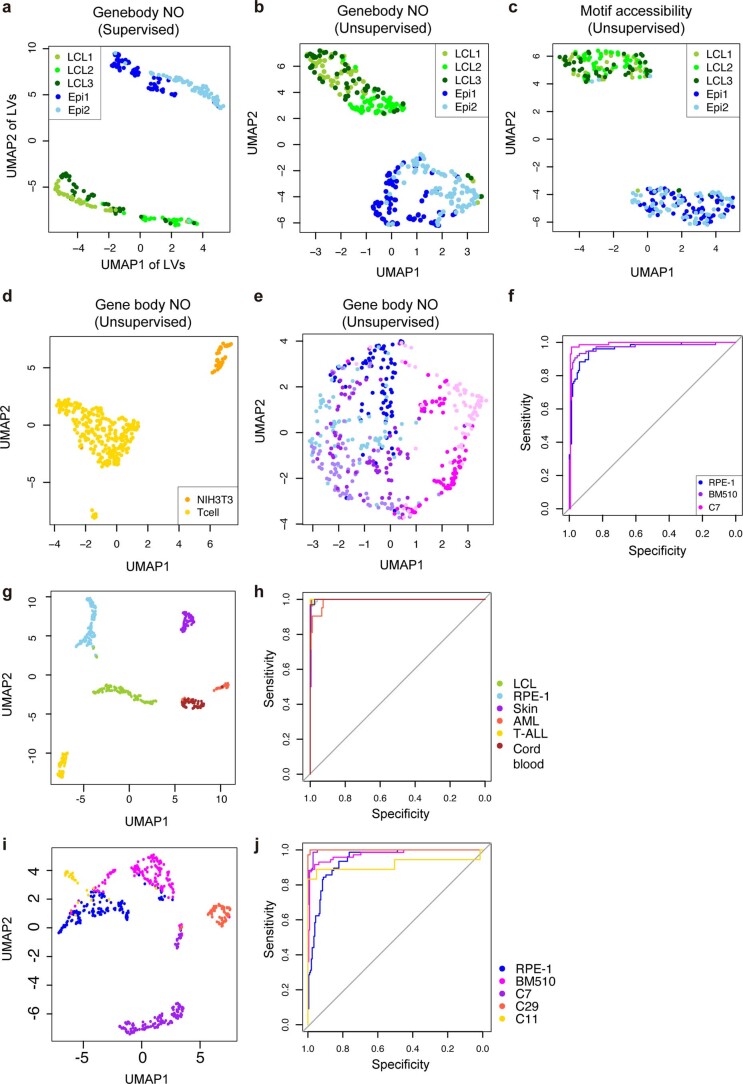
Utility of NO for cell-typing. **(a)** Cell-typing based on NO at gene bodies (AUC = 1). Epi1: RPE-1 replicate 1 (79 cells); Epi2: replicate 2 (77 cells); LCL1: HG01573 (46 cells); LCL2: HG02018 (50 cells), LCL3: NA19036 (50 cells); LV: latent variable. **(b)** UMAP visualization of Strand-seq libraries based on NO at gene-bodies (normalized by segmental ploidy status^24^). **(c)** We also explored dimensionality reduction of Strand-seq libraries based on DNA motif accessibility. Using the chromVAR package^110^, single-cell NO profiles for 2 kb DNase I hypersensitive sites (DHSs) were transformed into a deviation Z-score, which measures how likely a certain motif accessibility would occur when randomly sampling sets of peaks with similar GC content and read depth. For each single-cell, the deviation Z-score was calculated for 870 human TF motifs from the cisBP database^111^. These dimensionality reduction plots suggest that batch effect within the same cell type (three individuals in LCL, and two batches in RPE-1 sequenced separately) is minimal, and far less than the cell-type dependent variability. **(d)** UMAP using scMNase-seq^26^, including 45 NIH3T3 cells and 272 murine naive T cells, based on NO at the gene-bodies. **(e)** UMAP of RPE-1 (the originally commercially available cell line) and its transformed derived^37^ cell lines (BM510 and C7). Two biological replicates were sequenced for each cell line. **(f)** Receiver operating characteristic (ROC) using the PLS-DA based classifier. AUC for classifying each cell line was 0.9614, 0.9694, and 0.9892 for RPE-1, BM510, and C7 respectively. **(g-h)** Cell-typing for LCL, RPE-1, skin fibroblast, AML, T-ALL, and umbilical cord blood cells (**g**), and ROC curve depicting classification performance (overall AUC = 0.998) (**h**). (**i-j**) Cell-typing in five RPE-1 derived cell lines^37^ (RPE-1, BM510, C7, C29, and C11) (**i**), and ROC curve depiciting classification performance (overall AUC = 0.9648) (**j**).

Since NO within gene bodies reflects gene activity in MNase-seq data^28^, we hypothesized that Strand-seq based NO patterns could be used to infer gene expression. To investigate this, we tested whether NO globally reflects cellular gene expression patterns in the retinal pigment epithelium-1 (RPE-1) cell line, for which we previously generated both Strand-seq and RNA-seq data^24^. To profile NO globally, we pooled 33 million read fragments (including phased and nonphased reads) from 79 Strand-seq libraries into pseudobulk NO tracks. We identified an inverse correlation between NO at gene bodies and gene expression (*P* < 2.2 × 10^–16^; Spearman’s *r* of up to −0.24; Fig. 1g and Supplementary Fig. 4), where highly expressed genes showed significantly lower NO within their gene bodies (and vice versa). We next explored the utility of NO for cell-type inference (‘cell-typing’), based on the activity of lineage-specific genes, by implementing a multivariate dimensionality reduction framework. We performed in silico mixing of Strand-seq libraries from different LCLs and RPE cell lines, and built a classifier that separates distinct cell types by partial least squares discriminant analysis (PLS-DA). We used a training set of 179 mixed libraries, and initially considered 19,629 features, which reflect ENSEMBL^36^ genes with sufficient read coverage (Methods). After feature selection, 1,738 features were retained. We then used a nonoverlapping set of 123 cells to assess performance, all of which scNOVA classified accurately (area under the curve (AUC) = 1; Extended Data Fig. 3). Our framework also discriminated between cells from three related RPE cell lines derived from the same donor, which exhibit distinct SV landscapes^24,37^ (AUC = 0.96; Fig. 1h) indicating that scNOVA enables accurate cell-typing.

#### Gene activity changes between cell populations

**Extended Data Fig. 4:**
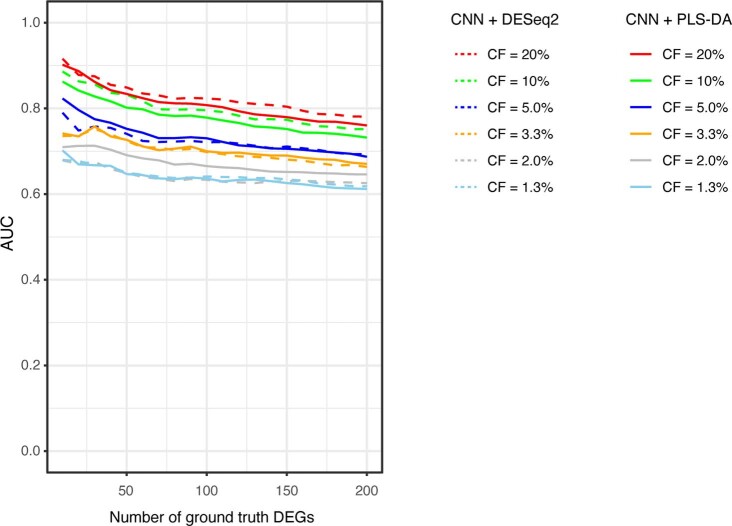
*In silico* downsampling experiments. We performed *in silico* cell mixing of RPE-1 and HG01573 cells to simulate application of scNOVA to different cell fractions (CFs). In this analysis six different CF ranges were considered (20, 10, 5, 3.3, 2, and 1.3). For each *in silico* cell mixing experiment, a total of 150 single cells were randomly subsampled for the major pseudo-clone (containing RPE-1 cells) and the minor pseudo-clone (HG01573 cells), by controlling the minor pseudo-clone CF at 20, 10, 5, 3.3, 2, and 1.3%, respectively. AUC, area under the curve. DEGs, differentially expressed genes. For each CF, we performed random subsampling of single-cell libraries 10 times, and depicted the respective mean AUC in the plot. Two different analysis modes - default (dashed lines, CNN with negative binomial generalized linear model), and alternative (solid lines, CNN with PLS-DA) are depicted. When the CF is larger than 10%, the default mode performs better, whereas for CFs smaller than 10%, the alternative mode outperforms the default mode.

Having established that scNOVA can use the expression of lineage-specific genes for cell-typing, we evaluated if it could predict gene expression differences between defined cell populations, such as subclones bearing distinct SVs. We devised a module that integrates deep convolutional neural networks and negative binomial generalized linear models (Supplementary Figs. 5 and 6), to measure differential gene activity between two defined cell populations. To benchmark this module, we mixed Strand-seq libraries from different cell lines in silico, creating ‘pseudoclones’, and evaluated the predicted changes in gene activity between defined pseudoclones (each composed of cells from one cell line) by analyzing NO at gene bodies (Supplementary Fig. 7 and Extended Data Fig. 4). We first compared RPE-1 to the HG01573 LCL line, and defined the ground truth of expression using RNA-seq. We found that the differential gene activity score of scNOVA (Methods) was highly predictive of the ten most differentially expressed genes, where analyses of pseudoclones comprising 156 RPE-1 and 46 HG01573 libraries revealed an AUC of 0.93 (we observed a similar performance when analyzing the 50 most differentially expressed genes; Fig. 1i). Gene activity changes inferred included well-known markers of epithelial (for example, *EGFR*, *VCAN*) and lymphoid (for example, *CD74*, *CD100*) cell types (Supplementary Table 2). The scNOVA predictions were informative also when we simulated minor subclones present with clonal frequency (CF) = 20%, CF = 5% and CF = 1.3%, resulting in AUCs of 0.92, 0.79 and 0.68, respectively (Extended Data Fig. 4). We obtained similar results when applying scNOVA to pseudoclones derived from different (genetically related) RPE cell lines (Supplementary Fig. 7). These benchmarking exercises suggest that scNOVA can accurately infer gene activity changes between defined cell populations, suggesting that this framework can be used to functionally characterize subclonal SVs.

### Functional outcomes of SVs in cell lines

To test this, we set out to investigate the functional outcomes of somatic SV landscapes in a panel of LCL samples^38^ (*N* = 25) from the 1000 Genomes Project^39^ (1KGP). Single-cell SV discovery in 1,372 Strand-seq libraries generated for this panel (Supplementary Table 1) discovered 205 somatic SVs, with 24 of 25 (96%) LCLs showing at least one SV subclone—a sevenfold increase compared to a previous report^40^ (Supplementary Table 3 and Supplementary Data). Of all the cell lines, 13 (52%) contained an SV subclone above 10% CF. This included the widely used NA12878 cell line^34,39^, in which we discovered a subclonal 500 kb deletion at19q13.12 (CF = 21%) that was mutually exclusive with two 22q11.2 deletions seen at CFs of 21% and 57%, respectively (Supplementary Figs. 9 and 10). The 22q11.2 SVs mapped to the well-known site of IGL recombination occurring during normal B cell development^41^. We hence focused on the 19q13.12 event, which resulted in the loss of a copy of *ZNF382*—a tumor suppressor and repressor of c-Myc^42^. Application of scNOVA measured significantly increased activity of *ERCC6*—a target gene of the c-Myc/Max transcription factor (TF) dimer^43^—and decreased activity of *PIEZO2* and *TRAPPC9*, in cells harboring this deletion (10% FDR; Supplementary Table 2).

To validate these findings, we reanalyzed Fluidigm and Smart-seq single-cell RNA-seq (scRNA-seq) datasets generated for NA12878 (refs. ^44,45^). We employed several established tools for SCNAs discovery from scRNA-seq data^46–48^ (Supplementary Table 4), all of which failed to discover any of the SV subclones seen in this cell line (Supplementary Table 4). Yet, upon directly inputting the respective SV breakpoint coordinates into the CONICSmat tool^46^, we succeeded in identifying the 19q13.12 deletion (denoted ‘19q-Del’) through ‘targeted SCNA recalling’. We next pursued differential gene expression analyses by scRNA-seq, comparing 19q-Del cells to unaffected (‘19q-Ref’) cells, and verified overexpression of *ERCC6* in 19q-Del cells (10% FDR; Supplementary Fig. 10). For *PIEZO2* and *TRAPPC9*, the scRNA-seq-based expression trends were consistent with scNOVA (Supplementary Fig. 10), but did not reach the FDR threshold. A search for the over-represented TF targets amongst the differentially active genes identified c-Myc and Max as the most over-represented TFs in 19q-Del cells (10% FDR; Supplementary Fig. 10). These results indicate that scNOVA can functionally characterize SVs inaccessible to scRNA-seq-based SCNA discovery.

**Fig. 2:**
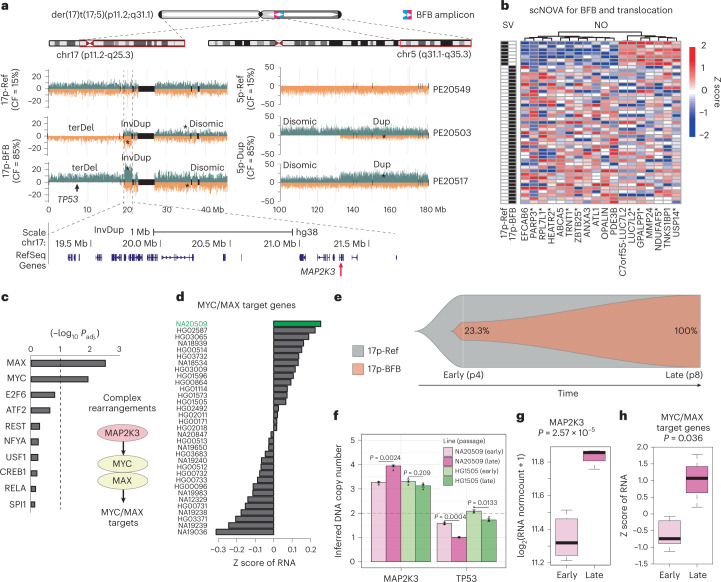
Linking subclonal SVs to their functional consequences in LCLs. **a**, Complex SVs in NA20509, with BFB-mediated rearrangements (17p) and a terminal dispersed duplication (5q) present with CF = 85%, shown for representative single cells. Ref, cells lacking complex SVs; InvDup, inverted duplication; terDel, terminal deletion. Reads are mapped to the W (orange) or C (green) strand. Gray, single cell IDs. **b**, Heatmap of 18 genes with altered gene activity amongst subclones, based on scNOVA (‘17p-BFB’, SV subclone; ‘17p-Ref’, 17p not rearranged). Asterisks denote TF targets of c-Myc and Max. **c**, Gene set over-representation analysis for TF target genes showing significant enrichment of c-Myc and Max targets in the 17p-BFB subclone. *P*_adj._, adjusted *P* value. Right, Model for c-Myc/Max target activation in NA20509 based on scNOVA, combined with previous knowledge. **d**, Mean RNA-seq expression *Z* scores of c-Myc/Max target genes across 33 LCLs. **e**, Fishplot showing CF changes over long-term culture from 23.3% (7 of 33 cells; p4) to 100% (30of 30 cells; p8). **f**, qPCR verifies clonal expansion of the BFB clone in p8 compared with p4 (*P* value based on FDR-corrected two-sided unpaired *t*-tests; *n* = 3). HG1505, control cell line with a somatically stable *MAP2K3* locus. Note that for both NA20509 and HG1505 the germline copy number of the *MAP2K3* locus was consistently estimated to be three. Data are presented as mean values ± s.e.m. **g**, RNA-seq shows significant increase of *MAP2K3* at p8 versus p4 (FDR-corrected two-sided Wald test, based on DESeq2; *n* = 5 and three biological replicates for p4 and p8, respectively). **h**, Mean RNA expression *Z* scores of c-Myc/Max target genes in NA20509 (differences between p4 and p8 were evaluated using a two-sided Wilcoxon rank sum test; *n* = 5 and three biological replicates for p4 and p8, respectively). Boxplot was defined by minima = 25th percentile – 1.5 × IQR, maxima = 75th percentile + 1.5 × IQR, center = median and bounds of box = 25th and 75th percentile (**g**–**h**).

We next focused on NA20509, the LCL with the most abundant SV subclone (85% CF). Somatic SVs in NA20509 arose primarily through the breakage-fusion-bride-cycle (BFB) process^24,49^ involving a 49 Mb terminal duplication on 5q, and a 2.5 Mb inverted duplication on 17p with an adjacent terminal deletion (terDel) (Fig. 2a). The 5q and 17p segments became fused into a derivative chromosome of around 115 Mb (Supplementary Fig. 13), which probably stabilized the BFB. We searched for global gene activity changes in this ‘17p-BFB’ subclone compared with the nonrearranged cells (‘17p-Ref’) and identified 18 dysregulated genes (Fig. 2b). Testing for gene set over-representation^50^ (Methods) revealed an enrichment of the target genes of c-Myc/Max heterodimers (10% FDR; Fig. 2c), that is, the same TFs we observed in the 19q-Del subclone in NA12878. Consistent with this, we identified somatic copy-number gain of *MAP2K3*, which encodes a gene activating c-Myc/Max^51^, resulting from the BFB (Fig. 2a).

We performed several orthogonal analyses to validate these findings. First, we verified all somatic SVs using deep WGS data generated for the 1KGP sample panel^52^ (Supplementary Fig. 13). Second, we analyzed RNA-seq data^38^ for this LCL panel, which revealed that NA20509 exhibits the highest *MAP2K3* expression and the highest c-Myc/Max target expression (Supplementary Fig. 14 and Fig. 2d). Third, we followed the 17p-BFB subclone in culture, by subjecting early (p4) and late passage (p8) cells to Strand-seq, which revealed outgrowth of the 17p-BFB subclone (CF = 23% at p4, CF = 100% at p8; *P* < 0.00001, Fisher’s exact test; Fig. 2e), suggesting these cells have a proliferative advantage. Quantitative real-time PCR experiments verified this clonal outgrowth pattern (Fig. 2f).

Since the functional impact of SVs on clonal expansion is unexplored in LCLs, we more deeply characterized the molecular phenotypes of 17p-BFB cells by pursuing RNA-seq in p4 and p8 cultures. We observed increased *MAP2K3* expression (1.39-fold, 10% FDR) at p8, consistent with *MAP2K3* dysregulation as a result of copy-number gain in the 17p-BFB subclone (Fig. 2g and Supplementary Note). Pathway-level analysis showed deregulation of c-Myc/Max target genes following clonal expansion (*P* = 0.036; Wilcoxon rank sum test; Fig. 2h and Supplementary Fig. 14). Collectively, these data link the outgrowth of SV subclones to the deregulation of c-Myc/Max targets, which could represent a common driver of clonal expansion in LCLs.

### Local effects of copy-balanced driver SVs in leukemia

**Fig. 3:**
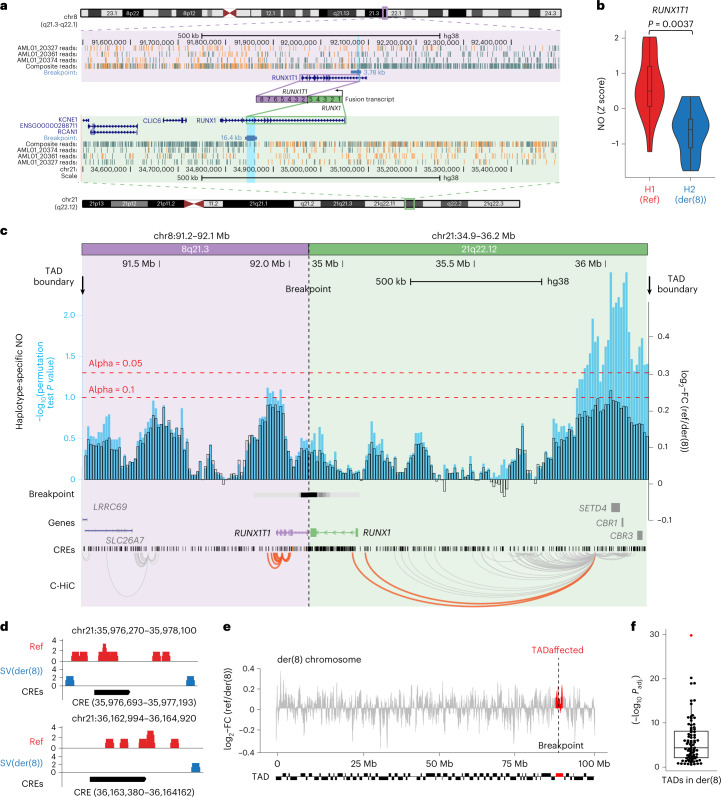
Haplotype-specific NO analysis shows local effects of a copy-neutral driver SV in AML. **a**, Balanced t(8;21) translocation in AML_1, discovered based on strand cosegregation (*P* value = 0.00003 for translocation discovery using strand cosegregation^24^, FDR-adjusted Fisher’s exact test; Supplementary Fig. 16). The SV breakpoint was fine-mapped to the region highlighted in light blue. Composite reads shown were taken from all informative cells in which reads could be phased (WC or CW configuration; Methods). **b**, A violin plot demonstrates haplotype-specific NO at the *RUNX1T1* gene body (10% FDR; two-sided Wilcoxon rank sum test followed by Benjamini–Hochberg multiple correction; *n* = 17 single cells; boxplot was defined by minima = 25th percentile – 1.5× IQR, maxima = 75th percentile + 1.5× IQR, center = median and bounds of box = 25th and 75th percentile), consistent with aberrant activity of the locus on der(8). **c**, Haplotype-specific NO around the SV breakpoint. FCs of haplotype-specific NO, measured between the *RUNX1-RUNX1T1* containing derivative chromosome (der(8)) and corresponding regions on the unaffected homolog (Ref), are shown in black, and –log_10_(*P* values) in light blue. Enhancer-target gene physical interactions based on chromatin conformation capture^56,93^ are depicted in orange (interactions involving *RUNX1* and *RUNX1T1*) and gray (involving other loci). **d**, Significant CREs located within the distal peak region, demonstrating haplotype-specific absence of NO on der(8) at 10% FDR, suggesting increased CRE accessibility on der(8). Within the segment around 0.8 to 1.1 Mb upstream of RUNX1, which showed pronounced haplotype-specific NO, we tested 69 CREs for haplotype-specific NO, which identified two significant CREs. **e**, Haplotype-specific NO measured between der(8) and corresponding regions of the unaffected homolog. Red, regions corresponding to the fused TAD. **f**, A beeswarm plot shows that the fused TAD (red) is an outlier in terms of haplotype-specific NO on der(8) (*P* values based on Kolmogorov–Smirnov tests; *n* = 83 TADs in der(8); boxplot was defined by minima = 25th percentile – 1.5× IQR, maxima = 75th percentile + 1.5× IQR, center = median and bounds of box = 25th and 75th percentile).

To deconvolute the effects of driver SVs in patients, we applied scNOVA to analyze the local consequences of balanced SVs, which are widespread in leukemia^3,53^. We analyzed primary cells from a patient with acute myeloid leukemia (AML) (32-year-old male; patient-ID = AML_1) bearing a balanced t(8;21) translocation that results in *RUNX1-RUNX1T1* gene fusion^54^. We sorted CD34^+^ cells from AML_1 (Supplementary Fig. 15), and sequenced 42 Strand-seq libraries. SV discovery revealed a 46,XY,t(8;21)(q22;q22) karyotype (Fig. 3a, Supplementary Fig. 16 and Supplementary Table 3) consistent with clinical diagnosis. We fine-mapped the translocation breakpoint to intron 1 of *RUNX1T1* and intron 5 of *RUNX1* (Supplementary Fig. 17), and subsequently identified haplotype-specific NO at 11 genes, genome-wide (10% FDR; Supplementary Table 2). This included *RUNX1T1*, which showed reduced NO on the derivative (H2) haplotype (Fig. 3b), consistent with increased gene activity mediated as a local effect of the translocation^55^. The remaining genes did not reside near a detected somatic SV, suggesting other factors (such as germline SNPs; Supplementary Fig. 17) may have affected their NO.

To systematically investigate potential local effects, we used a sliding window (Methods) to measure NO on both sides of the translocation breakpoint. We observed decreased NO, suggesting increased chromatin accessibility, from the breakpoint junction up to the respective nearest topologically associating domain (TAD) boundaries (Fig. 3c). This signal was most pronounced in an enhancer-rich region around 0.8 to 1.1 Mb upstream of *RUNX1* originating from chromosome 21 (*P* < 0.003; likelihood ratio test, adjusted using permutations; Fig. 3c), found to physically interact with the *RUNX1* promoter in CD34^+^ cells^56^. Within this segment, we identified two CREs with significantly reduced NO (10% FDR; Exact test) (Fig. 3d and Supplementary Table 5), which may foster *RUNX1-RUNX1T1* expression. Chromosome-wide analysis showed haplotype-specific NO patterns were restricted to the fused TAD (Fig. 3e,f), in line with these patterns resulting from the translocation.

**Extended Data Fig. 5:**
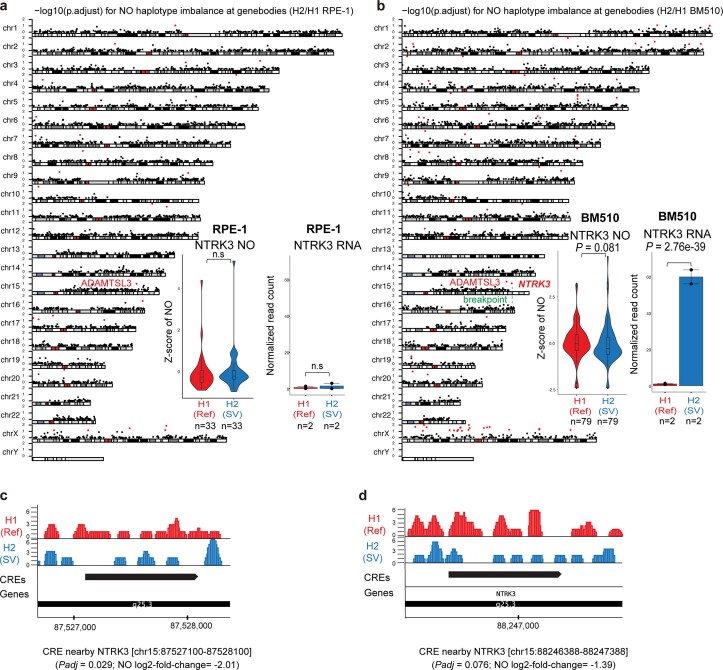
Haplotype-specific NO analysis in RPE-1 and BM510. (**a**, **b**) Haplotype-specific NO analysis of NO at gene bodies genome-wide in RPE-1 (**a**) and BM510 (**b**). For each chromosomal karyogram, the y-axis indicates the significance of haplotype-specific NO for each gene (-log10 p.adjust). All the significant genes were indicated in red dots (FDR 10%; two-sided wilcoxon rank sum test followed by Benjamini Hochberg multiple correction; derived from n = 33 cells and n = 79 cells for RPE-1 and BM510, respectively; Boxplots were defined by minima = 25th percentile - 1.5X interquartile range (IQR), maxima = 75th percentile + 1.5X IQR, center = median, and bounds of box = 25th and 75th percentile.). *NTRK3* (identified in BM510) is the only significant gene adjacent to an SV breakpoint. Haplotype-resolved RNA expression at the *NTRK3* locus is depicted using bar graphs in the right panel (two-sided likelihood ratio test followed by Benjamini Hochberg multiple correction; n = 2 biological replicates; Data are presented as mean values +/− SEM). (**c-d**) Haplotype-specific NO analysis at CREs. Browser track depicts the haplotype-resolved NO of the not rearranged (Ref) homolog in red, and the SV homolog in blue. scNOVA identified two CREs with significant haplotype-specific NO, including an intergenic CRE spanning chr15:87527100-87528100 (p.adjust = 0.029, log2-fold change = −2.01) (**c**) and an intronic CRE at chr15:88246388-88247388 (p.adjust = 0.076, log2-fold change = −1.39) (**d**).

**Extended Data Fig. 6:**
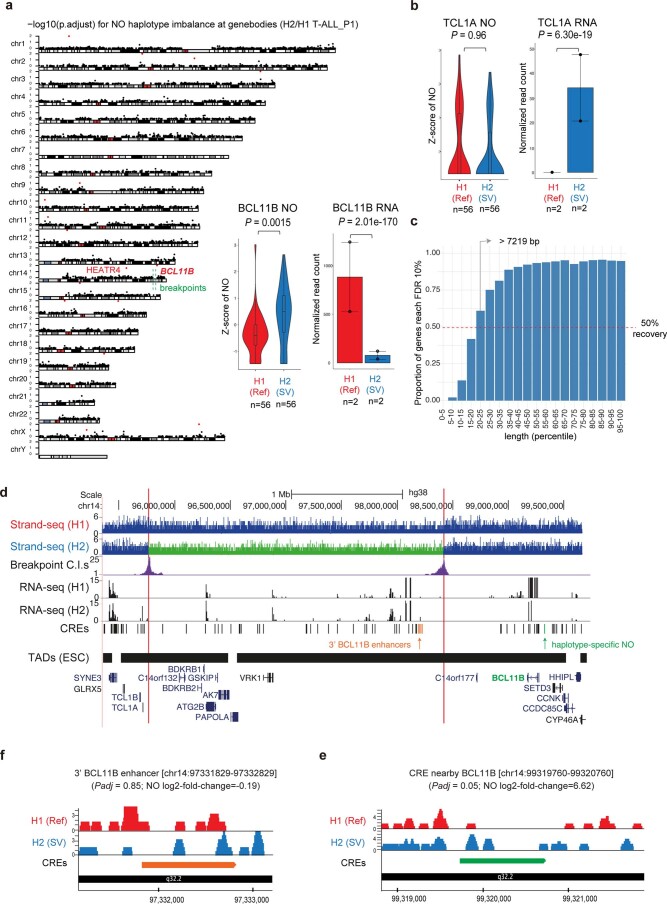
Haplotype-specific NO analysis in T-ALL_P1. (**a**) For each chromosomal karyogram, the y-axis indicates the significance of haplotype-specific NO at each gene (-log10 p.adjust). Genes with haplotype-specific NO are indicated using red dots (FDR 10%). An inlet figure depicts haplotype-specific NO (two-sided wilcoxon rank sum test and Benjamini Hochberg multiple correction; n = 56 cells) and RNA expression at the *BCL11B* gene locus (two-sided likelihood ratio test and Benjamini Hochberg multiple correction; n = 2 biological replicates), which has a nearby somatic SV (within 1 Megabase) and represents the (only) predicted local SV effect. (**b**) We did not measure haplotype-specific NO for *TCL1A* (two-sided wilcoxon rank sum test and Benjamini Hochberg multiple correction; n = 56 cells), a small gene with 4229 bp in size, in spite of its haplotype-specific gene expression^24^ (two-sided likelihood ratio test and Benjamini Hochberg multiple correction; n = 2 biological replicates). Boxplots were defined by minima=25th percentile-1.5X interquartile range (IQR), maxima=75th percentile+1.5X IQR, center=median, and bounds of box=25th and 75th percentile. For bargraphs, data are presented as mean values +/− SEM (**a-b**). (**c**) Simulation analysis revealed a minimum gene length (7219 bp) needed to robustly detect haplotype-specific NO at gene bodies, a gene length met by 80% of genes in the genome (Supplementary Notes**)**. (**d**) Inversion breakpoints and rearranged TADs. Known 3’ *BCL11B* enhancers^112^ are depicted in orange. In the not rearranged haplotype, they are located proximal to *BCL11B*, but in the inverted haplotype these enhancers they are located far away from *BCL11B*, and proximal to *TCL1A* in the different TAD boundary. (**e**) Application of scNOVA identified an intergenic CRE near the *BCL11B* with haplotype-specific NO. The browser track depicts the haplotype-resolved NO of the not rearranged (Ref) homolog in red and the SV homolog in blue. (**f**) The known 3’ *BCL11B* enhancer does not show significant haplotype-specific NO, but the inversion physically relocates these enhancers to the far distance from the *BCL11B*. A representative CRE is shown amongst four CREs overlapping with known 3’ *BCL11B* enhancers.

We also revisited Strand-seq datasets with previously reported copy-neutral SVs, including the BM510 cell line in which copy-neutral interchromosomal SVs resulted in *TP53*–*NTRK3* gene fusion^24^. In agreement with the oncogenic role of *TP53*–*NTRK3* (ref. ^24^), scNOVA identified *NTRK3* upregulation as the only significant local effect (10% FDR), consistent with allele-specific *TP53–NTRK3* expression measured on the rearranged haplotype (Extended Data Fig. 5). Second, we revisited a 2.6 Mb inversion mapping to 14q32 in a T-cell acute lymphoblastic leukemia (T-ALL) patient-derived xenograft (T-ALL_P1)^24^. scNOVA discovered downregulation of *BCL11B*, a known haploinsufficient T-ALL tumor suppressor^57^, as a significant local effect of this balanced inversion, supporting allele-specific silencing of *BCL11B* on the rearranged haplotype as measured by RNA-seq^24^ (Extended Data Fig. 6). These data collectively show that scNOVA allows linking balanced SVs to their local functional consequences—a functionality not provided by any previous single-cell multiomic method^20^.

### Dissecting functional effects of heterogeneous somatic SVs

**Fig. 4:**
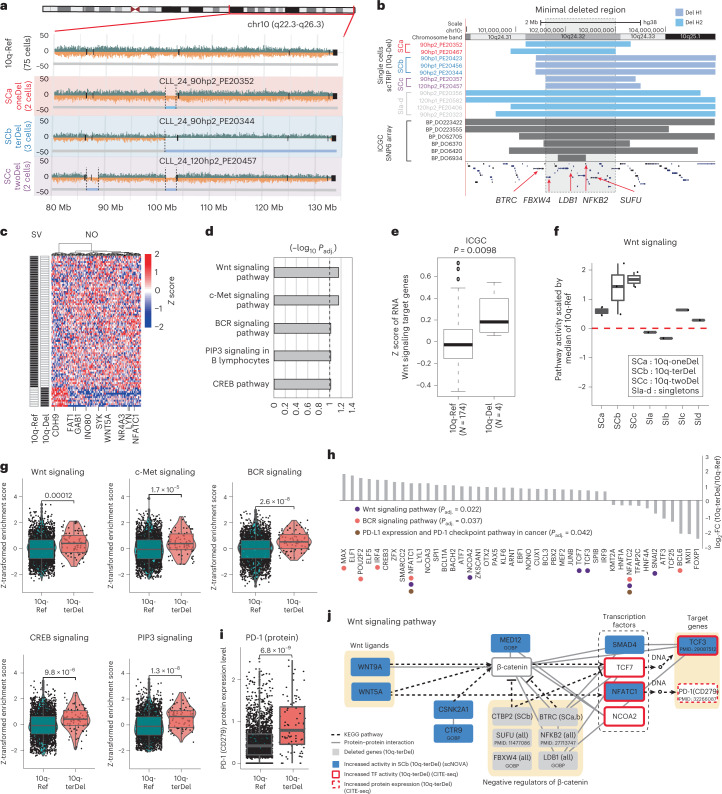
Deconvoluting consequences of subclonal SV heterogeneity in a CLL primary sample. **a**, Single-cell SV discovery in CLL_24. All cells exhibiting deletions (10q-Del) shown in Supplementary Fig. 18. 10q-Ref, cells bearing a not rearranged 10q. **b**, Minimal deleted region (chr10:101615000-103028000; hg38), displaying recurrent deletions in a separate cohort of CLLs^62^. **c**, Heatmap of genes with altered activity in 10q-Del based on scNOVA (alternative mode; 10% FDR). Genes from all significant pathways reported in **d** are highlighted. **d**, Pathway modules with differential activity, in cells exhibiting 10q-Del (10% FDR). **e**, Minimal deleted region-bearing CLL samples from the International Cancer Genome Consortium (ICGC) demonstrate overexpression of Wnt signaling genes compared with 10q-Ref (*P* = 0.0098; two-sided likelihood ratio test; *n* = 174 and *n* = 4 independent CLL samples for 10q-Ref and 10q-Del, respectively). **f**, Pathway activities ((–1) × *Z* score of NO) derived from jointly modeled NO at the gene bodies of Wnt signaling pathway genes for each SV-bearing CLL_24 cell. SIa-SId correspond to single cells exhibiting a deletion at 10q24 not shared by any other cell. *n* = 2, 3, 2 and 1 cells are depicted in the plot for SCa, SCb, SCc and SIa-SId, respectively. **g**, Single-cell gene set enrichment scores for five leukemia-related pathways from CITE-seq. Enrichment scores for 10q-terDel (*n* = 82) and 10q-Ref (*n* = 2,381) cells were compared using two-sided *t*-tests. **h**, Chart depicting 43 differentially active TFs between 10q-terDel and 10q-Ref cells based on DoRothEA^68^. Genes involved in the pathways over-represented by these TFs are annotated using colored dots. **i**, Differentially expressed surface protein CD279 (PD-1) in 10q-terDel (*n* = 82) compared with 10q-Ref (*n* = 2,381) cells based on a two-sided Wilcoxon rank sum test. **j**, Wnt pathway diagram showing the altered genes or TFs in SCb (10q-terDel) identified by scNOVA (blue nodes) and CITE-seq (red borders). Gray, known (see PubmedIDs) and computationally predicted regulators (based on Gene Ontology Biological Process (GOBP)) of Wnt signaling that are deleted in SCb. Throughout the figure, boxplots were defined by minima = 25th percentile – 1.5× IQR, maxima = 75th percentile + 1.5× IQR, center = median and bounds of box = 25th and 75th percentile.

We next set out to functionally dissect a leukemia sample with unknown genetic drivers, by characterizing B-cells from a 61-year-old patient with chronic lymphocytic leukemia (CLL) (CLL_24)^58^. Analysis of 86 Strand-seq libraries revealed an unprecedented level of somatic SVs, with 11 different karyotypes represented by 13 SVs occurring in subclones with CFs of 1–5% (Supplementary Table 3). This vastly exceeds intrapatient diversity estimates for CLLs from the Pan-Cancer Analysis of Whole Genomes (PCAWG), where maximally three subclones were reported^59^, highlighting how Strand-seq provides access to SVs escaping discovery by WGS^3,24^. Chromosome 10q showed especially pronounced subclonal heterogeneity; we identified seven partially overlapping deletions ranging from 2 to 31 Mb in size, and residing proximal to the fragile site *FRA10B*^60^ (Fig. 4a and Supplementary Fig. 18). These SVs clustered into a 1.4 Mb ‘minimal segment’ at 10q24.32, arising independently from both haplotypes (Fig. 4b). While previous studies reported somatic 10q24.32 deletions in 1–4% of CLLs^61–63^, molecular analysis of this recurrent somatic SV has so far been lacking.

We first compared all cells bearing a 10q24.32 deletion (‘10q-Del’, *N* = 11) to cells lacking such SV (‘10q-Ref’, *N* = 75), hence disregarding the fine-scale subclonal structure of CLL_24, and predicted 115 dysregulated genes (Fig. 4c and Supplementary Table 2). Next, we performed molecular phenotype analysis using MsigDB^64^ (Methods), which revealed that 10q-Del cells exhibit increased activity in several leukemia-relevant signaling pathways, including Wnt, c-Met (a pathway promoted by Wnt signaling^65^), B cell receptor (BCR) signaling, phosphatidylinositol (3,4,5)-trisphosphate (PIP3) signaling and the CREB pathway (10% FDR; Fig. 4d). RNA-seq data available for 178 CLLs^62^ and stratified by 10q24.32 status, revealed upregulation of Wnt and c-Met signaling—but not of BCR, PIP3 and CREB signaling—in CLLs exhibiting 10q24.32 deletions (10% FDR; CLLs with 10q-Del: *N* = 4; 10q-Ref: *N* = 174; Fig. 4e and Supplementary Fig. 24). These data therefore suggest a link between 10q24.32 deletion and the promotion of Wnt signaling.

We further tested whether the different 10q-Del events seen in CLL_24 subclones have led to distinct functional outcomes, focusing on three subclones represented by at least two cells: ‘SCa,’ showing one interstitial deletion directly at the minimal segment; ‘SCb,’ harboring a terDel, with the breakpoint located at the minimal segment boundary and ‘SCc,’ containing two interstitial deletions, at the minimal segment and at 10q23.31 (Fig. 4b and Supplementary Table 3). Molecular phenotype analysis of each subclone identified 109, 206 and 266 differentially active genes, respectively (Supplementary Table 2), with the most pronounced levels of Wnt upregulation in SCb and SCc (Fig. 4f). SCb showed the highest activation of c-Met, BCR and PIP3 signaling, whereas CREB signaling was highest in SCc (Supplementary Fig. 21). This suggests that deletion location and length at 10q24.32 affect their molecular consequences, and furthermore illustrates the ability of scNOVA to predict molecular differences in subclones represented by as few as two cells.

**Extended Data Fig. 7:**
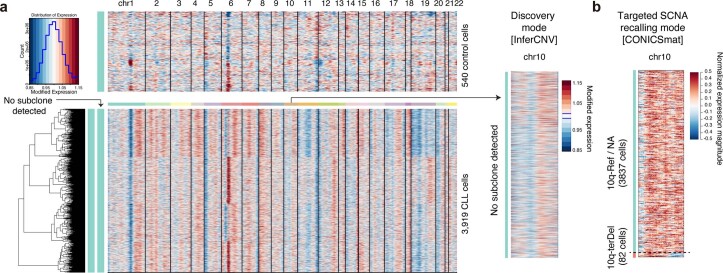
Inference of SCNAs using CITE-seq data from the CLL_24 sample. (**a**) InferCNV^48^ analysis of 3,919 high quality CLL cells, and 540 control cells (cells sequenced by CITE-seq not originating from the B-cell lineage; see Supplementary Fig. 25), profiled by CITE-seq. This analysis did not discover any subclones in CLL_24. (Note that the high variability observed on the 6p-arm, not only seen in CLL cells but also in control cells, likely arose from the presence of *MHC* genes in this locus, whose expression is cell cycle dependent^113^.) (**b**) CONICSmat based targeted SCNA recalling of the 10q-terDel (previously discovered in SCb; see Fig. 4b) using the high-resolution breakpoints derived from Strand-seq. Use of these SV breakpoints allowed CONICSmat to confidently call the 10q-terDel in 82 single cells from the CITE-seq data.

To more deeply characterize the CLL_24 subclones, we generated CITE-seq (cellular indexing of transcriptomes and epitopes by single-cell sequencing) data, which couples scRNA-seq with protein surface marker measurements^66^. Again, we attempted SCNA discovery in the scRNA-seq data, which failed to detect any SCNAs, or subclones, in CLL_24 (Supplementary Table 4). However, targeted SCNA recalling^46^ identified 82 CITE-seq cells harboring the greater than 31 Mb 10q-terDel of SCb (‘10q-terDel’), whereas the deletions in SCa (2.2 Mb) and SCc (2.1 Mb and 1.9 Mb, respectively) escaped detection (Extended Data Fig. 7 and Supplementary Notes). Having recovered the SCb subclone in the CITE-seq data, we performed single-cell gene set enrichment analysis^67^ (Methods), which verified that all pathways inferred by scNOVA (Wnt, c-Met, BCR, PIP3 and CREB) are upregulated in 10q-terDel cells (Fig. 4d,g). A gene regulatory network analysis^68^ comparing 10q-terDel with 10q-Ref cells identified 43 differentially active TFs (FDR 10%; Fig. 4h) and a functional enrichment analysis^69^ showed over-representation of Wnt signaling, BCR signaling and the PD-1 checkpoint pathway (Supplementary Table 16 and Fig. 4h); the PD-1 checkpoint pathway has been linked to immune resistance and transformation of CLL to aggressive lymphoma^70,71^. Since somatic lesions mediating PD-1 expression in CLL have remained elusive, we used the CITE-seq data to analyze PD-1 protein expression, which demonstrated upregulation of PD-1 in 10q-terDel-containing cells as the only significant hit at the protein level (Fig. 4i). Notably, *NFATC1*, a TF predicted to be differentially active by both scNOVA and CITE-seq, regulates Wnt^72^, PIP3 (refs. ^73,74^), CREB^75^ and BCR signaling^76^ as well as PD-1 expression^77^, and thus may contribute to global pathway dysregulation in CLL_24. Our analysis reveals subtle pathway activities of somatic deletions present at low CF (Fig. 4f,j), and collectively implicates 10q24.32 deletions in dysregulated Wnt signaling—a crucial pathway for CLL pathogenesis^78^.

### Functional characterization of subclonal chromothripsis

**Fig. 5:**
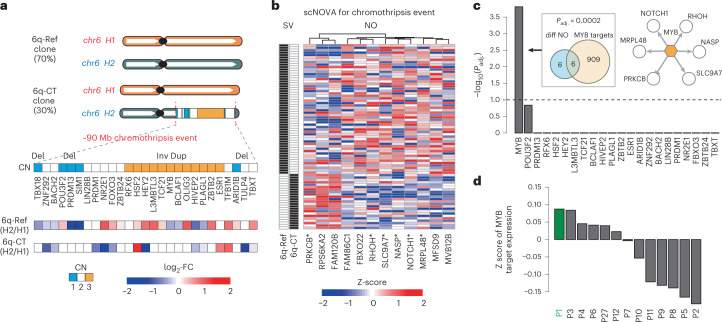
scNOVA identifies functional effects of a subclonal chromothripsis event. **a**, The 27 TF genes located in a segment that underwent chromothripsis^24^ on 6q in T-ALL_P1. Haplotype-specific NO measurements, which scNOVA generated for CREs assigned to the nearest genes, are depicted below. FC of normalized haplotype-specific NO is shown for each subclone. 6q-CT, subclone bearing chromothripsis on 6q; 6q-Ref, subclone bearing a not rearranged chromosome 6. **b**, Heatmap of 12 genes with differential activity between subclones in T-ALL_P1, based on scNOVA (denoted CT gene signature). Asterisks denote TF targets highlighted in **c**. **c**, TF target over-representation analyses for CT gene signature, revealing c-Myb as the only significant hit. Venn diagram depicting enrichment of c-Myb targets (*P* value based on an FDR-adjusted hypergeometric test). Upper right, network with c-Myb and its target genes based on scNOVA, combined with previous knowledge. **d**, Mean *Z* scores of c-Myb target gene expression measured by bulk RNA-seq in a panel of 13 T-ALL-derived samples. T-ALL_P1 (P1) exhibited the overall highest expression of c-Myb targets.

While chromothripsis is a widespread mutational process in cancer^3,4,22^, this process is not ascertained by previous single-cell multiomic methods, and its molecular outcomes remain largely elusive^3,79^. We previously discovered a subclonal chromothripsis event^24^ in T-ALL_P1 that affects most of 6q (denoted ‘6q-CT’; CF = 30%) (Fig. 5a and Supplementary Table 3); however, the consequences of this complex rearrangement were uncharacterized. Using scNOVA, we identified 12 genes with differential NO between 6q-CT and 6q-Ref cells (denoted the ‘CT gene signature’; 10% FDR; Fig. 5a,b and Supplementary Table 2). A closer analysis showed 27 TF genes overlapping the chromothriptic region (Fig. 5a). Gene set over-representation testing using the target genes of these TFs revealed that c-Myb, product of the *MYB* oncogene, was significantly enriched among the genes included in the CT gene signature (10% FDR; adjusted *P* = 0.00015; Fig. 5b,c and Supplementary Table 6). The *MYB* gene is located within a region that was duplicated (and inverted) as a result of 6q-CT, suggesting a potential dosage effect (Fig. 5a). Corroborating these predictions, we performed RNA-seq in a panel of 13 T-ALLs, amongst which T-ALL_P1 showed the highest expression of c-Myb targets (Fig. 5d and Supplementary Table 7). We also verified that *MYB* is allele-specifically expressed from the SV-affected haplotype (*P* = 0.0317; likelihood ratio test; Supplementary Fig. 30), which together nominates *MYB* as a candidate driver gene dysregulated as a consequence of 6q-CT.

**Fig. 6:**
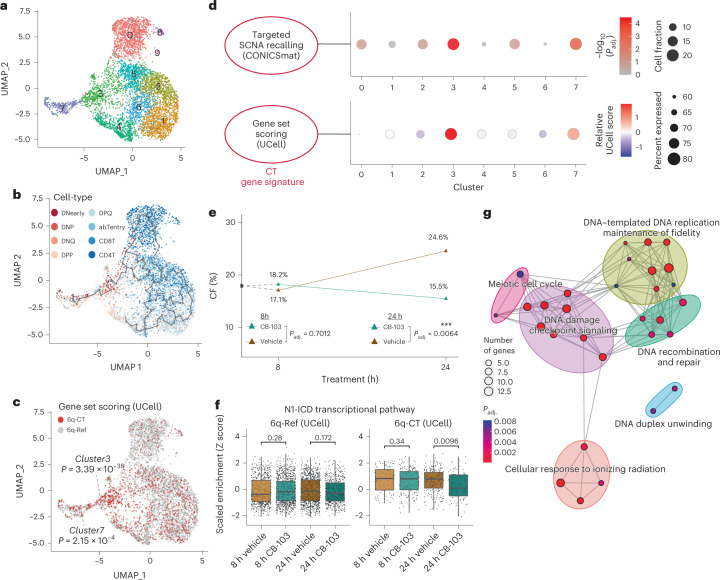
Targeting the chromothriptic subclone in cell culture. **a**, Uniform manifold approximation and projection (UMAP) of scRNA-seq data showing ten unsupervised clusters in T-ALL_P1. **b**, Overlay of gene set-derived cell-type annotation and inferred lineage trajectory onto this UMAP. **c**, Single cells whose expression profiles matched the CT gene signature (gene set UCell score > (median score + s.d.)) are assigned to ‘6q-CT’ and shown in red; the remaining cells did not meet the threshold for the CT gene signature (assigned ‘6q-Ref’ status’). *P* values depict enrichment of 6q-CT cells in clusters 3 and 7. **d**, Significant enrichment of 6q-CT cells in clusters 3 and 7 based on scRNA-seq. Upper panel, dot plot showing the significance of over-representation of 6q-CT calls in scRNA clusters based on targeted SCNA recalling (*P* values based on FDR-adjusted Fisher’s exact tests). Lower panel, gene set-level expression summary for the CT gene signature, derived using UCell^81^ with the directionality of expression changes taken into account. **e**, CF of 6q-CT cells after treatment with Notch inhibitor CB-103 (green) and vehicle control (brown) along a time course 8 h before and 24 h after treatment. CF was estimated by transferring gene set based CT annotations obtained from the scRNA-seq of T-ALL_P1 before treatment to the scRNA-seq of T-ALL_P1 after treatment. Changed CF (%) at 24 h compared with 8 h is shown in the plot on top of the 24 h datapoints. For each timepoint, the difference of CF under vehicle and CB-103 was evaluated by Fisher’s exact test (results are based on pairwise comparisons). **f**, Scaled enrichment scores obtained by single-cell gene set enrichment analysis for the ‘N1-ICD transcriptional pathway’ gene set. Scores across treatment conditions (vehicle versus CB-103) were compared using two-sided Wilcoxon rank sum tests. (Boxplot was defined by minima = 25th percentile – 1.5× IQR, maxima = 75th percentile + 1.5× IQR, center = median and bounds of box = 25th and 75th percentile; *n* = 665, 978, 915 and 556 cells for 6q-Ref from 8 h Vehicle, 8 h CB-103, 24 h Vehicle and 24 h CB-103; *n* = 91, 157, 213 and 88 cells for 6q-CT for each condition, respectively.) **g**, Network representation of GOBPs enriched by differentially expressed genes in 6q-CT compared with 6q-Ref cells under CB-103 treatment (24 h), subtracting any genes not specific to the drug treatment.

To more deeply characterize this sample, we generated scRNA-seq data for T-ALL_P1 (5,504 cells; Fig. 6a). Since scRNA-seq-based SCNAs discovery^46–48^ missed the 6q-CT event (Supplementary Table 4), we again performed targeted SCNA recalling (Supplementary Notes) generating confident calls for 838 (around 15%) cells in the scRNA-seq dataset (the remaining 4,666 cells lacked a confident assignment; ‘NA’). Out of these 838 cells, 729 were predicted to harbor the 6q-CT event, and 109 were called 6q-Ref. Unsupervised clustering^80^ of the scRNA-seq data stratified by 6q status (Methods) revealed that 6q-CT cells (as predicted through targeted recalling) were enriched in two expression clusters (clusters 3 and 7; *P* = 3.43 × 10^–5^ and 1.15 × 10^–3^; FDR-adjusted Fisher’s exact test; Fig. 6d and Supplementary Fig. 34), in line with a distinctive expression profile. To corroborate this, we applied UCell^81^ to assign cells into ‘6q-CT’ or ‘6q-Ref’ based on the CT gene signature, which confirmed enrichment of 6q-CT in clusters 3 and 7 (Fig. 6c,d; *P* = 3.39 × 10^–38^ and *P* = 2.15 × 10^–4^; FDR-adjusted Fisher’s exact test). Trajectory analysis^82^ showed the 6q-CT cells (as defined by UCell) were enriched for DNearly (double-negative early; *P* = 2.78 × 10^–13^), DNQ (double-negative quiescent; *P* = 1.27 × 10^–5^) and DPP (double-positive proliferating; *P* = 1.88 × 10^–7^) T cells (FDR-corrected Fisher’s exact tests; Fig. 6b and Supplementary Fig. 35), and depleted of mature CD4^+^ T cells (*P* = 1.45 × 10^–11^, Supplementary Fig. 35). This suggests a potential differentiation block at the progenitor stage as a result of 6q-CT and, more generally, that 6q-CT cells bear a distinctive molecular phenotype as a result of the chromothriptic rearrangements.

Having identified c-Myb pathway activation as a consequence of 6q-CT in TALL_P1, we hypothesized this molecular phenotype could guide drug targeting in cell culture. We selected *NOTCH1* as a suitable candidate for targeting this subclone because this c-Myb target was (1) inferred by scNOVA to be highly upregulated in 6q-CT cells (Fig. 5b) and (2) is targetable by different compounds and strategies^83^. We treated T-ALL_P1 cell cultures with the CB-103 pan-NOTCH small-molecule inhibitor (targeting the *Notch1* intracellular domain (N1-ICD)^84,85^) or a vehicle control for 8 h and 24 h (Methods). Using scRNA-seq (3,663 single cells) to analyze drug response patterns, we inferred 6q-CT and 6q-Ref cells at each timepoint by transferring the cell annotation labels from the untreated (reference) sample with Seurat^80^ (Fig. 6c and Supplementary Fig. 37). After 24 h in culture, vehicle-treated T-ALL_P1 cells showed a 45% relative increase in the 6q-CT subclone compared to 8 h (CF of 17.1% to 24.6%; *P* = 0.0180; FDR-adjusted Fisher’s exact test), indicating that 6q-CT cells expanded clonally. By contrast, upon CB-103 treatment, the CF of the 6q-CT subclone was reduced at 24 h (to CF = 15.5%; *P* = 0.0064; Fig. 6e and Supplementary Fig. 38), indicating that 6q-CT cells were preferentially lost with N1-ICD inhibition. Additionally, we observed specific depletion of the REACTOME N1-ICD gene set only in 6q-CT cells after 24 h of CD-103 treatment, consistent with specific subclone targeting (*P* = 0.0096; FDR-adjusted Wilcoxon rank sum test; Fig. 6f and Supplementary Fig. 39). These results highlight the potential of scNOVA to functionally characterize highly complex classes of DNA rearrangement (that is, chromothripsis events), and to clinically target subclones bearing complex cancer driver SVs.

## Discussion

The functional characterization of SVs is of critical importance for precision oncology^1–3^. Our method characterizes a wide spectrum of SV classes^24^, and couples these with NO analysis to link somatic SVs to local or global gene activity changes. Accounting for balanced SVs, scNOVA allows the investigation of copy-number stable (that is, euploid) malignancies previously inaccessible to single-cell multiomics^3,20^ (Supplementary Table 12). Strand-seq derived SCNA calls were far better resolved compared to scRNA-seq based calls (Supplementary Table 4), suggesting a more limited utility of scRNA-seq data for discovering SCNA drivers in cancer, with the exception of malignancies displaying extremely high levels of chromosomal instability with particularly large-scale SCNAs^3,86^.

We uncovered unprecedented karyotypic diversity in a CLL sample, comprising distinct deletions at 10q24.32, which we link to leukemia-related signaling pathways, particularly Wnt signaling. Read depth based profiling of SCNAs is prone to underreport such subclonal structural diversity^3^. Enrichment of cases bearing 10q24.32 deletions amongst relapsed/refractory and high-risk CLL^87^ suggests a potential role of Wnt pathway dysregulation mediated through 10q24.32 in disease progression. Whether the *FRA10B* fragile site is involved in the formation of these deletions remains to be seen and requires larger cohorts. Interestingly, CLL_24 exhibits a SNP (rs118137427; 3.7% allele frequency in Europeans) within *FRA10B* associated with the acquisition of 10q-terDel in normal blood^88^. Based on the PCAWG resource comprising 94 CLLs^2^, rs118137427 is seen in 2 out of 4 (50%) CLLs with 10q24.32 deletions, but in only 6 of 90 (6.7%) CLLs with 10q-Ref (*P* = 0.035; Fisher’s exact test), suggesting a possible link between SNPs at *FRA10B* and ITH in leukemia that warrants future investigation.

Our framework readily functionally characterizes complex rearrangements previously inaccessible to single-cell multiomics^3^. Complex somatic SVs are prevalent in cancer and linked with aggressive tumor phenotypes^2,3,22^ underlining significant potential of scNOVA for the comprehensive functional characterization of cancer cells. Since scNOVA does not require coupling distinct experimental modalities in each individual cell, it overcomes important methodological challenges^20^, including data sparseness and higher costs from generating data for more than one modality^20,89^. Additionally, the coverage achieved by Strand-seq enables the analysis of haplotype-specific NO along the entire genome (Supplementary Fig. 41), providing advantages over classical allele-specific analyses that are restricted to regionally phased SNPs^15^.

Nonetheless, important challenges remain, and the full spectrum of mutations arising in an individual cell is likely to remain inaccessible to a single method in the foreseeable future. Strand-seq does not capture SVs less than 200 kb that more rarely acts as cancer drivers^2^. Additionally, while scNOVA infers differentially active genes, it does not span the same dynamic expression range as scRNA-seq (Supplementary Table 12). This suggests that pairing scNOVA with targeted SCNA recalling by scRNA-seq can provide added value by allowing variants outside the detection range of other methods to be characterized. Finally, Strand-seq requires dividing cells for BrdU labeling^23^ (Fig. 1a), and is therefore not applicable for nondividing cells or fixed samples. However, it can be used for dividing cells in organoids, primary fresh frozen progenitor cells, cells in regenerating tissues and cancer samples amenable to culture. Our study used cell lines for benchmarking followed by proof-of-principle application in patient samples. Generalization of these analyses to larger cohorts will allow systematic investigation of the roles subclonal SVs play in leukaemogenesis.

We foresee a wide variety of potential future applications. Our framework offers potential for studies on the determinants and consequences of chromosomal instability in cancer, and may promote research into the interplay of genetic and nongenetic cancer determinants^20^. It likewise could be used to advance surveys of precancerous lesions^3,90^. Additionally, scNOVA may offer value in precision oncology by exposing subclonal driver alterations along with their targetable functional outcomes, to target cancer subclones in patients. Furthermore, SVs can accidentally arise in key model cell lines, as we demonstrate for widely used LCLs, and the features of scNOVA are ideally suited to functionally characterize unwanted heterogeneity in such samples. Unwanted somatic SVs also arise as a by-product of CRISPR-Cas9 genome editing, which generates micronuclei and chromosome bridges in human primary cells, structures that initiate the formation of chromothripsis^91^. scNOVA could promote the safety of therapeutically relevant genome editing in the future, by enabling the simultaneous detection and functional characterization of such potentially pathogenic editing outcomes.

In summary, scNOVA moves directly from SV landscapes to their functional consequences in heterogeneous cell populations. By making a broad spectrum of somatic SVs accessible for functional characterization genome-wide, this single-cell multiomic framework serves as a foundation for deciphering the impact of somatic rearrangement processes in cancer.

## Methods

### Strand-seq library preparation

NA20509 Strand-seq libraries were prepared as previously described^94^. Strand-seq libraries of primary leukemia samples were generated as follows: peripheral blood mononuclear cells of a previously untreated female CLL patient (routine diagnostics: *IGHV* unmutated, no *TP53* mutation, no detected alteration in 6q21, 8q24, 11q22.3, 12q13, 13q14 and 17p13) were isolated after obtaining informed consent. Cells were isolated and cultured using previously established protocols^95^. CLL cells were cultured at 1 × 10^6^ cells ml^–1^ in Roswell Park Memorial Institute (RPMI) medium (Gibco by Life technologies), supplemented with 10 % human serum (PAN BIOTECH), 1% Pen/Strep (GIBCO by Life Technologies) and 1% Glutamine (GIBCO by Life Technologies). Cells were stimulated with 1 µg ml^–1^ Resiquimod (Enzo) and 50 ng ml^–1^ IL-2 (Sigma). BrdU (40 µM; Sigma) was incorporated for 90 h and 120 h, respectively, to perform nontemplate strand labeling. Single nuclei from each timepoint were sorted into 96-well plates using a BD FACSMelody cell sorter, followed by Strand-seq library preparation (described below). In the case of the AML sample, frozen primary mononuclear cells from a bone marrow aspirate were thawed and stained with CD34-APC (clone 581; Biolegend), CD38-PeCy7 (clone HB7; eBioscience), CD45Ra-FITC (clone HI100; eBioscience), CD90-PE (clone 5E10; eBioscience) and LIVE/DEAD Fixable Near-IR Dead Cell Stain (Thermofisher). Single, viable, CD34^+^ cells (Supplementary Fig. 15) were sorted using a BD FACSAria Fusion Cell Sorter into ice-cold serum-free expansion medium (SFEM) supplemented with 100 ng ml^–1^ SCF and Flt3 (Stem Cell Technologies), 20 ng ml^–1^ IL-3, IL-6, G-CSF and TPO (Stem Cell Technologies). Cells were plated in Corning Costar Ultra-Low Attachment 96-well flat-bottom plates (Sigma) at 1 × 10^5^ cells ml^–1^ in warm medium as above. At 24 h after culture, 40 µM BrdU was added. Nuclei were isolated after 43 h total culture time, and BrdU-incorporating nuclei sorted into 96-well plates followed by Strand-seq library preparation. All Strand-seq libraries were automatically prepared using a Biomek FXP liquid handling robotic system, as described previously^23,96^. Libraries were sequenced on an Illumina NextSeq 500 sequencing platform (MID-mode, 75 base pair (bp) paired-end sequencing protocol).

### Strand-seq data preprocessing

Reads from Strand-seq (fastq) libraries were aligned to the hg38 assembly using BWA^97^, as previously described^24^. Sequence reads with low quality (MAPQ < 10), supplementary reads and duplicated reads were removed. Single-cell library selection was performed as described previously^24^. The single-cell footprints of different SV classes were discovered using the principle of scTRIP of Strand-seq data using the MosaiCatcher computational pipeline with default settings^24^.

### Coupling NO measurements and SV discovery in the same cell with scNOVA

We developed scNOVA as a computational framework for coupling discovered somatic SVs with analyses of NO profiles in the same cell. The scNOVA workflow covers a set of different operations from single-cell SV discovery (using the previously described scTRIP method^24^) to NO profiling at CREs, and gene as well as pathway dysregulation inference based on NO at gene bodies, and can be used in a haplotype-aware or -unaware manner (Extended Data Fig. 1). To maximize reusability, interoperability and reproducibility we combined all scNOVA modules into a coherent workflow using snakemake. Alternatively, these modules can be executed individually.

#### Data analysis and operational definition utilized for NO

We operationally defined NO closely following definitions from a previous study^28^: NO maps were calculated by counting how many reads from the Strand-seq libraries (which typically comprise mono-nucleosomal fragments around 140–180 bp in size; see Supplementary Table 1 and Supplementary Fig. 1) covered a given bp based on aligning reads to the GRCh38 (hg38) genome assembly with BWA^97^. Genomic regions with unusual (such as artificially high) coverage were considered artifacts, and were automatically excluded (‘blacklisted’) by our Strand-seq analysis workflow as previously described^24^. No further peak calling or smoothing was conducted, and no assumptions on the length of the nucleosomal DNA were made to derive NO maps, as nucleosome boundaries were determined on both sides of the nucleosome by paired-end sequencing^28^. For the calculation of NO around bound CTCF binding sites (downloaded from ENCODE^34^), the averaged profile was scaled^28^ to yield an NO equal to 1 at position –2,000 bp from the center of the bound CTCF site.

#### Cell type classification

We generated feature sets from the NO at the body of genes (defined as the region from the TSS to the transcription termination site, which includes exons and introns) at the single-cell level. When several sequencing batches from the same samples were available, we applied batch correction to the NO count matrix using ComBat-seq^98^. NO in gene body regions was normalized by segmental copy number status, and by library size to obtain reads per million, which we transformed into log_2_ scale. This feature set was used for the unsupervised dimension reduction plot (Extended Data Fig. 3) and for training of a supervised classification model based on PLS-DA^99^.

##### Haplotype-phasing of single-cell NO tracks

As previously described, Strand-seq directly resolves its underlying sequence reads onto haplotypes ranging from telomere to telomere^31^ (chromosome-length haplotyping). scNOVA phases NO profiles onto a chromosomal homolog using the StrandPhaseR algorithm^31^, which is employed wherever the template strand segregation pattern of a chromosome enables unambiguous haplotype-phasing, that is, for Watson/Crick (WC) or Crick/Watson (CW) template state configurations in Strand-seq libraries^31,96^. Haplotype-specific analyses pursued by scNOVA employ phased reads (normalized by locus copy number), whereas the inference of gene activity changes uses both phased reads (from chromosomes with a WC or CW configuration) and unphased reads (from chromosomes with a CC or WW configuration^31,96^).

##### Inference of haplotype-specific NO and identification of local effects of SVs

To dissect local effects of SVs, the scNOVA framework performs a genome-wide haplotype-specific NO analysis at gene bodies in pseudobulk, which yields a haplotype-specific NO matrix. Using this matrix, scNOVA then scans up to ±1 Mb around each somatic SV breakpoint to infer local effects of these breakpoints on haplotype-specific gene activity, using FDR-adjusted Wilcoxon rank sum tests. Once a local effect on gene activity is identified, scNOVA additionally provides the option to locally scan for CREs exhibiting haplotype-specific NO. To do so, user-provided CRE positions from the cell type of interest are used by scNOVA to calculate haplotype-specific NO at CREs, and the Exact test (10% FDR) is used for significance testing.

##### Inference of genome-wide changes in gene activity

This haplotype-unaware module of scNOVA considers all reads—whether phased or not—to infer gene activity alterations via analysis of differential patterns of NO along gene bodies. scNOVA obtains gene loci from ENSEMBL (GRCh38.81), converted into bed format (Genebody_hg38.81.bed). Strand-seq reads falling within the start and end position of genes (Genebody_hg38.81.bed) were identified with the Deeptool multiBamSummary function^100^, using the following parameters: [multiBamSummary BED-file –BED Genebody_hg38.81.bed –bamfiles Input.bam –extendReads –outRawCounts output.tab -out output.npz]. The scNOVA gene dysregulation inference module contains two steps: Step 1 filters out genes unlikely to be expressed (‘not expressed’, NEs), whereas Step 2 infers dysregulated (that is, differentially expressed) genes between subclones using a generalized linear model.

In Step 1, scNOVA first aims to infer gene expression ‘On’ and ‘Off’ states^101^ from NO, by analyzing NO as well as gene context-specific sequence features along gene bodies using deep convolutional neural networks^102^ (CNNs).

By default, scNOVA operates with the model trained with a pseudobulk of 80 cells, to estimate the probability of each gene to represent an NE in each clone. Genes likely to be unexpressed (NE status probability ≥0.9) across clones are filtered out in Step 1, and all remaining genes used in Step 2.

In Step 2, scNOVA by default employs negative binomial generalized linear models, available in the DESeq2 algorithm^103^, to infer genes with differential activity between individual cells or clones. As an input, scNOVA computes single-cell count tables of gene body NO. When running this step with subclones, all individual cells of the subclone are considered ‘replicates’ in DESeq2 terminology^103^. Subclones (or cells) are compared in a pairwise manner using a two-sided Wald test to infer genome-wide alterations in gene activity. Based on this, we defined the differential gene activity score as the sign of the fold change (FC) in NO at gene bodies, multiplied by –log_10_ *P* values. Genes with significantly altered activity were identified using a 10% FDR threshold. Additionally, to facilitate the analysis of small CF subclones, scNOVA provides an alternative mode which employs PLS-DA^99^ to identify discriminatory feature sets as gene sets showing altered activity. To do this, scNOVA builds a PLS-DA^99^ discriminant model to classify cells in a given subclone 1 and subclone 2 based on single-cell count tables of gene body NO as feature sets. This model provides a variable importance of projection (VIP) and significance compared with a null distribution in the form of a *P* value for each gene analyzed. Similar to the default setting, genes with altered activity were identified using a 10% FDR cutoff when using PLS-DA for inferring changes in gene activity between subclones. Benchmarking both modes (see Extended Data Fig. 4) suggested that, whereas both DESeq2 and PLS-DA offer acceptable performance, the alternative mode (PLS-DA) outperforms the default setting when the subclonal CF is below 10%, whereas the default mode (DESeq2) generated superior results for CF values of 10% or greater.

Genes with altered somatic copy number were masked (removed) when investigating gene activity changes based on NO at gene bodies, since differences in copy number status could confound differential NO measurements.

##### Molecular phenotype analysis in gene sets

This module of scNOVA uses defined gene sets, obtained from public resources, to identify over-represented sets of functionally related genes changing in activity between subclones (or individual cells). Two types of analyses are enabled by this module: (1) gene set over-representation analysis, which can be used to investigate, for example, the enrichment of targets of an important TF among genes showing a change in activity according to gene body analysis of NO; (2) joint modeling of NO across predefined gene sets, using pathway definitions from MSigDB^64^. Throughout the manuscript, we applied an FDR of 10% (*P*_adj._ < 0.1) as a significance threshold.

In the case of gene set over-representation analysis, we collected TF target genes from database entries (EnrichR^50^) as well as by reviewing the literature. When reviewing the literature, we created curated lists of target genes for TFs based on published genome-wide studies using the following strict criteria: (1) target genes show evidence of binding of the TF of interest by chromatin immunoprecipitation followed by sequencing (ChIP–seq); (2) the same genes must additionally show differential expression when the TF of interest is experimentally silenced (our curated target gene lists are available in Supplementary Table 7). For each TF, the significance of overlap between its target gene set and genes exhibiting differential NO was computed using hypergeometric tests, followed by controlling the FDR at 10%.

To jointly model differential NO across all genes of predefined pathways, scNOVA first generates a single-cell gene body NO table using Strand-seq read count data, with these read counts then being normalized using the median-of-ratios method from DESeq2 (ref. ^103^). For each member in the biological pathway gene sets from MSigDB^64^, scNOVA then computes mean normalized NO values, in each single-cell, as a proxy for pathway-level NO. Lowly variable genes (s.d. <80%) are removed. Pathway-level NO is compared between cells with and without SVs using linear mixed model fitting followed by likelihood ratio testing, and controlling the FDR at 10%. For linear mixed model fitting, SV status is defined as a fixed effect and different Strand-seq library batches are defined as random effects, by scNOVA.

### Quantitative real-time PCR

NA20509 was ordered from Coriell and taken into culture at passage four. The late passage was grown until passage eight in a time span of 8 weeks. HG01505 was taken into culture at passage five and was grown until passage nine within a total time span of 6 weeks. DNA, RNA and protein were isolated with the NucleoSpin TriPrep Mini kit (740966.50) according to the manufacturer’s protocol. qPCR was performed on genomic DNA. PCR primers for *MAP2K3* and *TP53* were obtained from Sigma. qPCR was performed using BD SYBR Green PCR Master Mix (4309155) with a final primer concentration of 300 nM each and 10 ng input gDNA. A *GAPDH* control region was used as a normalizer. The primer sequences for DNA qPCR are provided in Supplementary Table 17.

### Drug treatment with CB-103

Primary human T-ALL cells were recovered from cryopreserved bone marrow aspirates of patients enrolled in the ALL-BFM 2009 study. Patient-derived xenografts were generated as previously described by intrafemoral injection of 1 million viable primary ALL cells in NSG mice^104^ Patient-derived xenografts (T-ALL_P1)^24^ cells were frozen until processing. Human hTERT immortalized primary bone marrow mesenchymal stroma cells (MSC; provided by D. Campana) were cultured in Roswell Park Memorial Institute (RPMI) 1640 medium supplemented with 10% heat-inactivated fetal bovine serum, l-glutamine (2 mM), penicillin/streptomycin (100 IU ml^–1^) and hydrocortisone (1 μM). MSCs were seeded in 24-well plates at a concentration of 500,000 cells per well in 1 ml Aim V medium. After 24 h, T-ALL cells were added at a concentration of 1.5 million cells per well in 1 ml Aim V. CB-103 (MedChemExpress, HY-135145) or DMSO (vehicle) as control was added after an additional 24 h at a concentration of 10 μM. After 8 h and 24 h, cells were trypsinized, collected and frozen in 90% fetal bovine serum /10% DMSO.

### Single-cell RNA sequencing and data processing

For scRNA-seq library preparation, cryopreserved cells were thawed rapidly at 37 °C and resuspended in 10 ml warm RPMI medium with 100 μg ml^–1^ Dnase I. Cells were centrifuged for 5 mins at 300*g*, and resuspended in ice-cold PBS with 2% fetal bovine serum and 5 mM EDTA. Cells were stained on ice with anti-murine-CD45-PE (mCD45)(clone 30-F11; BioLegend; 1:20) in the dark for 30 mins. 1:100 4,6-diamidino-2-phenylindole (DAPI) was added and incubated in the dark for 5 mins before sorting. Triple negative cells (4,6-diamidino-2-phenylindole-mCD45-GFP^–^) were sorted (Supplementary Fig. 32) using a BD FACSAria fusion cell sorter into ice-cold 0.03% bovine serum albumin (BSA) in PBS. All isolated cells were used immediately for scRNA-seq libraries, which were generated as per the standard 10x Genomics Chromium 3′ (v.3.1 Chemistry) protocol. Completed libraries were sequenced on a NextSeq5000 sequencer (HIGH-mode, 75 bp paired-end).

Sequenced transcripts were aligned to both human and mouse genomes (GRCh38 and mm10) and quantified into count matrices using cell ranger mkfastq and count workflows (10X Genomics, v.3.1.0, default parameters). The R package Seurat^80^ (v.4.0.3) was used for quality control of single cells and unsupervised clustering of the data. Briefly, human cells were separated from multiplets/mouse contamination based on greater than 97 % of their reads aligning to GRCh38. Further filtering for high quality cells accepted only those with more than 200 but less than 20,000 total RNA counts, and a percentage of mitochondrial reads less than 10% for the untreated data, and less than 40% for the drug-treated samples. Finally, remaining mouse transcripts were removed before further analysis.

In the untreated data, normalization, scaling and regression of mitochondrial read percentage was carried out using the scTransform package^105^. Dimensionality reduction and differential expression analysis of identified clusters was performed as standard using Seurat. Trajectory analysis was performed using Monocle3 (ref. ^106^). In the drug treatment data, individual Seurat objects that had been quality controlled as above were normalized by scTransform^105,107^ and then integrated to correct for batch effects and allow for comparative analysis. To re-annotate clusters from the untreated data in the drug treatment data, the TransferData() function from Seurat^80^ was used to project labels from our reference (that is, untreated data) onto the integrated drug treatment data. Single-cell gene set enrichment analysis was performed using the R package ‘escape’^67^.

### Cellular indexing of transcriptomes and epitopes by single-cell sequencing

A peripheral blood-derived sample (CLL_24) was recovered from cryopreservation as previously described^108^ to reach viability above 90%. Then, 5 × 10^5^ viable cells were stained by a premixed cocktail of oligonucleotide-conjugated antibodies (Supplementary Table 14) and incubated at 4 °C for 30 min. We provided dilution used for each antibody in Supplementary Table 14. Cells were washed three times with ice-cold washing buffer. After completion, bead-cell suspensions, synthesis of complementary DNA and single-cell gene expression and antibody-derived tag (ADT) libraries were performed using a Chromium single cell v.3.1 3ʹ kit (10× Genomics) according to the manufacturer’s instructions. Then, 3′ gene expression and ADT libraries were pooled in a ratio of 3:1 aiming for 40,000 reads (gene expression) and 15,000 reads per cell (ADT), respectively. Sequencing was performed on a NextSeq 500 (Illumina). After sequencing, the cell ranger wrapper function (10x Genomics, v.6.1.1) cellranger mkfastq was used to demultiplex and to align raw base-call files to the human reference genome (hg38). The obtained FASTQ files were counted by the cellranger count command. If not otherwise indicated default settings were used. Single-cell gene set enrichment analysis was performed using the R package ‘escape’^67^.

### Single-cell gene signature scoring using UCell

The activity of the scNOVA-identified gene set from T-ALL_P1 in scRNA-seq data was profiled using the UCell package^81^. Briefly, signature genes considered were those with either increased (implying decreased expression) or decreased (implying increased expression) nucleosome occupancy (see Fig. 5b), or genes encoding TFs whose targets showed differential nucleosome occupancy (see Fig. 5c). The following gene set was used for T-ALL_P1: ‘PRKCB–’, ‘RPS6KA2–’, ‘FAM120B–’, ‘FAM86C1+’, ‘FBXO22+’, ‘RHOH+’, ‘SLC9A7+’, ‘NASP+’, ‘NOTCH1+’, ‘MRPL48+’, ‘MFSD9+’, ‘MVB12B+’, ‘MYB+’ (with ‘+’ for upregulated, and ‘–’ for downregulated). The score per single cell for the entire directional gene set was calculated using the AddModuleScore_UCell() function. Cells were considered to be ‘active’ for the signature genes if their respective UCell score was greater than or equal to the median UCell score of the entire dataset, plus the s.d. Similarly, for T-cell cell-type labeling, marker gene sets for T-cell subsets were obtained from Park et al.^109^ and single cells were scored for their activity in each gene set. Cells were labeled by their best-fit cell type, that is the cell-type whose gene set gave the highest UCell score.

### Reporting summary

Further information on research design is available in the Nature Research Reporting Summary linked to this article.

## Online content

Any methods, additional references, Nature Research reporting summaries, source data, extended data, supplementary information, acknowledgements, peer review information; details of author contributions and competing interests; and statements of data and code availability are available at 10.1038/s41587-022-01551-4.

### Supplementary information

Supplementary Information Supplementary Figs. 1–41, descriptions for Tables 1–17, notes for methodological details and Discussion.

Reporting Summary

Supplementary Table Supplementary Tables 1–17.

Supplementary Data Snapshots of somatic SV events in LCLs.

## Data Availability

Sequencing data from this study can be retrieved from the European Genome-phenome Archive (EGA) and the European Nucleotide Archive (ENA). LCL data are available under the following accessions: Strand-seq (PRJEB39750, PRJEB55038); RNA-seq (ERP123231); WGS (PRJEB37677). C11 cell line data are available under the accession PRJEB55012. Leukemia patient data and human primary cells derived data were deposited in the European Genome-phenome Archive (EGA) under the following accession numbers: skin fibroblast (EGAS00001006498); cord blood (EGAS00001006567). T-ALL Strand-seq and scRNA-seq (EGAS00001003365), CLL Strand-seq (EGAS00001004925), AML Strand-seq (EGAS00001004903), T-ALL bulk RNA-seq (EGAS00001003248), CLL bulk RNA-seq (EGAS00001005746), CLL CITE-seq (EGAS00001004925). Access to human patient data is governed by the EGA Data Access Committee.

## Code availability

The computational code of our analytical framework scNOVA is available open source at https://github.com/jeongdo801/scNOVA, with no restrictions on reuse. Other software used: Mosaicatcher (https://github.com/friendsofstrandseq/mosaicatcher-pipeline), StrandPhaseR (https://github.com/daewoooo/StrandPhaseR), InferCNV (https://github.com/broadinstitute/inferCNV/), HoneyBADGER (https://jef.works/HoneyBADGER/), CONICSmat (https://github.com/diazlab/CONICS), NucTools (https://homeveg.github.io/nuctools), Delly2 (https://github.com/dellytools/delly), BWA (v.0.7.15), STAR (v.2.7.9a), SAMtools (v.1.3.1), biobambam2 (v.2.0.76), deeptools (v.2.5.1), perl (v.5.16.3), Python (v.3.7.4), cuDNN (v.7.6.4.38), CUDA (v.10.1.243), TensorFlow (v.1.15.0), scikit-learn (v.0.21.3), matplotlib (v.3.1.1), R v.4.0.0, DESeq2, FlowJo and BD FACSDiva.
