# Supplementary material for: Functional analysis of structural variants in single cells using Strand-seq
Source: Nat Biotechnol. 2022 Nov 24;41(6):832–44. doi: 10.1038/s41587-022-01551-4 (PMC10264249; doi:10.1038/s41587-022-01551-4)
Supplement: Supplementary file 1 — Supplementary Figs. 1–41, descriptions for Tables 1–17, notes for methodological details and Discussion. [file 41587_2022_1551_MOESM1_ESM.pdf]

nature biotechnology

Article https://doi.org/10.1038/s41587-022-01551-4

Functional analysis of structural variants in single cells using Strand-seq

In the format provided by the authors and unedited

Supplementary Materials

Supplementary Materials to: “Functional analysis of structural variants in single cells using Strand-seq”

Index: This Supplement Information is divided into Figures, Tables, Data, Supplementary Notes for Methodological Details, Supplementary Discussion and References.

Supplementary Figures

Supplementary Tables

Supplementary Data

Supplementary Notes for Methodological Details

1. Estimating genome-wide coverage

2. Analysis and comparison of NO profiles derived from Strand-seq and MNase-seq

3. Visualization of NO at gene bodies for genes stratified by their expression level

4. Analysis of previously reported scMNase-seq data

5. Cell type classification

6. Haplotype-resolved SV discovery in single cells

7. CNN model to infer expressed genes based on NO

8. Identifying optimal parameters for inferring changes in gene activity using NO

9. NO-based inference of effects of multiple SVs using scNOVA

10. Molecular phenotype analysis in gene sets in cell lines and leukemia samples

11. Additional details with respect to the haplotype-specific NO analysis

12. Analysis of local effect of SVs in an AML patient

13. Analysis of local effect of SVs in T-ALL_P1

14. Analysis of local effect of SV in BM510

15. Bulk-cell RNA-seq data processing and allele-specific expression analysis

16. Bulk RNA-seq analysis in thirteen T-ALL patient-derived samples

17. Bulk RNA-seq analysis in 42 CLLs

18. Haplotype-resolved bulk RNA-seq analysis in LCLs from HGSVC consortium

19. Clinical diagnostic information for CLL_24

20. Clinical diagnostic information for AML_1

21. 10q deletion discovery in CLL samples from PCAWG

22. Strand-seq in a panel of lymphoblastoid cell lines (LCLs)

23. WGS-based subclonal SV analysis in NA20509

24. Manual curation of somatic SVs in LCLs to achieve a high-quality callset

25. scRNA-seq data analysis for inferring somatic copy number alterations (SCNAs)

26. Pseudotime/cell-type analysis of scRNA-seq data

27. Generation of composite track for AML_1 translocation

28. Overlap analysis of high-resolution SV calls and haplotype-specific NO in NA12878

29. Comprehensive summary of CITE-seq analysis

30. Minimum gene length required to detect haplotype-specific NO at gene body

Supplementary Discussion

I. Scope of scNOVA compared to single-cell multiomics methods focusing on SCNAs

II. Known and suspected Wnt signaling regulators near 10q24.32

III. Nucleosome repeat length measurements: considerations for future users

IV. Further details of functional outcomes of somatic rearrangement landscapes in lymphoblastoid cell lines

V. Comparison of haplotype-specific CRE activity in the chromosome X of female genome inferred by scNOVA, and reported by previous studies

VI. Germline copy number status of *MAP2K3* locus in NA20509

Supplementary Figures

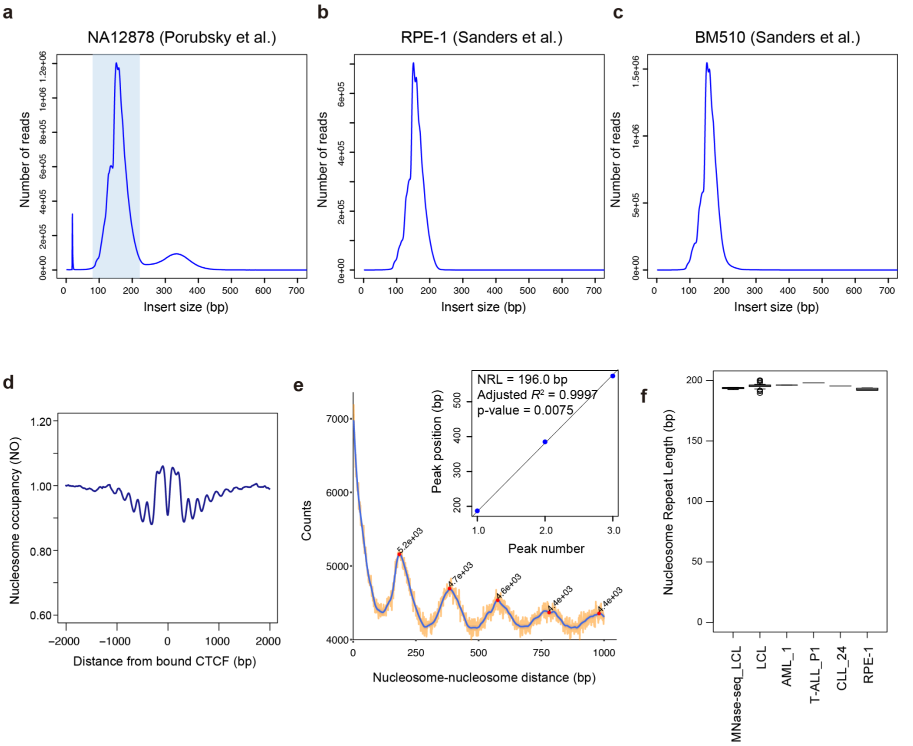

Figure S1. Strand-seq reflects the characteristics of MNase-seq profiles. (a-c) Fragment size distribution calculated from the distance between paired-end read alignment positions represent nucleosomal fragments in Strand-seq data from multiple independent experiments. **(a)** Strand-seq libraries from NA12878[2] show a bimodal read length (insert size) distribution implying existence of both mono-nucleosomal and di-nucleosomal sized fragments (see **Supplementary Discussion III**). NA12878 (also known as GM12878, or CEPH1463) represents a widely used human reference and benchmarking sample. **(b)** By comparison, Strand-seq libraries for RPE-1 (the originally commercially available cell line) and **(c)** the RPE-1 derived BM510 cell line[3] were generated using stringent size selection[1] at 250-350bp (a size representing mononucleosome fragments that still include the sequencing adapters). These data are unimodal reflecting the specific enrichment of mono-nucleosomal sized fragments. Strand-seq libraries newly generated in this study (**Table S1**) follow the strict size selection in [1], and select the 250-350bp (unimodal) sized fragments. For all down-stream analyses we performed an *in-silico* size selection of NA12878 for fragments between 80-220 base pairs in size (after trimming adapter sequences) to enrich for mono-nucleosomal sized fragments (indicated by the first peak in blue background), to be consistent with other Strand-seq libraries investigated in this study. **(d)** Genome-wide averaged nucleosome patterns at CTCF binding sites, based on pooled Strand-seq libraries generated for NA12878. CTCF binding sites for NA12878 were obtained from ENCODE[4]. **(e)** Representative histogram of distances between nucleosomes calculated from pooled Strand-seq HG00096 (N=69 single cell libraries) using NucTools[5]. Plot for chromosome 19 shown as an example. Peak positions represent the distances between the nearest-neighbor[6], followed by the 2nd, 3rd, etc. Inlet figure shows the scatter plot of peak position versus the peak number considering 1st~3rd neighboring nucleosomes, using the peaks in the histogram in (d). Nucleosome repeat length[6] was estimated based on slope values derived using linear regression as shown in the inlet scatter plot (p-value was calculated based on Spearman correlation test). **(f)** Nucleosome repeat length[6] estimates for pooled Strand-seq libraries shown for different cell types (n = 28, 1, 1, 1, 4 biologically independent samples for LCL, AML (AML_1), T-ALL (T-ALL_P1), CLL (CLL_24), and RPE-1, respectively). Publicly available bulk-cell MNase-seq data from LCLs[7] are shown for comparison. MNase-seq data were downsampled to 70 million fragments per experiment to make them as comparable as possible to the pooled Strand-seq data. Nucleosome repeat length[6] estimates were consistent between independent Strand-seq experiments (195.4 ± 0.4bp) and concordant with bulk MNase-seq (193.7 ± 0.6bp), showing these patterns are reproducible.

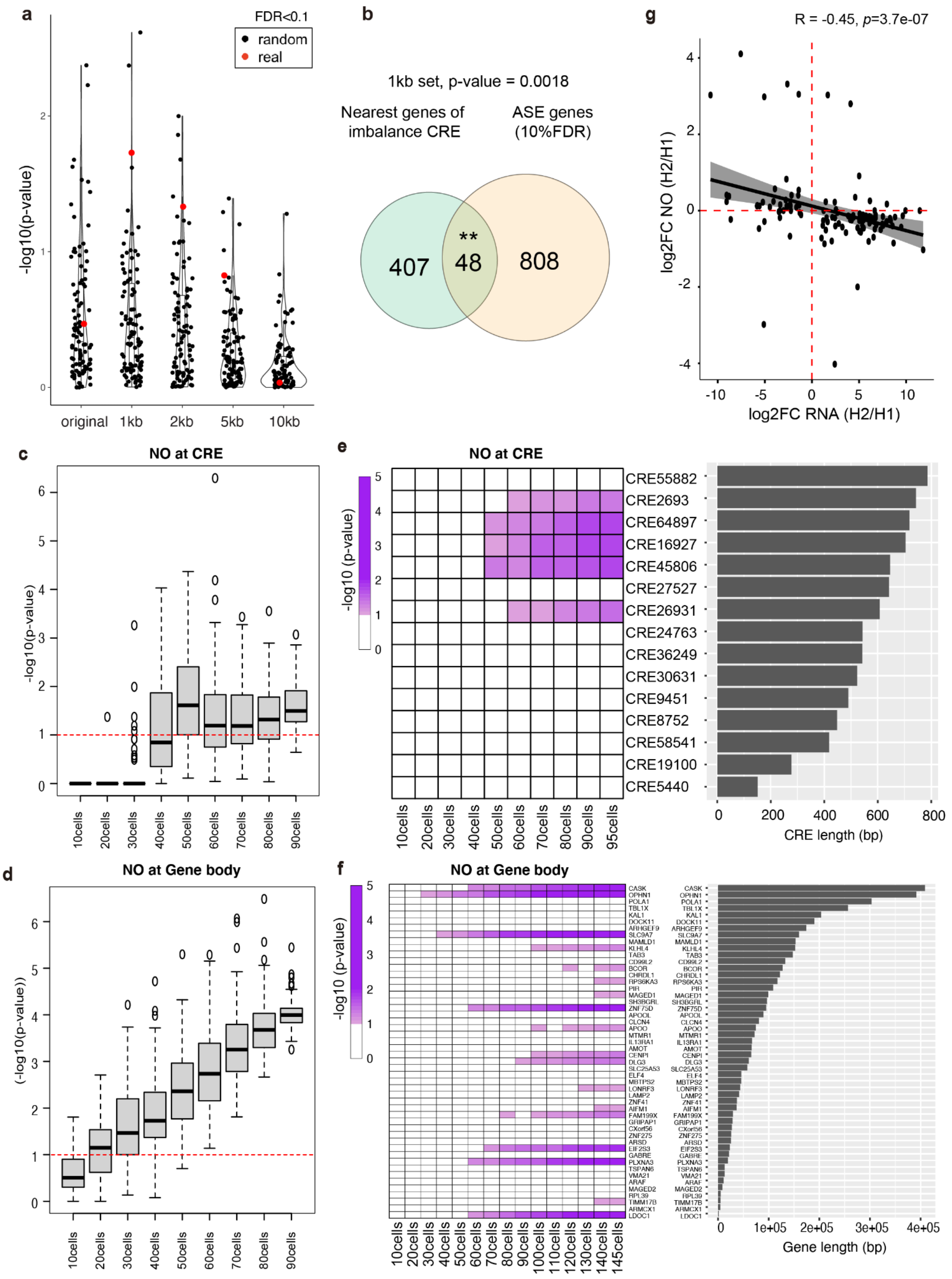

Figure S2. Haplotype-phased NO tracks reveal haplotype-specific NO. We extracted the genomic positions of 66,254 active CREs previously defined in NA12878 using bulk ATAC-STARR-seq[9], to parameterize the identification of haplotype-specific NO at CREs (*i.e.* scNOVA's 'Infer haplotype-specific NO' module). Because the average annotated CRE length was only 350bp ('original'), which is much smaller than average gene body length (67,104 bp), we expanded the search space around each CRE to examine whether this improved the ability of scNOVA to discern CREs displaying haplotype-specific NO in sparse single-cell data. We tested five sets of data, by extending CREs to 1kb, 2kb, 5kb, and 10kb intervals, each centered at the original CRE midpoint, and comparing these four sets to the originally defined[9] set of CREs. For each set, we measured haplotype-specific NO in the pseudo-bulk NO tracks generated from NA12878 Strand-seq data, using a 10% FDR cutoff. **(a)** Violin plot representing the enrichment score (-log10 p-value from hypergeometric test) of CREs with haplotype-specific NO on the inactive chromosome X. To confirm the enrichment signal is driven by the regulatory elements, we

performed a randomization test by shifting the previously defined CRE locations[9] (original length preserved) +- 50kb and repeating the haplotype-specific NO test for each CRE set (i.e. 'original', 1kb, 2kb, 5kb, and 10kb). Given the well-known X-inactivation process, we expect haplotype-specific NO at CREs to be enriched on the X chromosome. The chromosome X enrichment scores of 100 randomization trials are depicted as black dots in each violin plot (n = 100 independent trials; Together with violin plot, boxplots were defined by minima = 25th percentile - 1.5X interquartile range (IQR), maxima = 75th percentile + 1.5X IQR, center = median, and bounds of box = 25th and 75th percentile.), and the score calculated for the original (unshifted) CRE is depicted as a red dot. This randomization test shows that haplotype-specific NO at CREs is best resolved when using CREs of a 1kb length (achieved by accordingly extending previously defined[9] CRE locations). **(b)** Venn diagram showing significant overlap between target genes of CREs displaying haplotype-specific NO and genes showing allele-specific expression (ASE) in NA12878. Putative target genes for each CREs were assigned based on the nearest gene approach[10]. ASE genes were defined by phasing bulk RNA-seq data generated for NA12878 using Strand-seq based haplotype information, followed by examination of haplotype-specific read counts with EdgeR[11], using a 10% FDR. The enrichment *P*-value depicted was estimated using a one-sided hypergeometric test ($P<0.0018$). This is close to the 2-fold enrichment of allele-specific transcription factor (TF) binding previously linked with X inactivation[12]. Genes targeted by CREs with haplotype-specific NO show a significant proclivity to be allele-specifically expressed. (**c**) Downsampling analysis of cell number to detect haplotype specific NO for CREs on chromosome X. Among the 95 good quality cells of NA12878, we downsampled 90 cells to 10 cells, using a 10 cell interval, and randomly picking 100 sets of cells at each step. For each downsampling trial, we performed a hypergeometric test to evaluate enrichment of haplotype-specific NO in one haplotype of chromosome X. Y-axis denotes the enrichment score (-log10 p-value) from the test (one-sided hypergeometric test; n = 100 independent trials; Boxplots were defined by minima = 25th percentile - 1.5X interquartile range (IQR), maxima = 75th percentile + 1.5X IQR, center = median, and bounds of box = 25th and 75th percentile.). (**d**) Downsampling analysis for gene body NO on chromosome X. For each downsampling trial, we performed a two-sided Wilcoxon ranksum test to evaluate haplotype-specific NO. Y-axis denotes the enrichment score (-log10 p-value) from the test (n = 100 independent trials; Boxplots were defined by minima = 25th percentile - 1.5X interquartile range (IQR), maxima = 75th percentile + 1.5X IQR, center = median, and bounds of box = 25th and 75th percentile.). These down-sampling analysis showed that 40-50 cells were required in the pseudo-bulk pool to reliably identify the inactive X based on CREs, whereas only 10-20 cells were needed when using gene bodies instead (10% FDR). (**e**) Haplotype-specific NO at CREs previously analyzed for allele-specific activity. Heatmap depicting haplotype-specific NO measurements at CREs during downsampling analysis. We downsampled the cell number as described in (**c**), and for each trial inferred haplotype-specific NO using scNOVA for 15 CREs with previously reported haplotype specific activity[9]. Bar graph in the right panel shows the length of 15 CREs tested in this analysis. (**f**) Heatmap showing the haplotype-specific NO at gene bodies measured by downsampling analysis of cell number using BM510. We downsampled the cell number as described in (e), and for each trial we inferred haplotype-specific NO using scNOVA for chromosome X genes showing allele-specific expression (ASE) from the bulk RNA-seq of this cell line. Bar graph in the right panel shows the length of ASE genes tested in this analysis. (**g**) Scatter plot showing the negative correlation between log fold changes of haplotype-specific RNA expression (x-axis), and haplotype-specific NO (y-axis). Those two factors show significant negative correlation (spearman correlation coefficient = -0.45, p-value = 3.7e-07).

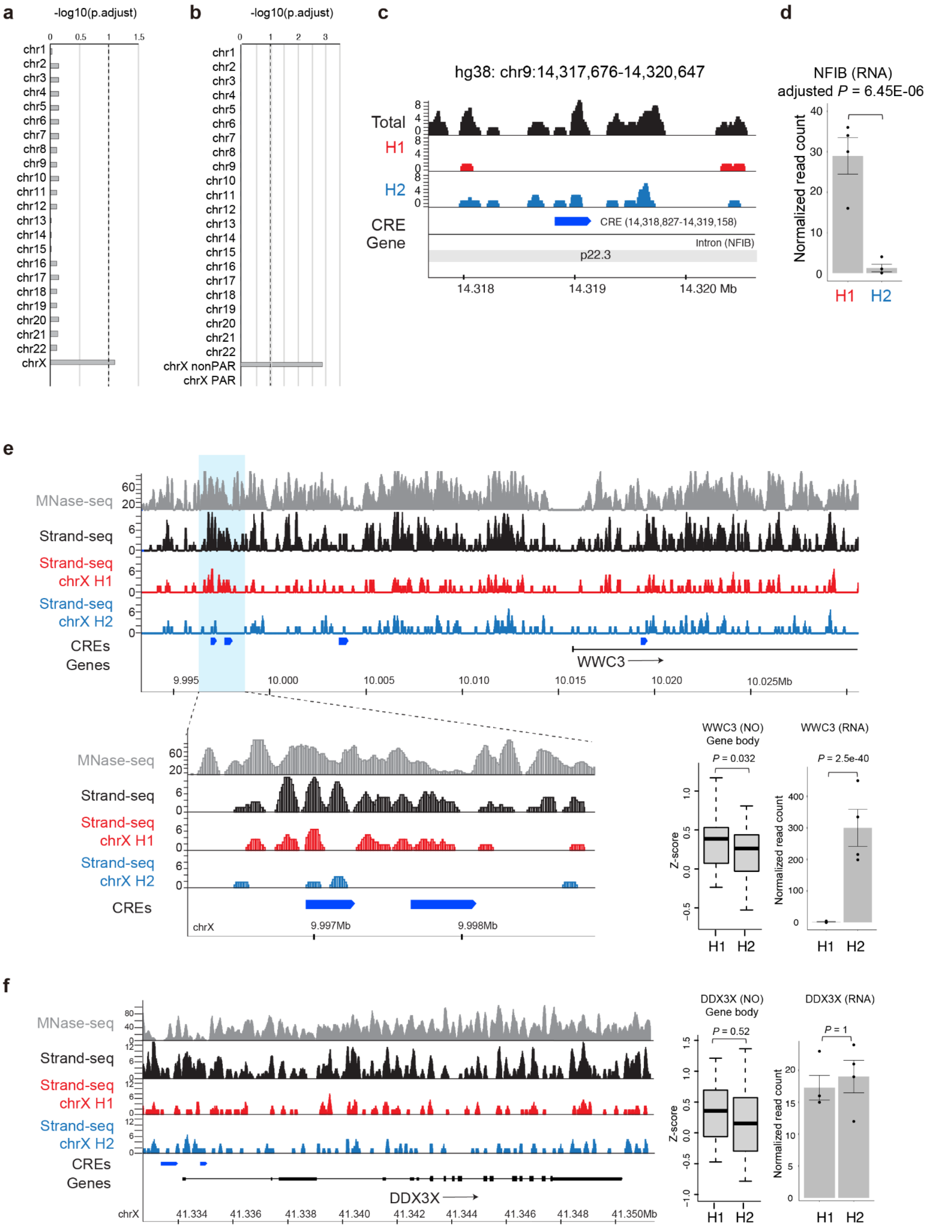

Figure S3. NA12878 haplotype-phased NO tracks, computed for CREs and gene bodies at the level of full-length chromosomes, are consistent with patterns of X-inactivation. (a) Single-cell level average NO signals for CREs displayed increased NO on the inactive X chromosome, indicating reduced CRE accessibility on this homologue (significant with 10% FDR; adjusted *P*=0.081, two-sided Wilcoxon signed-rank test). **(b)** Single-cell level average NO signals at gene bodies for expressed genes (FPKM>1 in bulk-cell RNA-seq data[4]) were computed per haplotype, which revealed increased NO on the inactive X chromosome (adjusted *P*=0.0012, two-sided wilcoxon signed-rank test, followed by controlling[13] the FDR). Pseudoautosomal regions (PAR) were tested separately from the remainder of chromosome X ("nonPAR"). (**c**) Pseudo-bulk haplotype-phased NO tracks based on Strand-seq, depicting a previously defined CRE[9] in NA12878 with haplotype-specific absence of NO on H1 (10% FDR). Total: aggregated phased and unphased Strand-seq reads. (**d**) Bar chart shows the allele-specific expression on H1 of *NFIB* – the inferred target gene of this CRE (derived from n = 4 biological replicates; Data are presented as mean values +/- SEM). (**e-f**) NO tracks of NA12878 based on bulk-cell MNase-seq and pooled (pseudo-bulk) Strand-seq data. Representative loci (**e**) undergoing X-inactivation (*WWC3*) and (**f**) escaping from X-inactivation (*DDX3X*) are shown. CRE definitions are based on[9]. Adjacent boxplot represent measurements of haplotype imbalances in NO at the respective gene bodies (nominal *P*<0.032 for *WWC3*, and *P*<0.52 for *DDX3X*; two-sided likelihood ratio test; Boxplots were defined by minima = 25th percentile - 1.5X interquartile range (IQR), maxima = 75th percentile + 1.5X IQR, center = median, and bounds of box = 25th and 75th percentile.).

Bar graphs depict haplotype-resolved bulk RNA-seq[4] read counts (FDR-adjusted $P<2.5E-40$ for *WWC3*, and $P<1$ for *DDX3X*; two-sided likelihood ratio test; derived from n = 4 biological replicates; Data are presented as mean values +/- SEM).

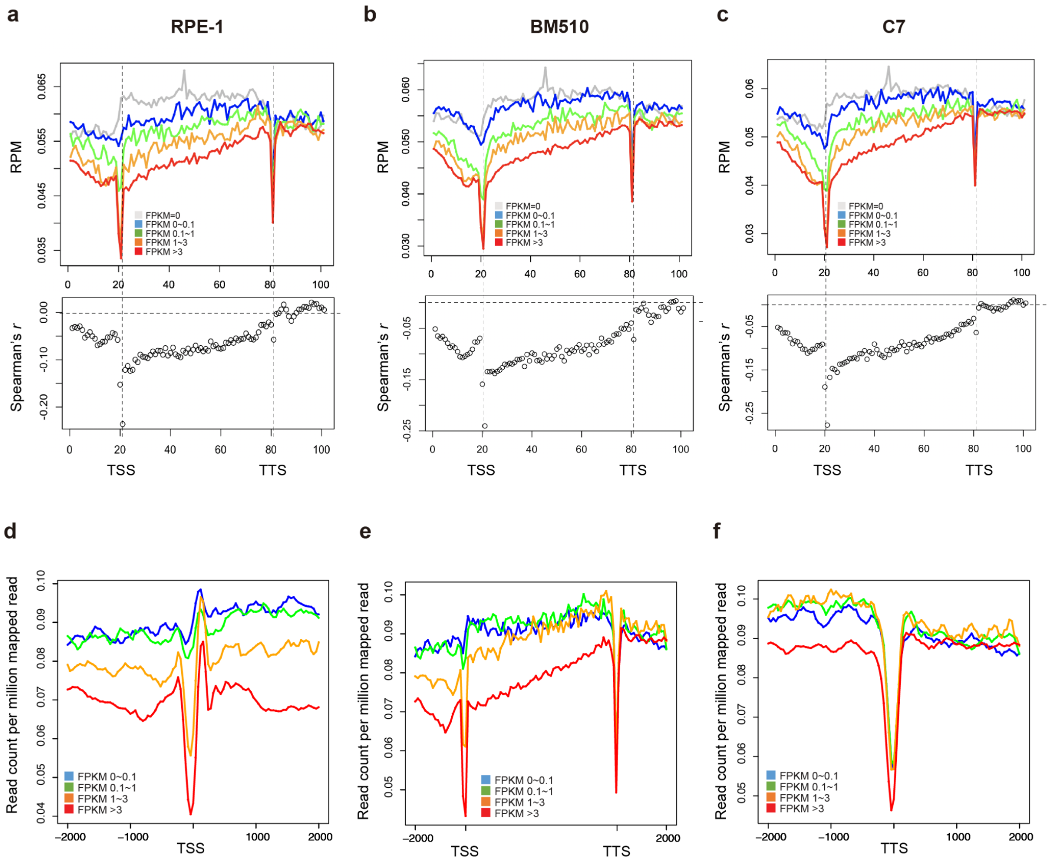

Figure S4. Inverse correlation between NO at the body of genes and bulk RNA-seq gene expression values. Inverse correlation shown for three RPE-1 derived cell lines[1]: the original RPE-1 cell line **(a)**, BM510 **(b)**, and C7 **(c)** NO was calculated for 101 bins spanning -2kb to +2kb of gene bodies as a read count per million mapped reads using ngsplot software[14]. For each of the bins, genome-wide correlation between NO and gene expression level from bulk RNA-seq data was measured using Spearman's rho. Inverse correlation between NO and gene expression was apparent along the entire gene body (see gray dots), with the most pronounced inverse correlation measured at the TSS. **(d-f)** An equivalent inverse correlation between NO and gene expression level was also seen in published scMNase-seq data from a mouse cell line[15] (NIH3T3) - consistent with (pooled) Strand-seq and (pooled) scMNase-seq based read tracks being highly concordant along the genome (see main text and **Figure 1**). Binned NO profiles of the **(d)** TSS (±2kb), **(e)** gene bodies (±2kb), and **(f)** the transcriptional termination site (TTS; ±2kb) were extracted from pooled scMNase-seq data[15] using 45 single cells in total. We downloaded raw fastq files (GSE96688) and aligned these data to the mouse mm10 reference genome. The mono-nucleosomal fraction was extracted (140-180bp) (**Supplementary Notes**), and NO for genomic bins around TSSs (±2kb), gene bodies (±2kb), and TTSs (±2kb) computed using ngsplot[14]. Genes were divided into four groups based on expression values (FPKM) measured by bulk RNA-seq of NIH3T3 cells[16] (depicted in red, orange, green and blue).

a

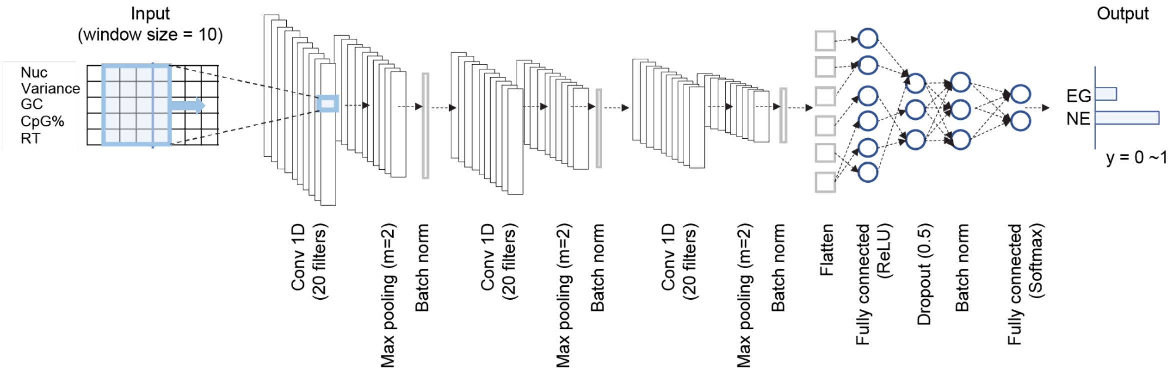

b

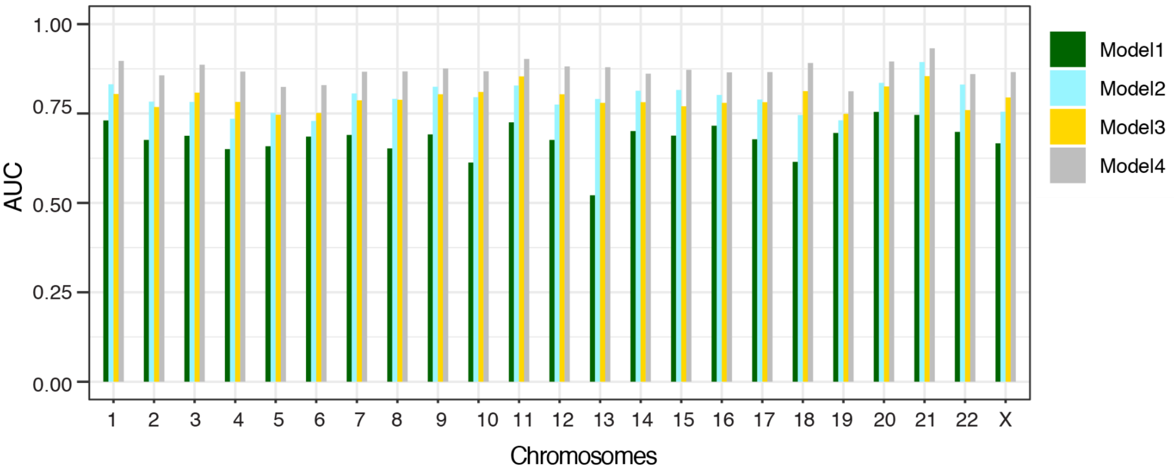

Figure S5. Parameterization of convolutional neural network (CNN) in scNOVA. (a) CNN architecture used by scNOVA. The 'Input' table shows a schematic representation of five layers of feature sets built into scNOVA's CNN, which include NO, single-cell variance of NO, GC%, CpG%, and replication timing (RT); the latter three features have been reported to be associated with nucleosome positioning patterns[15,19–21], and have therefore been included in the CNNs to assist bin stratification. **(b)** Performance evaluation by cross validation, using different features and setups ("Models"), here used for choosing the optimal CNN model. Model 1 uses two features, 2K-TSS (-1kb to +1kb around the TSS) and nucleosome depleted region (-400 to +100bp around the TSS)[22]. Model 2-4 considers the region -5kb to +5kb of gene bodies divided by 150 bins. Model 2 uses two layers of Strand-seq features (occupancy + variation). Model 3 uses three layers of genome annotation features only (CpG, GC, and RT). Model 4 uses five layers of features including Strand-seq features (occupancy + variation) and genome annotation features (CpG, GC, and RT). All models were trained by CNN except for Model 1, which was trained using a support vector machine (SVM) based setup[22]. The average AUC values of Model 1, 2, 3, and 4 were 0.679, 0.793, 0.791, and 0.871, respectively, and we thus chose Model 4 (occupancy, variation, CpG, GC, and RT) when parameterizing scNOVA. As an output, for each gene, this model provides the probability for a gene to be expressed (EG, expressed gene) or not expressed genes (NE, non-expressed gene), which when combined with scNOVA's generalized linear models can be used to robustly infer alterations in gene activity (see **Supplementary Methods**).

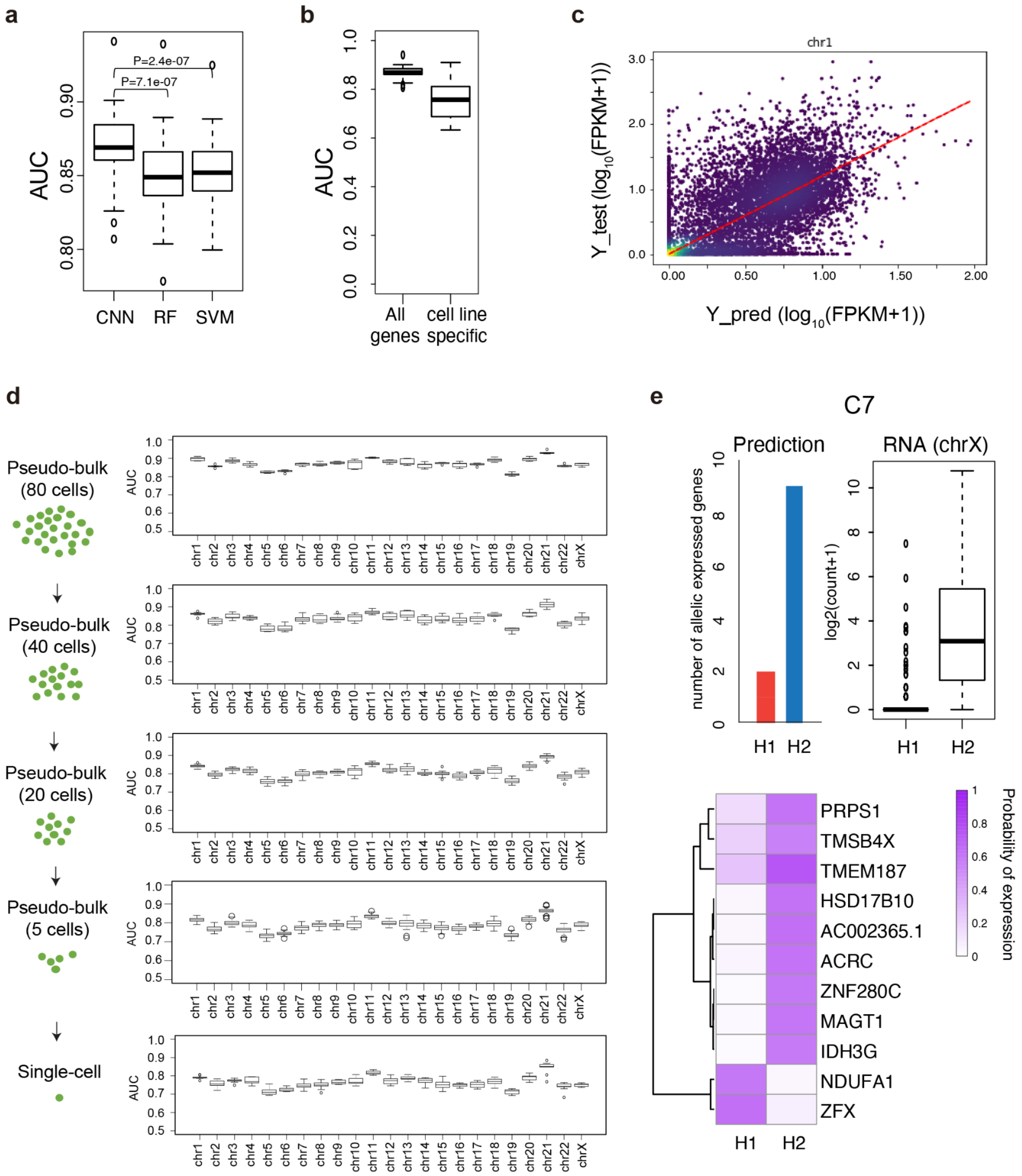

Figure S6. Performance evaluation for scNOVA's CNN and comparison with other machine learning models. (a) Comparison of AUC values based on leave-one-chromosome out cross validations from CNN, random forest (RF), and support vector machine (SVM). In this comparison, AUC was measured for the default (haplotype-unaware) CNN performing binary classification. All three models were trained with the same set of features, using pseudo bulk Strand-seq datasets from RPE-1 (79 cells), BM510 (plate 1: 70 cells; plate 2: 75 cells), and C7 (plate 1: 82 cells; plate 2: 72 cells). Published bulk-cell RNA-seq from these cell lines[1] was used to define ground truth labels for ~10,000 expressed genes per cell line. A boxplot depicts the AUC values from 23 cross validation experiments (one experiment per chromosome). The measured performance of the CNN surpassed random forest (RF) and support vector machine (SVM) based machine learning setups (two-sided Wilcoxon rank sum test followed by Bonferroni correction, P = 2.4e-07 for CNN vs. SVM, P = 7.1e-07 for CNN vs. RF; n = 23 independent trials). **(b)** AUC values for inferring gene expression ON/OFF status for all genes vs. cell type-specific genes (n = 23 independent trials). **(c)** Scatter plot of FPKM values measured by bulk RNA-seq (y axis), and inferred expression values predicted by the 'regression mode' of scNOVA's CNN (x axis). The scatter plot shows the result of leave-chromosome-1-out cross validation (Spearman correlation r=0.72; P<2.2e-16 based on Spearman correlation test). The mean Spearman correlation coefficient across all 23 chromosomes was 0.68 (P<2.2e-16 based on Spearman correlation test). **(d)** AUC values for each chromosome, estimated in downsampled aggregated ('pseudo-bulk') Strand-seq data, as well as in single cells, using scNOVA's CNN. The overall AUC was computed as the weighted average over 23 chromosome pairs, scaled by the number of genes

per chromosome. This downsampling analysis yielded AUC estimates of 0.78-0.87 in pooled, and AUC=0.76 in single cells for inferring expressed genes in RPE-1 cells. We note that coupling of the CNN with scNOVA's generalized linear models (see **Methods**) is highly recommended when using the scNOVA framework. **(e)** When exploring the utility of machine learning to infer gene activity based on NO tracks, we devised both haplotype-aware and -unaware CNNs. This panel depicts results generated by using haplotype-aware binary classification, with a haplotype-aware CNN, to infer the active X chromosome in the (female donor-derived) retinal pigment epithelial cell line C7. In C7, the CNN inferred the H2 haplotype as the active X chromosome. Bulk RNA-seq results[1] verified this inference (derived from n = 2 biological replicates). Boxplots were defined by minima = 25th percentile - 1.5X interquartile range (IQR), maxima = 75th percentile + 1.5X IQR, center = median, and bounds of box = 25th and 75th percentile throughout this figure.

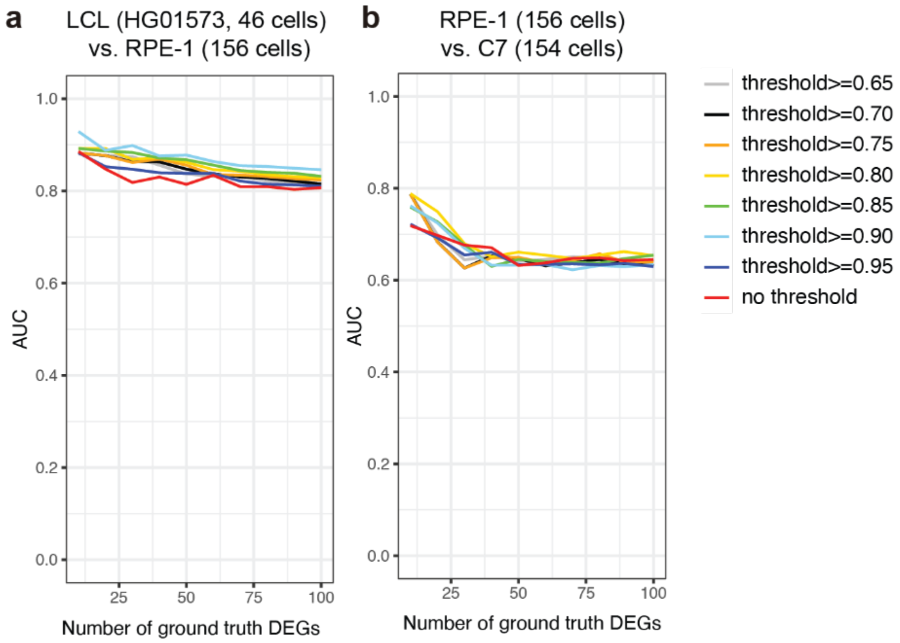

Figure S7. Parameterization of scNOVA's differential gene activity analysis module. (**a**) As explained in the Methods, scNOVA first infers expressed genes (EGs) and non-expressed genes (NEs) using its default CNN, and then removes genes inferred to represent NEs. It then uses negative binomial generalized linear models (as available in the DESeq2 package[23]) on all remaining genes, to investigate NO changes at gene bodies, and accordingly to infer changes in gene activity. AUC, area under the curve. DEGs, differentially expressed genes (DEGs). DEGs (the "ground truth") are based on bulk-cell RNA-seq data subjected to DESeq2, comparing RPE-1 and HG01573. Coloring indicates the threshold used to filter out NEs based on the CNN: *e.g.* the threshold>=0.90 means that genes showing a probability of at least 0.9 to be not expressed (expression status='OFF') were filtered out. We chose 0.9 as the default threshold parameter, the application of which improved performance. (**b**) We extended these benchmarks to pseudo-clones generated from two RPE cell lines, represented by 156 RPE-1 cells (the original hTERT-immortalized cell line) and 154 C7 (which underwent transformation)[3]. For these related RPE cell lines, we measured an AUC of 0.73 for the 10 most differentially expressed genes, and scNOVA inferred 615 genes to increase in activity in C7. In line with its transformed status, several cancer-related genes (e.g. CDK1, *EEF1A2*) were more active in C7, and "carcinoma" was the most enriched functional category (**Table S2**). We concluded that scNOVA enables accurate inference of global gene activity changes based on NO data.

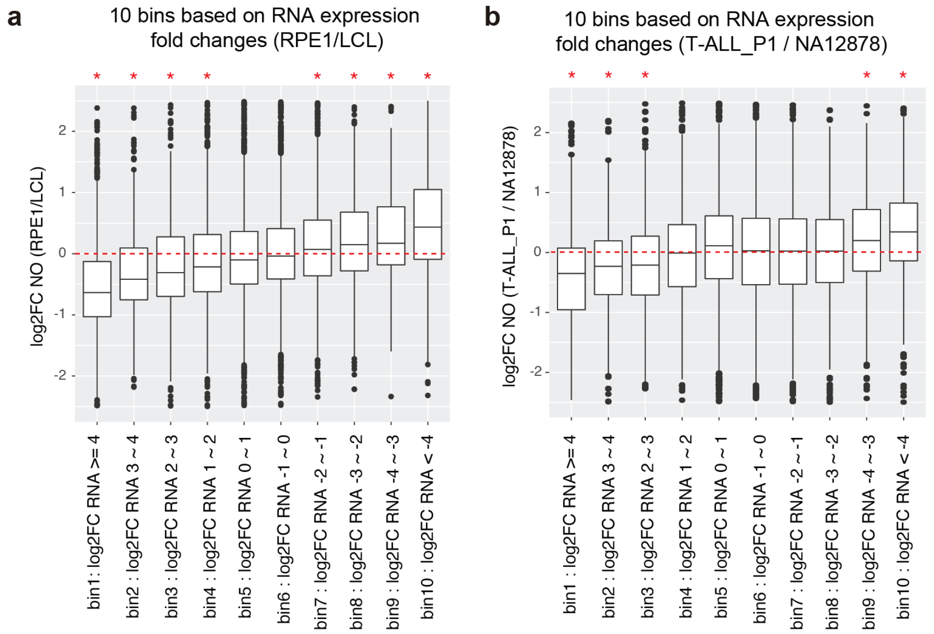

Figure S8. Evaluation of RNA fold changes between two samples that can be detected by scNOVA. (**a**) For the comparison of RPE-1 vs. LCL (HG01573), log2 fold changes of NO at gene bodies were depicted for 10 bins of genes stratified by their RNA expression fold changes (n = 79, and 46 cells for RPE-1 and LCL, respectively). Asterisks indicate that the fold change of NO is significantly different from 0 (adjusted p-value<0.1; one sample t-test followed by multiple correction). Adjusted p-value = 6.76E-88, 1.04E-04, 2.63E-03, 1.66E-02, 1.95E-14, 7.23E-10, 1.90E-06, and 1.84E-34 for bin1, 2, 3, 4, 7, 8, 9, and 10, respectively. (**b**) The same evaluation was repeated for T-ALL_P1 vs. NA12878 (n = 77, and 95 cells for T-ALL_P1 and NA12878, respectively). Adjusted p-value = 2.84E-26, 6.03E-06, 2.17E-06, 1.81E-03, and 1.06E-19 for bin1, 2, 3, 9, and 10, respectively. Boxplots were defined by minima = 25th percentile - 1.5X interquartile range (IQR), maxima = 75th percentile + 1.5X IQR, center = median, and bounds of box = 25th and 75th percentile.

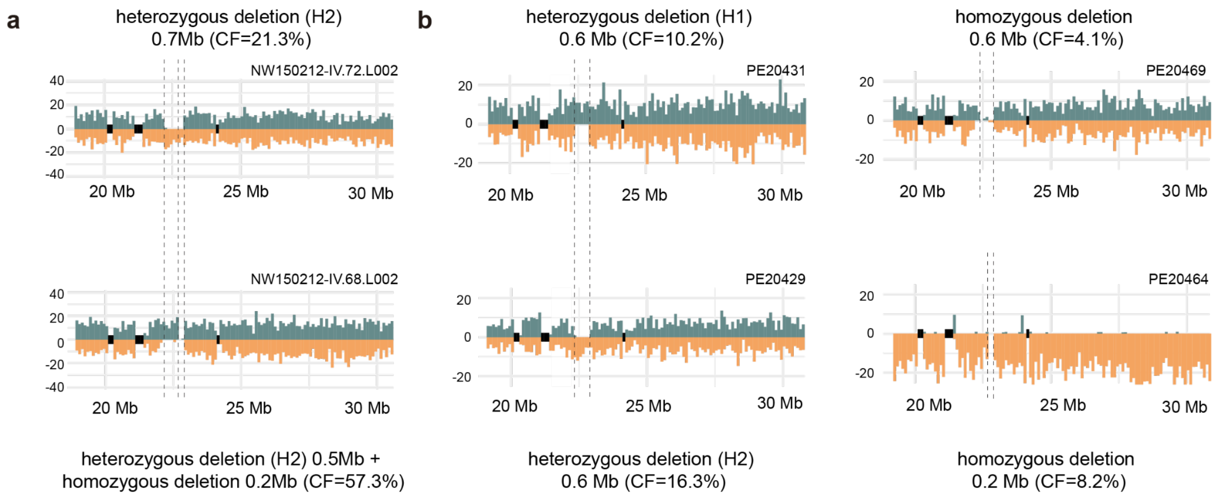

Figure S9. Representative plots of LCLs which show evidence for at least two subclones exhibiting 22q11.2 deletions. (**a**) Two 22q11.2 deletion bearing subclones found in NA12878, one subclone with 700kb heterozygous deletion (chr22:22.2Mb-22.9Mb), and the other subclone with 500kb heterozygous (chr22:22.2Mb-22.7Mb) and 200kb homozygous deletions (chr22:22.7Mb-22.9Mb). NA12878 additionally harbors a subclone bearing a 19q13.12 deletion (see main text). (**b**) Four 22q11.2 deletion bearing subclones detected in HG00171, which indicates that this LCL is a polyclonal cell line. Three subclones show hemizygous and/or homozygous deletion of a 0.6 Mb region (chr22:22.3Mb-22.9Mb), whereas one subclone shows homozygous loss of a 0.2 Mb region (chr22:22.7Mb-22.9Mb), at 22q11.2.

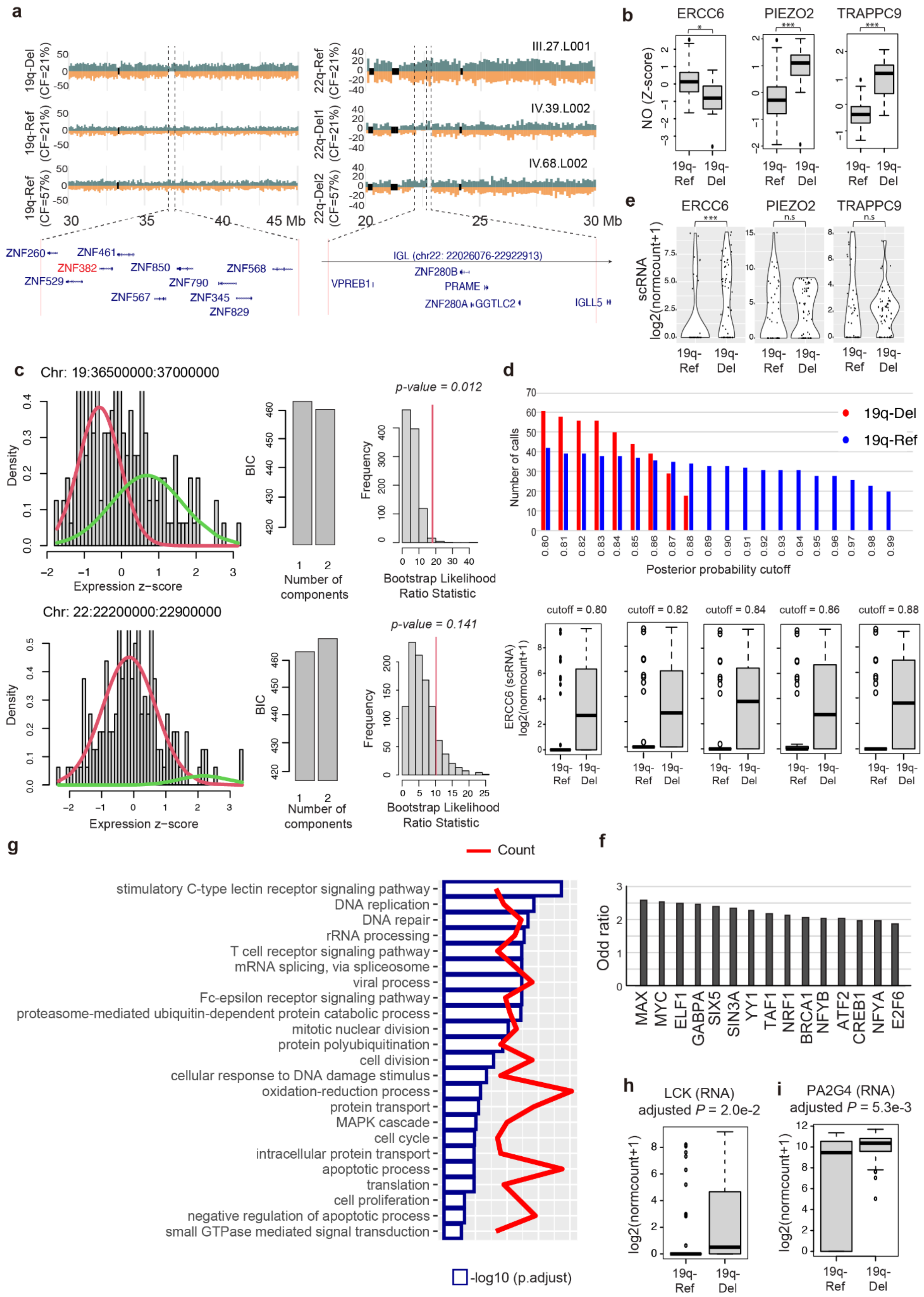

Figure S10. scNOVA analysis of SV subclones in NA12878 and their validation using scRNA-seq. (**a**) Mutually exclusive subclonal SVs in NA12878 single-cells. Del, Del1: hemizygous deletions; Del2: small homozygous loss region. Labels on the right side indicate single cell IDs. 19q-Del: subclone bearing 19q13.12 deletion. 19q-Ref: subclone bearing a not rearranged chromosome 19. (**b**) The boxplot of NO at gene bodies for three significant hits from scNOVA comparing chr19 deletion subclone (19q-Del) and the 19q- Ref subclone.

(p.adjust=0.063, 0.0043, 1.39E-05 for the *ERCC6, PIEZO2, TRAPPC9* respectively; two-sided Wald test followed by Benjamini Hochberg multiple correction; ***p.adjust<0.01, **p.adjust<0.05,*p.adjust<0.1; n = 16 and 60 cells for 19q-Del and 19q-Ref, respectively). **(c)** Analysis of NA12878 using CONICSmat[24] to perform targeted SCNA recalling of the 19q13.12 deletion region using published[25,26] Fluidigm and Smart-seq scRNA-seq data. Upper panel shows the histogram of mean expression Z-scores of the genes located within the 19q13.12 deletion region (chr19:36.5Mb-37Mb). CONICSmat fits these distributions to 1-component (absence of subclonal copy number changes) and 2-component (presence of subclonal copy number changes) mixture models, and compares log likelihood ratio of two models to evaluate significance of difference between two models (P<0.00012; two-sided Chi-square likelihood ratio test). The result of 2-component model fits are shown in the plot with red and green bimodal peaks. The bar graphs show the Bayesian information criterion (BIC) value of two models. The model with the lowest BIC (2-component) is preferred, suggesting the presence of an SCNA at 19q13.12. Lower panel shows the histogram of mean expression Z-scores of the genes for 22q11.2 deletion region (chr22:22.2Mb-22.9Mb). For this region, the BIC prefers a 1-component model (absence of SCNAs). The third plot (Bootstrap likelihood ratio statistic) shows the bootstrapping analysis to test the null hypothesis of a 1-component fit versus the alternative hypothesis of a 2-component fit based on gaussian mixture distribution. (**d**) In the case of the 19q13.12 region, for which CONICSmat inferred the 2-component model as the preferred model, the posterior probabilities of each individual cell were calculated to infer membership to the first component and the second component, and hence assign single cells as a "confident 19q-Del" (red) or "confident 19q-Ref cell" (blue). The bar graph shows the number of single-cells assigned to either confident 19q-Del or confident 19q-Ref for different posterior probability cutoffs. The box plots below show the comparison of *ERCC6* expression level from confident 19q-Del and confident 19q-Ref cells with different posterior probability cutoffs. These analyses are in strong support of the overexpression of *ERCC6* in cells exhibiting a 19q13.12 deletion (P=0.0067, 0.0050, 0.0051, 0.0092, and 0.052 for the posterior probability cutoffs of 0.80, 0.82, 0.84, 0.86, and 0.88 respectively; FDR-adjusted two-sided Wilcoxon rank sum test; derived from n = 160 cells in total) (**e**) Violin plots showing the RNA expression level of all three scNOVA hits in confident 19q-Del and confident 19q-Del cells in scRNA-seq defined with CONICSmat posterior probability 0.8. Differential expression between two groups were tested by FDR-adjusted two-sided Wilcoxon rank sum test (p.adjust=0.0067, 0.72, 0.59 for the *ERCC6, PIEZO2, TRAPPC9* respectively; ***p.adjust<0.01, **p.adjust<0.05,*p.adjust<0.1). **(f)** Odds ratio of TF targets enriched in cells bearing the 19q13.12 deletion, with c-Myc/Max target genes ranking highest (TF targets shown in this display exhibit adjusted P<1e-25 and combined score >100 based on EnrichR[27]), based on scRNA-seq in NA12878. **(g)** Gene ontology biological process (GOBP) terms over-represented among 1,896 up-regulated genes (FDR 1%, above 1.5 fold changes) in cells bearing the 19q-Del (61 cells) compared to 19q-Ref (42 cells), based on scRNA-seq (applying a posterior probability cutoff of 0.80). GOBPs with FDR 10%, computed using Fisher's exact test with DAVID software[28], with more than 10 up-regulated genes are shown. The bar graph shows -log10 (p.adjust) values, and the red line represents the number of up-regulated genes for each term. **(h-i)** Depiction of representative up-regulated c-Myc/Max target genes implicated in cell proliferation, based on the NA12878 scRNA-seq data (two-sided wilcoxon ranksum test followed by Benjamini Hochberg multiple correction; n = 71 and 42 cells for 19q-Del and 19q-Ref, respectively). Boxplots were defined by minima = 25th percentile - 1.5X interquartile range (IQR), maxima = 75th percentile + 1.5X IQR, center = median, and bounds of box = 25th and 75th percentile throughout this figure.

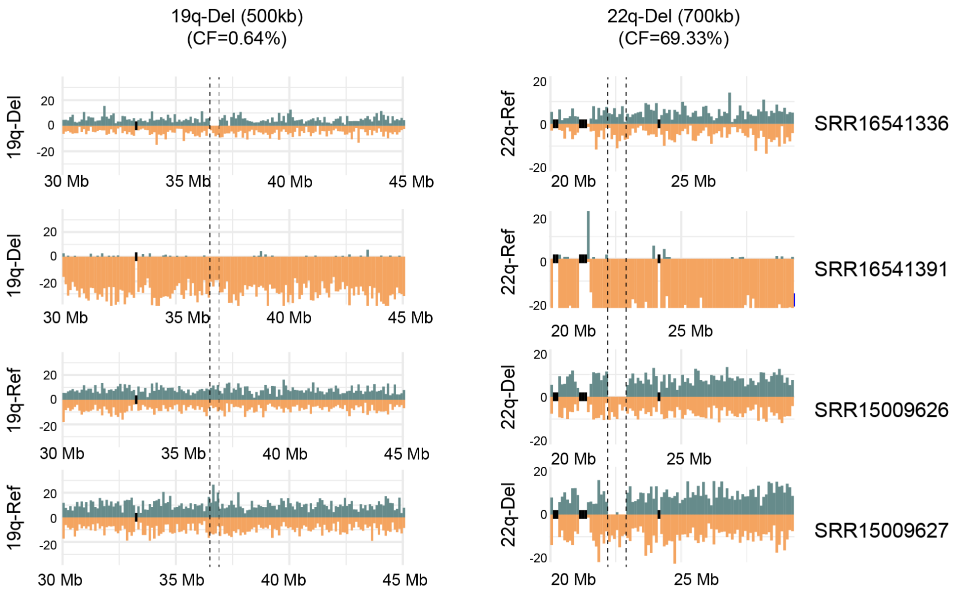

Figure S11. Validation of deletions in NA12878 in Strand-seq libraries generated in open nanoliter arrays. To confirm the presence of subclonal focal deletions in chr19q and chr22q in NA12878, we downloaded publicly available single-cell DNA sequencing data profiled by open nanoliter array based (OP) Strand-seq[59]. In total we analyzed 313 high quality single-cell libraries, and applied ArbiGent[60] for SV presence/absence calling of the 19q and 22q SVs discovered by the scNOVA framework (see main text). This analysis validated the 19q deletion in two single-cells (CF = 0.64%) as shown in the left panel (SRR16541336, SRR16541391). We additionally confirmed 22q deletions in 217 single-cells (CF = 69.33%), shown in the right panel for representative single-cells (SRR15009626, SRR15009627). As in the scNOVA analysis described in the main text, somatic deletion events 19q and 22q were mutually exclusive in these OP Strand-seq data.

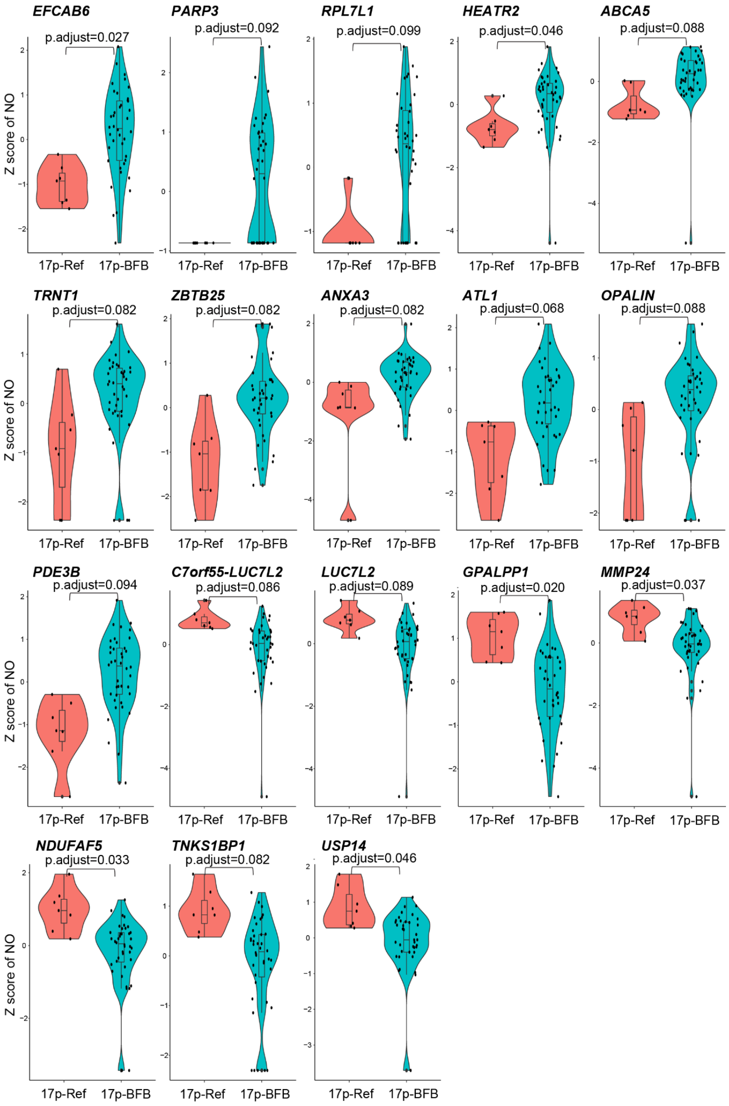

Figure S12. Violin plots of genes showing significant altered activity in the 17p-BFB clone compared to the 17p-Ref clone in NA20509. In total 18 genes were identified using 10% FDR cutoff. Z score of NO values at gene bodies, for each single cell, are depicted in violin plots (two-sided Wald test followed by Benjamini Hochberg multiple correction). This analysis was derived from n = 7 and 40 cells for 17p-Ref, and 17p-BFB, respectively. Boxplots were defined by minima = 25th percentile - 1.5X interquartile range (IQR), maxima = 75th percentile + 1.5X IQR, center = median, and bounds of box = 25th and 75th percentile.

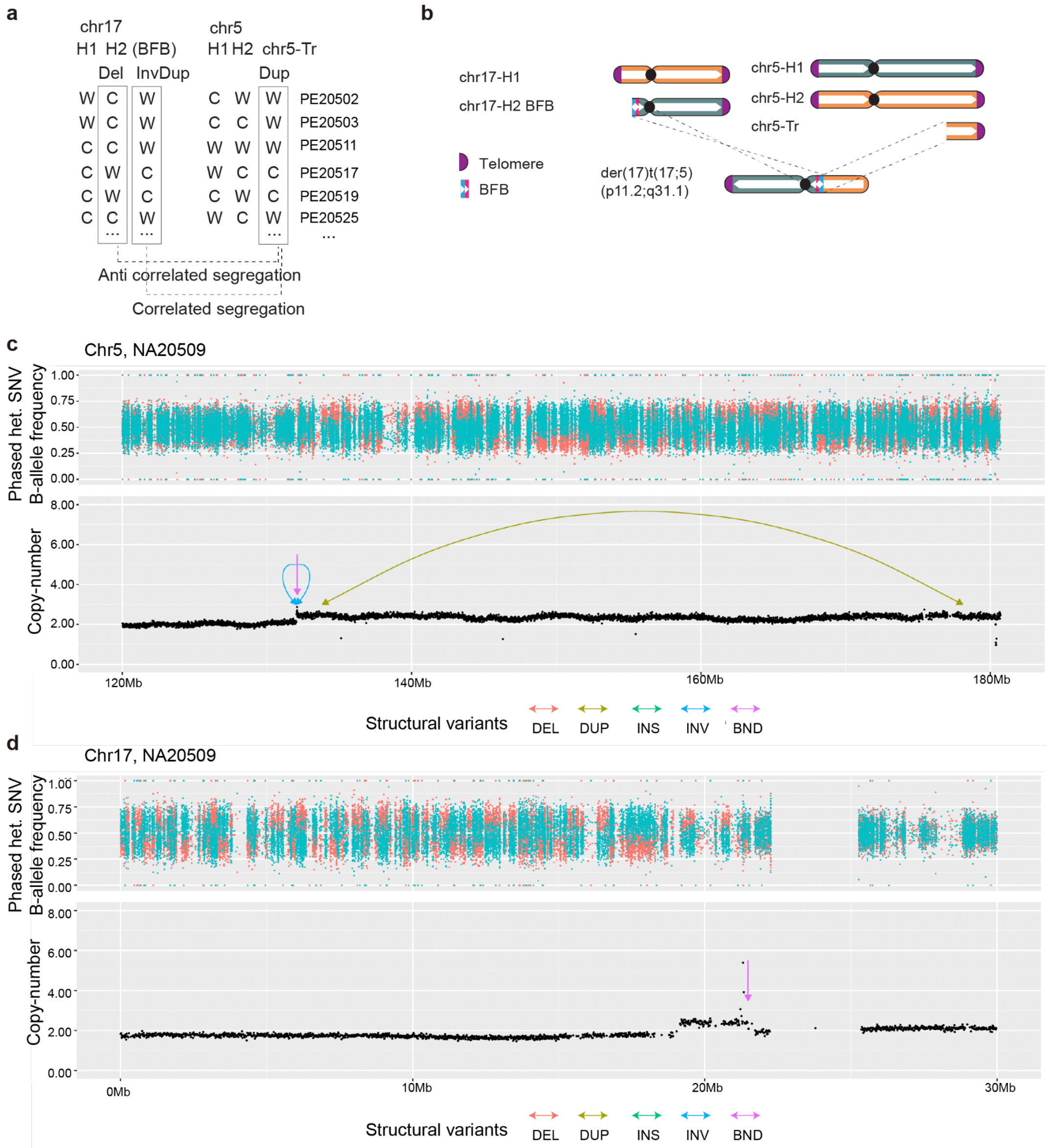

Figure S13. BFB-mediated subclonal complex rearrangement in NA20509. (**a**) Discovered unbalanced translocation with CF=85% (*P*=1.3e-07; FDR-adjusted Fisher's exact test; inversely correlated template strand co-segregation patterns[1] used for translocation discovery shown for six representative cells). (**b**) Subclonal karyotype of NA20509, with complex derivative chromosome highlighted. **(c-d)** Read depth plot and SV calls based on NA20509 (bulk-cell) WGS data, generated at the New York Genome Center using a different cell stock of NA20509 than used for Strand-seq library preparation of NA20509 (pursued at EMBL Heidelberg). WGS data were downloaded from the data portal of the International Genome Sample Resource (IGSR)[29], and analyzed as described in the **Supplementary Notes**. Phased heterozygous sites for haplotype 1 (cyan color) and haplotype 2 (salmon color) together with read-depth based copy numbers profiles verified **(c)** the presence of complex subclonal SVs including a large gain on chromosome 5, **(d)** subclonal terminal loss of chromosome 17 p-arm, and subclonal gain at 17p. SV analysis using Delly2[30] additionally verified the presence of a subclonal unbalanced translocation between chromosomes 5 and 17 (labeled 'BND'), showed a tail-to-tail inversion-type rearrangement at chromosome 5, and identified a tandem duplication-type rearrangement signature spanning parts of the terminal gain on chromosome 5. Further inspection of the Illumina WGS data suggested presence of the rearrangement-bearing subclone in 30-34% of cells (based on inspecting the core amplified region on chr5 and the core deleted region on chr17, respectively; see panels **(c)** and **(d)**). This is lower than the 85% CF detected in our own cell stock at the EMBL, perhaps since the CF of the respective subclone varies between distinct NA20509 cell stocks.

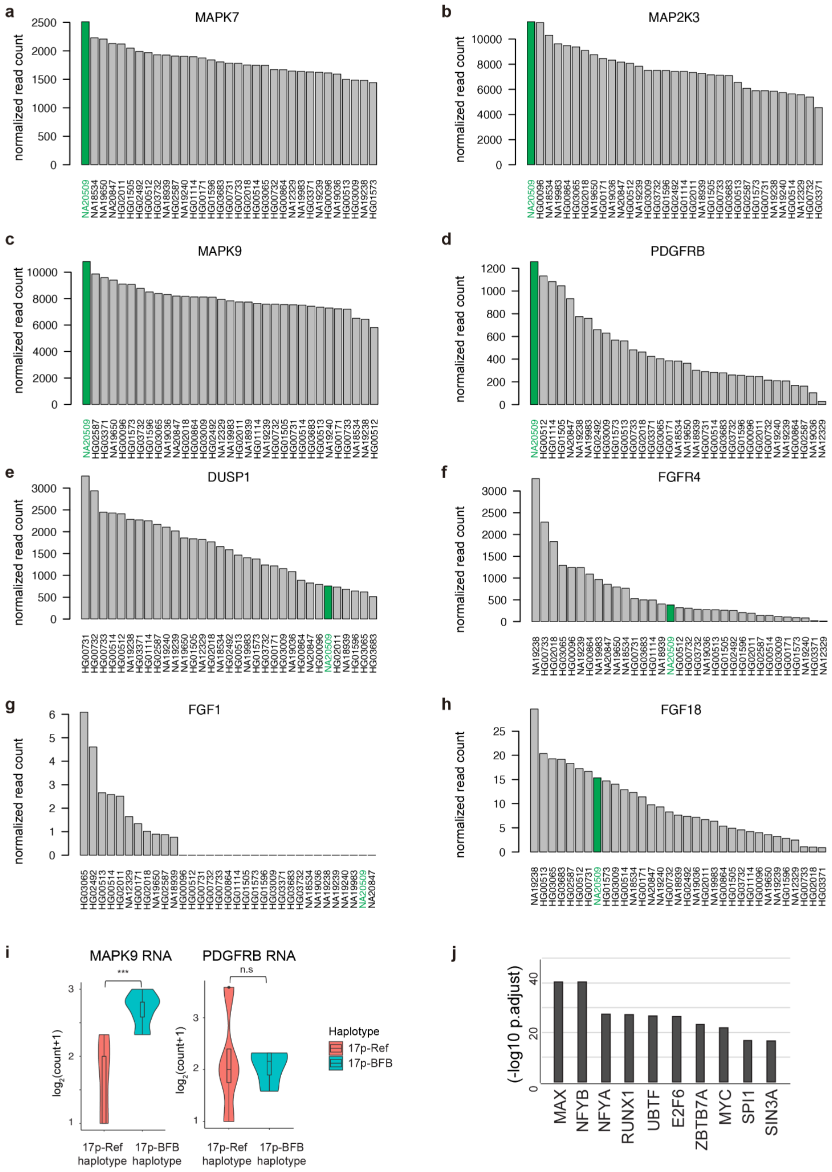

Figure S14. Transcriptome analysis in a panel of 33 LCLs with available[31] deep bulk-cell RNA-seq data. The figure depicts normalized read counts of MAPK pathway genes located in the duplicated region on chromosomes 17 **(a-b)** and 5 **(c-h)**. The expression level of NA20509 is highlighted with green. *MAP2K3* is an upstream regulator of c-Myc/Max in the MAPK signaling pathway. **(i)** Haplotype resolved RNA-seq read counts at heterozygous SNP sites within the *MAPK9* showed expression from both homologs, but also with increased expression in the BFB haplotype (17p-BFB) compared to the not rearranged haplotype (17p-Ref) (FDR-adjusted *P*=2.4e-3 and *P*=0.91 for *MAPK9* and *PDGFRB*, respectively; two-sided likelihood ratio test, ***p.adjust<0.01, **p.adjust<0.05,*p.adjust<0.1; derived from n = 4, and 12 heterozygous SNP sites for *MAPK9* and *PDGFRB* respectively; Boxplots were defined by minima = 25th percentile - 1.5X interquartile range (IQR), maxima = 75th percentile + 1.5X IQR, center = median, and bounds of box = 25th and 75th percentile.) suggesting that this gene might also contribute to the molecular phenotype (activation of c-Myc/Max targets) in the genomically rearranged subclone. (**j**) Significant TFs identified from TF-target over-representation analysis of differentially expressed genes in NA20509 late (p8) compared to early (p4) based on RNA-seq analysis. Top 10 significant TFs were shown in the bar graph (FDR 10%). This analysis identified Max, and its dimerization partner of c-Myc, independently as significant hits (FDR 10%). By comparison, we did not observe changes in expression of *MYC* at p8 versus p4. This is consistent with scNOVA, which predicted no *MYC* expression change in 17p BFB cells, and could be explained by c-Myc post transcriptional regulation following deregulation of *MAP2K3*[32].

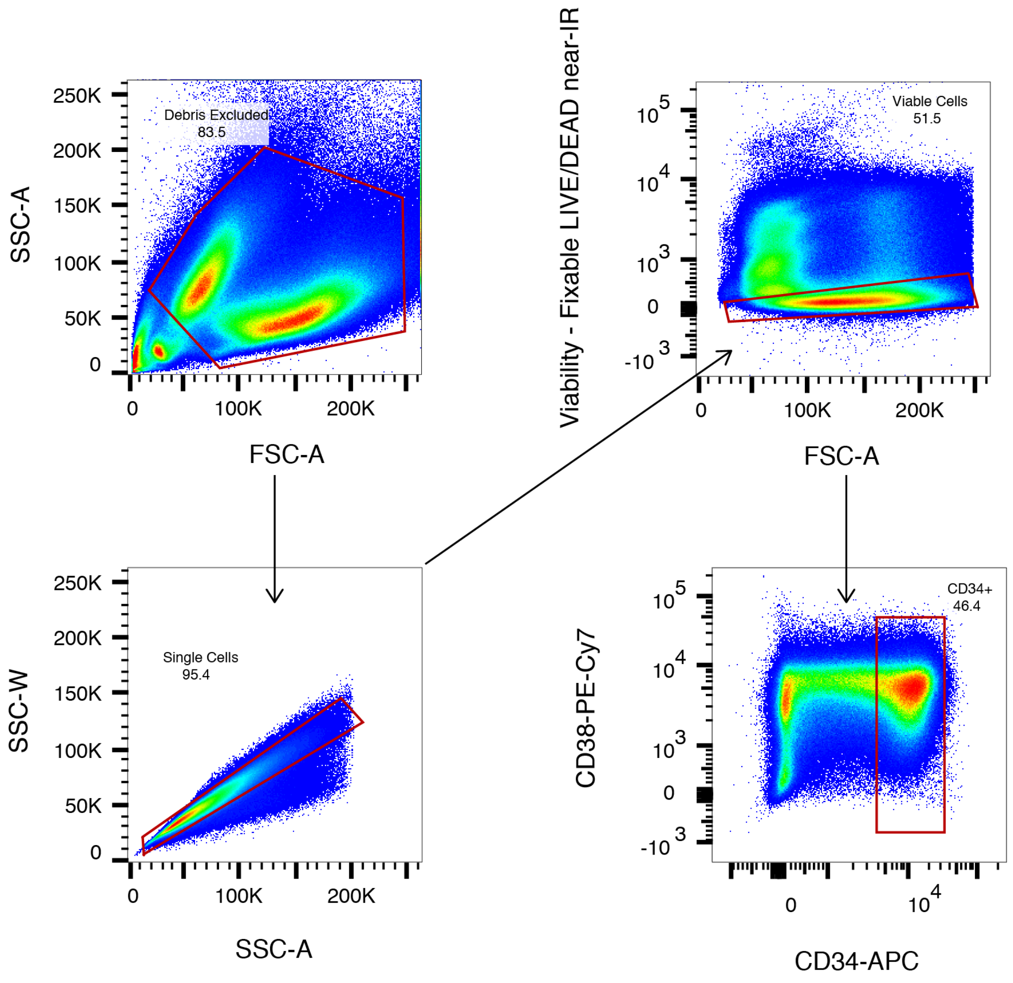

Figure S15. Gating strategy for isolation of CD34+ cells from AML patient AML_1.
Mononuclear cells from bone marrow aspirates were thawed and stained (**Methods**). Extracellular debris was gated out based on its low FSC-A vs SSC-A profile relative to cells. Doublets discrimination and exclusion was carried out by removing outliers in SSC-W vs SSC-A profiles; where doublets appear as outliers. Viable cells were identified by a low staining with Fixable LIVE/DEAD stain, an intracellular stain which does not strongly penetrate viable, intact cells. Finally, CD34+ cells were sorted from these single, viable cells. The red gate shows the selected population which is visualized in the consequent plot, indicated by an arrow. The final red gate indicates the population that was sorted, and used for Strand-seq library preparation.

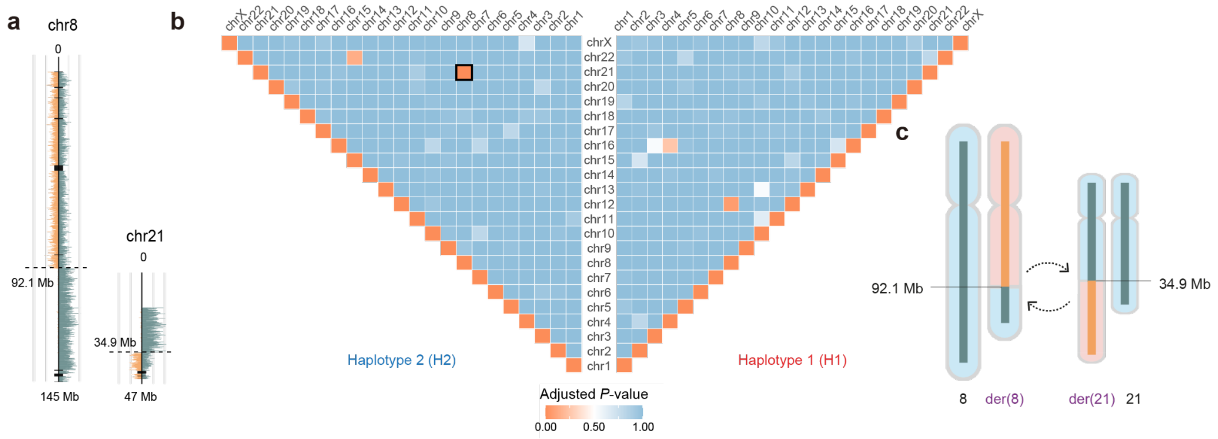

Figure S16. Identification of balanced translocation in AML_1. (**a**) Strand-seq based chromosome plot of representative single-cell from AML_1, which shows breakpoints at 92.1Mb of chromosome 8 and 34.9 Mb of chromosome 21. (**b**) TranslocatoR analysis (implemented in mosaiCatcher)[1] results are depicted in a pyramid plot. Each pixel in the pyramid represents the significance of co-segregation between two chromosomal segments, allowing to detect potential translocation partners. This analysis indicates that AML_1 contains a t(8;21) clonal translocation, consistent with clinical diagnosis. (*P*-value for translocation discovery using strand co-segregation: *P*=0.00003, FDR-adjusted Fisher's exact test). (**c**) The schematic diagram shows a normal and a derivative chromosomes resulting from t(8;21) translocation.

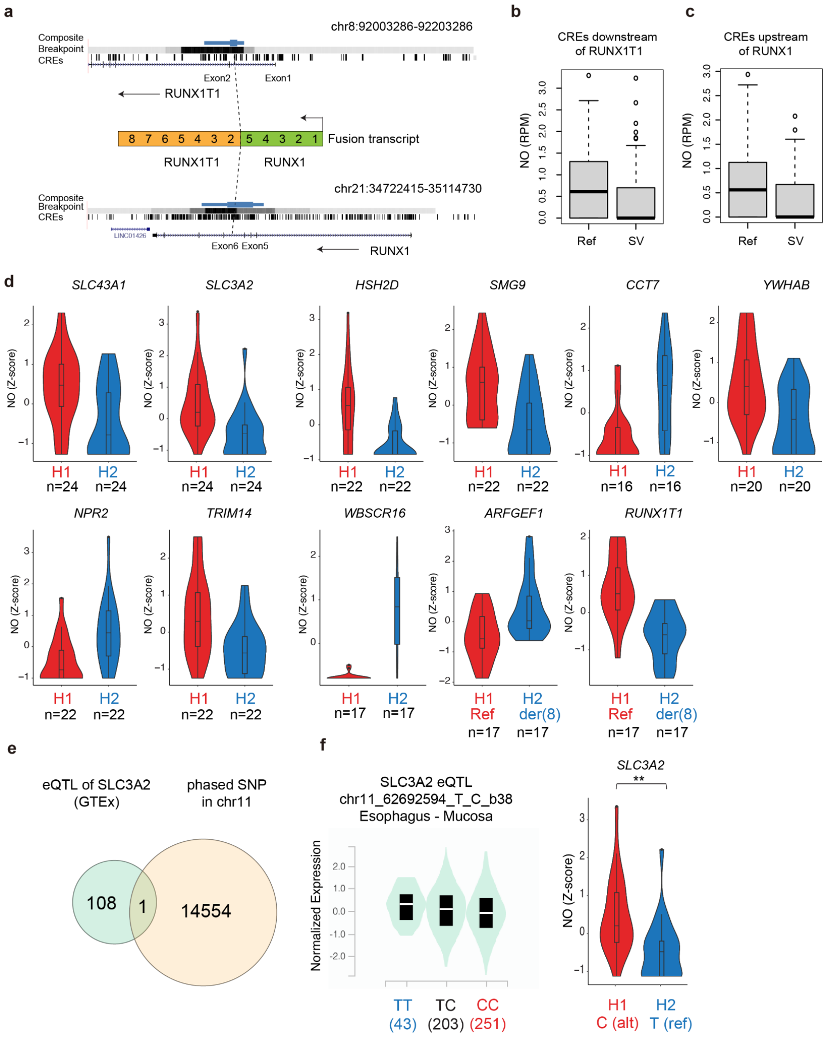

Figure S17. Haplotype-specific NO analysis in AML_1. (**a**) Strand-seq based breakpoint analysis using BreakpointR[44] located the translocation breakpoint in AML_1 within intron 1 of *RUNX1T1* on chromosome 8, and intron 5 of *RUNX1* on chromosome 21, recapitualing previously reported locations of t(8;21)(q22;q22.1) breakpoints[45]. CREs were defined as the union of ATAC-seq peaks from AMLs and the hematopoietic system from prior studies (**Table S5, Methods**). (**b**) Haplotype-specific NO at CREs within -278 to 22kb, adjacent to the translocation breakpoint which contains part of *RUNX1T1* (*P*<0.08; likelihood ratio test, adjusted using permutations; derived from n = 17 cells with WC/CW configuration in chromosome 8). Ref, not arranged homologue of chromosome 8. SV, translocated (derivative chromosome). (**c**) Haplotype-specific NO at CREs in the upstream segment residing between 0.82Mb and 1.12Mb of *RUNX1* (*P*<0.003; likelihood ratio test, adjusted using permutations; derived from n = 16 cells with WC/CW configuration in chromosome 21). **(d)** 11 genes demonstrate significant haplotype-specific NO in their gene bodies, genome-wide (FDR<10%; Wilcoxon rank-sum test; n indicates the number of cells with WC/CW configuration used for the statistical testing,). (**e-f**) eQTL analysis identified a SNP that may explain patterns of haplotype-specific NO seen for *SLC3A2*. (**e**) Among 109 eQTL SNPs known to be associated with *SLC3A2* gene expression level according to GTEx database[46], one SNP (rs2850596) could be unambiguously phased using Strand-seq data of the AML_1 sample. (**f**) Violin plot downloaded from GTEx shows that 'T' allele of rs2850596 is associated with higher expression of *SLC3A2* compared to the 'C' genotype. This may explain patterns of haplotype specific NO seen for the *SLC3A2* gene. Asterisk denotes FDR adjusted p-value (p.adjust = 0.0033 from two-sided wilcoxon ranksum test followed by Benjamini Hochberg multiple correction; ***p.adjust<0.01, **p.adjust<0.05,*p.adjust<0.1; derived from n = 24 cells with WC/CW configuration in chromosome 11). Boxplots were defined by minima = 25th percentile - 1.5X interquartile range (IQR), maxima = 75th percentile + 1.5X IQR, center = median, and bounds of box = 25th and 75th percentile throughout this figure.

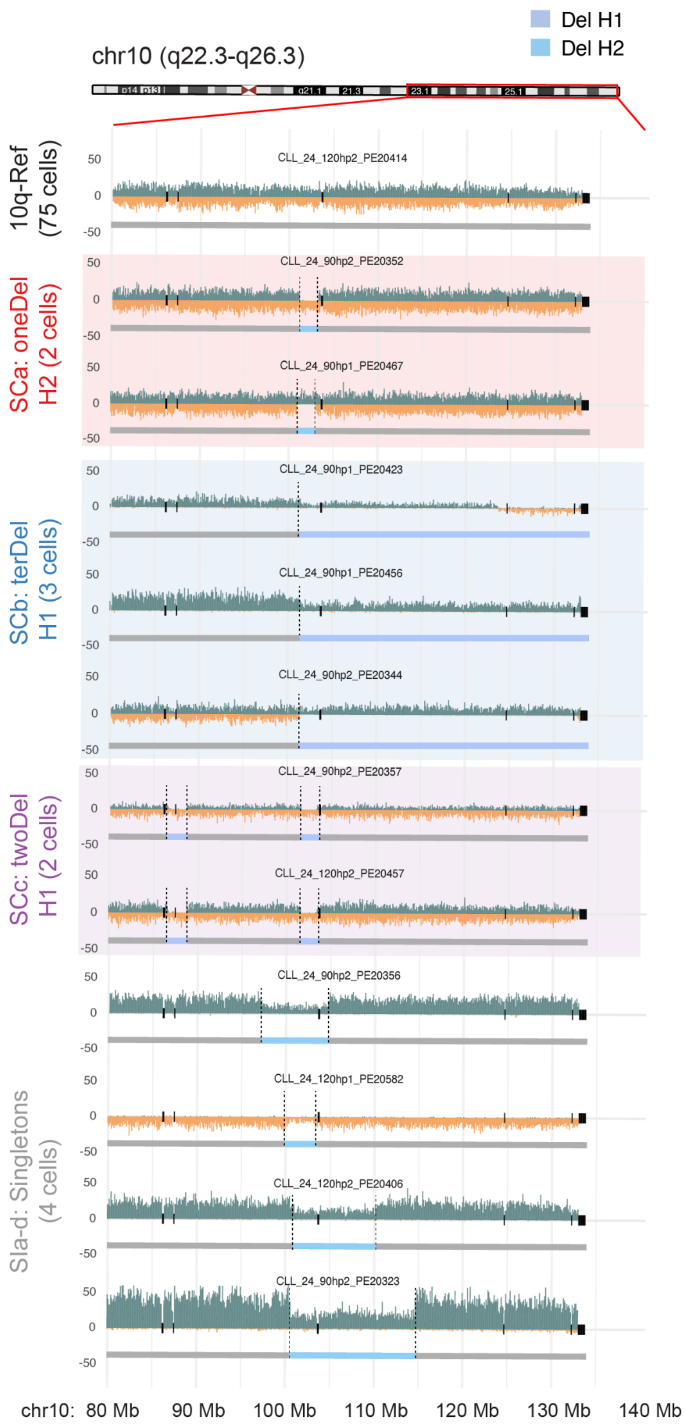

Figure S18. Single-cell SV discovery in CLL_24. A diversity of distinct yet overlapping deletions were observed at chromosome 10q24.32, representing subclones and single cells. 10q-Ref, not rearranged karyotype. SIa-d (singletons), here represented as a 'group' of four single cells with distinct/individual deletions, all bear deletions affecting the 'minimal region' (see main text). As we identified extensive subclonal heterogeneity in this sample, we used the lenient SV calling parameterization available using the MosaiCatcher pipeline[1] to allow for sensitive detection of SVs at CFs from 1 to 5%, including in individual cells.

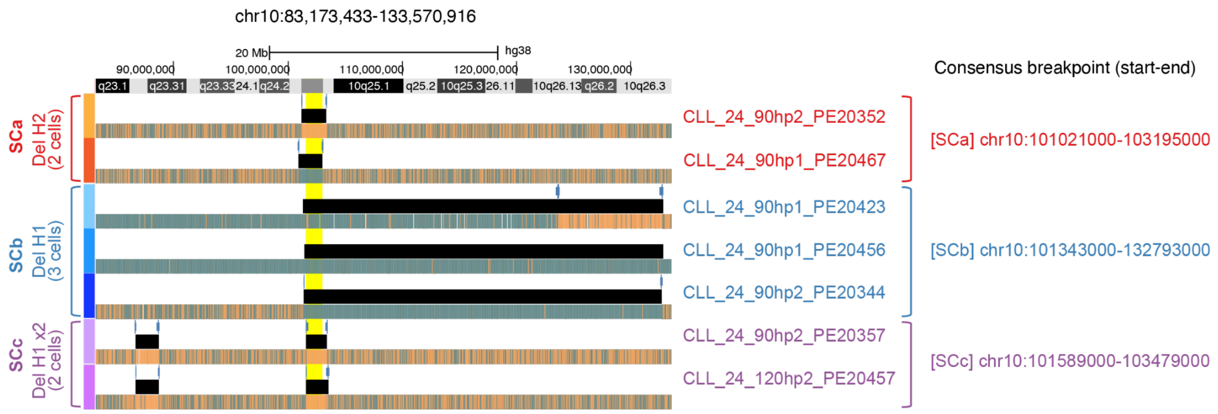

Figure S19. Investigation of relationships between SCa, SCb and SCc clones in CLL_24. Genome browser tracks showing the haplotype (denoted as H1, H2) and location of deletions for each single cell based on BreakpointR[44] analysis of Strand-seq data. In total seven cells belonging to the SCa, SCb, SCc clones were shown. For each of the single-cell libraries, three tracks are depicted in the plot: 1) confidence interval of breakpoints; 2) deleted position (black bar), and 3) composite read track. On the right-side of the browser track, consensus breakpoints of each subclone were shown. Based on these data, we formally tested the hypothesis that SCb and SCc might be daughter clones of SCa. This analysis showed that the SCa deletion arose on haplotype 2, whereas the deletions on SCb and SCc arose on haplotype 1 – which formally rules out such a relationship. Consistent with this haplotype based analysis, we found that the confidence intervals of the SCa deletion breakpoint do not overlap with the breakpoints of SCb and SCc (with the intervals being 322kb and 568kb apart, respectively).

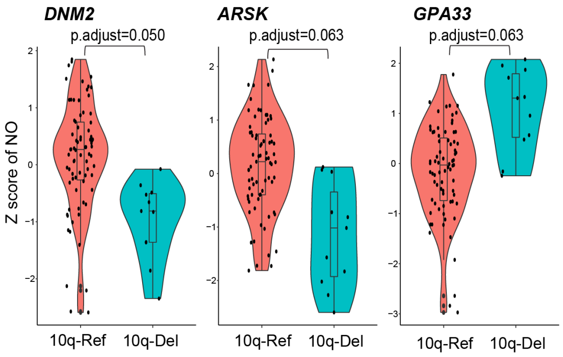

Figure S20. Violin plots of genes showing significant altered activity in the 10q-Del clone compared to the 10q-Ref clone in CLL_24. Three genes were identified using 10% FDR cutoff. The Z score of NO at gene bodies for each single cell in the 10q-Del clones and 10q-Ref clone is depicted in violin plots (two-sided Wald test followed by Benjamini Hochberg multiple correction). This analysis was pursued using n = 75 and 11 cells for 10q-Ref, and 10q-Del, respectively. Boxplots were defined by minima = 25th percentile - 1.5X interquartile range (IQR), maxima = 75th percentile + 1.5X IQR, center = median, and bounds of box = 25th and 75th percentile.

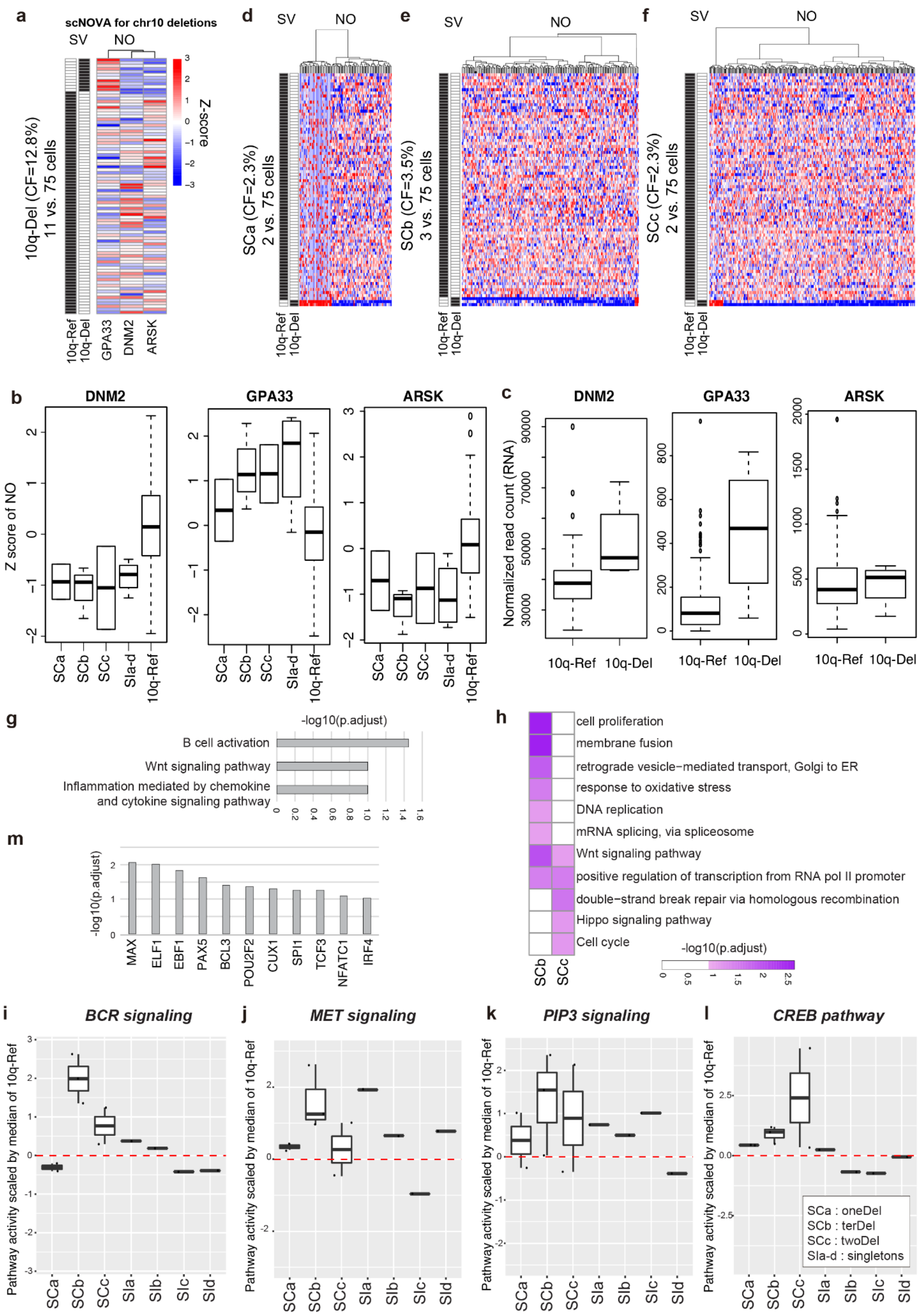

Figure S21. Inference of altered gene activities in CLL_24 subclones. We first applied the default scNOVA mode to compare all cells with a deletion at 10q24.32 ('10q-Del', *N*=11) to cells without this SV ('10q-Ref', *N*=75), hence disregarding the fine scale subclonal structure of CLL_24. This identified only three differentially active genes - *GPA33*, *ARSK* and *DNM2* - all of which reside on other chromosomes. **(a)** Heatmap of three genes altered in activity in cells bearing 10q24.32 deletions, using the default mode (DESeq2) of scNOVA (10% FDR). **(b)** NO of three significant hits from the default mode of scNOVA (10% FDR). '10q-Ref' denotes a not rearranged karyotype. SCa, SCb and SCc denote subclones harboring deletions (Del) in the 'minimal deleted region' at 10q24.32 (see main text; *N* = 2, 3, and 2 cells for SCa, SCb, and SCc, respectively). SIa-d combines four single cells that exhibit individual deletions in the same minimal deleted region (deletions seen in *N*=1 cell each). **(c)** Differential expression of genes of interest in an ICGC CLL cohort[33], in samples bearing a 10q24.32 deletion

compared to donors without such deletion ('10q-Ref'). Transcriptome-wide differential expression analysis showed up-regulation of *DNM2* (FDR 10%), supporting the inferences initially made using scNOVA (see main text). While *DNM2* over-expression in deletion donors recapitulates the altered gene activity in the SV subclone (p.adjust = 0.0696), expression changes of *GPA33* and *ARSK* were not significant in the comparison of donors with and without 10q deletion (p.adjust = 0.220, 1.000 for *GPA33* and *ARSK*, respectively). (**d-f**) Considering the 10q24.32 deletion subclones were seen at low-frequency (CF ≤3.5%), we next applied the alternative mode of scNOVA (PLS-DA-based; **Methods**). Heatmaps of genes altered in activity in different subclones (SCa, SCb, and SCc) bearing 10q24.32 deletions, using alternative the mode (PLS-DA) of scNOVA (10% FDR) are shown in panel (**d**) to (**f**) respectively. (**g**) Functional enrichment analysis of genes predicted from scNOVA's altered gene activity module (alternative mode using PLS-DA, **Fig 4c**) for 11 cells bearing 10q deletions (10q-Del) versus 75 cells without deletions (10q-Ref). **(h)** Functional enrichment analysis of genes predicted from scNOVA's altered gene activity module for individual subclones with chromosome 10q deletions. As the CFs of distinct subclones were below 10%, we use the alternative mode for inferring altered gene activity. This analysis predicted 109, 206, 266 genes with altered activity. Gene set over-representation analysis was performed using DAVID software[28]. **(i-l)** Pathway activities derived by jointly modeling NO at the gene bodies of gene sets for (**i**) BCL signaling, (**j**) MET signaling, (**k**) PIP3 signaling, and (**l**) CREB pathway in different subclones. Pathway activity ((-1)*Z-score of NO) was scaled by the median seen across unaffected (10q-Ref) cells from the CLL_24 sample. n = 2, 3, 2, and 1 cells for SCa, SCb, SCc, and SIa-d were shown, respectively. (**m**) Significant TFs identified from TF-target over-representation analysis of scNOVA hits (activated in SCb; 10q-terDel), supported by CITE-seq analysis of 10q-terDel clone (FDR 10%; Fig. 4h). Boxplots were defined by minima = 25th percentile - 1.5X interquartile range (IQR), maxima = 75th percentile + 1.5X IQR, center = median, and bounds of box = 25th and 75th percentile throughout this figure.

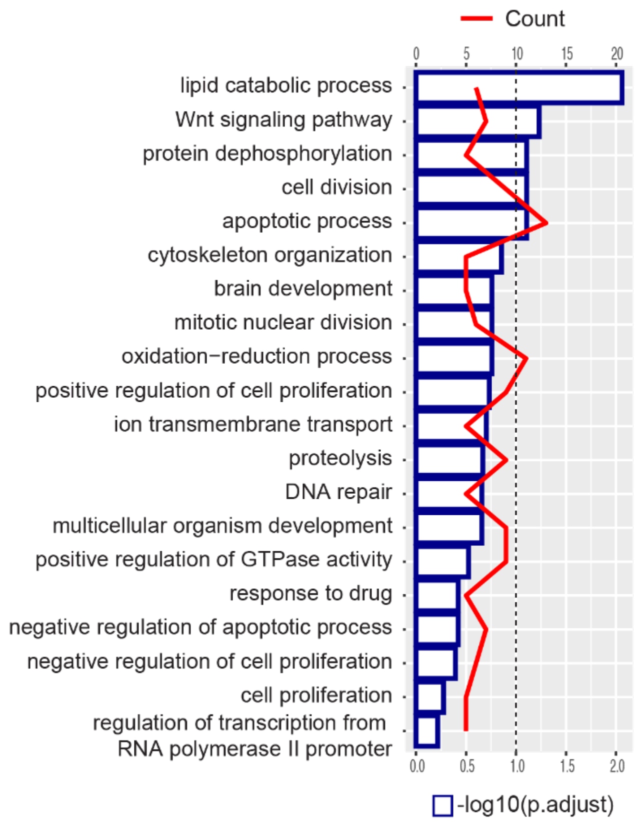

Figure S22. Functional enrichment analysis of genes clustering genomically at 10q23.2-26.3. In total, 235 genes were affected by at least one of the 10q-Del events in CLL_24. These 235 genes were tested for functional enrichment with the DAVID software[28], using Fisher's exact test, followed by FDR correction. The bar graph shows the -log10(p.adjust) value, and the line graph shows the number of genes clustering in this genomic region that are involved in each biological process. Five gene ontology biological processes reach significance at 10% FDR. This includes Wnt signaling, which represents the second most significantly enriched functional category – suggesting that Wnt signaling-related genes are clustered in this chromosomal region.

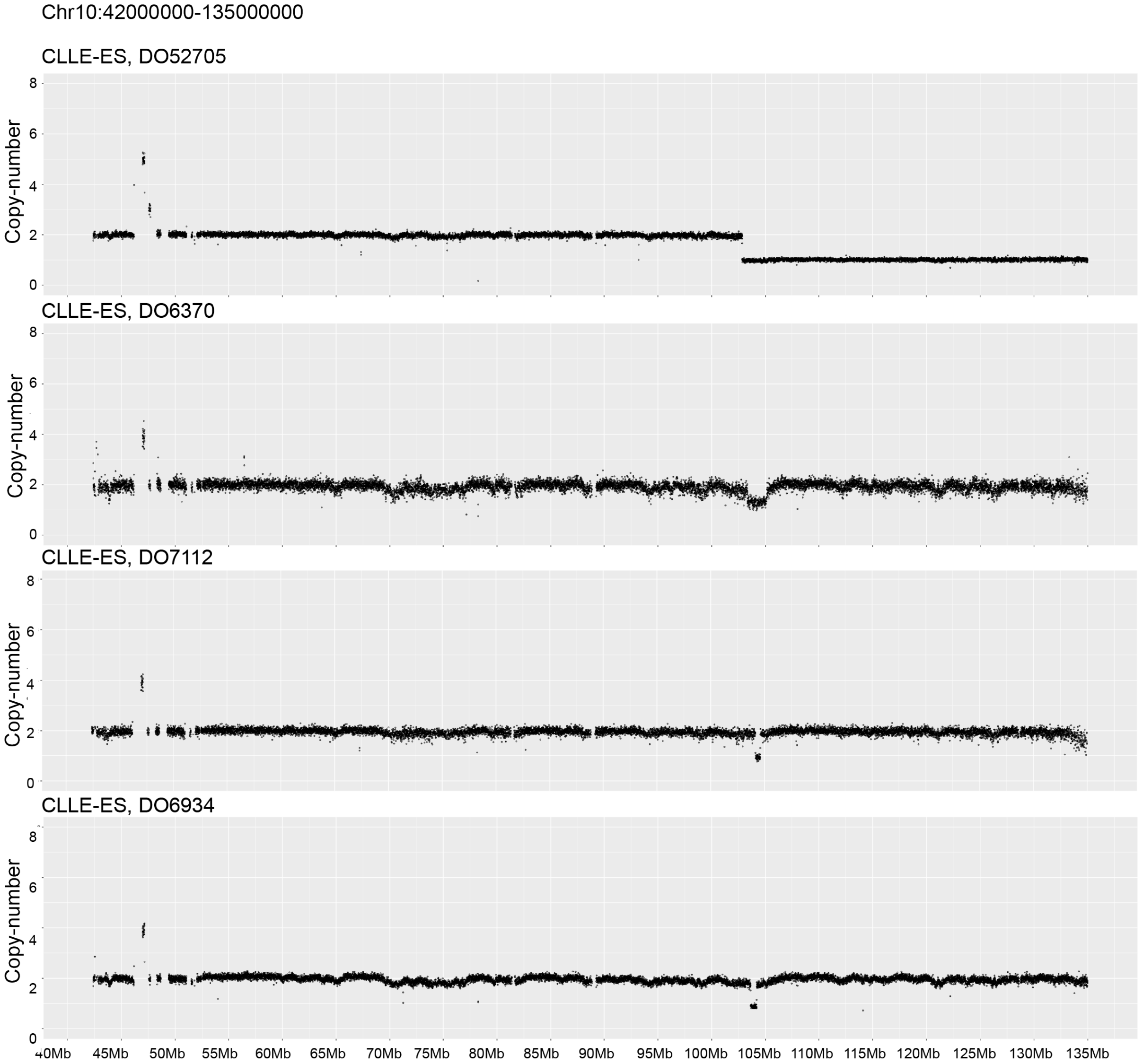

Figure S23. Recurrence of somatic deletions (Del) at the 10q24.32 'minimal deleted region', in CLL donors from PCAWG[34]. Analysis of these WGS datasets by read depth analysis, using Delly2[30], uncovered Dels intersecting with the minimally deleted segment, initially observed in CLL_24, in 4 out of 94 (>4%) cases (all cases shown above). Prior studies reported somatic 10q24.32 deletions in 1-4% of CLLs[35–37], and reported enrichment in relapsed/refractory and high-risk cases[38], suggesting these SVs may act as a driver.

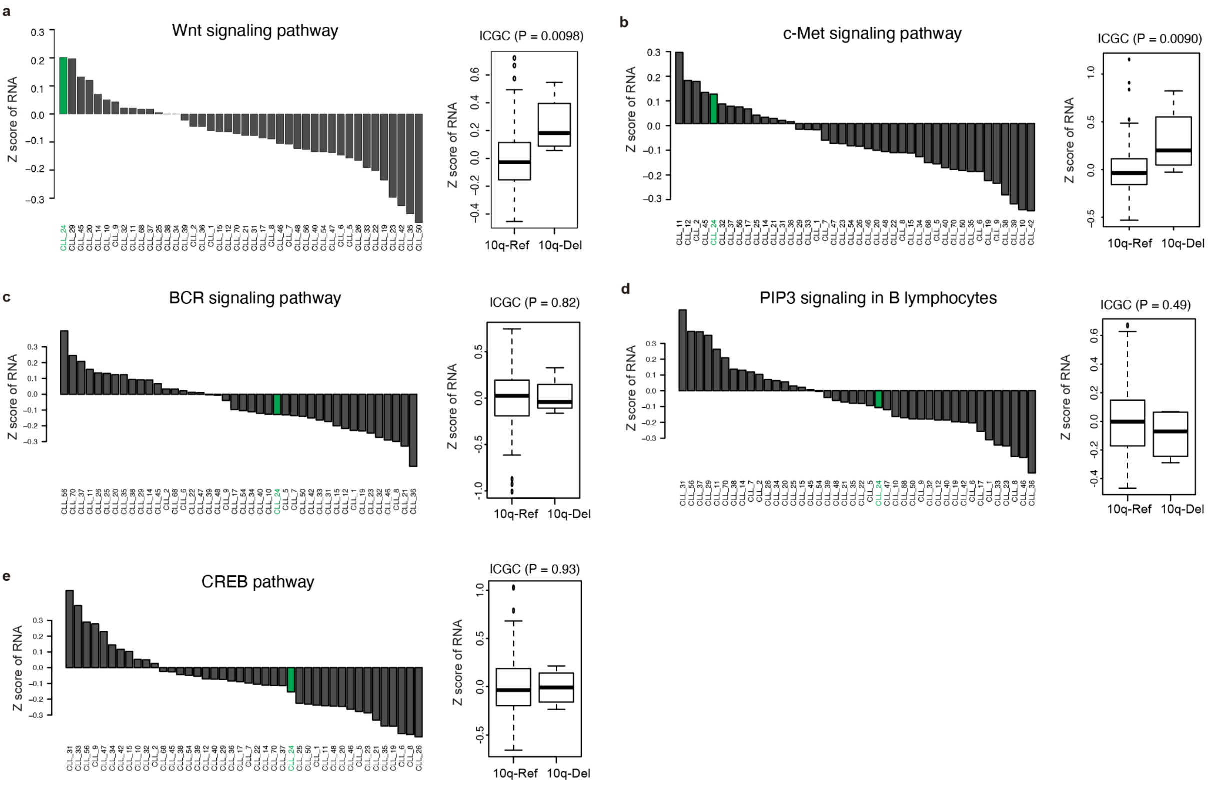

Figure S24. Validation of increased expression of leukemia related signaling pathways in association with 10q24.32 deletions in ICGC samples. (a) Left panel: Bulk RNA-seq analysis in 42 CLLs. Mean expression Z-scores for canonical Wnt signaling target genes are shown for each donor (green: CLL_24). Right panel: CLL samples from the ICGC[36] bearing deletions at the 10q24.32 minimal segment (10q-Del) show increased expression of Wnt pathway target genes compared to CLL samples with a not rearranged 10q karyotype (10q-Ref) (*P*=0.0090; two-sided likelihood ratio test; n = 174 and n = 4 independent CLL samples for 10q-Ref and 10q-Del, respectively; Boxplots were defined by minima = 25th percentile - 1.5X interquartile range (IQR), maxima = 75th percentile + 1.5X IQR, center = median, and bounds of box = 25th and 75th percentile throughout this figure.). Same analysis was applied for all the leukemia related signaling pathways identified in **Fig. 4d** including c-Met signaling pathway (**b**), BCR signaling pathway (**c**), PIP3 signaling in B lymphocytes (**d**), and CREB pathway (**e**). Note that c-Met signaling target genes (**Table S7**) were collected from prior literature, where potential c-Met target genes were defined using global gene expression profiling of wildtype and c-Met-deficient primary mouse hepatocytes[40]. According to the targeted analyses performed for these CLL samples as part of a clinical study, CLL_29 (2nd rank for Wnt signaling in **(a)**) is harboring a somatic 13q deletion, known to result in activation[41] of Wnt signaling via upregulation of *LEF-1*, which likely explains why the difference in Z-scores between CLL_24 and CLL_29 is relatively small. Similarly, CLL_11, CLL_12, CLL_2 (top ranking for c-Met signaling, which is promoted by the Wnt pathway[42]) contain either 13q (CLL_12, CLL_2) or 11q (CLL_11) deletion events. 11q somatic deletions result in activation[43] of Wnt signaling via *ATM* loss, followed by increase of *WNT5A*. These data are therefore broadly consistent with prior knowledge on the activation of Wnt signaling in CLL. Because the 10q24.32 region was not included in the targeted genotyping performed in the clinical trial, we do not know whether genetic alterations in this region additionally contributed to the abnormal activation of the Wnt and c-Met signaling pathways in these samples.

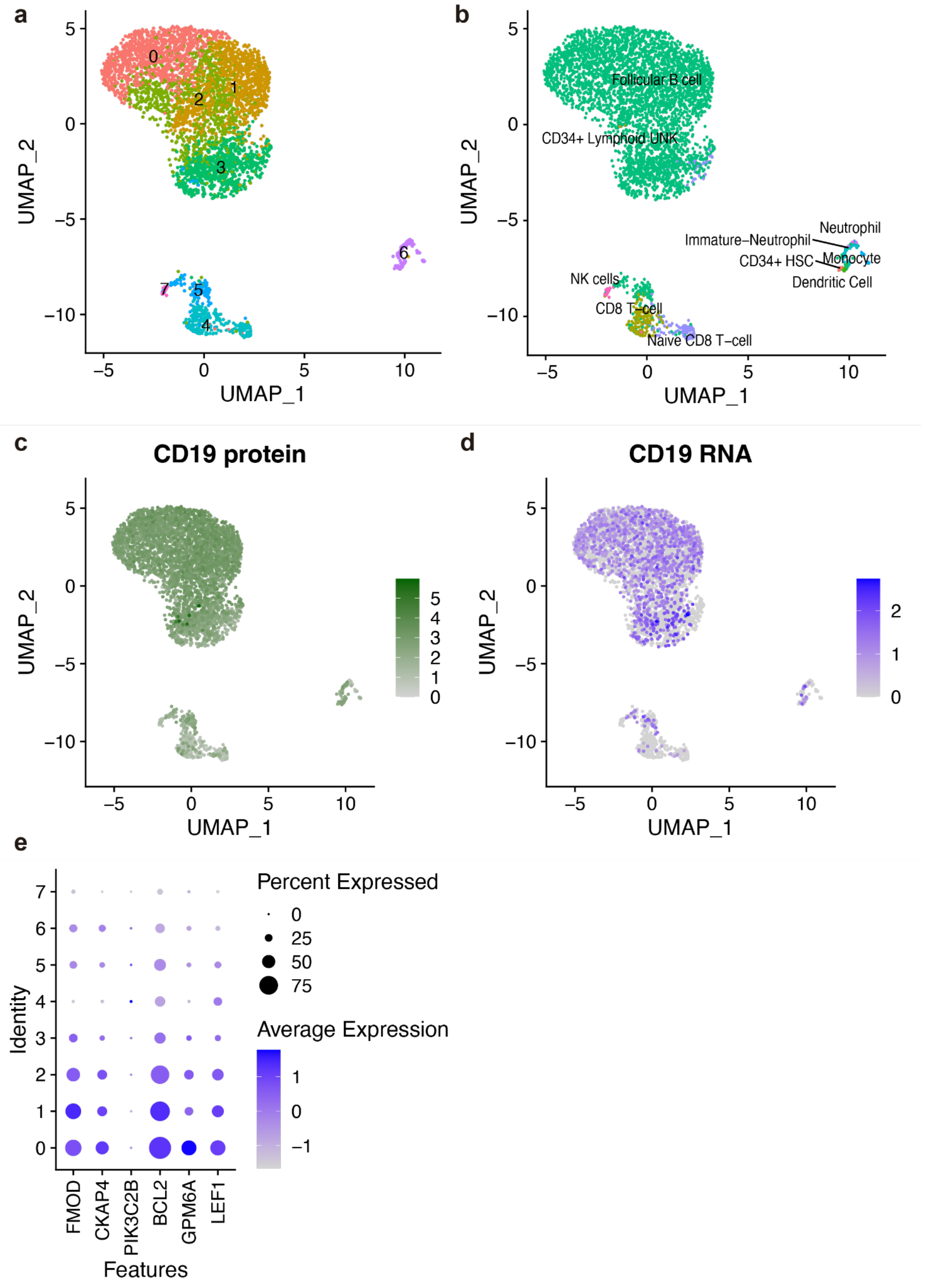

Figure S25. Transcriptional and protein markers, as well as reference-based cell-type annotations, discriminate B-cells from other contaminating immune cells. (**a**) Unsupervised clustering analysis of 4,459 high-quality single-cell RNA-seq libraries (CITE-seq), generated for CLL_24. (**b**) Reference-based cell type annotation of single-cell transcriptomes from CLL_24. Total bone marrow cells from the human cell atlas bone marrow dataset were used as a reference for cell type annotation[54]. (**c-d**) Average expression of CD19 protein (c) and RNA (d) across single cells. CD19 is a known marker of human B-cells. (**e**) Dotplot showing the average expression of 6 genes previously shown to distinguish leukemic B-cells from healthy lymphocytes[55].

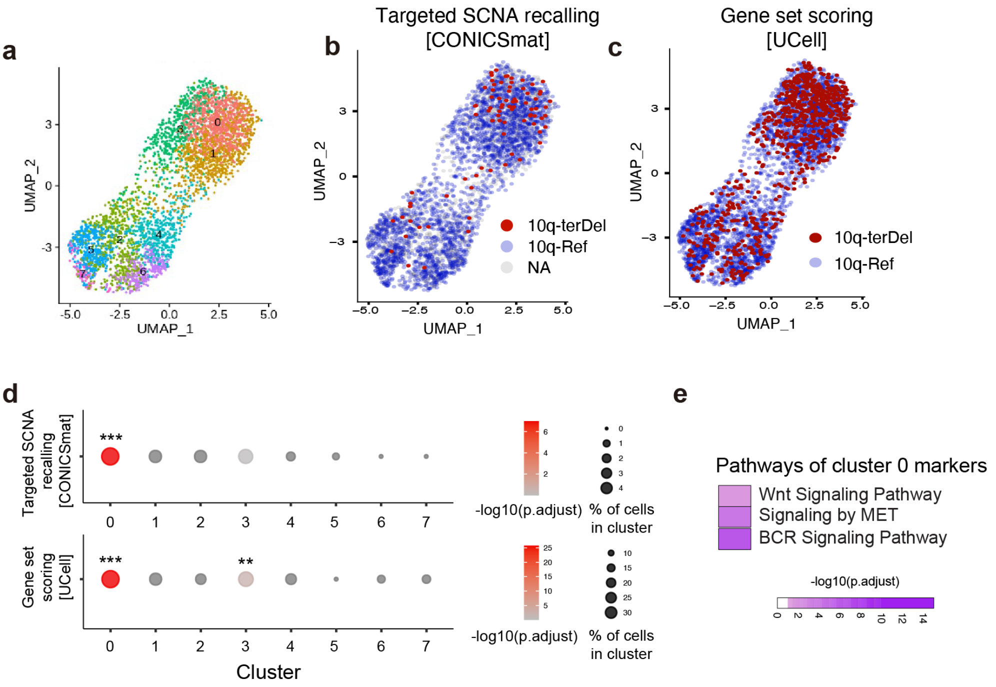

Figure S26. Inference of SCNAs using CITE-seq data from the CLL_24 sample. (**a**) UMAP of CITE-seq data based on DoroThEA TF activities[56] showing eight unsupervised clusters in CLL_24. (**b**) Single-cells confidently inferred to harbor the 10q-terDel event (shown in red) and cells confidently called '10q-Ref' (shown in blue) by targeted SCNA recalling were projected to the UMAP plot, revealing pronounced clustering of 10q-terDel cells in cluster 0 (see also panel (d)). (**c**) Single-cells inferred to exhibit the scNOVA-derived gene set of SCb (10q-terDel subclone) based on UCell (gene set UCell score > (median score + standard deviation)), are assigned to '10q-terDel', and shown in red; the remaining cells did not meet the threshold were assigned to '10q-Ref'. (**d**) Upper plot: Dot plot showing over-representation of CONICSmat (targeted SCNA recalling) based 10q-terDel calls in cluster 0 (adjusted P = 1.06E-07; one-sided hypergeometric test followed by Bonferroni multiple correction). Dot color shows the significance score (-log10 adjusted P) from Fisher's exact tests, and dot sizes denote the inferred percentage of 10q-terDel bearing cells in each cluster – the latter was computed as the number of 10q-terDel cells divided by all cells in each clusters including cells in which no confident targeted SCNA recalling was made ('NA')). (*** p.adjust<0.01, ** p.adjust <0.05, and * p.adjust<0.1). Over-representation analysis for UCell derived 10q-terDel calls (based on the scNOVA gene set detected in SCb) are shown in the lower panel (adjusted P = 1.74E-26 and 0.013 for cluster 0 and 3, respectively; one-sided hypergeometric test followed by Bonferroni multiple correction). (**e**) Signaling pathways inferred by scNOVA that are significantly over-represented among the marker genes of CITE-seq cluster 0, based on an pathway analysis pursued using ConsensusPathDB[57] (All other significant pathways provided in **Table S15**).

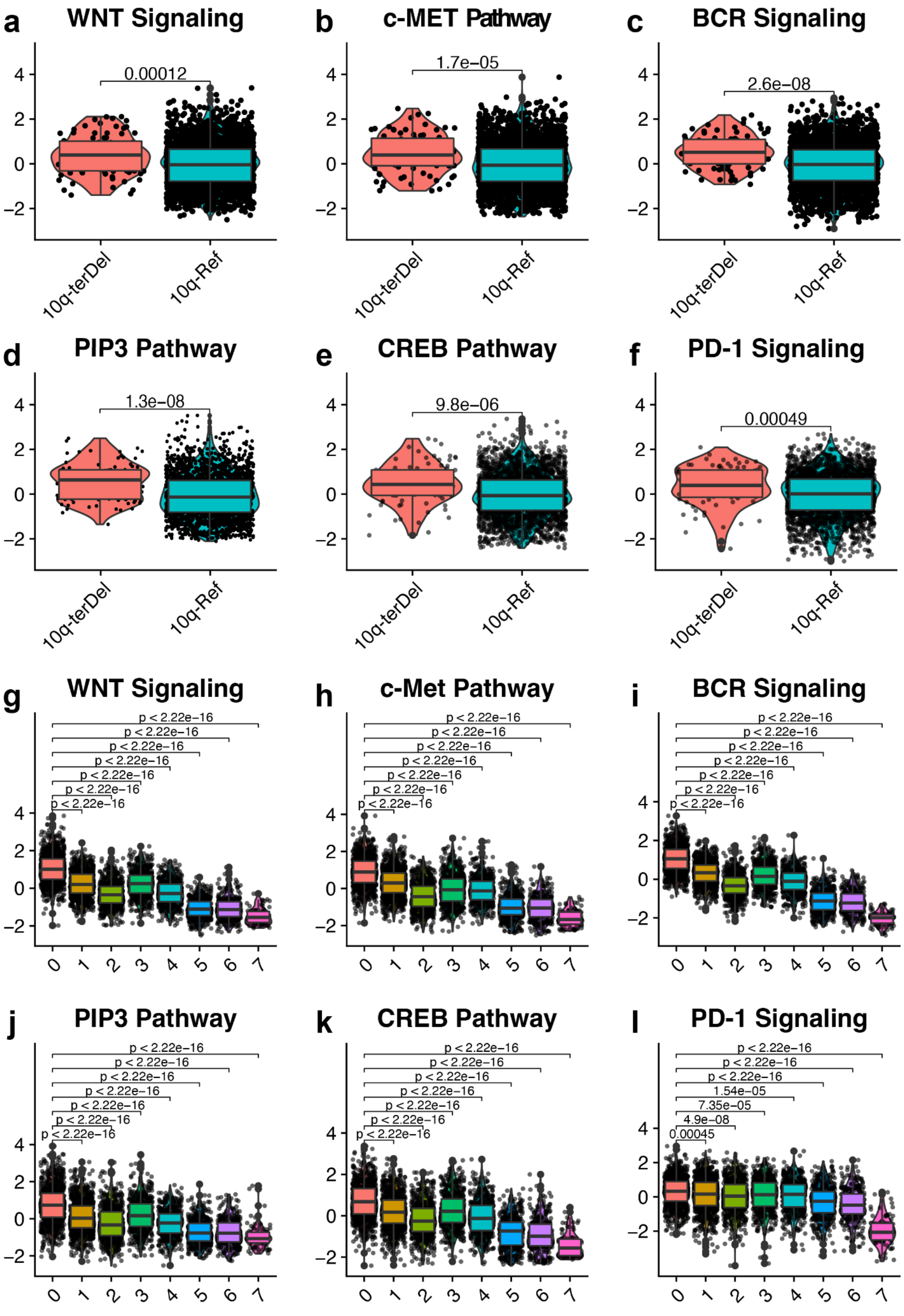

Figure S27. Single-cell gene set enrichment analysis for CLL_24. (**a**)-(**f**) Violin plots of enrichment scores for WNT signaling (KEGG), c-MET pathway (Biocarta), BCR signaling (KEGG) and PIP3 signaling (PID) pathways, comparing 10q-terDel cells to 10q-Ref cells (CONICSmat calls; two-sided Wilcoxon test), and (**g**)-(**l**) Violin plots of enrichment scores for the same pathways, comparing cluster 0 (c0) to all other DoRotheA clusters (FDR-corrected two-sided Wilcoxon rank-sum test). Note that 0-7 in the x-axis denotes DoRotheA cluster 0 to cluster 7. n = 831, 797, 546, 505, 444, 418, 291, and 87 for cluster 0 to cluster 7. Boxplots were defined by minima = 25th percentile - 1.5X interquartile range (IQR), maxima = 75th percentile + 1.5X IQR, center = median, and bounds of box = 25th and 75th percentile.

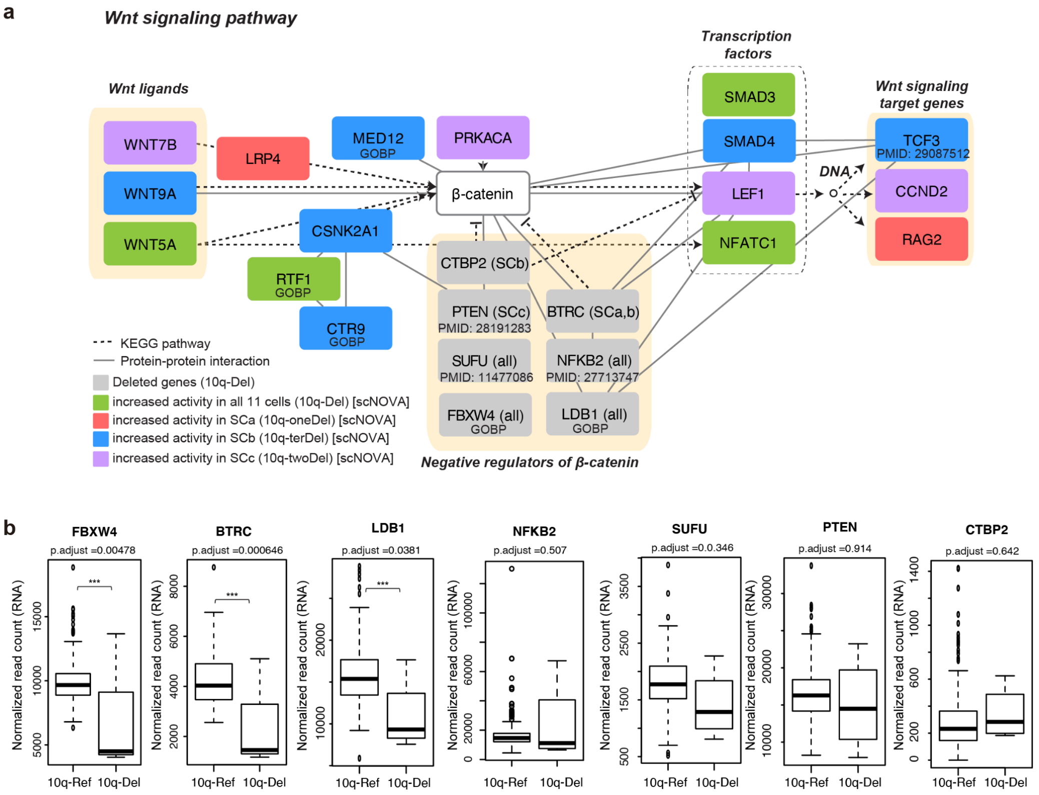

Figure S28. Functional connections between deleted genes in CLL_24 and dysregulated genes inferred by scNOVA. (**a**) Mapping of deleted genes and scNOVA predicted dysregulated genes to the canonical Wnt signaling pathway diagram. Node color denotes the type of alteration (deleted genes in gray, genes with increased activity in all 11 cells with deletion, SCa (oneDel), SCb (terDel) and SCc (twoDel) predicted by scNOVA in green, blue, and in purple, respectively). The principal framework of the diagram is based on the KEGG database, and if the nodes come from other sources, we have given the references under the gene name. Edges between nodes indicate either physical interaction based on the STRING database (solid lines), or pathway information based on KEGG (dashed lines). **(b)** Differential expression of genes of interest in an ICGC CLL cohort[33], in samples bearing the 'minimal region' Del (10q-Del; n = 4 independent CLL samples) compared to donors without Del (10q-Ref; n = 174 independent CLL samples). We analyzed the expression level of known or previously suspected negative regulators of Wnt signaling in the minimal deleted region in 10q. *BTRC* is a known negative regulator of Wnt signaling (**Supplementary Notes**), which is located very close to (only 58kb apart from) the minimal deleted region (deleted in 9/11 single-cells harboring the minimal region Del event). *BTRC* shows significant down-regulation in donors bearing the Del (FDR-adjusted *P*=0.000646), and hence its deletion may have caused or contributed to aberrant Wnt signaling. We also observed significant downregulation of *FBXW4* (FDR-adjusted *P*=0.00478), and *LDB1* (FDR-adjusted *P*=0.0381) deleted in all eleven 10q24.32 SV bearing cells. In the case of *SUFU* (deleted in 11/11 cells with 10q24.32 SV) a slight trend of downregulation (log_2 fold-change = -0.34) did not reach genome-wide significance (FDR-adjusted *P*-value=0.346). For *NFKB2* (deleted in 11/11 cells with 10q24.32 SV), the expression level in the 10q-Ref and 10q-Del samples likewise did not show significant differences (FDR-adjusted P-value=0.507). Adjusted *P*-values were obtained by two-tailed Wald's test from DESeq2[23] followed by FDR adjustment using Benjamini Hochberg correction. Additionally we analyzed the expression level of negative regulators of Wnt signaling in uniquely deleted 10q regions in the SCb (*CTBP2*) and SCc (*PTEN*) subclones; in both cases, the respective genes did not display genome-wide significant differential expression (FDR-adjusted P-value=0.642, 0.914, respectively). Boxplots were defined by minima = 25th percentile - 1.5X interquartile range (IQR), maxima = 75th percentile + 1.5X IQR, center = median, and bounds of box = 25th and 75th percentile.

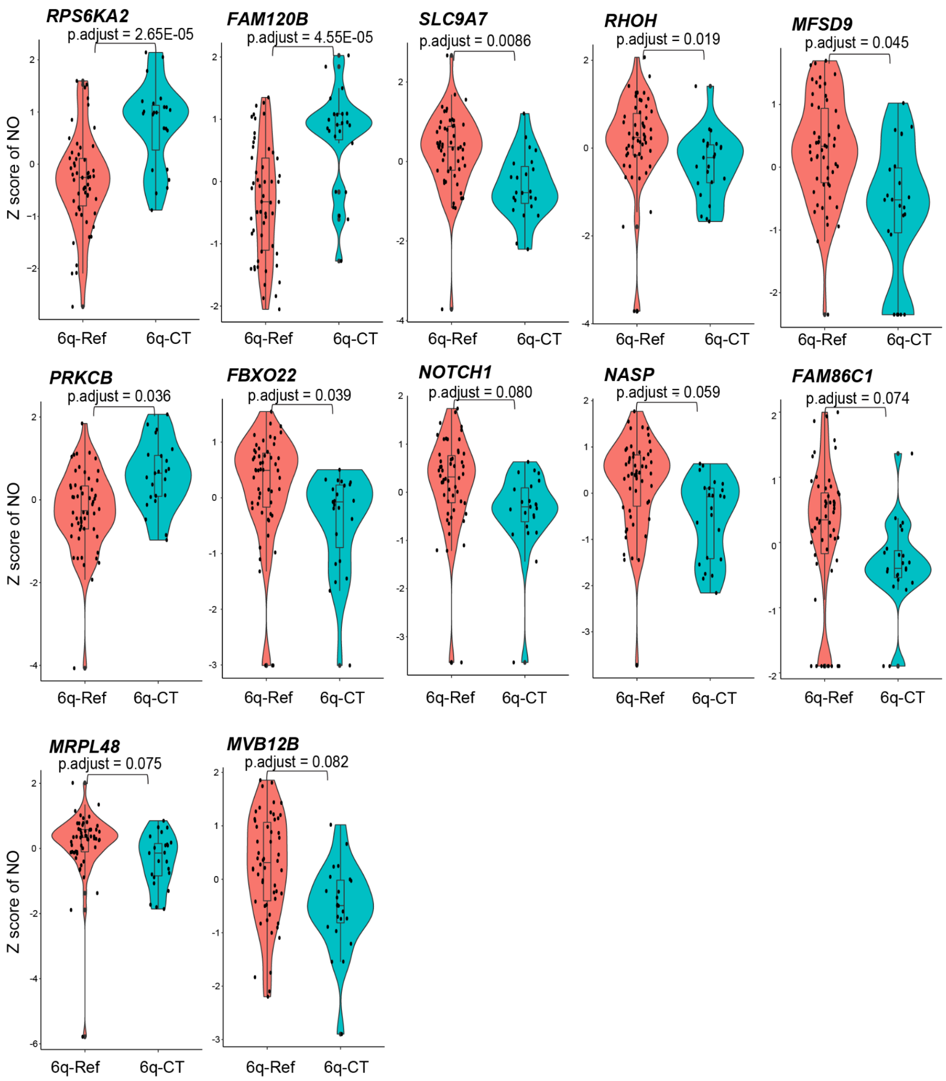

Figure S29. Violin plots of genes showing significant altered activity in the 6q-CT clone compared to the 6q-Ref clone in T-ALL_P1. Twelve genes were identified using a 10% FDR cutoff. The Z score of NO at gene bodies for each single cells in the 6q-CT clone and 6q-Ref clone was depicted using violin plots (two-sided Wald test followed by Benjamini Hochberg multiple correction). This analysis was derived from n = 54 and 23 cells for 6q-Ref, and 6q-CT, respectively. Boxplots were defined by minima = 25th percentile - 1.5X interquartile range (IQR), maxima = 75th percentile + 1.5X IQR, center = median, and bounds of box = 25th and 75th percentile.

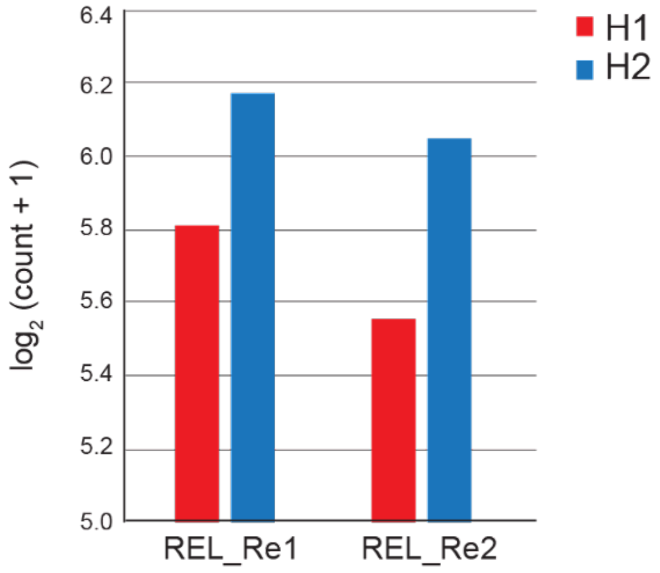

Figure S30. Validation of allelic increase of RNA expression of *MYB* on the chromothripsis haplotype. Increased *MYB* expression on haplotype 2 (H2) of chromosome 6, the chromosomal homolog exhibiting chromothripsis, based on haplotype-resolved RNA-seq pursued with two biological replicates (Re1 and Re2). Bulk RNA-seq data of T-ALL_P1 was analyzed to calculate allele-specific reads overlapping with heterozygous SNP sites. Allele-specific RNA-seq reads were counted using ASEReadCounter[48]. Allelic read counts were assigned to haplotype 1 (H1) or H2 using whole chromosome haplotype-phasing information from StrandPhaseR[49]. Allelic read counts along the gene were aggregated to retrieve haplotype-resolved gene-level read counts. *MYB* expression on H2 was 1.4-fold increased over H1 (P = 0.0317; likelihood ratio test, provided by EdgeR[11]).

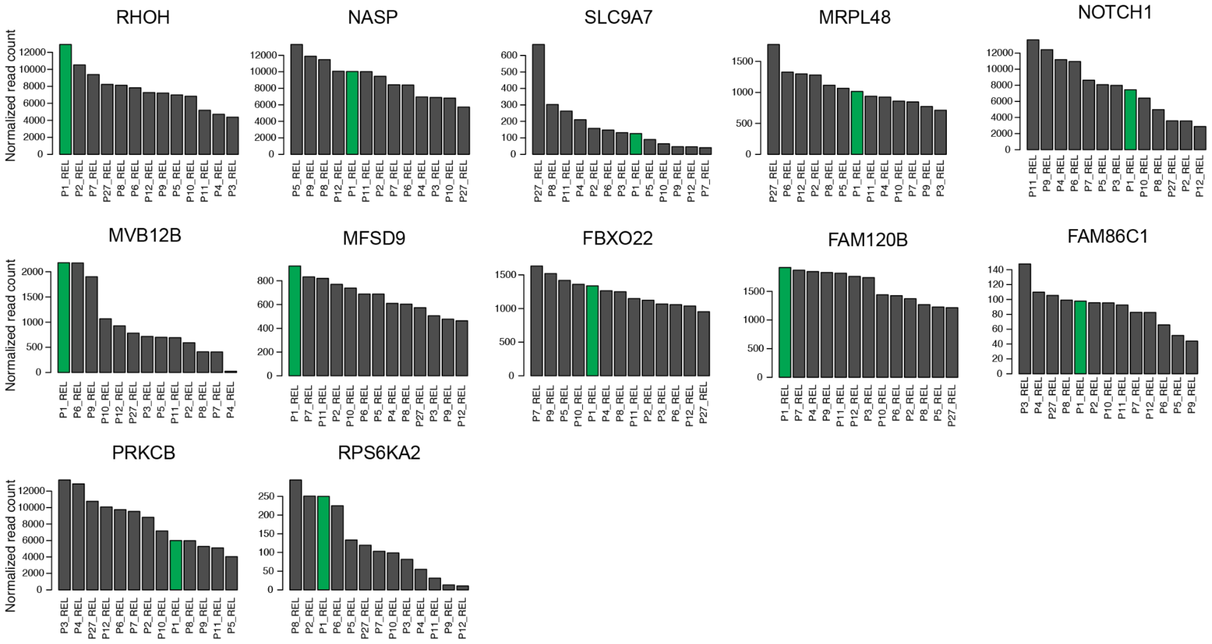

Figure S31. Expression level of genes predicted by scNOVA to be altered in activity in the SV subclone in T-ALL_P1. Bulk-cell RNA-seq based gene expression measurements are shown for all genes in **Fig. 5b**, which scNOVA inferred to change in activity as a consequence of chromothripsis, in a panel of 13 T-ALL derived samples. The index sample, T-ALL_P1 (P1), is highlighted in green.

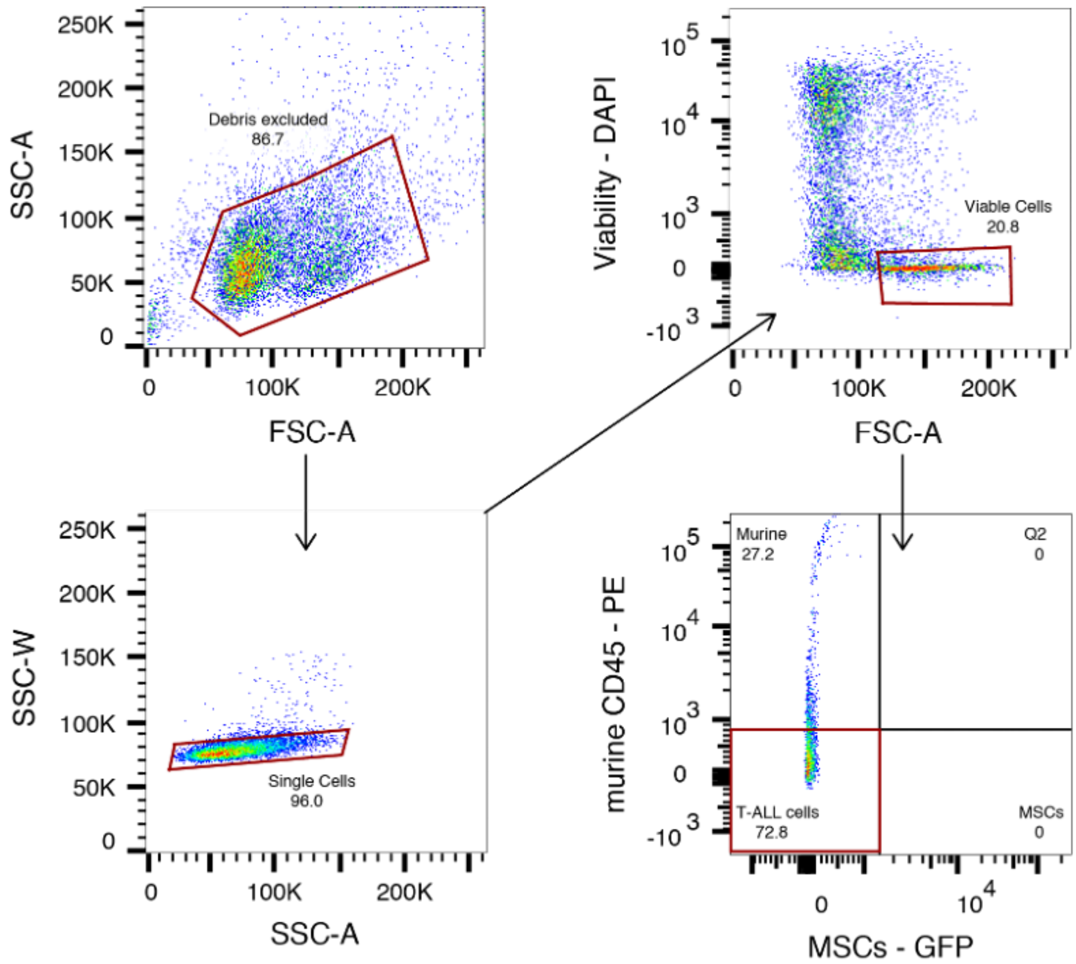

Figure S32. Gating strategy for single, viable T-ALL cell isolation from T-ALL sample T-ALL_P1 for scRNA-seq. Viable cells are identified by low staining with DAPI, a viable cell impermeable nuclear stain. Human T-ALL cells are selectively sorted from contaminating murine and feeder layer cells by their lack of murine CD45 and GFP expression. Red gate shows selected population which is visualized in consequent plot, indicated by arrow. The final red gate indicates the population which was sorted.

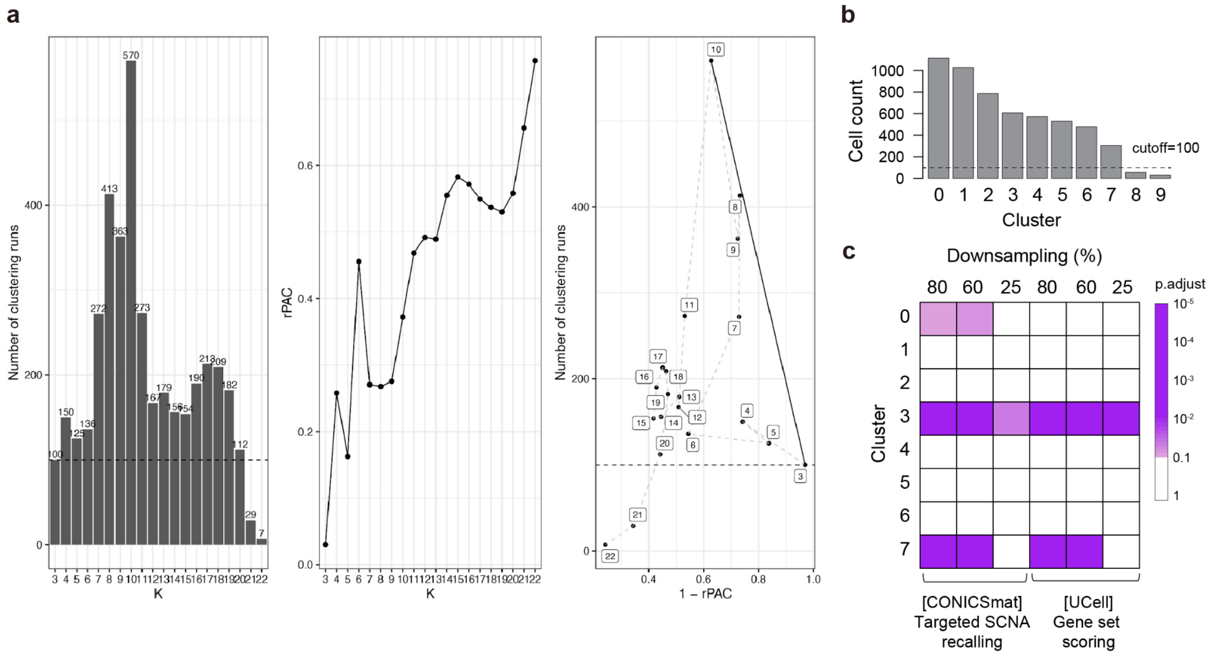

Figure S33. Evaluation of the robustness for the unsupervised clustering in T-ALL_P1 scRNA-seq. (a) To test the suitability of our chosen clustering parameter we used MultiK package[58]. MultiK identifies the optimal number(s) of clusters for a given single scRNA-seq experiment by testing resolution parameters between 0.05 and 2.0 in steps of 0.05. The data is randomly subsampled to 80% each run, and the resulting output is a summary of how often a given number of clusters was determined, regardless of the chosen resolution parameter. Our chosen resolution parameter gives rise to the optimal number of clusters (k = 10). (**b**) Bar graph showing the cell count assigned to each of the clusters. Amongst 10 clusters, 8 clusters which contain more than 100 single cells were subjected to the downstream analysis. (**c**) Downsampling analysis to test the enrichment for the 6q-CT subclone in transcriptome based unsupervised clusters. Downsampling was carried out by randomly subsetting the data to 80%, 60%, and 25% of single cells from the total data set, and repeating the enrichment analysis of CONICSmat and UCell-annotated 6q-CT cells per cluster. In each case and in both annotations, cluster 3 remained significant after multiple correction (FDR 10%); notably, cluster 3 in all cases reached the highest significance. Furthermore, cluster 3 was the only cluster remaining significant when downsampling/subsetting to 25% was performed.

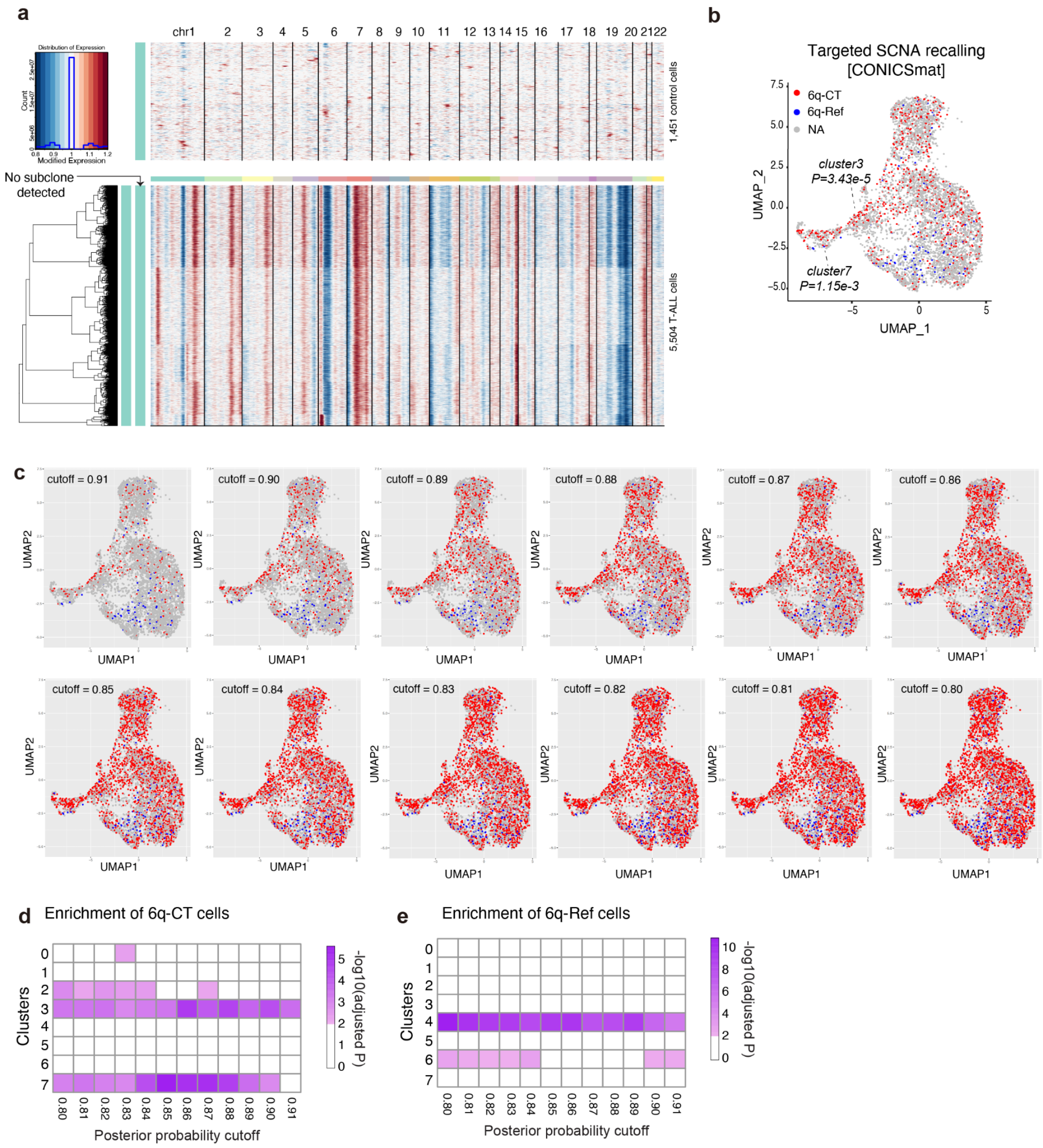

Figure S34. Exploration of posterior probability cutoffs for CONICSmat for the inference of SVs in T-ALL_P1 using scRNA-seq. (**a**) InferCNV[50] analysis of 5,504 high quality T-ALL_P1 cells, and 1,451 control cells. Control cells were downloaded from PBMC data provided by 10X Genomics. This analysis did not discover subclones in 5,504 T-ALL cells. (**b**) Single-cells harboring deletions in chromosome 6 were inferred using CONICSmat 'targeted SCNA recalling mode' by applying posterior probability cutoff 0.9. CONICSmat confidently performed targeted SCNA recalling for 15% of the scRNA-seq dataset for which 729 cells were predicted to harbor the chromothripsis rearrangement ('6q-CT'), and 109 cells were called '6q-Ref' across the entire chromosome 6 (for the remainder of 4,666 cells no confident assignment could be made). Confident 6q-CT cells are enriched in the cluster 3 and cluster 7 cells. Based on this result, we calculated an estimated range of CFs, 1) If we include uncallable cells to the calculation, chromothripsis is detected in 729 cells among 5,504 cells (estimated CF=13%). 2) If we exclude uncallable cells, the estimated CF of 6q-CT is 87%. The Strand-seq based CF estimate (30%) is well within this range. (**c**) 6q-CT calls were made by applying a range of posterior probability cutoffs, and projected to the UMAP. As the maximum posterior probability was 0.9133, we tested the cutoff range from 0.80 to 0.91, by increasing 0.1 each time. (**d-e**) Enrichment of confident 6q-CT calls (**d**) and confident 6q-Ref calls (**e**) for each of the unsupervised clusters were tested using fisher's exact test followed by Benjamini-Hochberg multiple correction. It shows that cluster 3 and cluster 7 are robustly enriched by confident 6q-CT calls, and the cluster 4 and cluster 6 are robustly enriched by confident 6q-Ref calls.

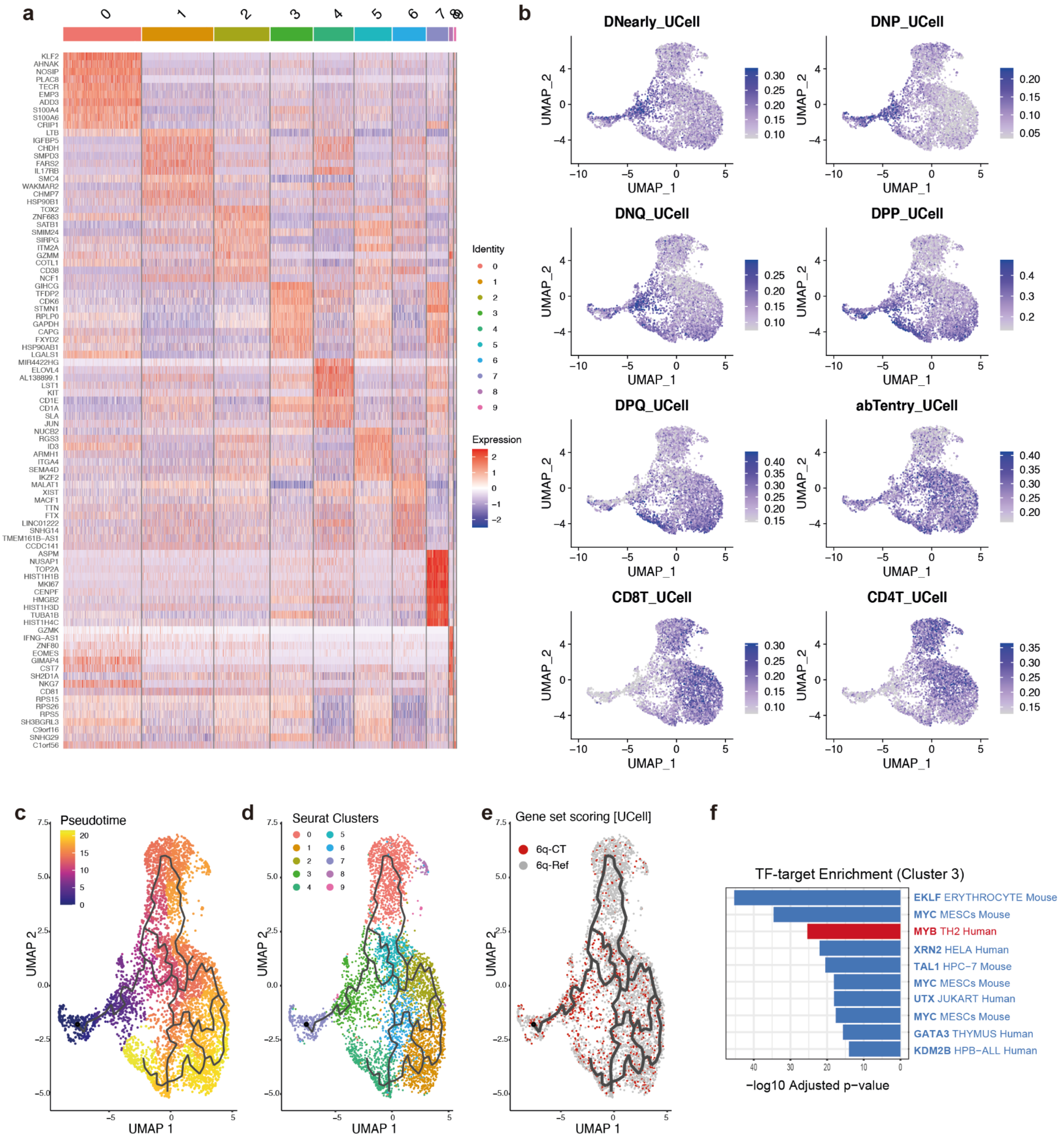

Figure S35. Lineage trajectory analysis of scRNA-seq of T-ALL_P1. (**a**) Marker genes for the ten unsupervised clusters identified from scRNA-seq of T-ALL_P1 (10X Genomics). (**b**) UCell scores for each of eight T-cell cell-types were projected to the UMAP of scRNA-seq of T-ALL_P1. T-cell cell-type marker genes were downloaded from the previous publication[51]. (**c-e**) Pseudotime trajectory analysis across clusters and subclone assignments for the T-ALL_P1 scRNA-seq data. (**f**) Bar graph showing *P*-values for the top 10 significant TFs from the TF-target over-representation analysis of differentially expressed genes for cluster 3. Red color denotes *MYB*, blue color denotes TFs which interact with/are transactivated by *MYB*. (For the corresponding *P*-values in cluster 7, where *MYB* was also significant, see **Table S10**). This analysis shows that differentially expressed genes for both cluster 3 and 7 are significantly enriched for *MYB* target genes (adjusted *P*=4.25e-26, and *P*=1.88e-26, respectively).

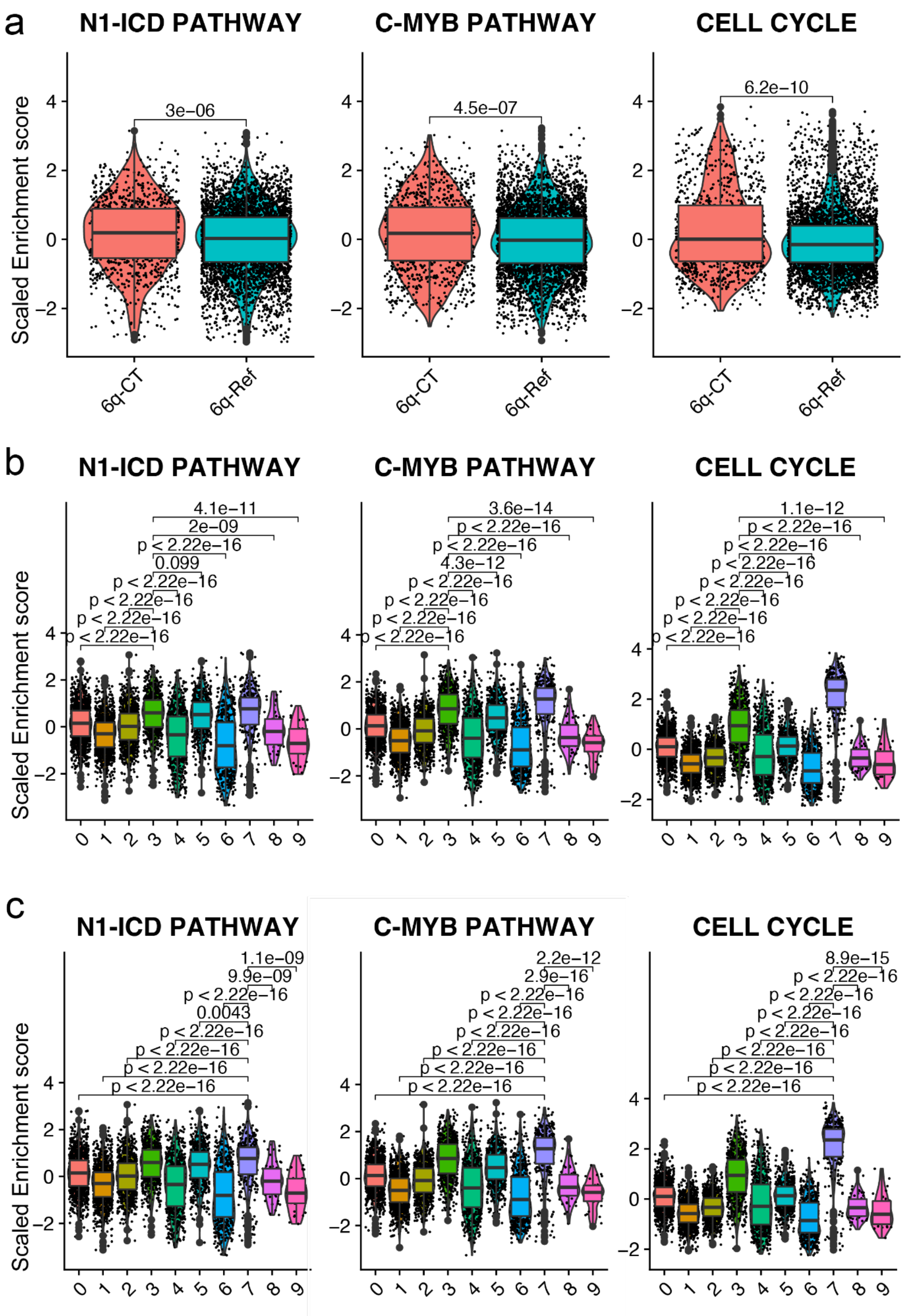

Figure S36. Single-cell gene set enrichment analysis for T-ALL_P1 scRNA-seq data. Violin plots of enrichment scores for *Notch1* intracellular domain (N1-ICD) Pathway (M611; Reactome) (left), c-MYB Pathway (M195; PID) (middle) and Cell Cycle (M7963; KEGG) (right), with pairwise comparisons (two-sided t-test) indicating significant differences between (**a**) UCell-based 6q-CT and 6q-Ref cells (n = 970, and 4,447 cells, respectively), (**b**) cluster 3 (c3) vs all other SCT-derived clusters and (**c**) cluster 7 (c7) vs all other SCT-derived clusters (n = 1,114, 1,026, 786, 607, 573, 529, 478, 304, 57, and 30 for cluster 0 to cluster 9 cells, respectively); 0-9 in the x-axis denotes SCT-derived cluster 0 to cluster 9. In this analysis, both clusters 3 and 7 show significantly higher enrichment scores for the c-Myb transcription factor network compared to all other clusters. Boxplots were defined by minima = 25th percentile - 1.5X interquartile range (IQR), maxima = 75th percentile + 1.5X IQR, center = median, and bounds of box = 25th and 75th percentile.

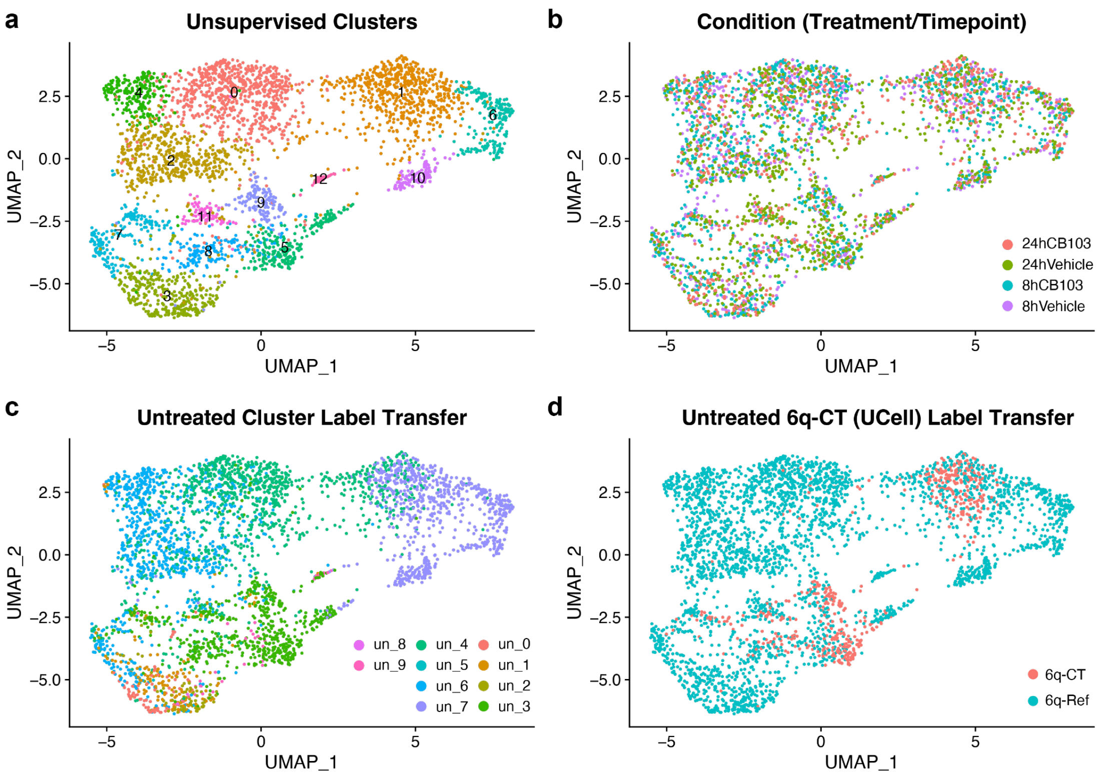

Figure S37. Unsupervised clustering and reference-based label transfer for T-ALL_P1 scRNA-seq experiments following drug treatment. While *MYB* itself is an attractive target in T-ALL_P1 (and leukemias in general[61]), c-Myb-targeting therapies have shown limited efficacy/specificity[62,63]. Instead, from the c-Myb targets inferred by scNOVA, we selected *NOTCH1* as a suitable candidate because *i*) it is inferred by scNOVA to be highly upregulated in 6q-CT (**Fig. 5b**) and *ii*) it has been shown to be readily targetable by a variety of compounds and strategies[64]. One such inhibitor of NOTCH signaling is CB-103, a pan-NOTCH small-molecule inhibitor which targets the *Notch1* intracellular domain (N1-ICD) and is currently in phase 2 clinical trials[65,66]. We treated T-ALL_P1 cell cultures with the CB-103 pan-NOTCH small-molecule inhibitor (targeting the *Notch1* intracellular domain (N1-ICD)[65,66]) or a vehicle control for 8h and 24h (**Methods**), and used scRNA-seq to analyze the differential drug response. (**a**) UMAP showing unsupervised clusters (k = 12) identified in scRNA-seq from integrating all four conditions (24h CB-103, 24h Vehicle, 8h CB-103, 8h Vehicle) from the T-ALL_P1 drug treatment experiment. Note that cluster IDs depicted were newly generated in this analysis, and thus do not correspond to the cluster IDs generated from the data shown in **Fig. 6a** (see **main text**). (**b**) UMAP from (a) overlaid with sample identity of the 4 integrated drug treatment samples. (**c**) Reference-based label transfer of unsupervised cluster labels identified in untreated data, from **Fig. 6a**. (**d**) UMAP from (a) overlaid with reference-based label transfer of cell labels (identified using UCell) from untreated data. Red dots correspond to cells assigned to bear the chromothripsis event; these cells are significantly over-represented in the original cluster 3 (here denoted un_3) and 7 (here denoted un_7) in panel (c) (FDR adjusted P=8.83e-10 and P=4.2e-6 for un_3 and un_7, respectively; Fisher's exact test).

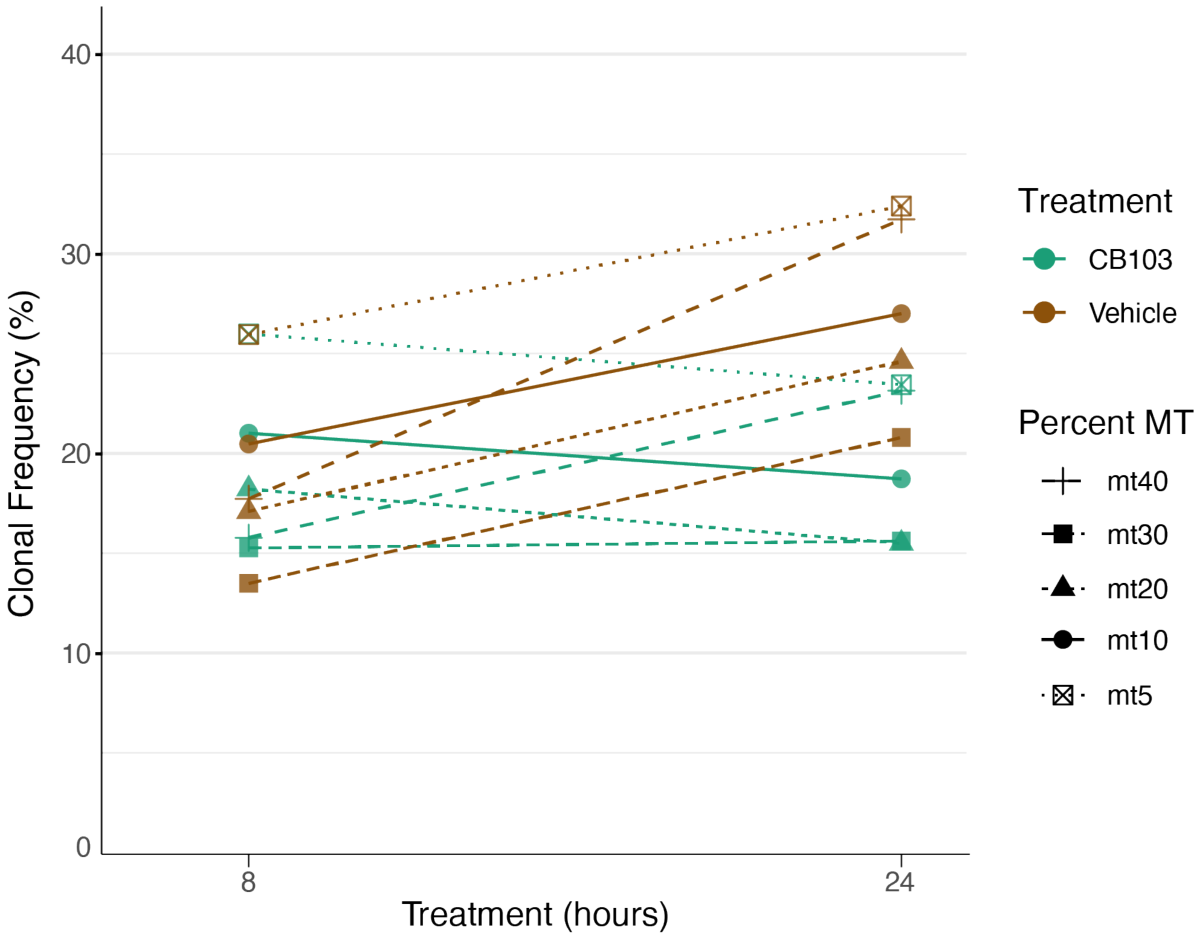

Figure S38. Detailed viability analysis for T-ALL_P1 drug treatment scRNA-seq. Lineplot depicting changes in CF of 6q-CT subclone between 8h and 24h in CB-103 and vehicle treated cells, at various viability (i.e. percent MT) cutoffs.

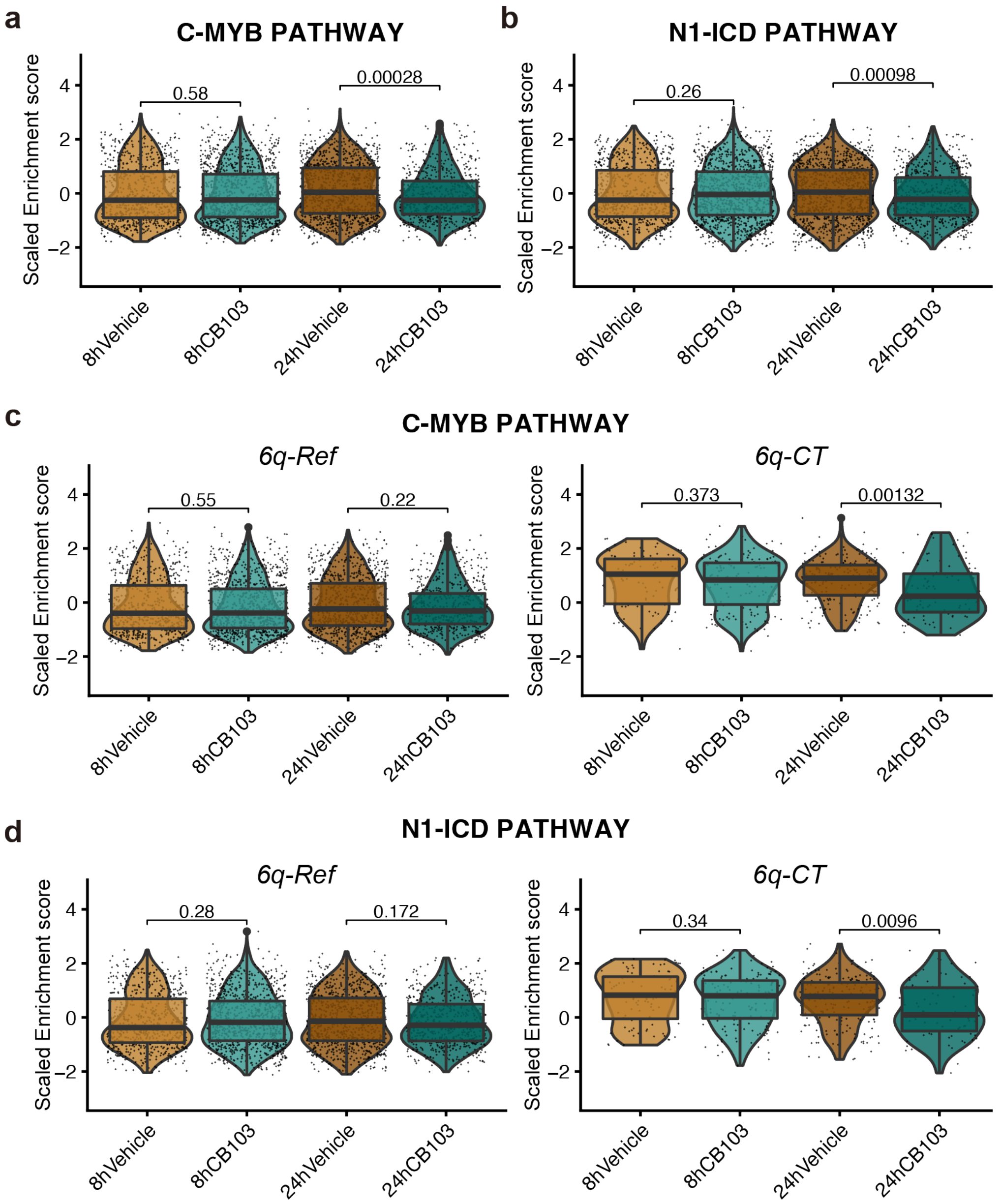

Figure S39. Single-cell gene set enrichment analysis for MSigDB pathways across conditions for scRNA-seq of T-ALL_P1 drug treatment data. Scaled enrichment score per treatment condition for (**a**) c-MYB Pathway (M195; PID) and (**b**) N1-ICD Pathway (M611; Reactome). Significant difference in scaled enrichment score between paired conditions is indicated on the plot (FDR-corrected two-sided Wilcoxon rank-sum test). (**c-d**) Scaled enrichment score from (a-b) split by 6q-status (left: 6q-Ref, right: 6q-CT). Significant difference in scaled enrichment score between paired conditions per 6q-status is indicated on the plot (FDR-corrected two-sided Wilcoxon rank-sum test; Boxplots were defined by minima = 25th percentile - 1.5X interquartile range (IQR), maxima = 75th percentile + 1.5X IQR, center = median, and bounds of box = 25th and 75th percentile.). This analysis demonstrated specific depletion of the REACTOME N1-ICD gene set in CB-103-treated 6q-CT cells after 24h, consistent with specific subclone-targeting (P=0.0096; FDR-adjusted Wilcoxon-rank sum test). We did not detect any significant effects on N1-ICD after 8h – in line with a prior report showing that N1-ICD protein levels are significantly reduced after 24h of treatment with CB-103, whereas they are unaffected following shorter-term treatment[66].

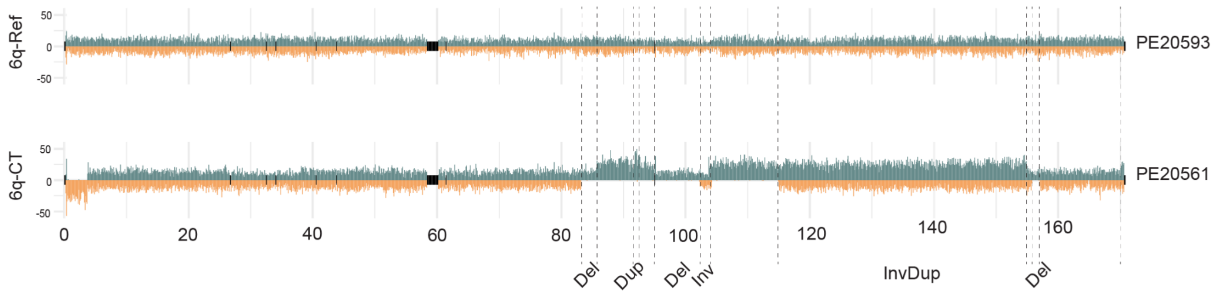

Figure S40. Verification of subclonal chromothripsis in the secondary xenograft of T-ALL_P1. In this study, we generated a new PDX of the T-ALL_P1 patient sample for the purpose of a drug treatment experiment (**Methods**). Strand-seq of the new PDX of T-ALL_P1 confirmed the presence of a subclonal chromothripsis event in chromosome 6, which is the same event previously seen in the 1st PDX model[1]. This secondary PDX was used for the drug treatment experiment shown in **Fig. 6e-g.**

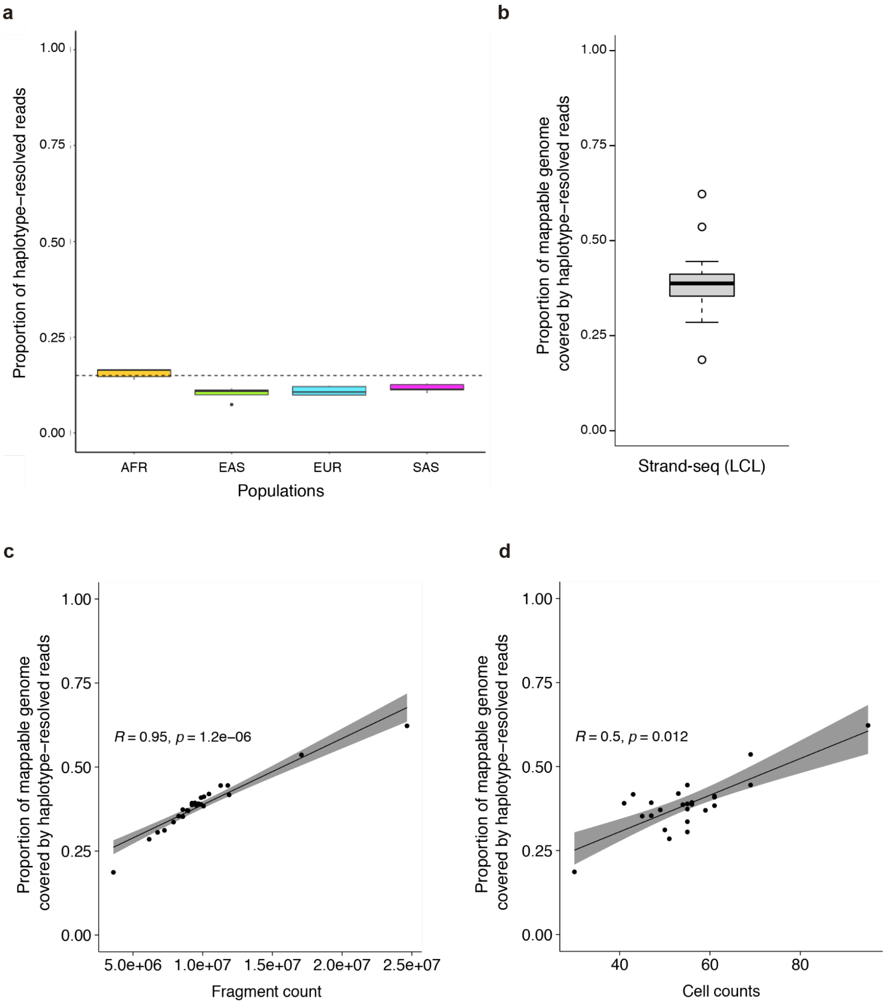

Figure S41. Comparison of the Strand-seq to conventional WGS to assess the proportion of the genome that can be accessible for allele specific analysis. (**a**) Proportion of Illumina WGS reads that can be haplotype-phased, and thus are accessible for 'classical' allele-specific analyses, in the 1KG haplotype reference panel [52]. We randomly picked five 1KG samples, sequenced by Illumina short read based WGS, from each continental 'super-population' (AFR: African ancestry, EAS: East Asian ancestry, EUR: European ancestry, SAS: South Asian ancestry; n = 5 independent samples for each continental population; Boxplots were defined by minima = 25th percentile - 1.5X interquartile range (IQR), maxima = 75th percentile + 1.5X IQR, center = median, and bounds of box = 25th and 75th percentile.) and assigned reads to haplotypes using phased heterozygous sequence variants. The horizontal dashed line marks 15% of the haplotype-resolved reads, which is the approximate upper bound for the fraction of reads that can be assigned to a haplotype to pursue classical allele-specific analyses. This is in agreement with previous studies that have typically ascertained only up to 15% of the genome using short read data[53]. This fraction is lower for samples from EUR, SAS and EAS populations, as expected, due to the smaller number of heterozygous SNPs compared to AFR populations. (**b**) Proportion of 1KG mappable regions that are haplotype-resolved by Strand-seq, using 25 LCLs (including 24 from a human diversity SV panel[31], as well as NA12878[2]). Single-cell libraries from chromosomes showing either a WC (Watson/Crick) or a CW configuration were haplotype-resolved using StrandPhaseR[49] and pooled into phased pseudo-bulk data sets (n = 25 biologically independent LCL samples; Boxplots were defined by minima = 25th percentile - 1.5X interquartile range (IQR), maxima = 75th percentile + 1.5X IQR, center = median, and bounds of box = 25th and 75th percentile.). Evaluations were restricted to the autosomes. (**c**) Proportion of 1KG mappable regions that are haplotype-resolved using Strand-seq, plotted by the fragment count achieved for each LCL sample. (**d**) Proportion of 1KG mappable regions haplotype-resolved by Strand-seq, plotted by the number of cells sequenced for each LCL sample. Regression line and 95% confidence intervals shown as a gray band in the plot (**c-d**). P-value was calculated based on the Spearman correlation test for (**c-d**). The data in (**b**), (**c**), and (**d**) suggest that Strand-seq has the ability to access up to the entire mappable genome, *e.g.*, to allow haplotype-specific NO analyses from

telomere to telomere. The actual fraction of genomic nucleotide bases accessed depends on the number of cells sequenced by Strand-seq, as well as on the sequencing depth (fragment count). It also depends on the specifics of nucleosome positioning, as outlined in **Fig. 1** in the main text, where the MNase digestion step used during Strand-seq library preparation directs sequencing to DNA regions protected by nucleosomes.

Supplementary Tables

Table S1. Characterization of nucleosomal fragments in Strand-seq libraries
(Table accompanying the submission as a spreadsheet)

Table S2. List of genes identified to be changing in activity when comparing major and minor clones with scNOVA. *(The Supplementary Material provides further details on some of the genes contained.)*
(Table accompanying the submission as spreadsheet)

Table S3. List of somatic SVs identified in single cells
(Table accompanying the submission as spreadsheet)

Table S4. Summary of inferring SCNAs from the scRNA-seq data sets
(Table accompanying the submission as spreadsheet)

Table S5. Reference list of ATAC-seq peaks from prior literature used to define putative CREs in AML_1
(Table accompanying the submission as spreadsheet)

Table S6. Reference list showing literature sources for sets of TF target genes, defined by TF binding (ChIP-seq) and RNA perturbation (RNA-seq or microarray) upon silencing of TFs
(Table accompanying the submission as spreadsheet)

Table S7. Gene sets used in expression analyses to verify TF target gene and pathway activities
(Table accompanying the submission as spreadsheet)

Table S8. Cell-wise genotypes and correlation matrix of NA12878 deletions on chr19 and chr22 determined using ArbiGent
(Table accompanying the submission as spreadsheet)

Table S9. List of differentially expressed genes identified for the eight unsupervised clusters in the T-ALL_P1 scRNA-seq data
(Table accompanying the submission as spreadsheet)

Table S10. Top 20 significant TFs for Cluster 3 and Cluster 7 of T-ALL_P1 scRNA-seq data identified using EnrichR analysis
(Table accompanying the submission as spreadsheet)

Table S11. Haplotype-specific CREs detected from T-ALL_P1, RPE-1, and BM510
(Table accompanying the submission as spreadsheet)

Table S12. Comparison of scNOVA with other single-cell multi-omics methods
(Table accompanying the submission as spreadsheet)

Table S13. Benchmarking analysis of scNOVA's differential gene activity analysis for different cell fractions (CF).
(Table accompanying the submission as spreadsheet)

Table S14. List of oligonucleotide-conjugated antibodies used for CITE-seq.
(Table accompanying the submission as spreadsheet)

Table S15. List of over-represented pathways of cluster 0 marker genes identified in the CITE-seq of CLL_24.
(Table accompanying the submission as spreadsheet)

Table S16. List of over-represented pathways of differentially active TFs of terDel cells identified in the CITE-seq of CLL_24.
(Table accompanying the submission as spreadsheet)

Table S17. The primer sequences for quantitative real time PCR (qPCR).
(Table accompanying the submission as spreadsheet)

Supplementary Notes for Methodological Details

1. Estimating genome-wide coverage

NA12878 Strand-seq data aligned to the hg38 reference assembly was downloaded[2] and sequence reads with low quality (MAPQ<10), supplementary reads, and duplicated reads removed. Coverage in each single cell was estimated as previously described[1].

2. Analysis and comparison of NO profiles derived from Strand-seq and MNase-seq

Raw reads from a previously published NA12878 MNase-seq experiment (single end, scqual and scfasta) were obtained from ENCODE (ENCSR000CXP). These MNase-seq data were generated using a SOLiD sequencer, and the data are therefore in color-space DNA sequence read format. We aligned these reads to the hg38 reference genome with bowtie (v.1.1.2)[67], using color-space read mapping enabled. After alignment, sequencing reads with poor quality (MAPQ<10), supplementary reads, and duplicated reads were removed.

```
bowtie --threads 4 -C -Q Input.csqual -f -S genome_hg38_CS Input.csfasta > output.sam
```

To obtain nucleosome positions and read depth signals (shown in **Fig. 1**, for example), the 'dpos' function provided by the DANPOS package[68] was applied to the aligned Strand-seq data, as well as the NA12878 MNase-seq data – to generate nucleosome midpoint positions genome-wide and wig files for browser track visualization with 10bp genomic bins. For the Strand-seq data, the paired-end=1

parameter was used. The Strand-seq track was haplotype-resolved into H1 and H2 tracks by pooling NO profiles generated from reads haplotype resolved using StrandPhaseR[49].

To compare nucleosomal positions obtained from pooled Strand-seq NO profiles and MNase-seq, genomic positions of human reference enhancer elements based on DNase-seq (DNase I hypersensitive sites [DHS] sequencing) and ChromHMM state analysis were downloaded [8], lifted over to hg38, and extended by 2kb centered at the midpoint. Correlation between NO in the enhancer elements based on pooled Strand-seq and MNase-seq was determined using Spearman's rho (0.68). We note that similar correlation coefficients were recently reported in a comparison of MNase-seq vs. scMNase-seq[15] – which corroborates the high quality of Strand-seq-derived NO profiles. Enhancer elements based on DHSs were defined using the Roadmap Epigenomics Consortium resource – comprising a union of 127 epigenomes in total – using data from the following URL:

https://personal.broadinstitute.org/meuleman/reg2map/HoneyBadger2_release/.

3. Visualization of NO at gene bodies for genes stratified by their expression level

To visualize NO at gene bodies, each gene locus was extended by 5kb upstream of TSS and 5kb downstream of TTS. Read coverage at single base resolution was calculated on these extended loci. As each gene has different length, the coverage was normalized by fitting the coverage vector to a spline and then sampling 101 points at equal intervals. Genes were grouped into five sets based on their RNA expression level (FPKM=0, FPKM 0~0.1, FPKM 0.1~1, FPKM 1~3, FPKM>3) and their average normalized coverage was plotted as line graphs (**Fig. 1g**).

4. Analysis of previously reported scMNase-seq data

Previously published single-cell MNase-seq (scMNase-seq) data from mouse cells (NIH3T3 cell line and murine naive T cells)[15] were downloaded (GSE96688) in fastq format. These raw data were aligned to the mouse reference genome (mm10) using bwa[69]. Sequencing reads with low quality (MAPQ<10), supplementary reads, and duplicated reads were removed. The mono-nucleosomal fraction was extracted (140-180bp) using samtools[70] and shell script with the following parameters.

```
samtools view -h alignment.bam | \
  awk 'substr($0,1,1)=="@" || ($9>= 140 && $9<=180) || ($9<=-140 && $9>=-180)' | \
  samtools view -b > alignment_mono.bam
```

After pre-processing, NO signals in the gene-bodies were analyzed using scNOVA (in a haplotype-unaware manner) to pursue cell type classification (**Extended Data Fig. 3**) and to correlate NO with gene expression (**Fig. S4**). The method potentially has extended applicability to other single-cell data sets such as scMNase-seq data. In the future, one could consider also integrating ATAC-seq into the Strand-seq assay, potentially enabling enhanced analysis of chromatin accessibility; however, this would come at the price of a lower resolution of SV calls, given the less uniform coverage of transposase-mediated compared to MNase-mediated cuts[71].

5. Cell type classification

To train the supervised cell type classifier based on gene body NO, we used 179 single-cell Strand-seq libraries generated from two diploid LCLs (50 cells from HG02018, 50 cells from NA19036), as well as from a replicate of the near-diploid RPE-1 cell line (79 cells; 'replicate 1' [26]). 19,629 ENSEMBL genes with at least one read detected at the respective gene bodies were considered as initial input feature sets. An X matrix [179 cells-by-features] and a Y matrix [179 cells-by-two cell type] were prepared for PLS-DA, in order to find latent variables which can explain the variability in Y using linear combinations of features in the X matrix. Using three latent variables which explain 98.48% of the variance of the Y variable, variable importance of projection (VIP) for each feature was calculated. Highly informative features (VIP>90% of null distribution) were selected to build the final classifier.

Classification performance of the final classifier was evaluated by leave-one-out cross validation using the 179 cells, which yielded 100% accuracy of classification (are under the curve (AUC)=1) with six latent variables. Finally, we applied this model to an independent validation using a different LCL (46 cells from HG01573) and the same RPE-1 epithelial cell line, albeit using a different replicate (77 cells; 'replicate 2'), verifying 100% classification accuracy with independently generated data (AUC=1). To generate the UMAP shown in **Extended Data Fig. 3**, 302 cells including those from the training set (179 cells) and the independent validation set (123 cells) were projected onto the final classification model. The resulting prediction score of six latent variables for each cell was used to perform UMAP[116] for dimensionality reduction.

6. Haplotype-resolved SV discovery in single cells

The scNOVA computational framework utilizes the previously described scTRIP method for haplotype-aware SV discovery of the full spectrum of somatic SVs ≥200kb in size in Strand-seq data, by executing the MosaiCatcher computational pipeline[26]. In brief, this pipeline integrates three 'channels' – template strand, read depth and haplotype-phase – to discover deletions, duplications, balanced inversions, inverted duplications, balanced translocations, unbalanced translocations and complex SV including BFBs and chromothripsis events, and it maps these SVs to defined chromosomal homologs or derivative chromosomes (which may include extrachromosomal DNA). All single cells are subjected to SV discovery, regardless of chromosomal template strand configuration[26] (such as Watson/Crick (WC), Crick/Crick (CC), or Watson/Watson (WW)), and joint modeling of the data is pursued which increases the detection sensitivity for SVs present in more than one single cell[26]. By default, scNOVA employs the 'strict' scTRIP SV caller, which has been optimized for detecting SVs with CF≥5% [26]. SV discovery can be bypassed in the scNOVA framework, to focus downstream functional investigation to user-defined somatic SVs.

7. CNN model to infer expressed genes based on NO

Both phased and unphased single-cell reads were used to generate NO profiles. Feature sets were incorporated into one-dimensional CNNs. To define the feature sets for each gene, we considered genomic regions spanning the body of genes, which we – for the purpose of the CNN – expanded from 5kb upstream of the TSS until 5kb downstream of the TTS, to include 5kb of flanking non-transcribed sequences on each flank which appeared informative as well (**Fig. 1f**). Each gene was divided into 150 bins, whereby we considered genomic annotations of the start and end coordinates of genes provided via ENSEMBL's GTF file, as follows: 50 bins for the region -5kb to the TSS, 50 bins for the gene body, and 50 bins for the region from the TTS to +5kb. Five layers of feature sets were derived for those 150 bins: NO, single cell variance of NO, GC content, CpG content, and replication timing, which we included in the CNNs to assist bin stratification. To compute NO for *Step 1*, the read depth within each of the 150 bins was first normalized by bin length using smooth spline fitting in R, and then normalized by library size to obtain read per million (RPM) measurements, which subsequently were scaled by the locus copy number status. We also implemented the alternative option to pursue copy number normalization before normalization by library size, which we recommend to use when applying scNOVA with copy number unstable cancer types. To compute the single cell variance of NO, coefficients of variation (CV = standard deviation / mean) for the single-cell read depth were calculated for each bin. Systematic effects of mean on the CV were regressed out using smooth spline fitting. GC and CpG content were computed based on the nucleotide content of each bin, using the Homer annotate peak tool[120]. For replication timing, we used a pre-processed signal track (hg19) from the UCSC Genome Browser database[121] (UW Repli-seq track), which we mapped to the hg38 genome using the UCSC LiftOver tool. scNOVA can also consider CNNs for inferring expression in a single cell, which

use four rather than five layers of feature sets (excluding single-cell variance of NO, which cannot be computed for a single cell).

To define ground-truth labels of not expressed genes (NEs) and expressed genes (EGs), we used bulk RNA-seq data from three RPE cell lines (RPE-1, BM510, and C7)[26]. Reads were aligned onto hg38 with STAR aligner (v2.5.3)[122], using gene annotations from ENSEMBL GTF (GRCh38.81). FPKM values were obtained with Alfred[123]; genes with FPKM>1 were labeled as EGs, and all remaining as NEs. We used the following numbers of EGs and NEs for training: RPE-1: 10413 EGs, 9131 NEs; BM510: 10339 EGs, 9205 NEs; C7: 10486 EGs, 9058 NEs. We used the hyperopt package[124] to search for optimal hyperparameters for the CNNs (**Fig. S5**).

In leave-one-chromosome-out cross-validation[119] experiments (where we trained a model leaving out a certain chromosome, and then applied the model to the chromosome previously left out), the CNNs outperformed random forest and SVM based models with the same set of features (**Fig. S6**). To assess model performance for different number of aggregated cells (clones of different sizes), we pooled Strand-seq data to generate randomized pseudo-bulk datasets for 80, 40, 20, 5, and 1 cell (s), respectively, and evaluated CNN performance using leave-one-chromosome out cross-validation. Trained models for each chromosome, and for different clone set sizes, are made available along with the code of scNOVA to facilitate application to new data sets.

8. Identifying optimal parameters for inferring changes in gene activity using NO

We parameterized, and examined the performance for, inferring differentially expressed genes (DEGs) using various RPE cell lines and LCLs (see main text). The initial parameterization of scNOVA was done using RPE-1 and HG01573 (LCL): To define the ground truth set of DEGs, we compared bulk-cell RNA-seq data from RPE-1 cell line versus HG01573 using DESeq2. For scNOVA analysis, we treated the 156 single-cell libraries from RPE-1 cell line as a single "pseudo-clone", and the 46 single-cell libraries from HG01573 as a second "pseudo-clone". The CNN was trained as described above, to define expressed genes (EGs) and non-expressed genes (NEs) for each pseudo-clone separately. We tested different thresholds for NEs to evaluate the ideal setup. The threshold≥0.95, for example, pertains to filtering out genes whose probability to be NE (based on the CNN) is equal to or larger than 95%. After filtering out genes classified as NEs, generalized linear model analysis (as available in the DEseq2 package) was performed to compare both pseudo-clones, which yielded $\log_2$-fold changes and *P*-values. Based on this, we defined the 'differential score' as a sign of $\log_2$-fold changes multiplied by $-\log_{10}$(FDR-adjusted *P*). As a measure of prediction accuracy of the 'differential score' to infer DEGs, we calculated AUC values using different numbers of "ground truth events" (represented by the top 10 up to top 100 differentially expressed genes identified through bulk RNA-seq; see e.g. **Fig. 1**, and **Fig. S7**). These examinations revealed the best performance when using the threshold≥0.90 to filter NEs, a setting that surpassed the use of generalized linear models alone for the inference of DEGs by NO analysis. scNOVA thus uses this threshold (≥0.90) by default. With this parameter setting, when controlling the FDR at 10%, scNOVA accurately captured 10/10 (100%) of the most overexpressed genes in RPE-1 (contributing a cell fraction (CF) of 77% in this simulation), compared to 7/10 for HG01573 (which contributed a CF of 23%).

9. NO-based inference of effects of multiple SVs using scNOVA

Given the strong influence of somatic SVs on the driver landscape of cancers, occasionally cells or clones will exhibit more than one SVs. In the presence of multiple SVs per sample or subclone, we recommend the following: (i) When using scNOVA to study local effects, we recommend analysis using the default strategy to scan +/-1 Mb adjacent to the respective SV breakpoints. (ii) For analyzing global effects with scNOVA, we recommend examination of all genes affected by the respective SVs

to identify candidate genes likely to be responsible for the observed gene or pathway alterations, and consideration of the respective SV class (e.g. inversion, deletion) that overlaps with (or is in very close proximity to) candidate genes.

10. Molecular phenotype analysis in gene sets in cell lines and leukemia samples

To identify a potential upstream regulator in the NA20509 and T-ALL_P1 subclones, we use the molecular phenotype analysis module of scNOVA with the first mode ('gene set over-representation analysis'; **Methods**). For NA20509, gene sets from the "ENCODE and ChEA consensus TFs from ChIP-X" category, provided by EnrichR[27], were used. For T-ALL_P1, we used TF target gene sets curated from the literature (**Methods, Table S7**) after realising that key TFs located in the chromothriptic region are not currently annotated in EnrichR.

To infer changes of pathway activity in the single-cells harboring 10q24.32 deletions in CLL_24, we used the molecular phenotype analysis module with the second mode ('joint modeling of differential NO across predefined gene sets'; **Methods**). Pathway level NO was compared between cells with and without 10q24.32 deletion based on linear mixed model fitting and likelihood ratio tests, using deletion status as a fixed effect and different plates (90hp1, 90hp2, 120hp1, and 120hp2) as a random effect (**Fig. 4d**).

11. Additional details with respect to the haplotype-specific NO analysis.

To infer haplotype-specific gene deregulation based on NO, we first filtered out genes inferred to be unexpressed (NE status probability$\geq$0.9) using scNOVA's CNN. For the remaining genes we computed gene body NO resolved by haplotype. For each gene, single-cell gene body NO from two haplotypes was compared using a wilcoxon ranksum test, controlled using an FDR of 10%. To allow inference of haplotype-specific NO at CREs, which we recommend to pursue locally (in proximity to SV breakpoints), scNOVA requires the location of annotated CREs as another input in bed format (REs_hg38.bed). CREs can be defined using DNase I hypersensitive sites (DHSs), or alternatively based on accessible chromatin segments obtained using ATAC-seq[71]. As a default functionality, scNOVA considers DHSs provided from 127 epigenomes[8] from the Roadmap Epigenomics Consortium and ENCODE (covering a variety of human tissue types). scNOVA also provides the option to use CREs from user-defined DNA accessibility profiling experiments (provided in 'bed' format). After aggregating haplotype-phased single-cell NO tracks into pseudo-bulk tracks, NO is measured based on assessing the read depth at defined CREs, using haplotype-resolved reads. Haplotype-specific NO is measured using the Exact Test followed by controlling the False Discovery Rate[13] (FDR), using EdgeR software[11]. CREs are assigned to their likely target genes using a nearest gene approach using the prioritisation rules described in[10].

12. Analysis of local effect of SVs in an AML patient

We applied haplotype-specific NO analysis as described above and in the Methods section to identify local effects of the clonal balanced translocation identified in AML_1. This analysis identified 11 genes with significant haplotype-specific NO, among which only *RUNX1T1* was identified as local SV effect, with the gene being well within the local search window (**Fig. 3, Fig. S17**). Following the identification of *RUNX1T1* through scNOVA, we inferred patterns of haplotype-specific NO at CREAs surrounding the *RUNX1-RUNX1T1* locus. We defined CREs active in AML by collecting accessible chromatin regions previously profiled by subjecting AML and normal hematopoietic cells to ATAC-seq (obtained from the GEO database – consensus CREs were defined as the union of peaks detected from at least one ATAC-seq dataset; see **Table S5**;). Haplotype-resolved single-cell NO measurements within CREs were scaled to reads per million (RPM). NO was normalised by locus copy number, in a haplotype-

aware manner. Average RPM values of single-cells at each CRE were transformed into log_2 scale using a pseudocount of 1.

We additionally considered a sliding window (300kb in size, moving 10kb each) along the derivative chromosome, to infer chromosome-wide haplotype-specific NO predictive for chromatin accessibility. For each sliding window, NO values at CREs from two homologues were compared using likelihood ratio tests to obtain nominal *P*-values *[P real]*. To control the type I error (multiple testing), we performed a permutation test by shuffling haplotype labels in the single-cell RPM matrix 1000 times. For each permutation we performed likelihood ratio tests to compare NO between the two haplotypes. We then computed the number of incidences we obtained the same or lower P-value than *[P real]* from 1000 randomizations, and divided this value by the permutation trials (*N*=1000) to estimate the permutation-adjusted *P*-value.

13. Analysis of local effect of SVs in T-ALL_P1

We revisited a 2.6 Mb 14q32 inversion identified in a T-ALL_P1, which we previously linked to outlier allele-specific expression of *TCL1A*[1]. In T-ALL_P1, scNOVA failed to detect *TCL1A*, one of the smallest genes in the genome (length: 4229 bp). We performed simulations, which revealed a minimum gene length requirement of 7219 bp to confidently detect haplotype-specific NO at gene bodies, a length requirement met by 80% of genes in the human genome (**Extended Data Fig. 6c**). However, this analysis revealed significant haplotype-specific NO at 18 genes, out of which *BCL11B* – a haplo-insufficent tumor suppressor in T-ALL [72]– was inferred as a local SV effect (with the gene residing <10kp away from the somatic inversion breakpoint). Notably, the inversion disrupts a "gene desert" region in 3′ of *BCL11B* previously described to comprise distal *BCL11B* enhancers[47,73], which suggests a concrete molecular mechanism – *i.e.* depletion of enhancer signals through repositioning[74]. Consistent with this notion, scNOVA inferred that *BCL11B* NO is significantly increased near the inversion breakpoint, indicating that *BCL11B* expression is decreased adjacent to an SV (**Extended Data Fig. 6a**) that depletes the region of known *BCL11B* enhancer elements. To validate scNOVA we performed bulk RNA-seq analysis, which confirmed allele-specific expression of *BCL11B* (FDR-adjusted *P*=2.0e-170) and demonstrate decreased expression on the respective SV haplotype, thus verifying the predictions made using scNOVA (inlet plot of **Extended Data Fig. 6a**). These data show that scNOVA allows deconvoluting local effects of copy-neutral SVs genome-wide, except for the smallest genes in the genome. In the case of T-ALL_P1, it notably appears that a 2.6 Mb copy-neutral inversion resulted in two 'hits' at once - mediating dysregulation of *TCL1A*[1] and silencing of *BCL11B*, a haploinsufficient tumor suppressor thought to collaborate with all major T-ALL oncogenic lesions[75].

Subsequent to these analyses, we performed a scan for haplotype-specific NO in annotated CREs near the SV breakpoint. To obtain the CRE positions in T-ALL_P1, ATAC-seq data of two biological replicates of T-ALL_P1 (EGAS00001003248)[76] were analyzed by aligning reads to the hg38 genome build using bwa[69] – allowing to define CREs in this T-ALL patient-derived sample. ATAC-seq replicates were merged using SAMtools[70], and open chromatin regions defined using the peak calling method provided through MACS[77] with the following parameters:

```
macs2 callpeak -t ATAC.bam -n output -g hs -q 0.05 --nomodel --shift -100 --extsize 200 -B --broad
```

Applying the exact test comparing haplotype-resolved NO at CREs identified an intergenic CRE (chr14:99319760-99320760, distal enhancer) with significantly increased NO nearby to inversion (FDR-adjusted *P*=0.05, **Extended Data Fig. 6e**), thus nominating this CRE as putatively contributing to the observed ASE of *BCL11B* in T-ALL_P1.

14. Analysis of local effect of SVs in BM510

We additionally applied the haplotype-specific NO analysis to identify local effect of a somatic balanced translocation detected in BM510 (present clonally based on 145 sequenced Strand-seq single

cell libraries). This analysis identified significant haplotype-specific NO at 69 genes. Among those, *NTRK3* (chr15:87,859,751-88,256,768), an oncogene[78] residing only 13.5 kbp away from the chromosome 15 breakpoint of its reciprocal t(15;17) translocation was nominated as the only SV local effect (**Extended Data Fig. 5a-b**). This haplotype-specific NO pattern is specific to BM510, with *NTRK3* showing no haplotype-specific NO signal in RPE-1, a closely related RPE cell line from the same donor lacking this translocation. Bulk RNA-seq data from BM510 are consistent with this local effect (ASE of *NTRK3* detected in BM510; FDR-adjusted *P*=2.76e-39). In line with a somatic effect, no ASE of *NTRK3* was seen for RPE-1 (FDR-adjusted *P*=1; see inlet plot of **Extended Data Fig. 5a-b**). No other gene near the SV breakpoint showed ASE based on RNA-seq – indicating that scNOVA accurately identifies haplotype-specific NO effects. We next performed an analysis of annotated CREs near *NTRK3* for haplotype-specific NO. We obtained CRE positions from the publicly available ATAC-seq dataset generated for the RPE-1 cell line[79]. We identified two CREs (chr15:87527100-87528100, distal enhancer; chr15:88246388-88247388, intronic) with a significant decrease in NO proximal to the translocation breakpoint (FDR-adjusted *P*=0.029; *P*=0.076, respectively; 10% FDR threshold; **Extended Data Fig. 5c-d**), which nominates these elements as candidate CREs putatively mediating or contributing to ASE of *NTRK3*.

15. Bulk-cell RNA-seq data processing and allele-specific expression analysis in LCLs

To define ground-truth differentially expressed genes between LCL and RPE-1 cell lines for parameterization of scNOVA, bulk-cell RNA-seq data was aligned to the human reference genome (GRCh38) with the STAR aligner (v2.5.3)[80], using the GTF file from ENSEMBL (GRCh38.81) with default parameters. Read counts for each gene were computed using HTSeq (v0.7.2)[81] by specifying the '-s no -t exon' option. Gene counts were normalized using the median-of-ratios method from DESeq2[23], and differentially expressed genes between conditions identified using the Wald test. To pursue allele-specific expression analysis, bulk-cell RNA-seq data were realigned to GRCh38 using GSNAP[82], using the variant-aware alignment mode to reduce allelic mapping biases. We resolved bulk-RNA-seq data by chromosome-length haplotype, using strand state and single nucleotide polymorphisms (SNPs) identified by Strand-seq (described in more detail below). ASEReadCounter[48] was used to compute allelic read counts (see also sections below).

NA12878. We pursued allele-specific expression analysis in NA12878 using bulk-cell RNA-seq data from ENCODE[4]. Allelic counts were assigned to either H1 (haplotype 1) or H2 (haplotype 2) based on the haplotype phasing obtained by StrandPhaseR[49]. SNP level allelic reads count were converted into gene level counts by summing up reads for each gene. Gene level counts of H1 and H2 were compared using the likelihood ratio test provided by EdgeR[11], followed by FDR adjustment. Genes with significant allele-specific expression (ASE) were identified using a FDR 10% threshold. Among ASE genes, monoallelic expressed genes were defined using definitions from a prior study, with genes showing a read-count proportion of the major allele >90% defined as monoallelic[83].

16. Bulk RNA-seq analysis in thirteen T-ALL patient-derived samples

Cells were collected from 13 pediatric T-ALL patients at the time of relapse to establish patient-derived-xenograft models as previously described[1,84]. Total RNA was extracted using TRIzol (Invitrogen Life Technologies). The RNA was then treated with TURBO DNase (Thermo Fisher Scientific) and purified using RNA Clean&Concentrator-5 (Zymo Research). We required a minimal RNA integrity number of 7, as measured using a Bioanalyzer (Agilent) with the Agilent RNA 6000 Nano kit. Cytoplasmic ribosomal RNA was depleted by Ribo-Zero rRNA Removal kit (Illumina), and RNA-seq libraries prepared from 1 μg of RNA using TruSeq RNA Library Prep (Illumina). These samples were sequenced on a Illumina HiSeq 2000 lane as 80 bp paired-end reads.

In order to confirm the subclonal perturbation of c-Myb in T-ALL_P1 predicted by scNOVA's infer altered gene activity module, RNA read counts were normalized using the median-of-ratios method from the DESeq2 package. Normalized read counts for each gene were standardized to obtain a Z score. After filtering out lowly variable genes (coefficient of variation (CV) of normalized read count < 25%), the average Z score of c-Myb target genes was calculated for each sample and plotted in **Fig. 5d** (we thereby considered the same c-Myb target genes as for the over-representation test in **Fig. 5c, Table S7**).

To verify allelic expression of *MYB* in the chromothripsis affected region in T-ALL_P1, we realigned reads to the human reference genome (GRCh38) using GSNAP as described above. Then we obtained haplotype-phased heterozygous SNP sites, based on the Strand-seq read data, using StrandPhaseR[2]. Using these phased SNP sites (input.vcf), haplotype-resolved allele-specific RNA read counts were obtained using ASEReadCounter[48], with the following parameters:

```
GenomeAnalysisTK.jar –R <reference.fasta> -T ASEReadCounter -o <output.csv> -I <input.bam> -sites <input.vcf> -U ALLOW_N_CIGAR_READS --minMappingQuality 10 --minBaseQuality 2 -drf DuplicateRead
```

SNP level allelic reads count were summarized into gene level counts by summing up reads for each gene. Gene level counts of H1 and H2 were compared using the likelihood ratio test provided by EdgeR[11], followed by FDR adjustment (**Fig. S30**).

17. Bulk RNA-seq analysis in 42 CLLs

In order to further corroborate the subclonal gene activity changes inferred in CLL_24, we performed bulk RNA-seq in a cohort of 42 CLL samples, which included CLL_24. Leukemia cells were isolated from blood using Ficoll density gradient centrifugation. Cells were viably frozen and kept on liquid nitrogen until use. Cells were thawed, allowed to recover in RPMI medium (Thermo Fisher Scientific) containing 10 % human serum (Sigma Aldrich) for 3h and filtered through a 40 µm cell strainer. Tumor cells were collected by Magnetic-activated cell sorting (MACS) using CD19 beads (Miltenyi Biotec). RNA was isolated using QIAzol Lysis Reagent (Qiagen), QIAshredder (Qiagen) and the RNeasy Mini Kit (Qiagen). Stranded mRNA sequencing, using a TruSeq Stranded Total RNA Library Preparation Kit was performed on a Illumina NextSeq 500. These RNA-seq data had originally been aligned to GRCh37.75/hg19 using STAR (v2.6.0c)[80] and counted with htseq-count[81]. For the purpose of this study, we made use of the resulting gene-level count table of protein-coding genes of interest (*i.e.*, such mapping to the relevant pathways uncovered with scNOVA). 59 CLLs were initially available to us, from which we removed *N*=8 samples exhibiting chromosome 17q13 deletions targeting *TP53*, since CLLs with *TP53* aberrations form a clinically distinct subset of CLLs[85], and since cross talk between the p53 and Wnt signaling has been reported, with p53 loss promoting Wnt signaling[86,87]. We additionally removed *N*=9 samples exhibiting trisomy 12, as this group of samples is known to express a unique set of pathways when compared to other CLL samples[88]. RNA read counts of the remaining 42 CLLs were normalized, Z-scores were derived, and lowly variable genes were filtered out as described above. To measure Wnt signaling activity from these transcriptomic data, we obtained 49 known target genes of TFs involved in Wnt canonical signaling (*CTNNB1*, *LEF1*, *TCF7*, and *TCF7L2*) from the TRRUST database[89], here called 'Wnt signaling target genes (**Table S7**)'. The mean Z score of 49 genes was computed and visualized. CLL_24 showed the most pronounced bulk RNA-seq overexpression of Wnt signalling pathway members (ranking first amongst all of the 42 considered CLL samples of this cohort; **Fig. S24**).

We also analysed bulk RNA-seq data of CLL patients from the ICGC resource[33], by considering 395 CLL samples with available SCNA data. To be consistent with the analysis of CLL primary samples from the Heidelberg-based cohort, we removed samples exhibiting trisomy 12 or 17q13 deletions affecting *TP53*, which yielded 306 donors in total. We observed somatic 10q24.32 losses (deletion) in

six out of 306 donors (**Fig. 4b**). Among those 306 donors, bulk RNA-seq data was available from 178 donors including 4 donors with 10q24.32 losses overlapping the minimal deleted regions defined in CLL_24. RNA read count of the 178 CLL samples were normalized, Z scores were derived, and lowly variable genes filtered out as described above. Mean Z score of Wnt signaling target genes of the donors with and without 10q24.32 deletions were compared using the generalized linear model (GLM) likelihood ratio test, controlling for gender and age (**Fig. 4e**).

18. Haplotype-resolved bulk RNA-seq analysis in LCLs from HGSVC consortium

To verify the subclonal activation of c-Myc/Max in NA20509, we analyzed bulk RNA-seq data in the whole panel of LCLs recently used by the Human Genome Structural Variation Consortium (HGSVC) to construct an SV germline reference resource[31]. RNA read count of the 33 LCLs were normalized, Z scores were derived, and lowly variable genes were filtered out as described in section 10. The mean Z score of c-Myc/Max target genes was calculated and visualized using a bargraph (**Fig. 2d**). c-Myc/Max heterodimer target genes were downloaded from the Molecular signatures database (Msigdb, **Table S7**)[90].

To examine the allelic expression of genes residing in regions of complex chromosomal rearrangement in NA20509, we firstly detected SNP sites from the pooled NA20509 Strand-seq libraries using freebayes[91]. Using these SNP sites (input.vcf), allele specific read counts were obtained using ASEReadCounter[48] with the following parameters:

```
GenomeAnalysisTK.jar –R <reference.fasta> -T ASEReadCounter -o <output.csv> -I <input.bam> -sites <input.vcf> -U ALLOW_N_CIGAR_READS --minMappingQuality 10 --minBaseQuality 2 -drf DuplicateRead
```

To haplotype-resolve allelic counts to either the unaffected homolog (haplotype 1) or the homolog bearing the BFB (haplotype 2, *i.e.* the derivative chromosome), we used the strand states of Strand-seq reads (Watson (W) or Crick (C)) along chromosome 17. In single cells in which the two homologs of chromosome 17 have a WW majority configuration[1] (WW strand state seen for most of chromosome 17), the BFB-mediated inverted duplication[1] will always exhibit DNA reads on the C strand belonging to haplotype 2 (the same applies to single cells with a CC majority configuration on chromosome 17, for which W reads belonging to haplotype 2 can be extracted from the inverted duplication). Among the 40 single-cells containing the BFB event, we could collect 14 cells with either a WW or CC majority configuration, allowing extraction of reads from the derivative chromosome haplotype; similarly, we collected 8 cells with either a WW or CC majority configuration for chromosome 5, which enabled extraction of reads from the terminally duplicated haplotype. These operations unambiguously phase-resolved 138 genes located on the derivative chromosome, with at least 2 phased heterozygous SNPs seen for *MAPK9,* and *PDGFRB*. RNA phased-resolved allelic read counts for these SNPs were compared using the likelihood ratio test followed by FDR-adjustment (**S14i**).

19. Clinical diagnostic information for CLL_24

CLL_24 was obtained from the peripheral blood mononuclear cells of a previously untreated female CLL patient with age 61 at sampling. According to routine diagnostic methods, the patient sample showed no IGHV hypermutation, had no *TP53* mutation, and had no alterations at 6q21, 8q24, 11q22.3, 12q13, 13q14 and 17p13.

20. Clinical diagnostic information for AML_1

This sample was obtained as a diagnostic bone marrow from the first aspiration of an AML with a t(8;21) translocation (known to result in *RUNX1:RUNX1T1/ETO:AML1* gene fusion), arising after cytostatic therapy for testicular cancer in a young man. 95% of cells were identified as blasts with monocyte differentiation by microscopy. In the initial diagnostic flow cytometry characterization, 65%

of cells showed monocyte differentiation markers and were positive for CD33, CD13, CD38, HLA-DR, CD11c, and CD15. A subpopulation of these blasts were CD34+/CD117+ and also partly positive for CD19 (common in t(8:21) AML). This sample also carried a *FLT3*-TKD mutation (p.Asp835Tyr, CF=44%).

21. 10q deletion discovery in CLL samples from PCAWG

To assess the frequency of deletions affecting 10q24.32 in CLL we analyzed 94 CLL samples included in the ICGC/TCGA Pan-Cancer Analysis of Whole Genomes (PCAWG) resource [34]. We first generated GC and mappability corrected fragment counts for the paired-end WGS of each sample using Delly[30]. These normalized fragment counts were then binned in 10kbp windows and screened for chr10q deletions, using Delly2[30]. As shown in **Fig. S23**, at least 4 out of the 94 CLL samples (4.3%) harbor somatic deletions intersecting with the 10q24.32 minimal region.

22. Strand-seq in a panel of lymphoblastoid cell lines (LCLs)

24 EBV-transformed LCLs (Coriell Institute) were cultured in BrdU (100uM concentration; Sigma) for 18 or 24 hours, and single isolated nuclei (0.1% NP-40 lysis buffer[92] sorted into 96-well plates using the BD FACSMelody cell sorter (NA12329, NA18534, NA18939, NA19650, NA19983, NA20509, NA20847, HG00096, HG00171, HG00864, HG01114, HG01505, HG01596, HG02011, HG02492, HG02587, HG02818, HG03009, HG03065, HG03125, HG03371, HG03486, HG03683, HG03732). In each sorted plate 94 single cells, one 100-cell positive control and one 0-cell negative control were deposited. Strand-seq libraries were prepared, sequenced and selected using the same protocol as for the primary leukemia samples. These LCLs were previously released[31] and used to construct a haplotype-resolved germline SV resource in a human population diversity panel by the Human Genome Structural Variation Consortium[31] A mean of 54 high-quality single cells (41 to 71 cells) were sequenced to a median depth of 338,271 mapped nonduplicate fragments per cell. We used a threshold of CF≥10% for discovering unwanted somatic SVs in these LCLs, using the scTRIP method[1]. Translocation discovery was pursued using the 'majority mode'[1].

23. WGS-based subclonal SV analysis in NA20509

The availability of NA20509 WGS data from the New York Genome Center[93] allowed us to attempt verification of the presence of somatic SVs. We first aligned the data to GRCh37 using bwa[69], called SNPs and InDels using freebayes[91] and haplotype-phased variants using eagle2[94] using the 1KG phase 3 reference panel[52]. Phased haplotype blocks were used to identify heterozygous sites deviating from the expected 1:1 ratio, in conjunction with GC and mappability corrected read-depth plots (**Fig. S13**) generated using Delly2[30]. Phased heterozygous sites and read-depth estimated copy numbers deviated from the expected pattern for copy number 2, and thus independently confirmed the presence of subclonal SVs in NA20509, including the terminal gain on chromosome 5, and the terminal loss of the chromosome 17 p-arm with an adjacent gain event (**Fig. S13**). SV analysis using Delly2[30] also verified the presence of a subclonal translocation from chromosome 17 (position 21,479,415) to chromosome 5 (position 132,093,890). Delly2 further revealed a tail-to-tail inversion-type rearrangement at chromosome 5 with breakpoint positions 132,029,510 and 132,053,373, and a tandem duplication-type rearrangement spanning the terminal gain on chromosome 5 with breakpoint positions 133,831,567 and 178,087,753 (GRCh37 coordinates).

24. Manual curation of somatic SVs in LCLs to achieve a high-quality callset

We used scTRIP (methods from the MosaiCatcher pipeline)[1] to discover somatic SVs in LCLs. The extent and diversity of deletions at 22q11.2 intersecting the immunoglobulin lambda locus (*IGL*) motivated additional curation of these somatic SV events. 17/25 LCLs harbored deletions at 22q11.2.

Making use of the somatic SV calls and single-cell segmentation results from the MosaiCatcher pipeline[1], we defined putatively deleted segments, which we subjected to manual inspection in single cells followed by reanalysis using the ArbiGent SV genotyping tool[31]. ArbiGent performs analysis of Strand-seq data cell-by-cell, to allow precise haplotype-resolved genotype assignment into homozygous and heterozygous deletion events, at defined genomic intervals. These genotype assignments were accepted if the log10 likelihood ratio between SV and reference state was greater than 0.5, and as such – in a few cases – superseded variant calls created with the MosaiCatcher pipeline.

Additional curation and analysis of NA12878 somatic SVs. NA12878 is perhaps the single most sequenced human cell line presently existing[4,52,95]. We thus regarded the discovery of previously unknown somatic SVs in this cell line as a surprise, which motivated careful curation and manual inspection. Altogether, we analysed 75 Strand-seq libraries from NA12878, including cells prepared as a single batch[2], to allow for robust single-cell somatic SV discovery. 59 single-cell genomes harbored a somatic 22q11.2 deletion (chr22:22200000-22900000) on haplotype 2 (H2) intersecting the *IGL* locus. Application of scNOVA revealed differential gene activity patterns between these *IGL* locus deletion bearing cells (denoted clone 2), and cells unaffected by 22q11.2 SVs (clone 1) – revealing 8 significant genes (10% FDR). Out of these 8 genes, 5 genes were located in a short chromosome 19 interval and inferred to be less occupied by nucleosomes due to the decrease of read depth in clone 1. Prompted by this observation, we performed manual inspection of this region, which revealed that clone 1 harbors a ~500kb hemizygous deletion on chromosome 19, which confidently maps to haplotype 2 (H2), but which was missed in the initial single-cell population segmentation pursued using the MosaiCatcher pipeline[1]. Separate segmentation of individual cells using the MosaiCatcher pipeline fine-mapped this candidate deletion to a 500kb interval (chr19:36500000-37000000), thus corroborating its presence by single-cell SV discovery. We further subjected this candidate interval to cell-by-cell genotype analysis using ArbiGent[31], which in line with the MosaiCatcher pipeline genotyped a high-confidence somatic deletion at chr19:36500000-37000000. ArbiGent genotype calls were accepted in cells where the log10-likelihood-ratio between reference and deleted states was larger than 0.5, allowing confident genotyping in 65/75 (87%) of cells. ArbiGent revealed strict mutually exclusivity between this interstitial deletion on chromosome 19, and 22q11.2 somatic deletions (**Table S8**) – mirroring the pattern we had observed for NA20509. As described in the section below, this chromosome 19 deletion is also verified by scRNA-seq data from different NA12878 cell stocks. Finally, we further used Arbigent to analyse 20 additional NA12878 Strand-seq libraries, prepared as a separate biological replicate[2], which once again showed the presence of subclones carrying mutually exclusive interstitial deletions on chromosome 19 and at 22q11.2. These results hence show the presence of different somatic subclones in the key NA12878 human reference model cell line.

25. scRNA-seq data analysis for inferring somatic copy number alterations (SCNAs)

Discovery mode

Three broadly used single-cell transcriptome based SCNA analysis tools InferCNV[50], HoneyBADGER[96], and CONICSmat[24] were used for SCNA discovery. As inferCNV and HoneyBADGER require matched normal cell annotations, we first defined normal cell population if it's available within the same sample. If not, we downloaded cell-type matched normal cell profiles from the GEO database as reported in **Table S4**.

To run InferCNV, we provided single-cell count matrices with analysis_mode = 'subclusters', cutoff=0.1 for 10X, cutoff=1 for SMART-seq and Fluidigm as recommended in the manual. For HoneyBADGER, CPM normalized single-cell count matrices were converted into log(CPM+1), and put into HoneyBADGER for the CNV discovery with default parameters. CONICSmat is originally developed for 'targeted SCNA recalling' mode for estimating copy number of candidate SCNA regions

obtained from DNA-seq data, however it also provides chromosome-arm level SCNA discovery in case no matched DNA-seq data is available. For this, above mentioned $\log_2$(CPM/10+1) – the standard input values for CONICSmat were put into CONICSmat for the chromosome-arm level discovery.

Targeted SCNA recalling mode

Single-cell count matrices were normalized to obtain count per million (CPM) values. These values were converted into $\log_2$(CPM/10+1) – the standard input value for CONICSmat, a tool for inferring the copy number state of chosen candidate genomic regions using scRNA-seq data[24].

For each of the candidate SV regions, we applied CONICSmat, with parameters set to allow considering regions with at least 10 expressed genes. Firstly to verify the presence of SCNAs in regions of interest, CONICSmat generates distributions of average expression levels across single-cells, and then fits to the 1-component and 2-component mixture models[24]. It further compares the likelihood ratios of being 1-component (unimodal, absence of subconal SCNAs) and 2-component (bimodal, presence of subclonal SCNAs) to determine the most likely state in those regions based on the Bayesian information criterion (BIC). Candidate SCNAs likely to be bimodal using a 1% FDR criterion were considered further for downstream analysis. For those candidate SCNAs, the posterior probability of each single-cell to be belonging to the SV component, and the 'Reference' (Ref) component was computed. Single-cells with a posterior probability above the cutoff for one of these two components were used in downstream analyses.

26. Pseudotime/cell-type analysis of scRNA-seq data

Pseudotime analysis was carried out using the R package Slingshot[97], implementing UMAP and cell clusters identified using Seurat (see Methods for further details.). Cell type analysis was performed using UCell (as per main methods), using the T cell differentiation stage-specific gene sets outlined in [51].

27. Generation of composite track for AML_1 translocation

To allow fine-resolution analysis of the AML_1 translocation breakpoint, composite tracks of Strand-seq data were generated separately for the chromosome 8 and chromosome 21 breakpoint regions, and the resulting tracks produced using BreakpointR[44]. For the chromosome 8 track, 11 cells in the WC orientation for the majority of the chromosome and the translocated segment in the WW or CC orientation were used to generate the composite tracks. Cells with the translocated segment in the WW orientation were merged into a single bam file, and the merged file converted to bed format using bedtools bamtobed. Directionality of the reads was then manually inverted (i.e. all '-' reads changed to '+', and vice versa), and the bed file converted back to a bam file. Finally, this bam file was merged with the cells harboring the translocation in the CC orientation; giving a pseudo-bulk bam file of cells with the same directionality. Bedgraphs of directional reads, breakpoints and confidence intervals were then generated using the BreakpointR package, and the tracks were visualized in the UCSC genome browser. The chromosome 21 composite track was generated in a similar manner, with the following differences: 11 cells in which the majority of the chromosome was in the WW or CC orientation were used. Cells with the majority of the chromosome in the WW orientation were inverted for read-directionality as above, and the same workflow used to produce directional bedgraphs and breakpoints for visualization in the UCSC genome browser.

28. Overlap analysis of SV calls and haplotype-specific NO in NA12878

When mapping haplotype-specific NO at CREs in NA12878, in order to reduce false positive hits resulting from small (<200kb) duplications and deletions, we used the high-resolution SV call set recently generated by long-read sequencing through the Human Genome Structural Variation

Consortium[31] (HGSVC), based on 64 unrelated haplotype-resolved genome assemblies. These data contain 12,703 heterozygous SVs in NA12878, including 5,315 deletions and 7,388 insertions. When we compared the NA12878 call set with haplotype-specific NO calls generated by scNOVA, the vast majority (718 among 727 haplotype-specific NO calls [98.8%]) did not overlap any SVs. 9 haplotype-specific NO calls were excluded, as they overlap SVs and thus may potentially confounded by copy number variants. Following the exclusion of these haplotype-specific NO calls, we calculated the enrichment of chromosome X CREs (P=0.015, hypergeometric test), and also the enrichment of genes showing allele-specific expression (ASE) in **Fig. S2b** (P=0.0018, hypergeometric test).

29. Comprehensive summary of the CITE-seq analysis

After removing low quality single cells based on the percentage of mitochondrial reads (<15%), the total read count (<15000), and number of unique genes detected (>200), we obtained 4,459 high quality single-cells. We further subsetted the data to ensure we profiled exclusively B-cells (with CLL arising from the B-cell lineage). After unsupervised clustering of the data using the Seurat standard workflow (resolution = 0.5, to minimize the granularity of the clustering) (**Fig S25a**), we used the human cell atlas (HCA) bone marrow dataset[54] to carry out a reference-based cell type annotation of the data (**Fig S25b**). These annotations indicated that clusters 0-3 likely correspond to B-cells. Further supporting these cell-type identities, we observed that these clusters expressed high levels of *CD19* RNA and protein (**Fig S25c, S25d**), a canonical B-cell marker in both healthy and leukemic cells[98]. We next examined the expression of 6 genes highly associated with leukemic B-cells[55], which showed high average expression in clusters 0-3. Therefore, we subsetted to just cells from clusters 0-3, and reran data scaling, variable feature detection and unsupervised clustering using Seurat's scTransform function (resolution = 0.8, percentage of mitochondrial reads was regressed out), which resulted in 3,919 high quality B-cells used for further downstream analysis.

Using these cells, we initially attempted to identify SCNAs using InferCNV[50], HoneyBADGER[96], CONICSmat[24]. From this analysis we were unable to discover any somatic SV subclones within the CITE-seq data (**Table S4, Extended Data Fig. 7a**), in spite of the fact that the SV region comprises 152 expressed genes for the 10q-terDel (SCb) and 24 expressed genes in the minimally deleted region (shared by SCa, SCb, SCc and SIa-d). However, when we applied the targeted SCNA recalling mode of CONICSmat leveraging the 10q-terDel breakpoint identified through Strand-seq, we could assign 82 single-cells confidently to the SCb (10q-terDel) subclone and 2,381 single-cells to confident normal cells showing absence of the 10q-terDel event (**Extended Data Fig. 7b**) – resulting in a similar estimated CF of the 10q-terDel subclone to that identified in the Strand-seq data (2.14% and 3.49%, respectively). None of the other SVs discovered with Strand-seq could be confidently called using the targeted SCNA recalling mode of CONICSmat, presumably due to the smaller size and lower number of expressed genes affected by the respective deletions. Overall, these data suggest limitations of scRNA-seq based SCNA calling outside of highly copy number unstable cancer types[34].

We coupled this karyotypic heterogeneity with molecular phenotype heterogeneity by performing unsupervised clustering of the scRNA-seq data based on DoRothEA regulons[56] and VIPER[99] TF activity scores, which identified 8 clusters of cells exhibiting differential TF activity (**Fig. S26a**). Single-cells which were inferred to contain the 10q-terDel by CONICSmat showed significant enrichment in cluster 0 (Fisher's exact test, adjusted P = 1.06e-07), while confident 10q-Ref cells showed significant enrichment in clusters 4, 5, and 6 (Fisher's exact test, adjusted P = 0.044, 0.006, and 0.006 respectively) (**Fig. S26b, S26d**). Next we annotated each single cell with an UCell score[100], which measures the gene set-level activity of the 10q-terDel-associated gene signature inferred by scNOVA. In agreement with the CONICSmat-based targeted SCNA recalling, we found cluster 0 to be significantly enriched for cells with a high UCell score (Fisher's exact test, adjusted P = 1.74e-26; cutoff for high score = > median UCell score + standard deviation) followed by a somewhat less pronounced

trend seen for cluster 3 (adjusted P = 6.56e-3) (**Fig. S26c, S26d**). These orthogonal analyses therefore suggested that the 10q-terDel clone is highly enriched in scRNA-seq cluster 0 (**Fig. S26d**). Notably, cluster 0 showed 641 up-regulated genes and 69 down-regulated genes compared to clusters 4-6, and those 641 up-regulated genes were over-represented by Wnt signaling pathway genes (Fisher's exact test, adjusted P = 0.028, **Fig. S26e**), which supports the association between the terDel seen in SCb and Wnt aberration.

Having confidently identified the SCb (10q-terDel) subclone in the CITE-seq data, we compared the DoRothEA/VIPER-inferred TF activity of '10q-terDel' (N=82 cells) to the '10q-Ref' (N=2381 cells) cells to identify TFs with altered activity in the 10q-terDel clone (**Fig. 4h**). The 43 differential TFs from DoRothEA analysis (p.adjust<0.1; likelihood ratio test) contain 2 diffNO signature genes from scNOVA analysis of the SCb (10q-terDel) clone (*TCF3, ATF7*, both are predicted to be more active in the SCb (10q-terDel) clone from scNOVA) and 11 TFs which match with TF-target enrichment analysis of SCb-(10q-terDel-) activated gene signature (**Fig. S21**). Note that *NFKB2* - previously suggested as a potential target gene of recurrent somatic 10q24.32 deletions[39] was tested in this DoRothEA/VIPER analysis, but it did not show significant difference between 10q-terDel and 10q-Ref cells (FDR 10%).

Overall, the 43 differential TFs identified through the DoRothEA analysis showed enrichment for Wnt signaling, BCR signaling, and the PD-1 checkpoint pathway in cancer (adjusted P = 0.011, 0.0014, and 0.042 respectively; ConsensusPathDB[57]). *NFATC1* is one of the differentially active TF in terDel clone supported by both scNOVA and CITE-seq, and involved in Wnt signaling[101] and BCR signaling[102], and positively regulates PD-1 expression[103]. Interestingly, the terDel bearing cells showed significant over-expression of PD-1 at the protein level measured by CITE-seq (**Fig. 4i**), even relative to cells from within the same cluster (cluster 0). Elevated *PD-1/PD-L1* is known to confer increased immune resistance to CLL cells[104]. These data collectively suggest that the terDel subclone activates leukemia-related signaling pathways including Wnt signaling, results in *NFATC1* activation and *PD-1* overexpression.

30. Minimum gene length required to detect haplotype-specific NO at gene body

scNOVA identifies haplotype-specific NO based on the FDR-corrected Wilcoxon ranksum test of haplotype-resolved single-cell NO at gene body. Gene lengths in the GRCh38 genome range from 74bp to 2,304,996bp, which motivated us to evaluate the minimum gene length required to allow for reliable detection of haplotype-specific NO in gene bodies. To estimate this, we simulated haplotype-specific NO for each gene by assigning all reads to one haplotype, and assigning '0 coverage' to the other haplotype. Then we used the Wilcoxon ranksum test implemented in the scNOVA pipeline, to compute FDR-corrected p-values estimating haplotype-specific NO for each simulated genes. We stratified the genes into 20 bins according to their length. For each bin, we calculated the proportion of genes we recover as a significant hit (FDR 10%) in the above simulation. In the case of the lowest size bin (0~5 percentile, which corresponds to 74 bp to 1678 bp), we recovered none of the genes due to lack of read counts. In the case of the 20th bin with the highest gene length (95~100 percentile, which corresponds to 256,255 bp to 1,304,996 bp), we recovered 94.75 % of genes as a significant hit.

When we performed this analysis across all bins (**Extended Data Fig. 6c**) from the 1st lowest size bin (0~5 percentile) to the 20th bin (95~100 percentile), the 5th bin (20-25 percentile, corresponds to 7219 bp to 9528 bp) began to recover over 50% of simulated genes as significant hit (above the red line). Based on this we concluded that > 7219 bp (20 percentile of gene length) is required, to recover more than half of simulated genes as a significant hit and 80% of genes fulfill this criterion.

Supplementary Discussion

I. Scope of scNOVA compared to single-cell multiomics methods focusing on SCNAs

To evaluate the scope of scNOVA in relation to prior single-cell multiomics method focusing on large-scale (>10Mb) SCNAs, we analysed the abundance of different classes of somatic driver mutation, differentiated by variant class and size, using the PCAWG resource of 2,658 whole-cancer genomes[34]. We downloaded the PCAWG set of patient-centric drivers (https://dcc.icgc.org/releases/PCAWG/driver_mutations)[34,105] to compute the relative abundance of driver mutation classes: Across PCAWG, out of a total of 13,219 annotated drivers, 5,913 represent somatic point mutations (including single nucleotide variants, SNVs; multi-nucleotide variants, MNVs; and <50bp short insertions and deletions (indels)), 6,490 represent copy number imbalanced SVs and another 816 are copy number balanced SVs. Hence somatic SVs (defined as copy number imbalanced+copy-balanced SVs) contribute 55% of all annotated somatic drivers, and significantly outnumber compared to point mutations in a typical cancer genome[34]. Second, to enable comparison of SV drivers by size and class we generated a consolidated list of somatic SV drivers from PCAWG with resolved breakpoint coordinates, and computed confident size estimates from these (achieved for N=3,342 SVs). Out of these, 83% (2,765 SVs) were ≥200kb in length and thus at the size range[1] accessible to scNOVA. By comparison, 37% (1,244 SVs) represented SCNAs ≥10Mb in length.

II. Known and suspected Wnt signaling regulators near 10q24.32

A closer analysis of genes located within the relevant 10q arm showed that several Wnt signaling genes reside in this genomic region (**Fig. 4b, S28**). Indeed, six genes implicated or suspected to suppress canonical Wnt signaling, *SUFU* (deleted in 11/11 cells with 10q24.32 SVs), *FBXW4* (11/11), *NFKB2* (11/11), *LDB1* (11/11), *BTRC* (9/11) and *LZTS2* (5/11), were repeatedly deleted in CLL_24, and represent candidates for further study: *BTRC*, an F-box containing protein, has been shown to be involved in ubiquitin mediated proteolysis of β-catenin to maintain low levels of β-catenin in the cytoplasm, and as such influence Wnt signaling[106]. *BTRC* has been reported as mutated in multiple human cancer cell lines and clinical tumor samples[107], is located close to the 10q24.32 minimal region (<60kb apart), and is significantly downregulated in CLLs from the ICGC that harbor a 10q24.32 somatic deletion (FDR-adjusted P=0.000646; **Fig. S28**). *FBXW4*, located within the minimally deleted 10q24.32 region, has been implicated in tumor suppression [108] and as another F-box containing protein has been suspected, albeit not yet shown, to play a roles in ubiquitin mediated proteolysis of β-catenin[109]. Like *BTRC*, we observed *FBXW4* to be significantly downregulated in CLLs from the ICGC that harbor a 10q24.32 somatic deletion (FDR-adjusted P=0.00478; **Fig. S28**). *SUFU*, a tumor suppressor gene located in the minimally deleted region, can suppress Wnt signaling by forming a complex with β-catenin, and by enhancing β-catenin translocation to the cytoplasm[110]. *SUFU* loss of function has been shown to result in the failure to suppress Wnt signaling in medulloblastoma (with loss of function of *SUFU* mediating overactivity of both the Sonic Hedgehog signaling pathway and the Wnt signaling pathway in medulloblastoma)[111]. *LZTS2*, deleted in 5/11 cells exhibiting 10q24.32 somatic deletions, is likewise a tumor suppressor and a known negative regulator of Wnt signaling[112]. *NFKB2*, located within the minimally deleted 10q24.32 region, was previously proposed – albeit not yet shown – to be involved in the pathogenic effect of 10q24 deletion in CLL[35,113,114]. *NFKB2* is a gene that encodes a subunit of the transcription factor complex nuclear factor-kappa-B (NF-kB) which is essential for lymphocyte development and immune function[115]. NF-kB signaling negatively regulates the Wnt/β-catenin pathway either indirectly through the functions of NF-kB target genes (e.g., *LZTS2*) or directly by interfering with the formation of transcriptional complex β-catenin/TCF/p300[116]. In addition, LDB1 negatively regulates Wnt β-catenin in various model systems[117].

Furthermore, we found *CTBP2* and *PTEN* which are known to negatively regulate Wnt

signaling in the uniquely deleted regions of SCb (terDel) and SCc (twoDel) clones which might explain the heterogeneity of signaling alteration we observed from scNOVA. *CTBP2* acts as an antagonist of beta-catenin/TCF activation[118] and loss of *PTEN* is associated with increased active beta-catenin[119]. Notably, the presence of uniquely deleted negative regulators of Wnt signaling (*CTBP2* for SCb and *PTEN* for SCc deletion) provides a potential explanation for the somewhat different functional effects of those deletions predicted by scNOVA.

III. Nucleosome repeat length measurements: considerations for future users

The main text reports very similar nucleosome repeat length[6] estimates when comparing Strand-seq (195.4 ±0.4bp) and MNase-seq (193.7 ±0.6bp). Thus, nucleosome repeat length estimates from Strand-seq are, encouragingly, highly reproducible and consistent with bulk MNase-seq; this formed the foundation of our work. Some considerations on the measurement of nucleosome repeat length are given here, to guide future users:

During Strand-seq library preparation single-cell genomic DNA is fragmented using MNase. As described extensively in [92], the protocol calls for precisely 0.5U of MNase per single cell reaction, adding the enzyme immediately after diluting into fresh Master Mix. The incubation time should then proceed for precisely 8 minutes before stopping the reaction by addition of EDTA. All steps of MNase digestion reaction were previously optimized to generate mononucleosomal fragments of 150~200 bp in length. The timing and enzymatic activity are critical for the digestion procedure, and therefore any deviations in these components can result in changes in the DNA fragmentation pattern seen in the sequenced libraries. Furthermore, after library preparation there is a final size-selection step, upon pooling the single cell libraries. During this step the 250-350bp band (including adapter sequences) is specifically excised to enrich the mononucleosome fragments. Changes to the size selection step or possible fragment contamination during size selection can change the fragment lengths present in the final sequence data, for instance if dinucleosomal fragments are also included in the excised band (e.g. as seen in the NA12878 raw data, before processing; see **Fig. S1**). Looking forward, we advise users to carefully control the size selection step and the digestion procedure, to ensure that the same reproducibility of nucleosomal patterns as observed for our automated Strand-seq library generation pipeline[1] can be achieved.

IV. Further details of functional outcomes of somatic rearrangement landscapes in lymphoblastoid cell lines

17 LCLs exhibited deletions 200–700kb in size, comprising the *IGL* locus on chromosome 22q11, present at CFs from 1.4% up to 89%. 13 LCLs (52%) showed homozygous loss events, and 8 LCLs (32%) exhibited two or more subclones harboring distinct 22q11.2 deletions (**Table S3, Fig. S9**). 22q11.2 undergoes V(D)J rearrangements during B-cell development, a physiological process resulting in up to ~1Mb-sized deletions[120], suggesting that these SVs likely emerged as a result of normal B-cell biology. However, since the expression changes conferred by 22q11.2 deletions in cultured LCLs are unknown, this alteration provided a test case for linking SV to a molecular phenotype. Using scNOVA, we inferred the lncRNA *FLJ22447* (NCBI Gene ID: 400221), present on 14q23, to be upregulated in cells with the 22q11.2 deletion (22q-Del) compared to cells with a normal 22q status (22q-Ref) (**Table S2**). Although so far only poorly characterized, this lncRNA was shown to be upregulated in carcinoma-associated fibroblasts where it reinforced interleukin-33 signalling, promoted a proliferation phenotype, and correlated with poor disease prognosis in oral cancer[121]. How this lncRNA is dysregulated via 22q11.2 deletion remains to be seen, but it is possible that overexpression of this gene may support the expansion of cultured LCLs harbouring 22q11.2 SVs.

V. Comparison of haplotype-specific CRE activity in the chromosome X of female genome inferred by scNOVA, and reported by previous studies

In this study, haplotype-resolved scNOVA analysis in NA12878 showed that haplotype-specific CREs are 1.4 fold enriched for chromosome X compared to autosomes (**Figure S4**). This observation (1.4 fold enrichment) is in line with the haplotype-specific chromatin activities previously measured using ChIP-seq[12] and thus is likely to reflect X chromosome biology. In this previous study, 15% of TF binding sites showed allele specific signals on chromosome X, compared to 2-10% on the autosomes[12]. Based on this data, only a subset of chromosome X TF binding sites are haplotype-specific (15%), and haplotype-specific events are 1.5~7 fold enriched based on the TFs[12].

VI. Germline copy number status of *MAP2K3* locus in NA20509

According to the recently published T2T-CHM13 assembly annotation[122], the BFB amplified region in NA20509 is near a collapsed duplication (containing *KCNJ18* gene) – but it does not directly overlap this collapsed segment. However, further analysis of the distal region of BFB amplification based on dot plots of the T2T-CHM13 assembly aligned against GRCh38 revealed a duplication of *MAP2K3* gene in the distal region of the amplified segment.

Motivated by this data, we checked the germline copy number status of *MAP2K3* in NA20509 and HG1505 (control cell line which doesn't harbor subclonal SVs) using DNA qPCR experiments. These experimental data suggested the germline copy number of the *MAP2K3* locus is estimated to be 3 in both cell lines (**Fig. 2f**)[122]. DNA qPCR of NA20509 early (p4) and late (p8) showed a 1.33 fold increase of copy number from 3 to 4 copies in the late passage (p8). We further checked the RNA expression of *MAP2K3* in NA20509 early and late passage using RNA-seq. It showed a 1.39 fold increase of *MAP2K3* expression, in agreement with somatic copy number gain (**Fig. 2g**).

VII. Analysis of the CT gene signature inT-ALL_P1

When we applied scNOVA to the 6q-CT event seen in T-ALL_P1 (CF=30%), we identified 12 genes with differential NO between 6q-CT and 6q-Ref cells (for simplicity, denoted 'CT gene signature'; 10% FDR; **Fig. 5a-b**; **Table S2**). The dysregulated genes mostly (10/12 (83%)) resided in genomic regions other than 6q, suggesting that predominantly global (*trans*) regulatory effects arose from the 6q-CT event. Half (6/12) of the CT gene signature were well-known targets of c-Myb (**Fig. 5b-c**; **Table S6**), of which 5 were predicted to be upregulated in 6q-CT cells. This included *RHOH* - a Rho GTPase frequently overexpressed in T-ALL[123], *NOTCH1* - a TF and prototypical T-ALL oncogene[124], and *SLC9A7 (NHE7)* - a membrane protein associated with an immature (and more aggressive) class of T-ALL[125]. The only c-Myb target gene inferred to be downregulated was *PRKCB* - a PKC kinase with reported tumor suppressive functions[126].

Supplementary References

1. Sanders, A. D. *et al.* Single-cell analysis of structural variations and complex rearrangements with tri-channel processing. *Nat. Biotechnol.* **38**, 343–354 (2020).
2. Porubský, D. *et al.* Direct chromosome-length haplotyping by single-cell sequencing. *Genome Res.* **26**, 1565–1574 (2016).
3. Mardin, B. R. *et al.* A cell-based model system links chromothripsis with hyperploidy. *Mol. Syst. Biol.* **11**, 828 (2015).
4. ENCODE Project Consortium. An integrated encyclopedia of DNA elements in the human genome. *Nature* **489**, 57–74 (2012).
5. Vainshtein, Y., Rippe, K. & Teif, V. B. NucTools: analysis of chromatin feature occupancy profiles from high-throughput sequencing data. *BMC Genomics* **18**, 158 (2017).
6. Teif, V. B. *et al.* Genome-wide nucleosome positioning during embryonic stem cell development. *Nat. Struct. Mol. Biol.* **19**, 1185–1192 (2012).
7. Gaffney, D. J. *et al.* Controls of nucleosome positioning in the human genome. *PLoS Genet.* **8**, e1003036 (2012).
8. Roadmap Epigenomics Consortium *et al.* Integrative analysis of 111 reference human epigenomes. *Nature* **518**, 317–330 (2015).
9. Wang, X. *et al.* High-resolution genome-wide functional dissection of transcriptional regulatory regions and nucleotides in human. *Nat. Commun.* **9**, 5380 (2018).
10. Satpathy, A. T. *et al.* Massively parallel single-cell chromatin landscapes of human immune cell development and intratumoral T cell exhaustion. *Nat. Biotechnol.* **37**, 925–936 (2019).
11. Robinson, M. D., McCarthy, D. J. & Smyth, G. K. edgeR: a Bioconductor package for differential expression analysis of digital gene expression data. *Bioinformatics* **26**, 139–140 (2010).
12. Rozowsky, J. *et al.* AlleleSeq: analysis of allele-specific expression and binding in a

network framework. *Mol. Syst. Biol.* **7**, 522 (2011).

13. Benjamini, Y. & Hochberg, Y. Controlling the False Discovery Rate: A Practical and Powerful Approach to Multiple Testing. *J. R. Stat. Soc. Series B Stat. Methodol.* **57**, 289–300 (1995).
14. Shen, L., Shao, N., Liu, X. & Nestler, E. ngs.plot: Quick mining and visualization of next-generation sequencing data by integrating genomic databases. *BMC Genomics* **15**, 284 (2014).
15. Lai, B. *et al.* Principles of nucleosome organization revealed by single-cell micrococcal nuclease sequencing. *Nature* **562**, 281–285 (2018).
16. Beck, S. *et al.* Implications of CpG islands on chromosomal architectures and modes of global gene regulation. *Nucleic Acids Res.* **46**, 4382–4391 (2018).
17. Schep, A. N., Wu, B., Buenrostro, J. D. & Greenleaf, W. J. chromVAR: inferring transcription-factor-associated accessibility from single-cell epigenomic data. *Nat. Methods* **14**, 975–978 (2017).
18. Weirauch, M. T. *et al.* Determination and inference of eukaryotic transcription factor sequence specificity. *Cell* **158**, 1431–1443 (2014).
19. Hansen, R. S. *et al.* Sequencing newly replicated DNA reveals widespread plasticity in human replication timing. *Proc. Natl. Acad. Sci. U. S. A.* **107**, 139–144 (2010).
20. Fenouil, R. *et al.* CpG islands and GC content dictate nucleosome depletion in a transcription-independent manner at mammalian promoters. *Genome Res.* **22**, 2399–2408 (2012).
21. Lai, W. K. M. & Pugh, B. F. Understanding nucleosome dynamics and their links to gene expression and DNA replication. *Nat. Rev. Mol. Cell Biol.* **18**, 548–562 (2017).
22. Ulz, P. *et al.* Inferring expressed genes by whole-genome sequencing of plasma DNA. *Nat. Genet.* **48**, 1273–1278 (2016).
23. Love, M. I., Huber, W. & Anders, S. Moderated estimation of fold change and dispersion for RNA-seq data with DESeq2. *Genome Biol.* **15**, 550 (2014).
24. Müller, S., Cho, A., Liu, S. J., Lim, D. A. & Diaz, A. CONICS integrates scRNA-seq with

DNA sequencing to map gene expression to tumor sub-clones. *Bioinformatics* **34**, 3217–3219 (2018).

25. Marinov, G. K. *et al.* From single-cell to cell-pool transcriptomes: stochasticity in gene expression and RNA splicing. *Genome Res.* **24**, 496–510 (2014).
26. Li, H. *et al.* Reference component analysis of single-cell transcriptomes elucidates cellular heterogeneity in human colorectal tumors. *Nat. Genet.* **49**, 708–718 (2017).
27. Kuleshov, M. V. *et al.* Enrichr: a comprehensive gene set enrichment analysis web server 2016 update. *Nucleic Acids Res.* **44**, W90–7 (2016).
28. Huang, D. W., Sherman, B. T. & Lempicki, R. A. Systematic and integrative analysis of large gene lists using DAVID bioinformatics resources. *Nat. Protoc.* **4**, 44–57 (2009).
29. Clarke, L. *et al.* The international Genome sample resource (IGSR): A worldwide collection of genome variation incorporating the 1000 Genomes Project data. *Nucleic Acids Res.* **45**, D854–D859 (2017).
30. Rausch, T. *et al.* DELLY: structural variant discovery by integrated paired-end and split-read analysis. *Bioinformatics* **28**, i333–i339 (2012).
31. Ebert, P. *et al.* Haplotype-resolved diverse human genomes and integrated analysis of structural variation. *Science* (2021) doi:10.1126/science.abf7117.
32. Ivanov, A. A. *et al.* OncoPPi-informed discovery of mitogen-activated protein kinase kinase 3 as a novel binding partner of c-Myc. *Oncogene* **36**, 5852–5860 (2017).
33. Zhang, J. *et al.* International Cancer Genome Consortium Data Portal--a one-stop shop for cancer genomics data. *Database* **2011**, bar026 (2011).
34. ICGC/TCGA Pan-Cancer Analysis of Whole Genomes Consortium. Pan-cancer analysis of whole genomes. *Nature* **578**, 82–93 (2020).
35. Edelmann, J. *et al.* High-resolution genomic profiling of chronic lymphocytic leukemia reveals new recurrent genomic alterations. *Blood* **120**, 4783–4794 (2012).
36. Puente, X. S. *et al.* Non-coding recurrent mutations in chronic lymphocytic leukaemia. *Nature* **526**, 519–524 (2015).
37. Malek, S. N. The biology and clinical significance of acquired genomic copy number

aberrations and recurrent gene mutations in chronic lymphocytic leukemia. *Oncogene* **32**, 2805–2817 (2013).

38. Edelmann, J. *et al.* Genomic alterations in high-risk chronic lymphocytic leukemia frequently affect cell cycle key regulators and NOTCH1-regulated transcription. *Haematologica* **105**, 1379–1390 (2020).
39. Ghamlouch, H., Nguyen-Khac, F. & Bernard, O. A. Chronic lymphocytic leukaemia genomics and the precision medicine era. *Br. J. Haematol.* **178**, 852–870 (2017).
40. Kaposi-Novak, P. *et al.* Met-regulated expression signature defines a subset of human hepatocellular carcinomas with poor prognosis and aggressive phenotype. *J. Clin. Invest.* **116**, 1582–1595 (2006).
41. Rodríguez, A. E. *et al.* Molecular characterization of chronic lymphocytic leukemia patients with a high number of losses in 13q14. *PLoS One* **7**, e48485 (2012).
42. Boon, E. M. J., van der Neut, R., van de Wetering, M., Clevers, H. & Pals, S. T. Wnt signaling regulates expression of the receptor tyrosine kinase met in colorectal cancer. *Cancer Res.* **62**, 5126–5128 (2002).
43. Russell, R. *et al.* Loss of ATM accelerates pancreatic cancer formation and epithelial-mesenchymal transition. *Nat. Commun.* **6**, 7677 (2015).
44. Porubsky, D. *et al.* breakpointR: an R/Bioconductor package to localize strand state changes in Strand-seq data. *Bioinformatics* **36**, 1260–1261 (2020).
45. Wilde, L., Cooper, J., Wang, Z.-X. & Liu, J. Clinical, Cytogenetic, and Molecular Findings in Two Cases of Variant t(8;21) Acute Myeloid Leukemia (AML). *Front. Oncol.* **9**, 1016 (2019).
46. Carithers, L. J. *et al.* A Novel Approach to High-Quality Postmortem Tissue Procurement: The GTEx Project. *Biopreserv. Biobank.* **13**, 311–319 (2015).
47. Nagel, S. *et al.* Activation of TLX3 and NKX2-5 in t(5;14)(q35;q32) T-cell acute lymphoblastic leukemia by remote 3’-BCL11B enhancers and coregulation by PU.1 and HMGA1. *Cancer Res.* **67**, 1461–1471 (2007).
48. McKenna, A. *et al.* The Genome Analysis Toolkit: a MapReduce framework for

analyzing next-generation DNA sequencing data. *Genome Res.* **20**, 1297–1303 (2010).

49. Porubsky, D. *et al.* Dense and accurate whole-chromosome haplotyping of individual genomes. *Nat. Commun.* **8**, 1293 (2017).
50. Patel, A. P. *et al.* Single-cell RNA-seq highlights intratumoral heterogeneity in primary glioblastoma. *Science* **344**, 1396–1401 (2014).
51. Park, J.-E. *et al.* A cell atlas of human thymic development defines T cell repertoire formation. *Science* **367**, (2020).
52. 1000 Genomes Project Consortium *et al.* A global reference for human genetic variation. *Nature* **526**, 68–74 (2015).
53. PCAWG Transcriptome Core Group *et al.* Genomic basis for RNA alterations in cancer. *Nature* **578**, 129–136 (2020).
54. Hay, S. B., Ferchen, K., Chetal, K., Grimes, H. L. & Salomonis, N. The Human Cell Atlas bone marrow single-cell interactive web portal. *Exp. Hematol.* **68**, 51–61 (2018).
55. McCarthy, B. A. *et al.* A seven-gene expression panel distinguishing clonal expansions of pre-leukemic and chronic lymphocytic leukemia B cells from normal B lymphocytes. *Immunol. Res.* **63**, 90–100 (2015).
56. Garcia-Alonso, L., Holland, C. H., Ibrahim, M. M., Turei, D. & Saez-Rodriguez, J. Benchmark and integration of resources for the estimation of human transcription factor activities. *Genome Res.* **29**, 1363–1375 (2019).
57. Kamburov, A. & Herwig, R. ConsensusPathDB 2022: molecular interactions update as a resource for network biology. *Nucleic Acids Res.* **50**, D587–D595 (2022).
58. Liu, S., Thennavan, A., Garay, J. P., Marron, J. S. & Perou, C. M. MultiK: an automated tool to determine optimal cluster numbers in single-cell RNA sequencing data. *Genome Biol.* **22**, 232 (2021).
59. Hanlon, V. C. T. *et al.* Construction of Strand-seq libraries in open nanoliter arrays. *Cell Rep Methods* **2**, 100150 (2022).
60. Porubsky, D. *et al.* Recurrent inversion polymorphisms in humans associate with genetic instability and genomic disorders. *Cell* (2022) doi:10.1016/j.cell.2022.04.017.

61. Cicirò, Y. & Sala, A. MYB oncoproteins: emerging players and potential therapeutic targets in human cancer. *Oncogenesis* **10**, 19 (2021).

62. Pattabiraman, D. R. & Gonda, T. J. Role and potential for therapeutic targeting of MYB in leukemia. *Leukemia* **27**, 269–277 (2013).

63. Liu, X., Xu, Y., Han, L. & Yi, Y. Reassessing the Potential of Myb-targeted Anti-cancer Therapy. *J. Cancer* **9**, 1259–1266 (2018).

64. Majumder, S. *et al.* Targeting Notch in oncology: the path forward. *Nat. Rev. Drug Discov.* **20**, 125–144 (2021).

65. Study of CB-103 in Adult Patients With Advanced or Metastatic Solid Tumours and Haematological Malignancies. https://clinicaltrials.gov/ct2/show/NCT03422679.

66. Lehal, R. *et al.* Pharmacological disruption of the Notch transcription factor complex. *Proc. Natl. Acad. Sci. U. S. A.* **117**, 16292–16301 (2020).

67. Langmead, B., Trapnell, C., Pop, M. & Salzberg, S. L. Ultrafast and memory-efficient alignment of short DNA sequences to the human genome. *Genome Biol.* **10**, R25 (2009).

68. Chen, K. *et al.* DANPOS: dynamic analysis of nucleosome position and occupancy by sequencing. *Genome Res.* **23**, 341–351 (2013).

69. Li, H. & Durbin, R. Fast and accurate short read alignment with Burrows-Wheeler transform. *Bioinformatics* **25**, 1754–1760 (2009).

70. Li, H. *et al.* The Sequence Alignment/Map format and SAMtools. *Bioinformatics* **25**, 2078–2079 (2009).

71. Buenrostro, J. D., Giresi, P. G., Zaba, L. C., Chang, H. Y. & Greenleaf, W. J. Transposition of native chromatin for fast and sensitive epigenomic profiling of open chromatin, DNA-binding proteins and nucleosome position. *Nat. Methods* **10**, 1213–1218 (2013).

72. Huang, X., Du, X. & Li, Y. The role of BCL11B in hematological malignancy. *Exp. Hematol. Oncol.* **1**, 22 (2012).

73. Li, L. *et al.* A far downstream enhancer for murine Bcl11b controls its T-cell specific

expression. *Blood* **122**, 902–911 (2013).

74. Claringbould, A. & Zaugg, J. B. Enhancers in disease: molecular basis and emerging treatment strategies. *Trends Mol. Med.* **27**, 1060–1073 (2021).

75. Gutierrez, A. *et al.* The BCL11B tumor suppressor is mutated across the major molecular subtypes of T-cell acute lymphoblastic leukemia. *Blood* **118**, 4169–4173 (2011).

76. Erarslan-Uysal, B. *et al.* Chromatin accessibility landscape of pediatric T-lymphoblastic leukemia and human T-cell precursors. *EMBO Mol. Med.* e12104 (2020).

77. Feng, J., Liu, T., Qin, B., Zhang, Y. & Liu, X. S. Identifying ChIP-seq enrichment using MACS. *Nat. Protoc.* **7**, 1728–1740 (2012).

78. Jin, W. *et al.* TrkC plays an essential role in breast tumor growth and metastasis. *Carcinogenesis* **31**, 1939–1947 (2010).

79. Van Rechem, C. *et al.* Collective regulation of chromatin modifications predicts replication timing during cell cycle. *Cell Rep.* **37**, 109799 (2021).

80. Dobin, A. *et al.* STAR: ultrafast universal RNA-seq aligner. *Bioinformatics* **29**, 15–21 (2013).

81. Anders, S., Pyl, P. T. & Huber, W. HTSeq--a Python framework to work with high-throughput sequencing data. *Bioinformatics* **31**, 166–169 (2015).

82. Wu, T. D. & Nacu, S. Fast and SNP-tolerant detection of complex variants and splicing in short reads. *Bioinformatics* **26**, 873–881 (2010).

83. Stamoulis, G. *et al.* Single cell transcriptome in aneuploidies reveals mechanisms of gene dosage imbalance. *Nat. Commun.* **10**, 4495 (2019).

84. Richter-Pechańska, P. *et al.* PDX models recapitulate the genetic and epigenetic landscape of pediatric T-cell leukemia. *EMBO Mol. Med.* **10**, (2018).

85. Campo, E. *et al.* TP53 aberrations in chronic lymphocytic leukemia: an overview of the clinical implications of improved diagnostics. *Haematologica* **103**, 1956–1968 (2018).

86. Wellenstein, M. D. *et al.* Loss of p53 triggers WNT-dependent systemic inflammation to drive breast cancer metastasis. *Nature* **572**, 538–542 (2019).

87. Lee, K.-H. *et al.* A genomewide study identifies the Wnt signaling pathway as a major target of p53 in murine embryonic stem cells. *Proc. Natl. Acad. Sci. U. S. A.* **107**, 69–74 (2010).

88. Abruzzo, L. V. *et al.* Trisomy 12 chronic lymphocytic leukemia expresses a unique set of activated and targetable pathways. *Haematologica* **103**, 2069–2078 (2018).

89. Han, H. *et al.* TRRUST v2: an expanded reference database of human and mouse transcriptional regulatory interactions. *Nucleic Acids Res.* **46**, D380–D386 (2018).

90. Liberzon, A. *et al.* Molecular signatures database (MSigDB) 3.0. *Bioinformatics* **27**, 1739–1740 (2011).

91. Garrison, E. & Marth, G. Haplotype-based variant detection from short-read sequencing. *arXiv [q-bio.GN]* (2012).

92. Sanders, A. D., Falconer, E., Hills, M., Spierings, D. C. J. & Lansdorp, P. M. Single-cell template strand sequencing by Strand-seq enables the characterization of individual homologs. *Nat. Protoc.* **12**, 1151–1176 (2017).

93. Byrska-Bishop, M. *et al.* High coverage whole genome sequencing of the expanded 1000 Genomes Project cohort including 602 trios. *Cold Spring Harbor Laboratory* 2021.02.06.430068 (2021) doi:10.1101/2021.02.06.430068.

94. Loh, P.-R. *et al.* Reference-based phasing using the Haplotype Reference Consortium panel. *Nat. Genet.* **48**, 1443–1448 (2016).

95. Zook, J. M. *et al.* An open resource for accurately benchmarking small variant and reference calls. *Nat. Biotechnol.* **37**, 561–566 (2019).

96. Fan, J. *et al.* Linking transcriptional and genetic tumor heterogeneity through allele analysis of single-cell RNA-seq data. *Genome Res.* **28**, 1217–1227 (2018).

97. Street, K. *et al.* Slingshot: cell lineage and pseudotime inference for single-cell transcriptomics. *BMC Genomics* **19**, 477 (2018).

98. Wang, K., Wei, G. & Liu, D. CD19: a biomarker for B cell development, lymphoma diagnosis and therapy. *Exp. Hematol. Oncol.* **1**, 36 (2012).

99. Alvarez, M. J. *et al.* Functional characterization of somatic mutations in cancer using

network-based inference of protein activity. *Nat. Genet.* **48**, 838–847 (2016).

100. Andreatta, M. & Carmona, S. J. UCell: Robust and scalable single-cell gene signature scoring. *Comput. Struct. Biotechnol. J.* **19**, 3796–3798 (2021).

101. Fromigué, O., Haÿ, E., Barbara, A. & Marie, P. J. Essential role of nuclear factor of activated T cells (NFAT)-mediated Wnt signaling in osteoblast differentiation induced by strontium ranelate. *J. Biol. Chem.* **285**, 25251–25258 (2010).

102. Li, L. *et al.* B-cell receptor-mediated NFATc1 activation induces IL-10/STAT3/PD-L1 signaling in diffuse large B-cell lymphoma. *Blood* **132**, 1805–1817 (2018).

103. Oestreich, K. J., Yoon, H., Ahmed, R. & Boss, J. M. NFATc1 regulates PD-1 expression upon T cell activation. *J. Immunol.* **181**, 4832–4839 (2008).

104. Böttcher, M. *et al.* Control of PD-L1 expression in CLL-cells by stromal triggering of the Notch-c-Myc-EZH2 oncogenic signaling axis. *J Immunother Cancer* **9**, (2021).

105. Sabarinathan, R. *et al.* The whole-genome panorama of cancer drivers. *bioRxiv* 190330 (2017) doi:10.1101/190330.

106. Liu, L.-J. *et al.* Aberrant regulation of Wnt signaling in hepatocellular carcinoma. *World J. Gastroenterol.* **22**, 7486–7499 (2016).

107. Wang, Z., Liu, P., Inuzuka, H. & Wei, W. Roles of F-box proteins in cancer. *Nat. Rev. Cancer* **14**, 233–247 (2014).

108. Lockwood, W. W., Chandel, S. K., Stewart, G. L., Erdjument-Bromage, H. & Beverly, L. J. The novel ubiquitin ligase complex, SCF(Fbxw4), interacts with the COP9 signalosome in an F-box dependent manner, is mutated, lost and under-expressed in human cancers. *PLoS One* **8**, e63610 (2013).

109. Ianakiev, P. *et al.* A novel human gene encoding an F-box/WD40 containing protein maps in the SHFM3 critical region on 10q24. *Biochem. Biophys. Res. Commun.* **261**, 64–70 (1999).

110. Meng, X. *et al.* Suppressor of fused negatively regulates beta-catenin signaling. *J. Biol. Chem.* **276**, 40113–40119 (2001).

111. Taylor, M. D. *et al.* Failure of a medulloblastoma-derived mutant of SUFU to suppress

WNT signaling. *Oncogene* **23**, 4577–4583 (2004).

112. Johnson, D. T. *et al.* Deletion of leucine zipper tumor suppressor 2 (Lzts2) increases susceptibility to tumor development. *J. Biol. Chem.* **288**, 3727–3738 (2013).

113. Bassaganyas, L. *et al.* Sporadic and reversible chromothripsis in chronic lymphocytic leukemia revealed by longitudinal genomic analysis. *Leukemia* **27**, 2376–2379 (2013).

114. Stevens-Kroef, M. J. *et al.* Identification of prognostic relevant chromosomal abnormalities in chronic lymphocytic leukemia using microarray-based genomic profiling. *Mol. Cytogenet.* **7**, 3 (2014).

115. Gerondakis, S. & Siebenlist, U. Roles of the NF-kappaB pathway in lymphocyte development and function. *Cold Spring Harb. Perspect. Biol.* **2**, a000182 (2010).

116. Ma, B. & Hottiger, M. O. Crosstalk between Wnt/β-Catenin and NF-κB Signaling Pathway during Inflammation. *Front. Immunol.* **7**, 378 (2016).

117. Ahmed, S., Pryce, B. R., Al-Zahrani, K. N. & Sabourin, L. A. The LIM domain binding protein 1, Ldb1, has distinct roles in Neu-induced mammary tumorigenesis. *Biochim. Biophys. Acta Mol. Cell Res.* **1865**, 1590–1597 (2018).

118. Kim, T. W. *et al.* Ctbp2-mediated β-catenin regulation is required for exit from pluripotency. *Exp. Mol. Med.* **49**, e385 (2017).

119. Persad, A. *et al.* Active β-catenin is regulated by the PTEN/PI3 kinase pathway: a role for protein phosphatase PP2A. *Genes Cancer* **7**, 368–382 (2016).

120. Collins, A. M. & Watson, C. T. Immunoglobulin Light Chain Gene Rearrangements, Receptor Editing and the Development of a Self-Tolerant Antibody Repertoire. *Front. Immunol.* **9**, 2249 (2018).

121. Ding, L. *et al.* A novel stromal lncRNA signature reprograms fibroblasts to promote the growth of oral squamous cell carcinoma via LncRNA-CAF/interleukin-33. *Carcinogenesis* **39**, 397–406 (2018).

122. Aganezov, S. *et al.* A complete reference genome improves analysis of human genetic variation. *Science* **376**, eabl3533 (2022).

123. Bhavsar, P. J., Infante, E., Khwaja, A. & Ridley, A. J. Analysis of Rho GTPase

expression in T-ALL identifies RhoU as a target for Notch involved in T-ALL cell migration. *Oncogene* **32**, 198–208 (2013).

124. Ferrando, A. A. The role of NOTCH1 signaling in T-ALL. *Hematology Am. Soc. Hematol. Educ. Program* 353–361 (2009).

125. Wang, M. & Zhang, C. Low LEF1 expression is a biomarker of early T-cell precursor, an aggressive subtype of T-cell lymphoblastic leukemia. *PLoS One* **15**, e0232520 (2020).

126. Antal, C. E. *et al.* Cancer-associated protein kinase C mutations reveal kinase's role as tumor suppressor. *Cell* **160**, 489–502 (2015).
