# Supplementary material for: Functional analysis of structural variants in single cells using Strand-seq
Source: Nat Biotechnol. 2022 Nov 24;41(6):832–44. doi: 10.1038/s41587-022-01551-4 (PMC10264249; doi:10.1038/s41587-022-01551-4)
Supplement: Supplementary file 2 — Reporting Summary [file 41587_2022_1551_MOESM2_ESM.pdf]

nature portfolio

Corresponding author(s): Ashley D. Sanders & Jan O. Korbel

Last updated by author(s): Sep 29, 2022

Reporting Summary

Nature Portfolio wishes to improve the reproducibility of the work that we publish. This form provides structure for consistency and transparency in reporting. For further information on Nature Portfolio policies, see our Editorial Policies and the Editorial Policy Checklist.

Statistics

For all statistical analyses, confirm that the following items are present in the figure legend, table legend, main text, or Methods section.

n/a	Confirmed	
☐	☒	The exact sample size (n) for each experimental group/condition, given as a discrete number and unit of measurement
☐	☒	A statement on whether measurements were taken from distinct samples or whether the same sample was measured repeatedly
☐	☒	The statistical test(s) used AND whether they are one- or two-sided<br>*Only common tests should be described solely by name; describe more complex techniques in the Methods section.*
☒	☐	A description of all covariates tested
☐	☒	A description of any assumptions or corrections, such as tests of normality and adjustment for multiple comparisons
☐	☒	A full description of the statistical parameters including central tendency (e.g. means) or other basic estimates (e.g. regression coefficient) AND variation (e.g. standard deviation) or associated estimates of uncertainty (e.g. confidence intervals)
☐	☒	For null hypothesis testing, the test statistic (e.g. F, t, r) with confidence intervals, effect sizes, degrees of freedom and P value noted<br>*Give P values as exact values whenever suitable.*
☒	☐	For Bayesian analysis, information on the choice of priors and Markov chain Monte Carlo settings
☒	☐	For hierarchical and complex designs, identification of the appropriate level for tests and full reporting of outcomes
☐	☒	Estimates of effect sizes (e.g. Cohen's d, Pearson's r), indicating how they were calculated

Our web collection on statistics for biologists contains articles on many of the points above.

Software and code

Policy information about availability of computer code

Data collection	Our study uses sequencing data output by illumina sequencers and hence no special software was used for collecting it.
Data analysis	The computational code of our analytical framework is hosted on GitHub (see https://github.com/jeongdo801/scNOVA). All code is available freely for academic research.<br>Other software used:<br>Mosaicatcher (https://github.com/friendsofstrandseq/mosaicatcher-pipeline), StrandPhaseR (https://github.com/daewoooo/StrandPhaseR), InferCNV (https://github.com/broadinstitute/inferCNV/), HoneyBADGER (https://jef.works/HoneyBADGER/), CONICSmat (https://github.com/diazlab/CONICS), NucTools (https://homeveg.github.io/nuctools), Delly2 (https://github.com/dellytools/delly), BWA (v0.7.15), STAR (v2.7.9a), SAMtools (v1.3.1), biobambam2 (v2.0.76), deeptools (v2.5.1), perl (v5.16.3), Python (v3.7.4), cuDNN (v7.6.4.38), CUDA (v10.1.243), TensorFlow (v1.15.0), scikit-learn (v0.21.3), matplotlib (v3.1.1), R version 4.0.0, DESeq2, FlowJo, BD FACSDiva™

For manuscripts utilizing custom algorithms or software that are central to the research but not yet described in published literature, software must be made available to editors and reviewers. We strongly encourage code deposition in a community repository (e.g. GitHub). See the Nature Portfolio guidelines for submitting code & software for further information.

Data

Policy information about availability of data

All manuscripts must include a data availability statement. This statement should provide the following information, where applicable:

- Accession codes, unique identifiers, or web links for publicly available datasets
- A description of any restrictions on data availability
- For clinical datasets or third party data, please ensure that the statement adheres to our policy

Sequencing data from this study can be retrieved from the European Genome-phenome Archive (EGA) , and the European Nucleotide Archive (ENA) [accessions: LCL data are available under the following accessions: Strand-seq (PRJEB39750, PRJEB55038); RNA-seq (ERP123231); WGS (PRJEB37677). C11 cell line data are available under the accession PRJEB55012. Leukemia patient data and human primary cells derived data were deposited in the European Genome-phenome Archive (EGA), under the following accession numbers: skin fibroblast (EGAS00001006498); cord blood (EGAS00001006567). T-ALL Strand-seq and scRNA-seq (EGAS00001003365), CLL Strand-seq (EGAS00001004925), AML Strand-seq (EGAS00001004903), T-ALL bulk RNA-seq (EGAS00001003248), CLL bulk RNA-seq (EGAS00001005746), CLL CITE-seq (EGAS00001004925).] Access to human patient data is governed by the EGA Data Access Committee.

Field-specific reporting

Please select the one below that is the best fit for your research. If you are not sure, read the appropriate sections before making your selection.

☒ Life sciences ☐ Behavioural & social sciences ☐ Ecological, evolutionary & environmental sciences

For a reference copy of the document with all sections, see nature.com/documents/nr-reporting-summary-flat.pdf

Life sciences study design

All studies must disclose on these points even when the disclosure is negative.

Sample size	No sample-size calculation was performed, since this study focuses on establishing a novel methodology rather than on performing statistical tests between groups of samples.
Data exclusions	We excluded low quality single-cell libraries that showed very low, uneven coverage, or an excess of 'background reads' yielding noisy single cell data prior to analysis. Cells with incomplete BrdU incorporation or cells undergoing more than one DNA synthesis phase under BrdU exposure are largely excluded during cell sorting and thus get only rarely sequenced during Strand-seq experiments.
Replication	To ensure reproducibility of our computational findings we have organized our main workflow using Snakemake, a widely used workflow manager, and we provide the workflow description (Snakefile) along with a Bioconda environment that facilitates easy installation of all dependeencies (with well-defined versions). We have repeated the analyses of our datasets and can confirm consistent and reproducible results from these workflows. To ensure reproducibility of our experimental findings, we generated replicates wherever possible, which confirmed reproducibility of the result.
Randomization	Does not apply (there are no experimental groups in our study)
Blinding	Does not apply. (this study focuses on intra-sample comparison rather than performing statistical tests between groups of samples).

Reporting for specific materials, systems and methods

We require information from authors about some types of materials, experimental systems and methods used in many studies. Here, indicate whether each material, system or method listed is relevant to your study. If you are not sure if a list item applies to your research, read the appropriate section before selecting a response.

Materials & experimental systems

n/a	Involved in the study	
☐	☒	Antibodies
☐	☒	Eukaryotic cell lines
☒	☐	Palaeontology and archaeology
☒	☐	Animals and other organisms
☐	☒	Human research participants
☐	☒	Clinical data
☒	☐	Dual use research of concern

Methods

n/a	Involved in the study	
☒	☐	ChIP-seq
☐	☒	Flow cytometry
☒	☐	MRI-based neuroimaging

Antibodies

Antibodies used	FACS (clone, manufacturer, catalogue number, lot number): APC mouse anti-human CD34 (clone 581; Biolegend; #343509; Lot:

Antibodies used

B260867), PeCy7 mouse anti-human CD38 (clone HB7; eBioscience; #15538396; Lot: 1974952), FITC mouse anti-human CD45Ra (clone HI100; eBioscience; #15526406; Lot: 4329359), PE mouse anti-human CD90 (clone 5E10; eBioscience; # 15526836; Lot:1982684), PE anti-murine CD45 (clone 30-F11; Biolegend; #103106; Lot: B361031). CITE-seq (clone, manufacturer, catalogue number, barcode number): CD10 (HI10a; Biolegend; 312231; 0062), CD103 (Ber-ACT8 ; Biolegend; 350231; 0145), CD11b (ICRF44; Biolegend; 301353; 0161), CD11c (S-HCL-3; Biolegend; 371519; 0053), CD127 (A019D5; Biolegend; 351352; 0390), CD134 (Ber-ACT35; Biolegend; 350033; 0158), CD137 (4B4-1; Biolegend; 309835; 0355), CD150 (A12 (7D4); Biolegend; 306313; 0870), CD152 (BNI3; Biolegend; 369619; 0151), CD16 (3G8; Biolegend; 302061; 0083), CD161 (HP-3G10; Biolegend; 339945; 0149), CD183 (G025H7; Biolegend; 353745; 0140), CD184 (12G5; Biolegend; 306531; 0366), CD185 (J252D4; Biolegend; 356937; 0144), CD19 (HIB19; Biolegend; 302259; 0050), CD194 (L291H4; Biolegend; 359423; 0071), CD195 (J418F1; Biolegend; 359135; 0141), CD197 (G043H7; Biolegend; 353247; 0148), CD2 (TS1/8; Biolegend; 309229; 0367), CD20 (2H7; Biolegend; 302359; 0100), CD21 (Bu32; Biolegend; 354915; 0181), CD22 (S-HCL-1; Biolegend; 363514; 0393), CD223 (11C3C65; Biolegend; 369333; 0152), CD23 (EBVCS-5; Biolegend; 338523; 0897), CD24 (ML5; Biolegend; 311137; 0180), CD244 (C1.7; Biolegend; 329527; 0189), CD25 (BC96; Biolegend; 302643; 0085), CD27 (O323; Biolegend; 302847; 0154), CD273 (24F.10C12; Biolegend; 329619; 0008), CD274 (29E.2A3; Biolegend; 329743; 0007), CD278 (C398.4A; Biolegend; 313555; 0171), CD279 (EH12.2H7; Biolegend; 329955; 0088), CD28 (CD28.2; Biolegend; 302955; 0386), CD29 (TS2/16; Biolegend; 303027; 0369), CD3 (UCHT1; Biolegend; 300475; 0034), CD31 (WM59; Biolegend; 303137; 0124), CD32 (FUN-2; Biolegend; 303223; 0142), CD357 (108-17; Biolegend; 371225; 0360), CD366 (F38-2E2; Biolegend; 345047; 0169), CD38 (HIT2; Biolegend; 303541; 0389), CD39 (A1; Biolegend; 328233; 0176), CD4 (RPA-T4; Biolegend; 300563; 0072), CD43 (CD43-10G7; Biolegend; 343209; 0357), CD44 (IM7; Biolegend; 103045; 0073), CD45 (HI30; Biolegend; 304064; 0391), CD45RA (HI100; Biolegend; 304157; 0063), CD45RO (UCHL1; Biolegend; 304255; 0087), CD47 (CC2C6; Biolegend; 323129; 0026), CD48 (BJ40; Biolegend; 336709; 0029), CD5 (UCHT2; Biolegend; 300635; 0138), CD56 (QA17A16; Biolegend; 392421; 0084), CD57 (QA17A04; Biolegend; 393319; 0168), CD62L (DREG-56; Biolegend; 304847; 0147), CD69 (FN50; Biolegend; 310947; 0146), CD7 (CD7-6B7; Biolegend; 343123; 0066), CD70 (113-16; Biolegend; 355117; 0027), CD73 (AD2; Biolegend; 344029; 0577), CD79b (CB3-1; Biolegend; 341415; 0187), CD86 (IT2.2; Biolegend; 305443; 0006), CD8a (RPA-T8; Biolegend; 301067; 0080), CD95 (DX2; Biolegend; 305649; 0156), Kappa (MHK-49 ; Biolegend; 316531; 0894), KLRG1 (SA231A2; Biolegend; 367721; 0153), Lambda (MHL-38 ; Biolegend; 316627; 0898), TIGIT (A15153G; Biolegend; 372725; 0089), Isotype Ctrl (MOPC-21; Biolegend; 400199; 0090), Isotype Ctrl (HTK888; Biolegend; 400973; 0241), Isotype Ctrl (MPC-11; Biolegend; 400373; 0092), Isotype Ctrl (RTK4530; Biolegend; 400673; 0095), Isotype Ctrl (MOPC-173; Biolegend; 400285; 0091)

Validation

All antibodies were validated for the specific application by the manufacturer and validation data is available on the manufacturer's website.
FACS
CD34 CD34 https://www.biolegend.com/fr-ch/products/apc-anti-human-cd34-antibody-6090 DOI: 10.1538/expanim.49.97
CD38 CD38 https://www.thermofisher.com/antibody/product/CD38-Antibody-clone-HB7-Monoclonal/25-0388-42 DOI: 10.1016/j.stem.2021.02.001
CD45Ra PTPRC https://www.thermofisher.com/antibody/product/CD45RA-Antibody-clone-HI100-Monoclonal/14-0458-82 DOI: 10.1080/2162402X.2017.1371399
CD90 THY1 https://www.thermofisher.com/antibody/product/CD90-Thy-1-Antibody-clone-eBio5E10-5E10-Monoclonal/12-0909-42 DOI: 10.1186/s41232-017-0049-2
CD45 PTPRC https://www.biolegend.com/fr-fr/products/pe-anti-mouse-cd45-antibody-100 DOI: 10.4049/jimmunol.176.11.6532
CITE-seq
CD10 MME https://www.biolegend.com/en-us/search-results/totalseq-a0062-anti-human-cd10-antibody-15949?GroupID=BLG5905 doi: 10.1084/jem.181.6.2271
CD103 ITGAE https://www.biolegend.com/nl-be/products/totalseq-a0145-anti-human-cd103-integrin-%CE%B1e-antibody-16194 doi: 10.1538/expanim.49.97
CD11b ITGAM https://www.biolegend.com/en-us/products/totalseq-a0161-anti-human-cd11b-antibody-15927 doi: 10.4049/jimmunol.1302846
CD11c ITGAX https://www.biolegend.com/de-de/products/totalseq-a0053-anti-human-cd11c-antibody-16231 doi: 10.1016/j.jri.2011.01.014
CD127 IL7R https://www.biolegend.com/en-us/search-results/totalseq-a0390-anti-human-cd127-il-7ralpha-antibody-15943?GroupID=BLG9274 doi: 10.1038/nbt.3973
CD134 TNFRSF4 https://www.biolegend.com/en-us/products/totalseq-a0158-anti-human-cd134-ox40-antibody-16437 doi: 10.2215/CJN.06460612
CD137 TNFRSF9 https://www.biolegend.com/en-us/products/totalseq-trade-a0355-anti-human-cd137-4-1bb-antibody-16737 doi: 10.4049/jimmunol.165.5.2903
CD150 SLAMF1 https://www.biolegend.com/fr-ch/search-results/totalseq-a0870-anti-human-cd150-slam-antibody-18039 doi: 10.1016/j.cell.2021.12.018
CD152 CTLA4 https://www.biolegend.com/de-at/products/totalseq-a0151-anti-human-cd152-ctla-4-antibody-15707 doi: 10.1084/jem.176.6.1595
CD16 FCGR3A https://www.biolegend.com/de-de/products/totalseq-a0083-anti-human-cd16-antibody-15765 doi: 10.1189/jlb.0408244
CD161 KLRB1 https://www.biolegend.com/nl-be/products/totalseq-a0149-anti-human-cd161-antibody-16156 doi: 10.1084/jem.188.5.867
CD183 CXCR3 https://www.biolegend.com/nl-nl/search-results/totalseq-a0140-anti-human-cd183-cxcr3-antibody-16163 DOI: 10.1016/j.cell.2021.12.018
CD184 CXCR4 https://www.biolegend.com/en-gb/products/totalseq-a0366-anti-human-cd184-cxcr4-antibody-17277 DOI: 10.1074/jbc.M610931200
CD185 CXCR5 https://www.biolegend.com/ja-jp/products/totalseq-a0144-anti-human-cd185-cxcr5-antibody-16330 DOI: 10.7554/eLife.63632
CD19 CD19 https://www.biolegend.com/nl-nl/products/totalseq-a0050-anti-human-cd19-antibody-15777 DOI: 10.3324/haematol.2009.013151
CD194 CCR4 https://www.biolegend.com/de-de/products/totalseq-a0071-anti-human-cd194-ccr4-antibody-16170 doi: 10.1016/j.xpro.2021.100900
CD195 CCR5 https://www.biolegend.com/en-us/search-results/totalseq-a0141-anti-human-cd195-ccr5-antibody-16161 DOI: 10.1016/j.cell.2021.12.018
CD197 CCR7 https://www.biolegend.com/en-gb/search-results/totalseq-a0148-anti-human-cd197-ccr7-antibody-16352?GroupID=BLG9613 DOI: 10.1016/j.cell.2019.05.031

CD2 CD2 https://www.biolegend.com/de-de/clone-search/totalseq-a0367-anti-human-cd2-antibody-16714 DOI:https://doi.org/10.1074/jbc.271.10.5369
CD20 MS4A1 https://www.biolegend.com/ja-jp/products/totalseq-a0100-anti-human-cd20-antibody-16173 DOI: 10.1203/01.PDR.0000130480.51066.FB
CD21 CR2 https://www.biolegend.com/fr-ch/products/totalseq-a0181-anti-human-cd21-antibody-16203?GroupID=ImportedGROUP1 DOI: 10.1002/eji.1830260714
CD22 CD22 https://www.biolegend.com/en-us/products/totalseq-a0393-anti-human-cd22-antibody-15936 DOI: 10.1016/j.coi.2005.03.005
CD223 LAG3 https://www.biolegend.com/fr-ch/products/totalseq-a0152-anti-human-cd223-lag-3-antibody-16157?GroupID=BLG14890 DOI: 10.1016/j.cell.2021.12.018
CD23 FCER2 https://www.biolegend.com/en-us/search-results/totalseq-a0897-anti-human-cd23-antibody-18091 DOI: 10.1128/JVI.46.3.800-807.1983
CD24 CD24 https://www.biolegend.com/en-us/products/totalseq-a0180-anti-human-cd24-antibody-16331 PMID: 14581365
CD244 CD244 https://www.biolegend.com/en-us/search-results/totalseq-a0189-anti-human-cd244-2b4-antibody-16196?GroupID=BLG8490 DOI: 10.1182/blood-2011-02-339135
CD25 IL2RA https://www.biolegend.com/fr-lu/products/totalseq-a0085-anti-human-cd25-antibody-15770 DOI: 10.1016/j.cell.2019.05.031
CD27 CD27 https://www.biolegend.com/ja-jp/products/totalseq-a0154-anti-human-cd27-antibody-16174 DOI: 10.1016/j.cell.2019.05.031
CD273 PDCD1LG2 https://www.biolegend.com/de-de/products/totalseq-a0008-anti-human-cd273-b7-dc-pd-l2-antibody-15932 DOI: 10.4049/jimmunol.170.3.1257
CD274 PDCD1LG1 https://www.biolegend.com/de-at/products/totalseq-a0007-anti-human-cd274-b7-h1-pd-l1-antibody-16195?GroupID=BLG5404 DOI: 10.4049/jimmunol.170.3.1257
CD278 ICOS https://www.biolegend.com/en-gb/products/totalseq-a0171-anti-human-mouse-rat-cd278-icos-antibody-17152 DOI: 10.4049/jimmunol.171.2.783
CD279 PDCD1 https://www.biolegend.com/de-at/products/totalseq-a0088-anti-human-cd279-pd-1-antibody-15772 DOI: 10.4049/jimmunol.181.10.6707
CD28 CD28 https://www.biolegend.com/de-de/products/totalseq-a0386-anti-human-cd28-antibody-16787 DOI: 10.1016/j.febslet.2006.11.044
CD29 ITGB1 https://www.biolegend.com/en-us/products/totalseq-a0369-anti-human-cd29-antibody-16664?GroupID=BLG10310 DOI: 10.1182/blood-2004-07-2598
CD3 CD3E https://www.biolegend.com/de-at/products/totalseq-a0034-anti-human-cd3-antibody-15707 DOI: 10.4049/jimmunol.180.11.7431
CD31 PECAM1 https://www.biolegend.com/en-gb/products/totalseq-a0124-anti-human-cd31-antibody-16332 DOI: 10.1182/blood-2006-10-047092
CD32 FCGR2A https://www.biolegend.com/en-us/products/totalseq-a0142-anti-human-cd32-antibody-16168 DOI: 10.1182/blood-2010-11-316158
CD357 TNFRSF18 https://www.biolegend.com/fr-lu/products/totalseq-a0360-anti-human-cd357-gitr-antibody-17349?GroupID=BLG15183 DOI: 10.1016/j.cell.2021.12.018
CD366 HAVCR2 https://www.biolegend.com/fr-fr/products/totalseq-a0169-anti-human-cd366-tim-3-antibody-17350 DOI: 10.1182/blood-2008-02-142596
CD38 CD38 https://www.biolegend.com/fr-fr/products/totalseq-a0389-anti-human-cd38-antibody-16899 DOI: 10.1538/expanim.49.97
CD39 ENTPD1 https://www.biolegend.com/it-it/products/totalseq-a0176-anti-human-cd39-antibody-16204 DOI: 10.7554/eLife.63632
CD4 CD4 https://www.biolegend.com/fr-ch/products/totalseq-a0072-anti-human-cd4-antibody-15762 DOI: 10.7554/eLife.63632
CD43 SPN https://www.biolegend.com/fr-ch/products/totalseq-a0357-anti-human-cd43-antibody-17546 PMID: 7507092
CD44 CD44 https://www.biolegend.com/nl-nl/products/totalseq-a0073-anti-mouse-human-cd44-antibody-15923 DOI: 10.1186/1479-5876-7-89
CD45 PTPRC https://www.biolegend.com/en-gb/search-results/totalseq-a0391-anti-human-cd45-antibody-15934?GroupID=GROUP658 DOI: 10.1038/emboj.2012.192
CD45RA PTPRC https://www.biolegend.com/it-it/products/totalseq-a0063-anti-human-cd45ra-antibody-15775 DOI: 10.4049/jimmunol.0901967
CD45RO PTPRC https://www.biolegend.com/nl-nl/products/totalseq-a0087-anti-human-cd45ro-antibody-15771 DOI: 10.4049/jimmunol.180.11.7431
CD47 CD47 https://www.biolegend.com/de-at/products/totalseq-a0026-anti-human-cd47-antibody-15957 PMID: 10572074
CD48 CD48 https://www.biolegend.com/en-gb/products/totalseq-a0029-anti-human-cd48-antibody-15942 DOI: 10.1189/jlb.0611308
CD5 CD5 https://www.biolegend.com/en-gb/clone-search/totalseq-a0138-anti-human-cd5-16333?GroupID=BLG5902 DOI: 10.1073/pnas.1001515107
CD56 NCAM1 https://www.biolegend.com/en-us/products/totalseq-a0084-anti-human-cd56-recombinant-antibody-15766?GroupID=GROUP28 DOI: 10.1186/s40364-020-00253-w
CD57 B3GAT1 https://www.biolegend.com/fr-fr/products/totalseq-a0168-anti-human-cd57-recombinant-antibody-17680 DOI: 10.1016/j.xpro.2021.100900
CD62L SELL https://www.biolegend.com/de-at/products/totalseq-a0147-anti-human-cd62l-antibody-16334 DOI: 10.4049/jimmunol.181.9.6563
CD69 CD69 https://www.biolegend.com/de-at/products/totalseq-a0146-anti-human-cd69-antibody-16200 DOI: 10.1093/toxsci/kfp224
CD7 CD7 https://www.biolegend.com/fr-fr/products/totalseq-a0066-anti-human-cd7-antibody-15944 DOI: 10.1038/nbt.3973
CD70 CD70 https://www.biolegend.com/it-it/products/totalseq-a0027-anti-human-cd70-antibody-16184 DOI: 10.1182/blood-2009-08-239145
CD73 NT5E https://www.biolegend.com/en-us/search-results/totalseq-a0577-anti-human-cd73-ecto-5-nucleotidase-antibody-16773 DOI: 10.1016/j.joen.2011.05.022
CD79b CD79B https://www.biolegend.com/nl-nl/products/totalseq-a0187-anti-human-cd79b-ig%CE%B2-antibody-16433 DOI: 10.1016/j.cell.2019.05.031
CD86 CD86 https://www.biolegend.com/en-gb/products/totalseq-a0006-anti-human-cd86-antibody-15937?GroupID=BLG11941

DOI: 10.7554/eLife.63632
CD8a CD8A https://www.biolegend.com/fr-ch/products/totalseq-a0080-anti-human-cd8a-antibody-15763
DOI: 10.1186/1479-5876-7-89
CD95 FAS https://www.biolegend.com/de-de/search-results/totalseq-a0156-anti-human-cd95-fas-antibody-16363 DOI: 10.4049/jimmunol.0903133
Kappa IGKC https://www.biolegend.com/it-it/products/totalseq-a0894-anti-human-ig-light-chain-kappa-antibody-17854
DOI: 10.1016/j.bbmt.2013.06.007
KLRG1 KLRG1 https://www.biolegend.com/en-us/search-results/totalseq-a0153-anti-human-klrg1-afa-antibody-16530?GroupID=GROUP28 DOI: 10.7554/eLife.63632
Lambda IGLC2 https://www.biolegend.com/it-it/search-results/totalseq-a0898-anti-human-ig-light-chain-lambda-antibody-18163
DOI: 10.1016/j.cell.2021.12.018
TIGIT TIGIT https://www.biolegend.com/nl-be/products/totalseq-a0089-anti-human-tigit-antibody-15773 DOI: 10.1038/s41388-018-0288-y
Isotype Ctrl Mouse IgG1, κ https://www.biolegend.com/ja-jp/products/totalseq-a0090-mouse-igg1-kappa-isotype-control-15774
DOI: 10.1182/blood-2011-02-339135
Isotype Ctrl Armenian Hamster IgG https://www.biolegend.com/de-de/products/totalseq-a0241-armenian-hamster-igg-isotype-ctrl-17278?Clone=HTK888 DOI: 10.1189/jlb.1107802
Isotype Ctrl Mouse IgG2b, κ https://www.biolegend.com/it-it/products/totalseq-a0092-mouse-igg2b-kappa-isotype-control-15778
DOI: 10.4049/jimmunol.180.12.7989
Isotype Ctrl Rat IgG2b, κ https://www.biolegend.com/it-it/products/totalseq-a0095-rat-igg2b-kappa-isotype-ctrl-16228
DOI: 10.4049/jimmunol.181.1.104
Isotype Ctrl Mouse IgG2a, κ https://www.biolegend.com/nl-be/products/totalseq-a0091-mouse-igg2a-kappa-isotype-control-15779
DOI: 10.1016/j.immuni.2020.08.004

Eukaryotic cell lines

Policy information about cell lines

Cell line source(s)

hTERT RPE-1 cells were purchased from ATCC (CRL-4000) and checked for mycoplasma contamination. C11 cells were derived in-house (from hTERT RPE-1 cells) as described previously (PMID: 32268084). GM20509, and HG01505 cell lines were purchased from Coriell and taken into culture at passage 4 (early) and passage 8 (late).

Authentication

Authentication was performed by confirming the presence of known somatic DNA rearrangements in these cell lines (e.g. the previously-reported unbalanced translocation in the case of RPE-1). Additional authentication was done at the level of SNPs shared between the cell lines, which are derived from the same anonymous donor.

Mycoplasma contamination

The cell lines tested negative for mycoplasma

Commonly misidentified lines
(See ICLAC register)

No commonly misidentified lines used in this study.

Human research participants

Policy information about studies involving human research participants

Population characteristics

We included one AML sample donor (AML_1), one CLL sample donor (CLL24), and one T-ALL sample donor (TALL P1) in this study. Patient AML_1 was 32 years of age, male, and the sample was obtained as a diagnostic bone marrow from the first aspiration of an AML with a t(8;21) translocation, arising after cytostatic therapy for testicular cancer. CLL_24 patient was 61 years old age, female, and the sample was obtained from the peripheral blood mononuclear cells. TALL patient P1 was 12 years of age, female, diagnosed with acute lymphoblastic leukemia (ALL) and the relapse sample was obtained for analysis.

Recruitment

Information about enrollment for P1 (AIEOP-ALL BFM 2009) can be found here:
https://www.kinderkrebsinfo.de/fachinformmationen/studiieenportal/abgeschlossene_studieen_register/aieop_bfmm_all_2009/indeex_ger.html or: https://clinicaltrials.gov/t2/show/NCT01117441
There was no sample selection bias which may affect to the results.

Ethics oversight

Samples used in this analysis have received approval from the relevant institutional review boards and ethics committees (University of Kiel). Written informed consent had been obtained from all the patients and the experiments conformed to the principles set out in the WMA Declaration of Helsinki and the Department of Health and Human Services Belmont Report. The protocols used in this study received approval from the relevant institutional review boards and local ethics committees. Written informed consent was obtained from patients, and all experiments were consistent with current bioethical policies. T-ALL experiments were approved by the ethics commission of the Kanton Zurich (approval number 2014-0383).

Note that full information on the approval of the study protocol must also be provided in the manuscript.

Clinical data

Policy information about clinical studies

All manuscripts should comply with the ICMJE guidelines for publication of clinical research and a completed CONSORT checklist must be included with all submissions.

Clinical trial registration

P1: https://clinicaltrials.gov/ct2/show/NCT01117441

Study protocol

The patients were treated according to the respective protocols AIEOP-ALL 2009 (P1) (details of which can be found above, in

Study protocol	Recruitment section)
Data collection	This study is prospective, controlled, randomized and multi-centered. More than 70 clinics in Germany, Austria and Switzerland are participating in the study.<br>NCT01117441 (P1) Clinical Trial title: International Collaborative Treatment Protocol For Children And Adolescents With Acute Lymphoblastic Leukemia; Allocation: Randomized; Enrollment: 4750 participants; Study Start Date: June 2010; Estimated Completion Date : December 2021
Outcomes	Since the study is still ongoing (in the case of NCT01117441 recruitment is not yet completed) and the follow-up time is too short, outcomes are not yet available and not applicable.

Flow Cytometry

Plots

Confirm that:

☒ The axis labels state the marker and fluorochrome used (e.g. CD4-FITC).

☒ The axis scales are clearly visible. Include numbers along axes only for bottom left plot of group (a 'group' is an analysis of identical markers).

☒ All plots are contour plots with outliers or pseudocolor plots.

☒ A numerical value for number of cells or percentage (with statistics) is provided.

Methodology

Sample preparation	Primary human T-ALL cells were recovered from cryopreserved bone marrow aspirates of patients enrolled in the ALL-BFM 2009 study. Patient-derived xenografts (PDX) were generated by intrafemoral injection of 1 Million viable primary ALL cells in NSG mice. PDX-derived (P1) cells were frozen until processing. Primary human AML cells were isolated via bone marrow aspiration from the donor AML_1 during diagnosis, and frozen until processing. All samples were then processed as follows: cryopreserved cells were thawed rapidly at 37 ℃ and resuspended in 10 ml warm Roswell Park Memorial Institute (RPMI) medium with 100 µg/ml Dnase I. Cells were centrifuged for 5 mins at 300 g, and resuspended in ice-cold phosphate buffered saline (PBS) with 2% foetal bovine serum (FBS) and 5mM EDTA. Samples were then stained on ice in the dark for 30 mins as follows: T-ALL P1 was stained with anti-murine-CD45-PE (mCD45) (clone 30-F11; BioLegend; 1:20), and DAPI (1:200) was added for 5 mins prior to sorting; AML_1 was stained with CD34-APC (clone 581; Biolegend), CD38-PeCy7 (clone HB7; eBioscience), CD45Ra-FITC (clone HI100; eBioscience), CD90-PE (clone 5E10; eBioscience), and LIVE/DEAD™ Fixable Near-IR Dead Cell Stain (Thermofisher). After staining, cells were washed once in 4 ml ice-cold PBS with 2% FBS and 5 mM EDTA and centrifuged at 300 g for 5 mins. Cells were resuspended in ice-cold PBS with 2% FBS and 5 mM EDTA for sorting.
Instrument	BD FACSAria™ Fusion Cell Sorter
Software	FlowJo, BD FACSDiva™
Cell population abundance	Due to limited sample material, post-sort purities were not re-assessed using flow cytometry. Instead, this was done by gating and quantification of populations using Flowjo (Supplemental Figures S21 and S27). In the case of AML_1: 83.5% of the total events were included after gating out debris in the FSC-A vs SSC-A plot; 95.4% of these events were within the Single Cells gate (based on SSC-W vs SSC-A); 51.5% of these Single Cells were gated as Viable Cells (based on Fixable Live/Dead Near-IR viability stain vs FSC-A); and the final sorting population of CD34+ cells represented 46.4 % of the Viable Cells (based on CD34-APC vs CD38-PeCy7). In TALL P1: 86.7% of the total events were retained after debris exclusion (based on FSC-A vs SSC-A); 96% of these events were gated as Single Cells (based on SSC-W vs SSC-A); 20.8% of the Single Cells were gated as Viable Cells (based on DAPI viability stain vs FSC-A); and of these Viable Cells, 72.8% were gated as human, T-ALL cells (based on low murine CD45-PE expression).
Gating strategy	For TALL P1: FSC-A vs SSC-A was the starting gate, wherein debris was excluded. Next, Single Cells were gated based on the exclusion of outliers in SSC-W vs SSC-A. Viable Cells were then gated within this population based on a low staining for DAPI (DAPI-viability vs FSC-A). Finally, human T-ALL cells were discriminated from murine immune cells based on a low expression of murine CD45 (murine CD45 - PE vs GFP). The full gating strategy is depicted in Supplemental Figure S27.<br>For AML_1: The first gate excluded any cellular debris based on FSC-A vs SSC-A. These cells were then sub-gated to identify only Single Cells, based on removal of outliers from the SCC-W vs SSC-A plot. Viable Cells were gated within the Single Cells based on a low intracellular staining for the viability stain Fixable LIVE/DEAD near-IR (Fixable LIVE/DEAD near-IR Viability vs FSC-A). Finally, the ultimate sorting population of CD34+ AML cells was gated based on a high expression of CD34 (CD34-APC vs CD38 PeCy7). The full gating strategy is depicted in Supplemental Figure S21.

☒ Tick this box to confirm that a figure exemplifying the gating strategy is provided in the Supplementary Information.
