# Supplementary material for: Functional analysis of structural variants in single cells using Strand-seq
Source: Nat Biotechnol. 2022 Nov 24;41(6):832–44. doi: 10.1038/s41587-022-01551-4 (PMC10264249; doi:10.1038/s41587-022-01551-4)

Snapshots of somatic SV events in LCLs

Single-cell SV events in LCLs (NA12329, 59 single cells profiled)

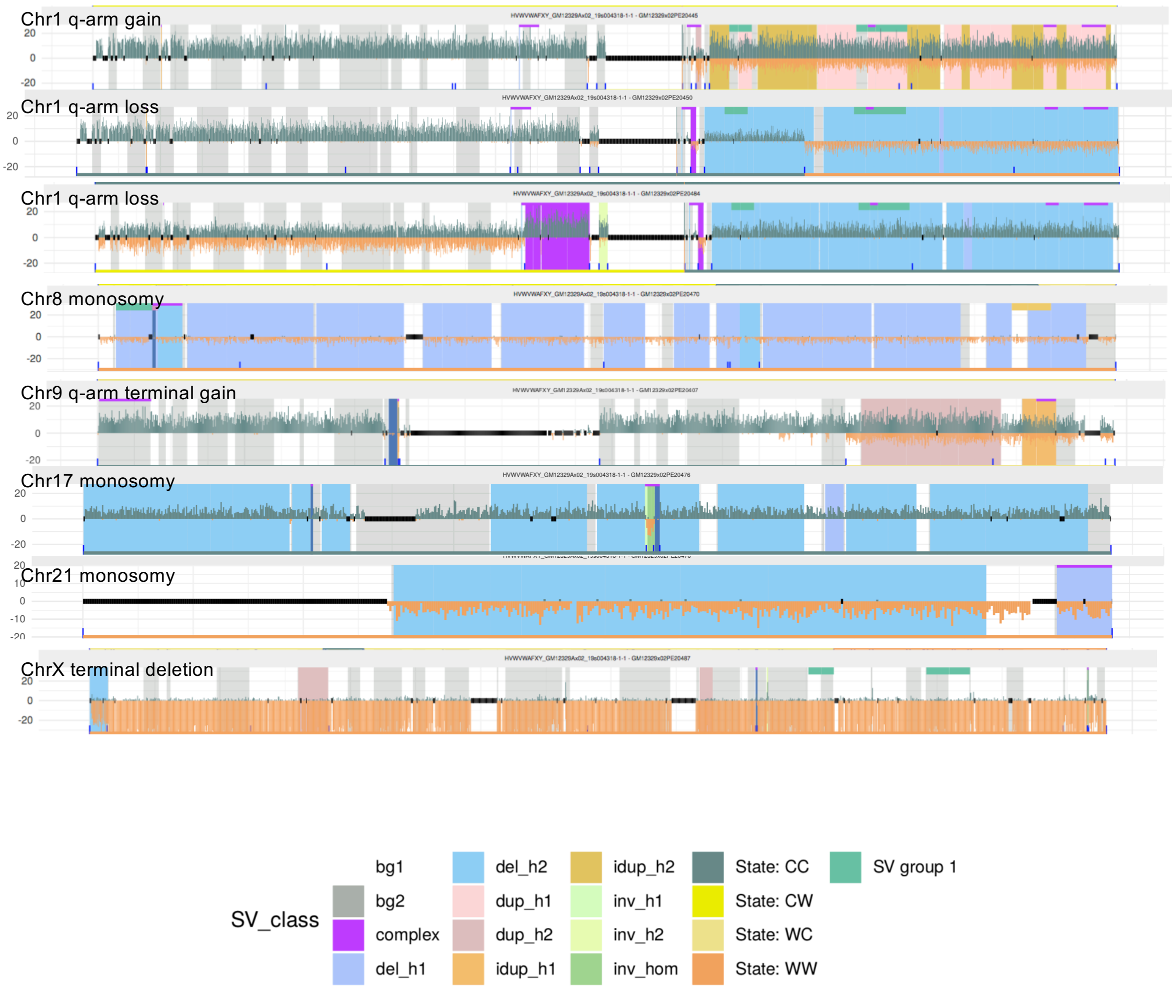

Single-cell SV events in LCLs (NA18534, 56 single cells profiled)

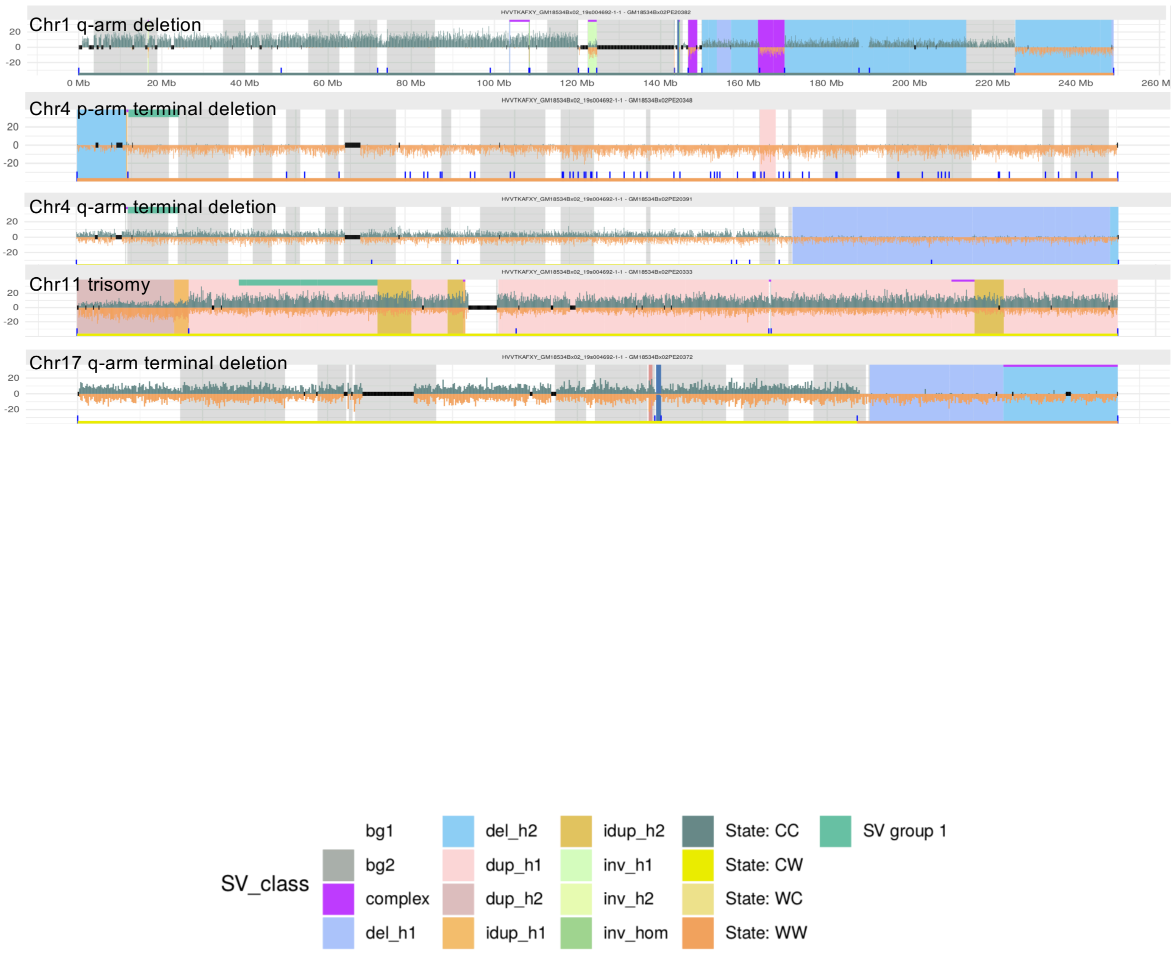

Single-cell SV events in LCLs (NA18939, 41 single cells profiled)

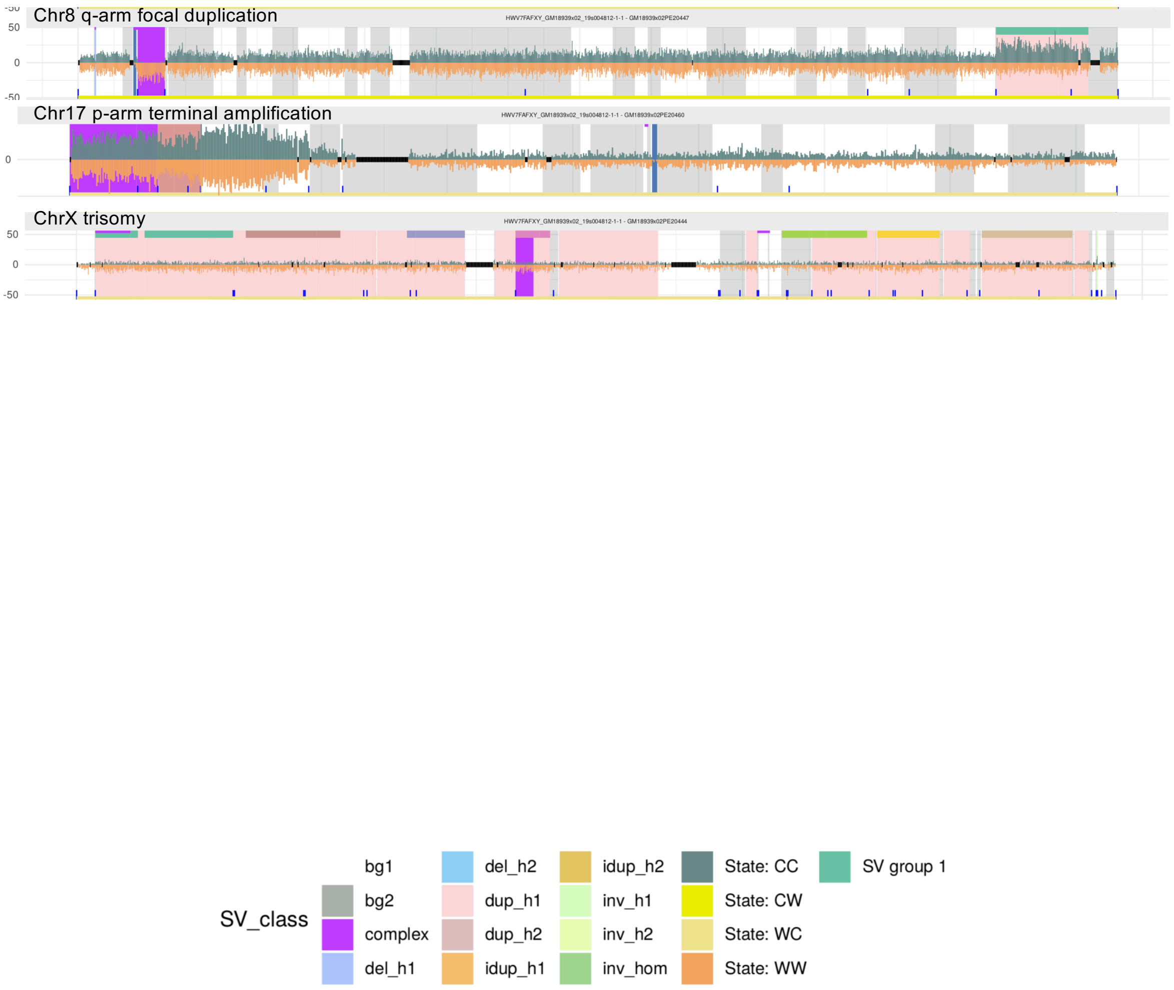

Single-cell SV events in LCLs (NA19650, 55 single cells profiled)

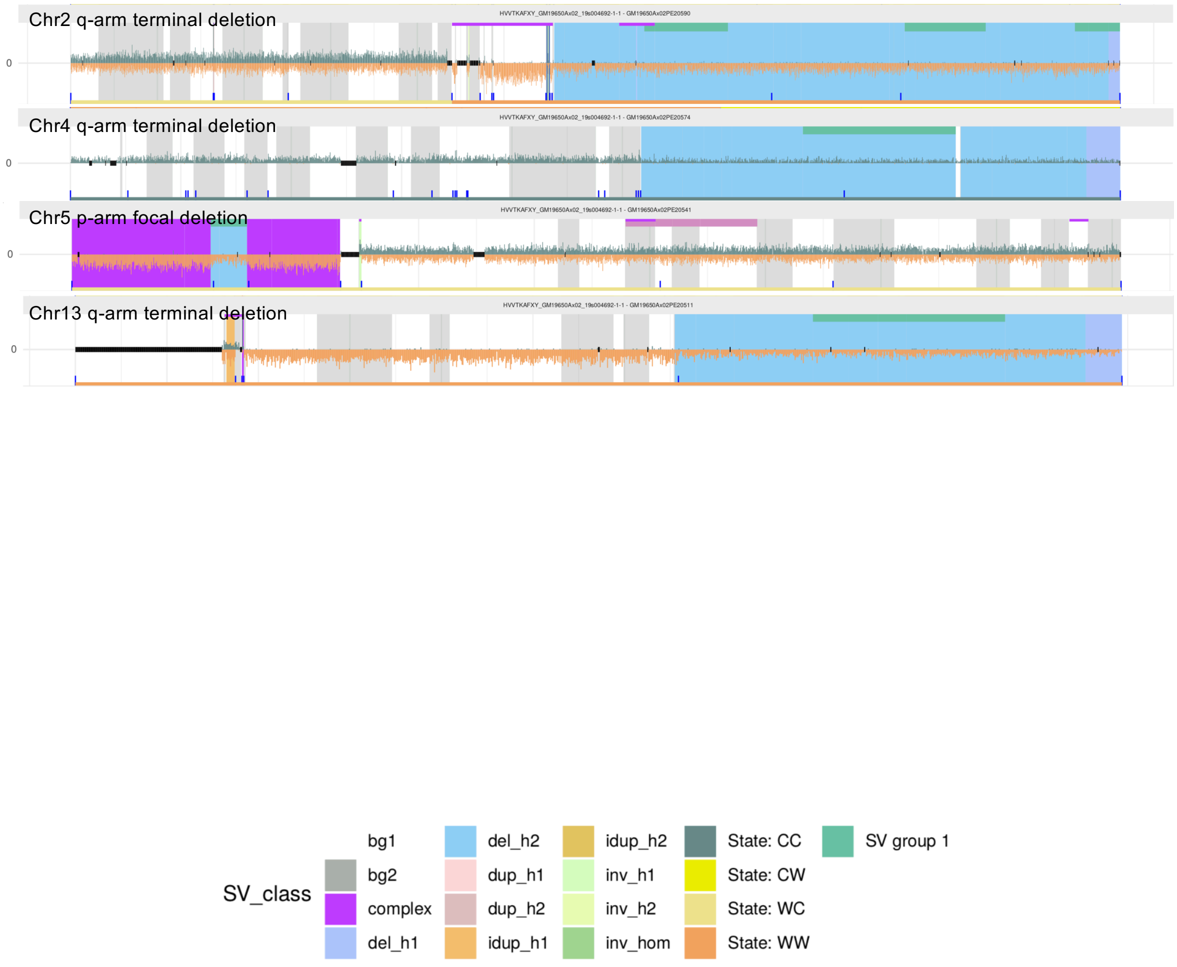

Single-cell SV events in LCLs (NA19983, 45 single cells profiled)

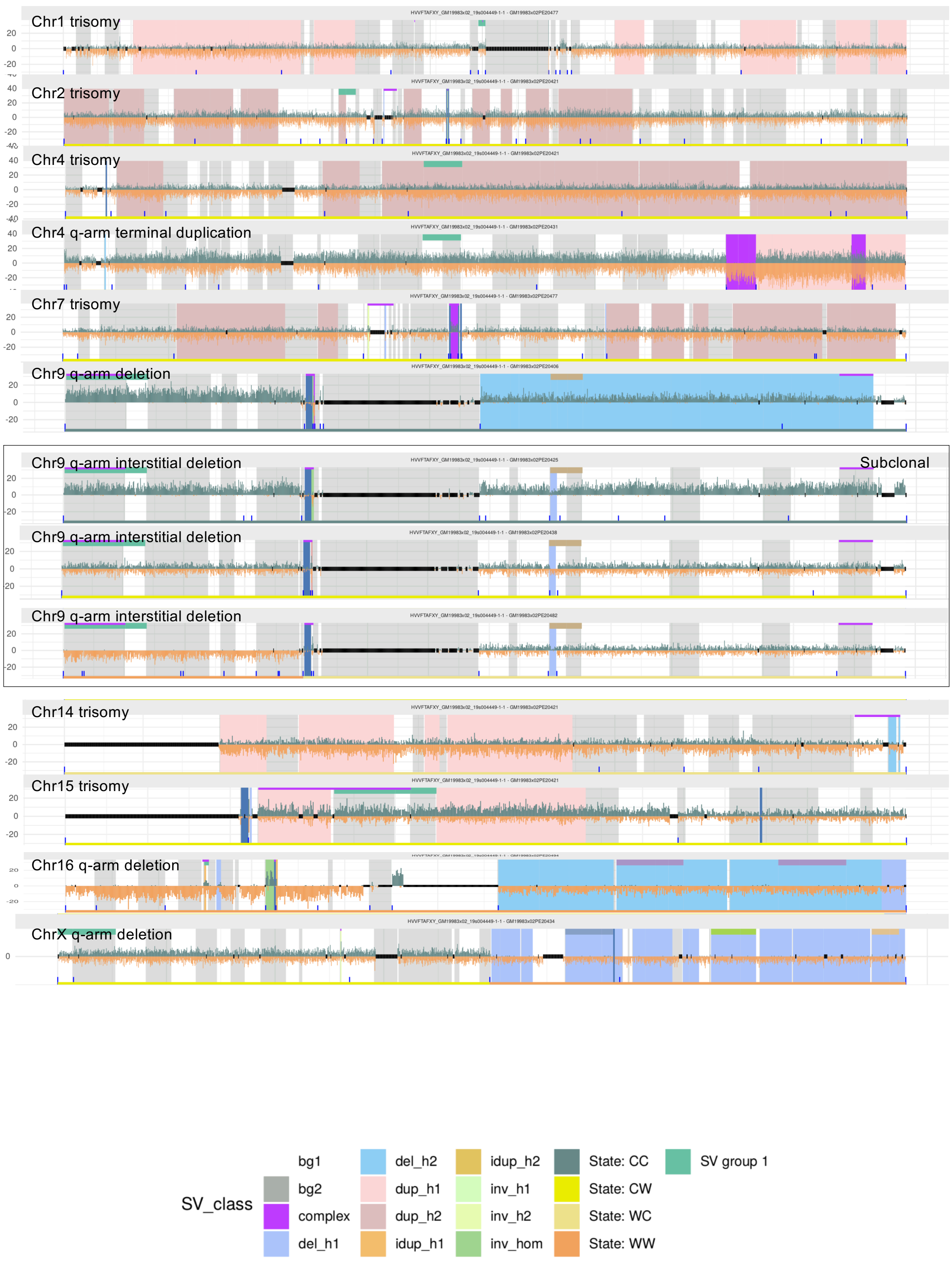

Single-cell SV events in LCLs (NA20509, 47 single cells profiled)

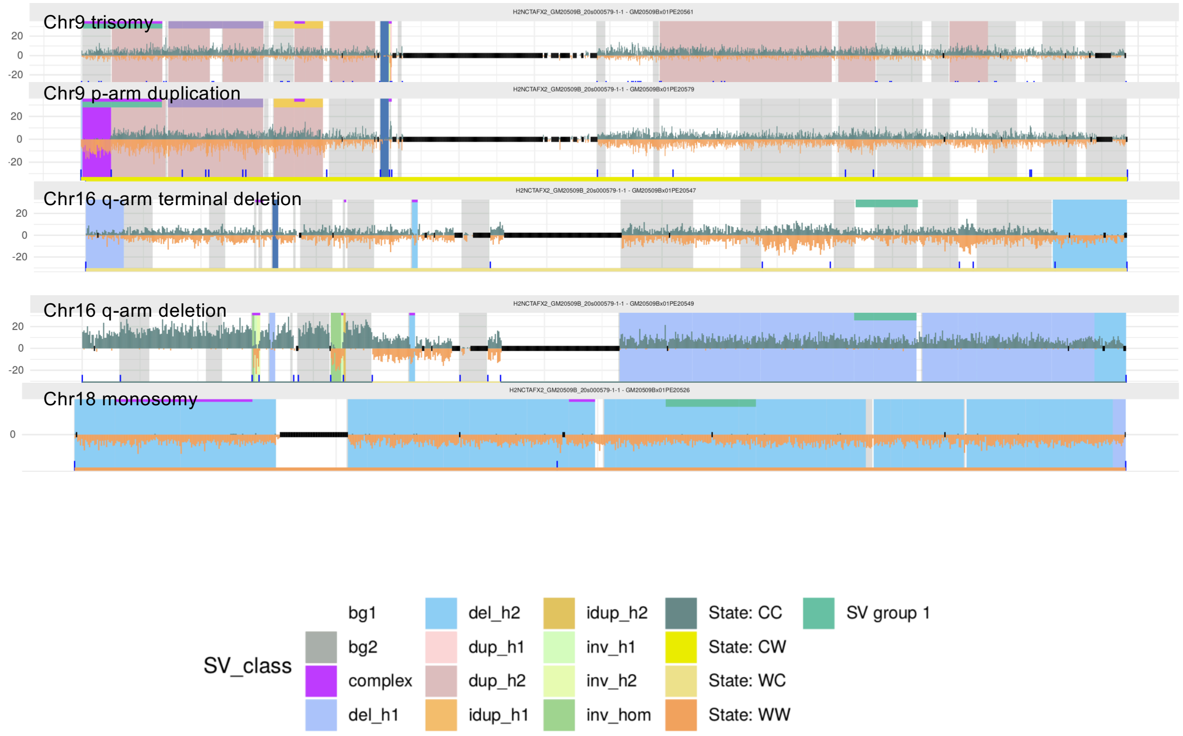

Single-cell SV events in LCLs (NA20847, 55 single cells profiled)

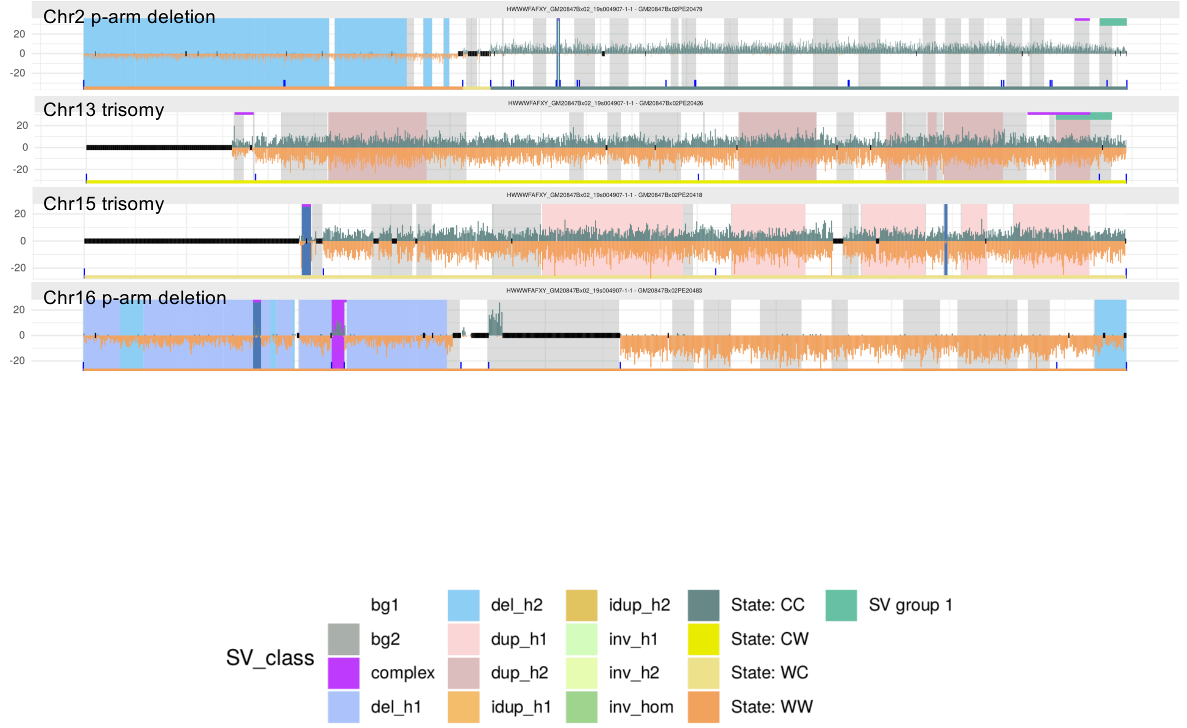

Single-cell SV events in LCLs (HG00096, 69 single cells profiled)

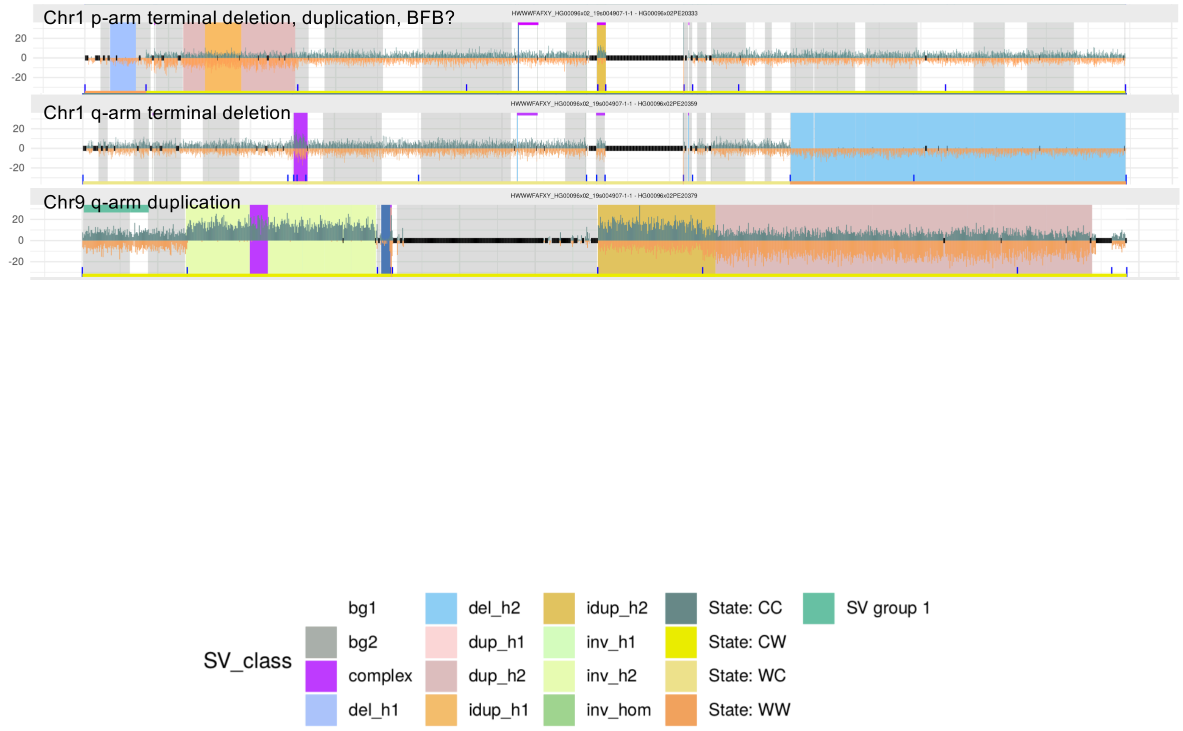

Single-cell SV events in LCLs (HG00171, 49 single cells profiled)

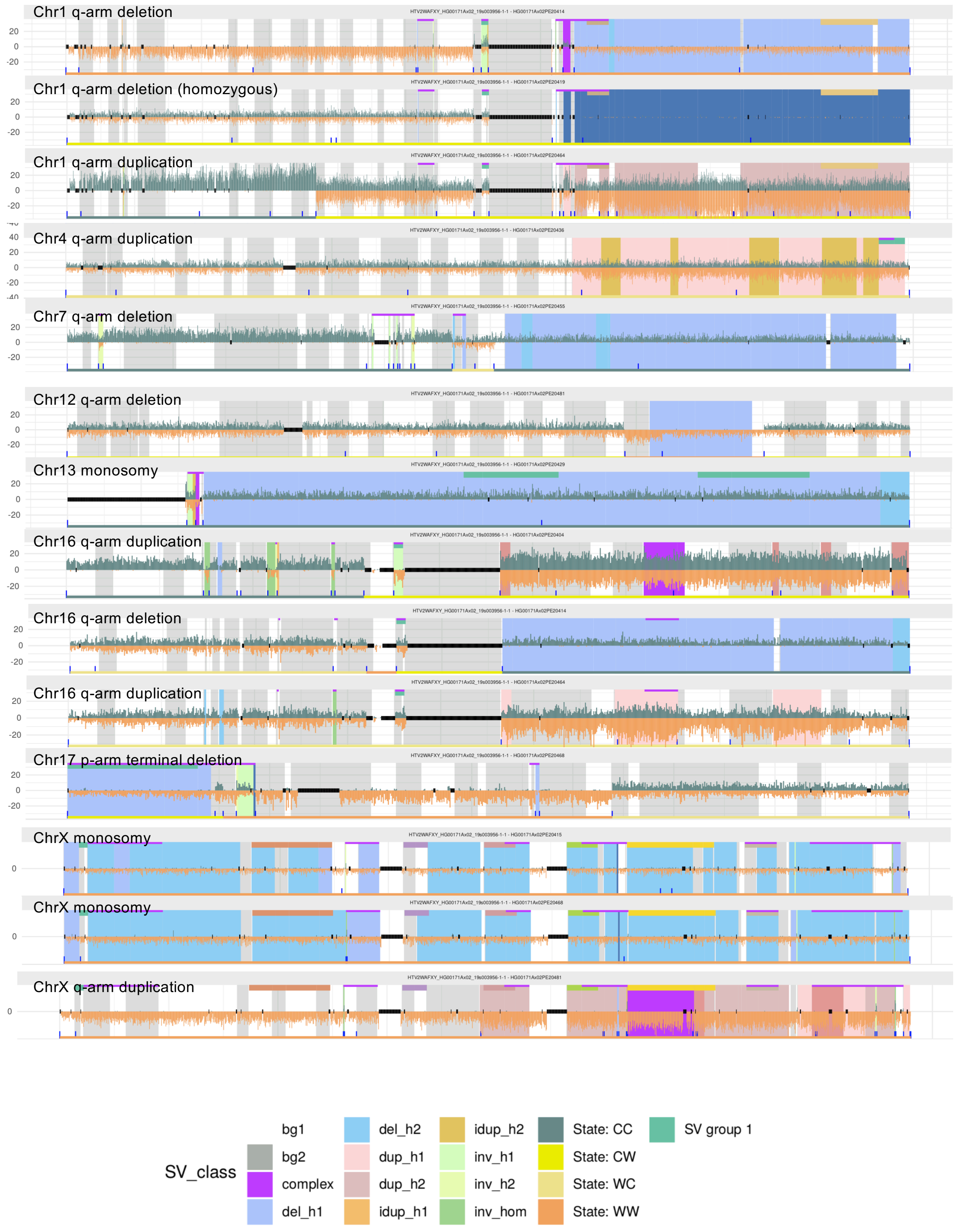

Single-cell SV events in LCLs (HG00864, 43 single cells profiled)

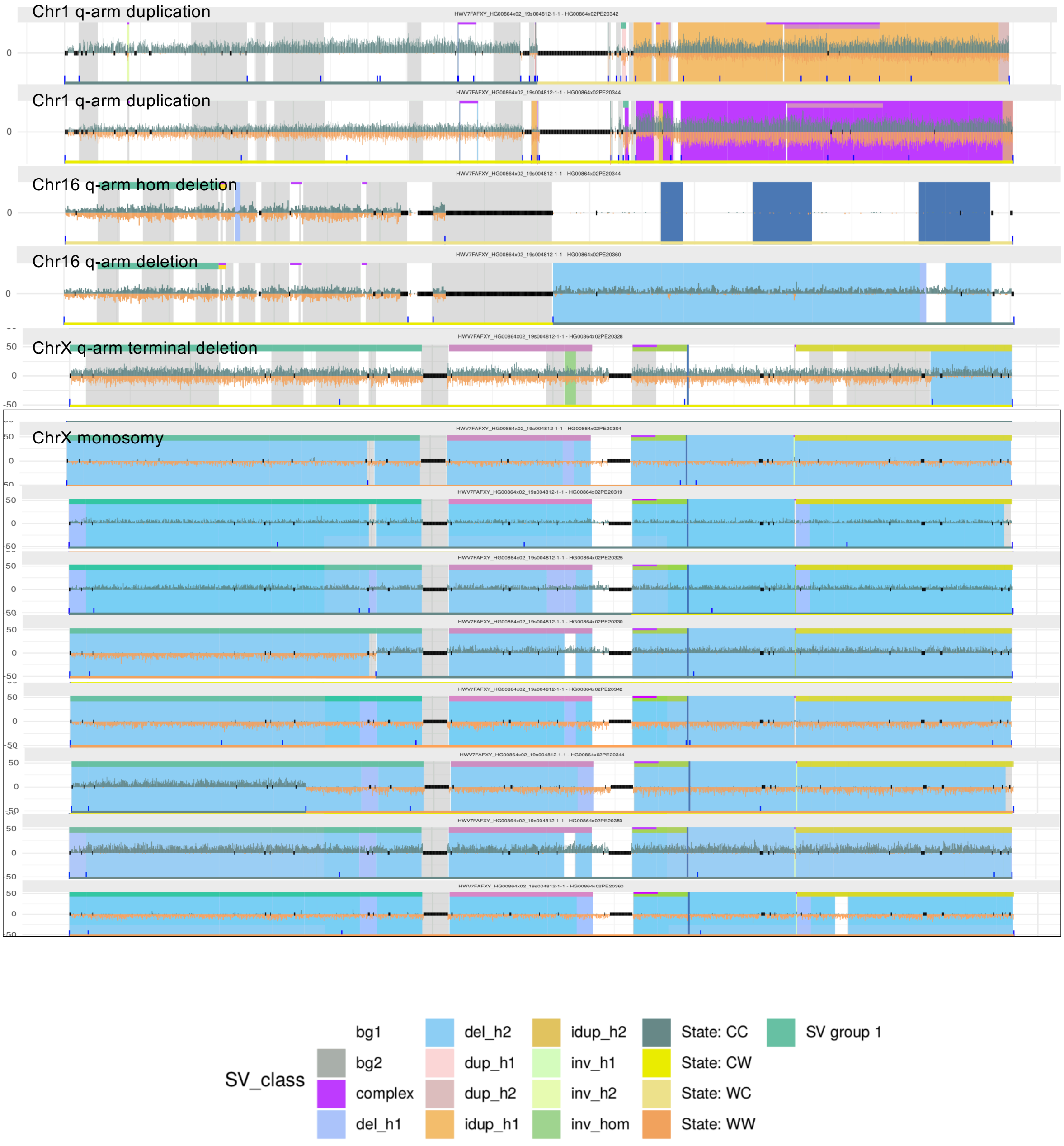

Single-cell SV events in LCLs (HG01114, 51 single cells profiled)

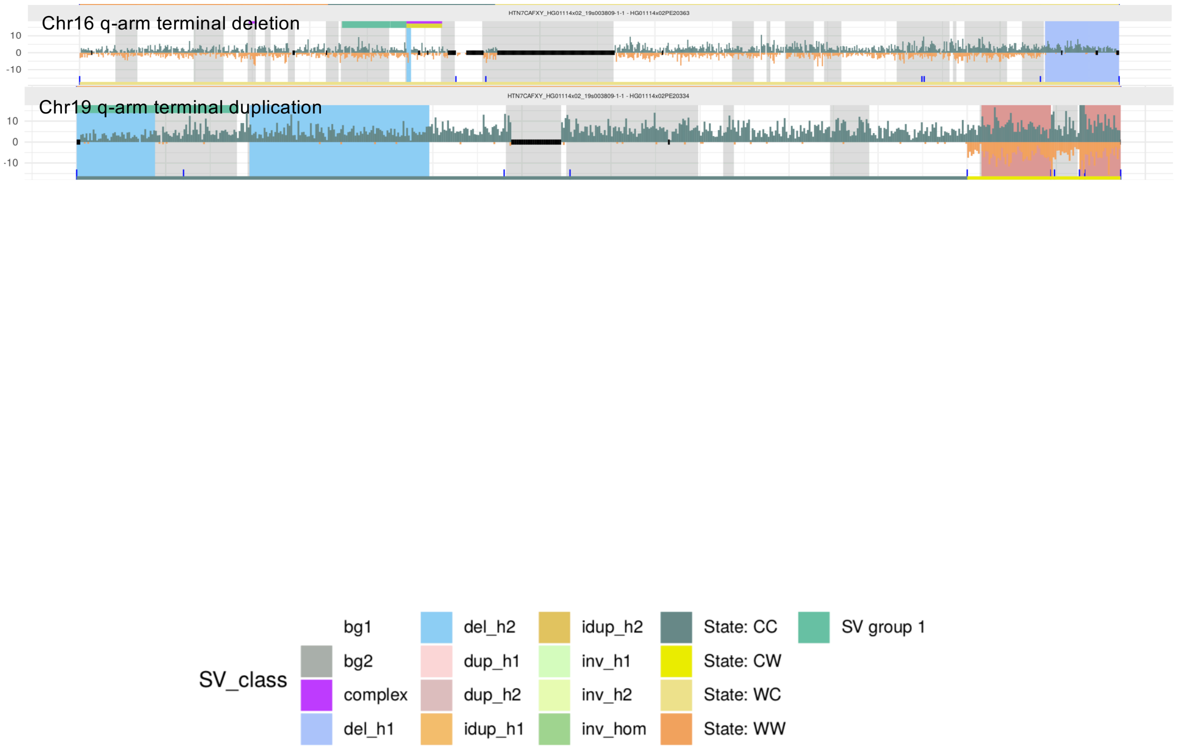

Single-cell SV events in LCLs (HG01596, 53 single cells profiled)

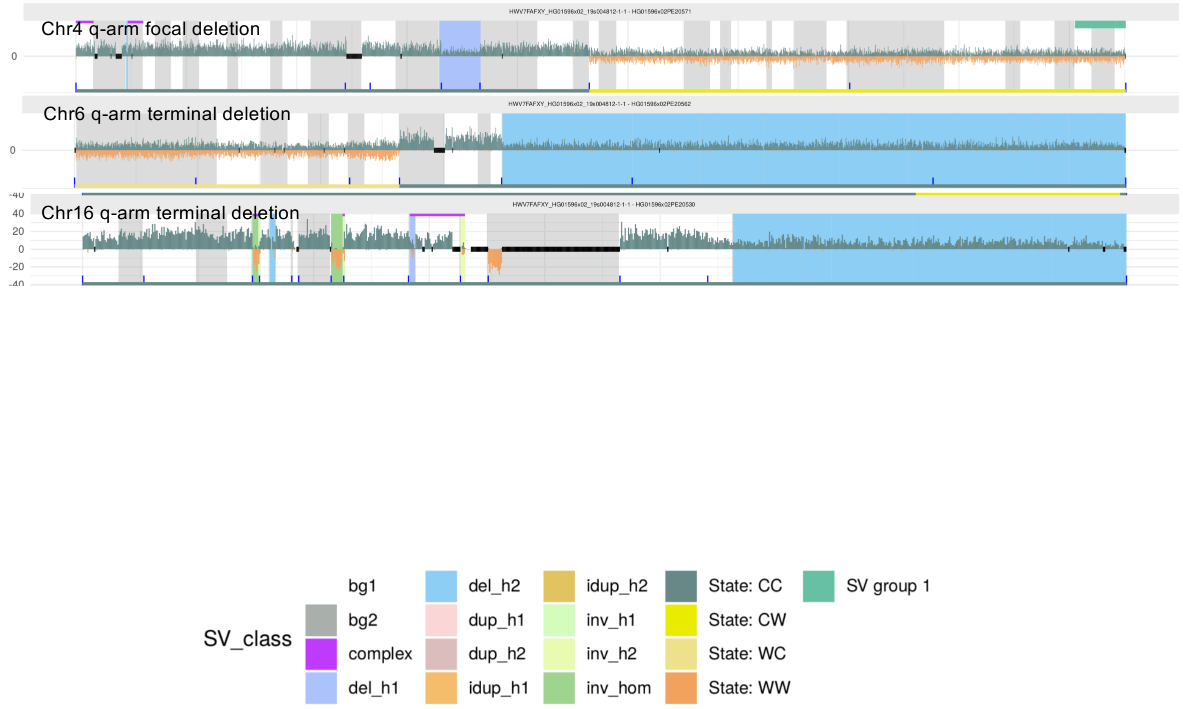

Single-cell SV events in LCLs (HG02011, 55 single cells profiled)

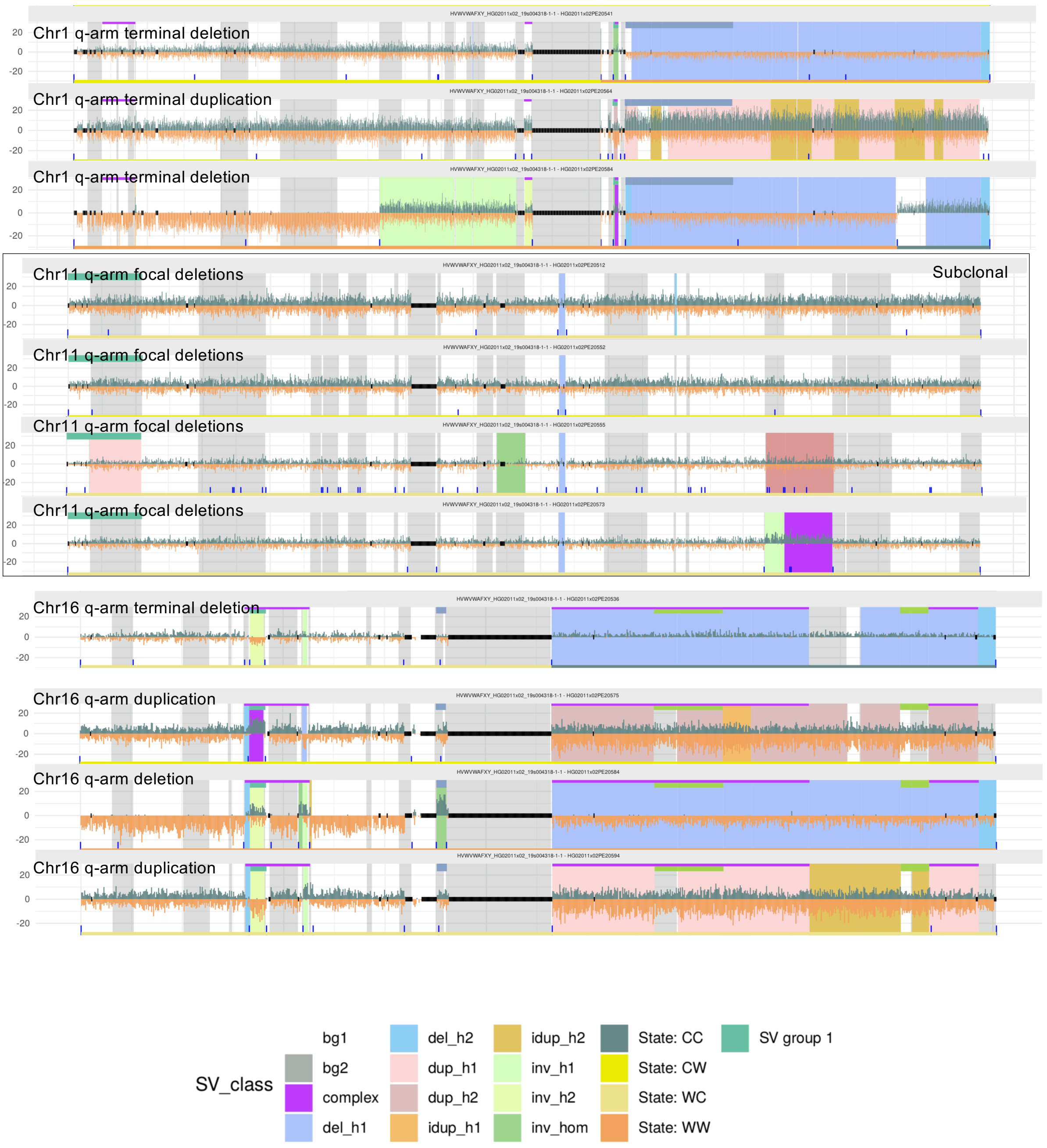

Single-cell SV events in LCLs (HG02492, 30 single cells profiled)

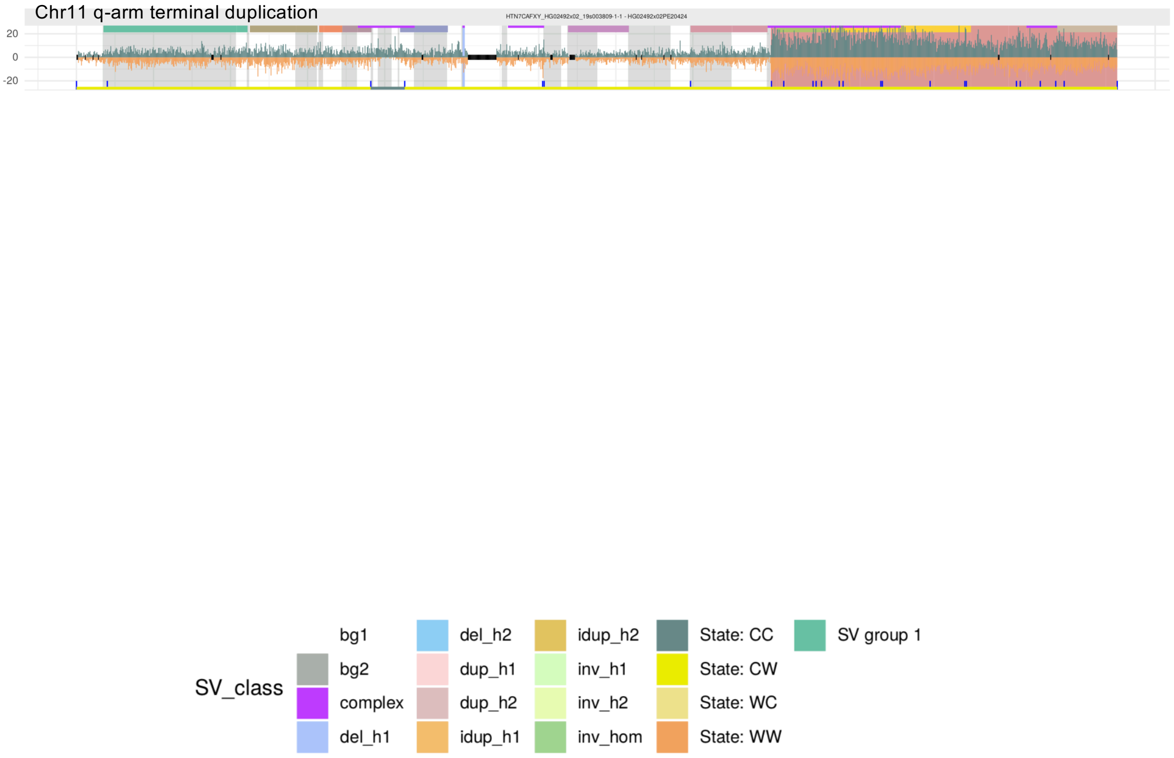

Single-cell SV events in LCLs (HG02587, 54 single cells profiled)

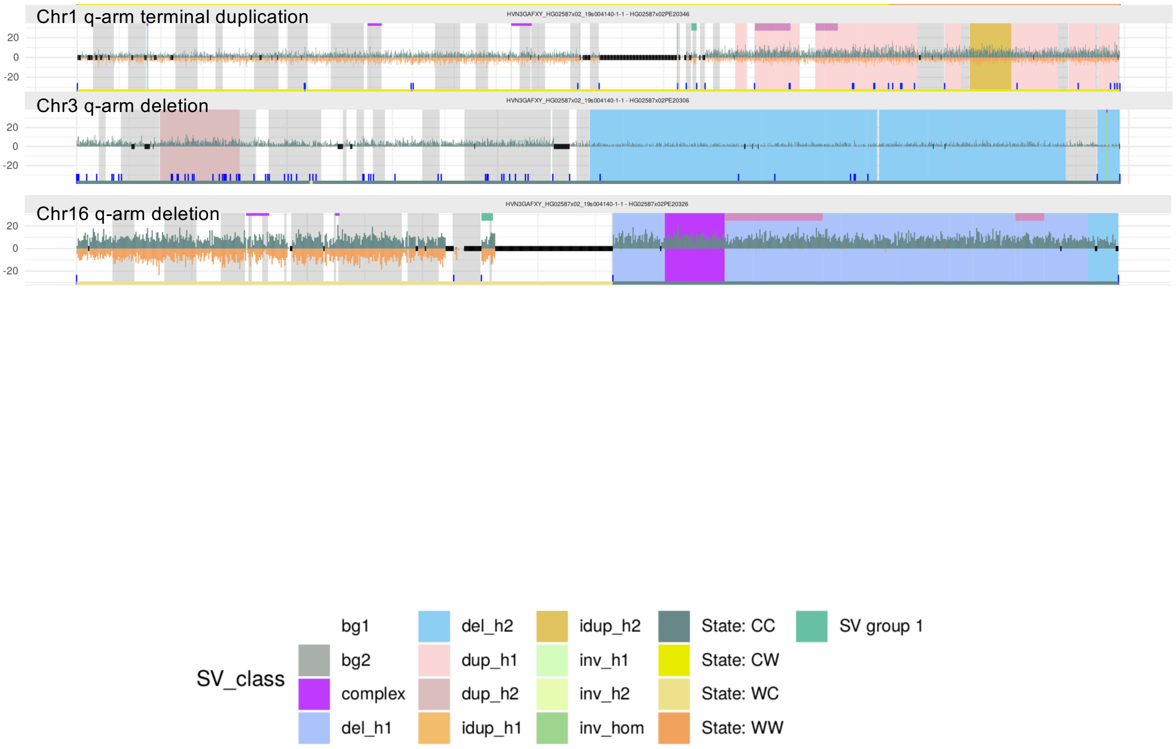

Single-cell SV events in LCLs (HG02818, 69 single cells profiled)

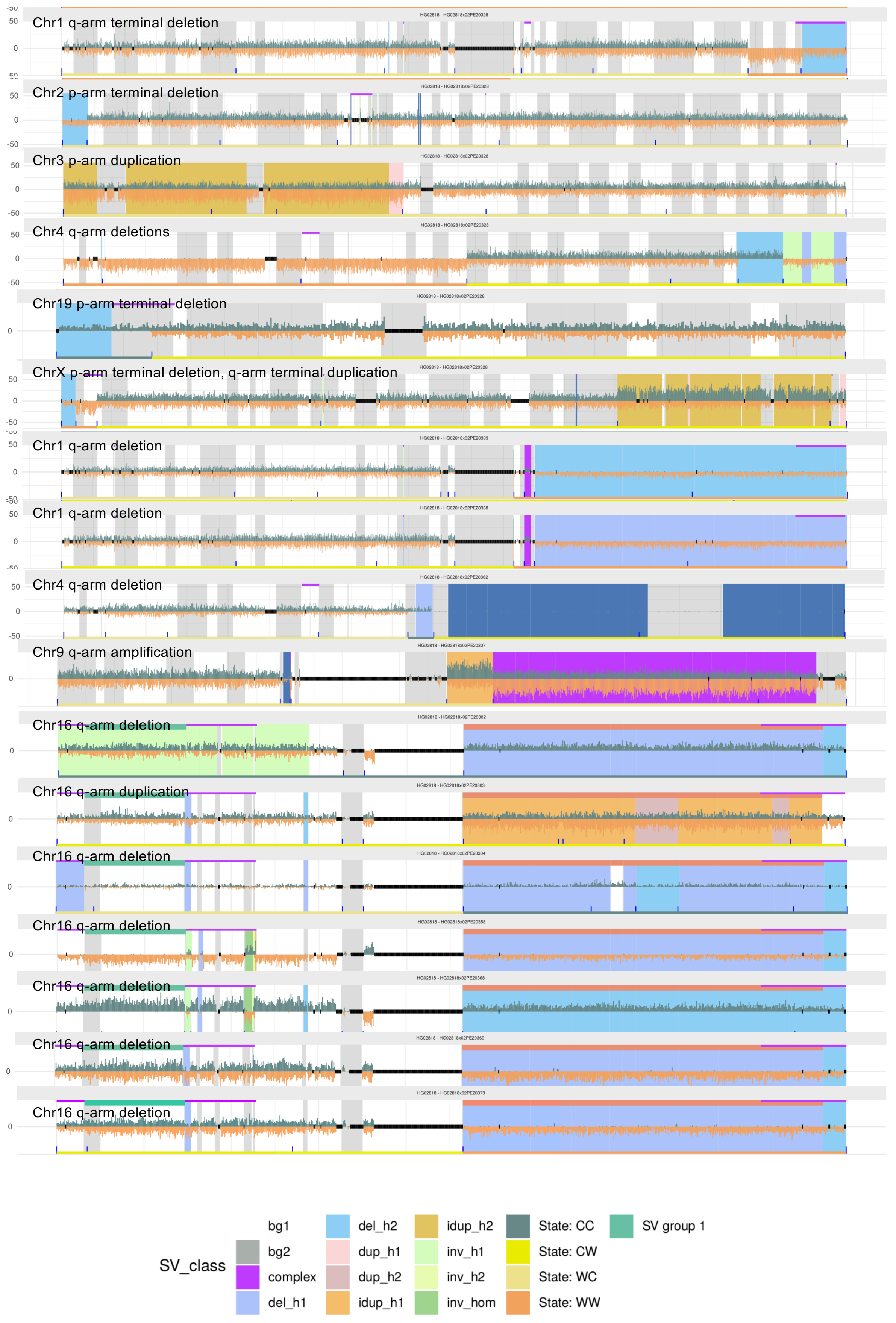

Single-cell SV events in LCLs (HG03009, 55 single cells profiled)

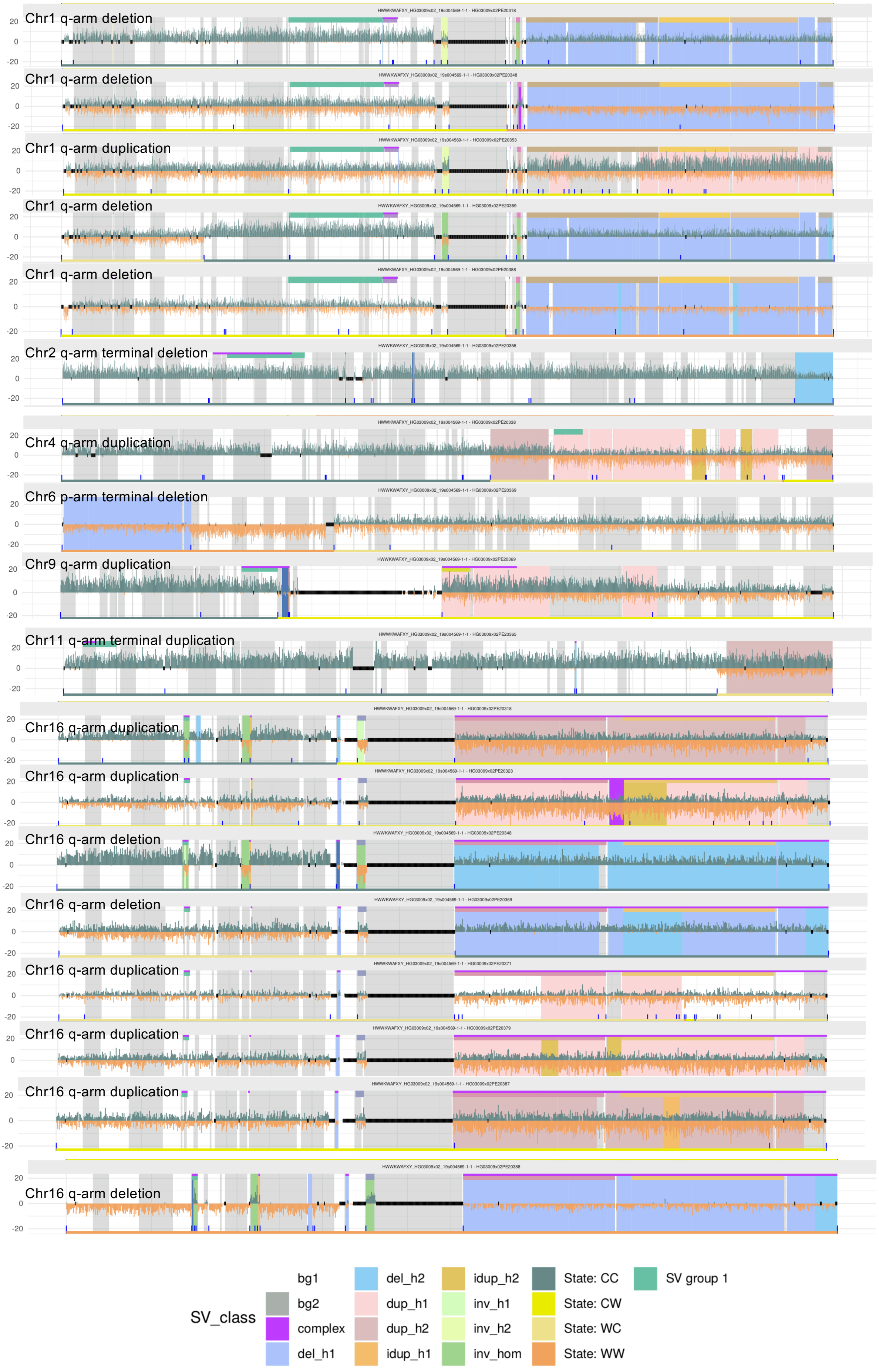

Single-cell SV events in LCLs (HG03065, 56 single cells profiled)

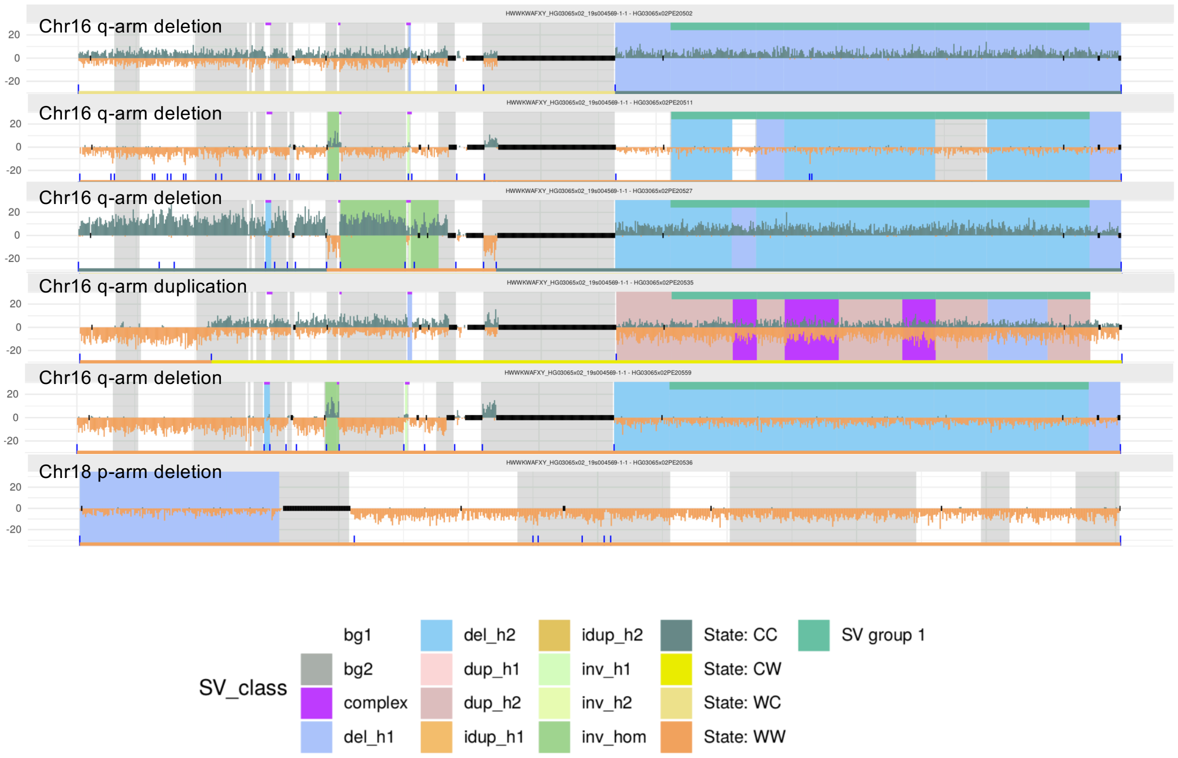

Single-cell SV events in LCLs (HG03125, 55 single cells profiled)

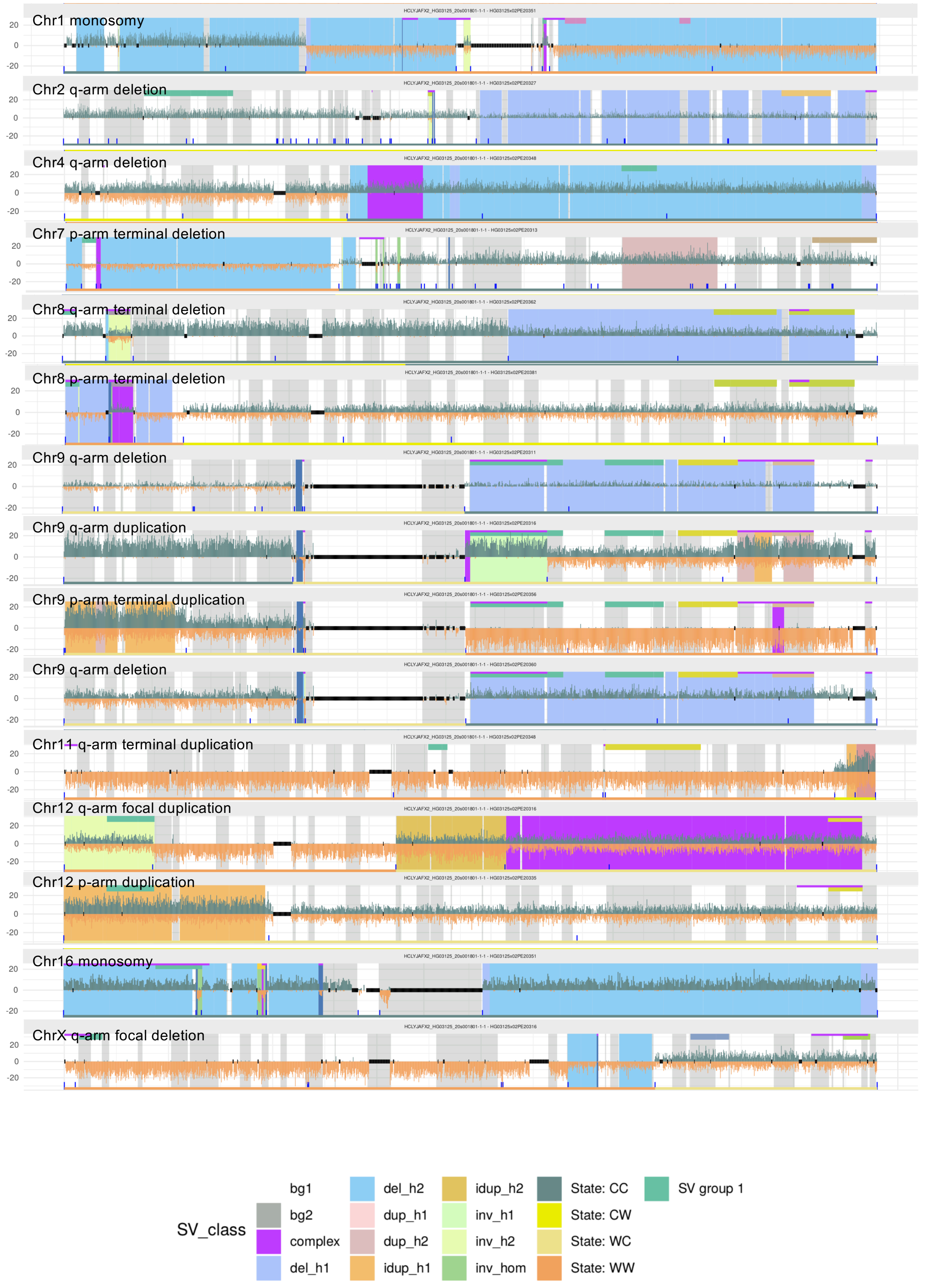

Single-cell SV events in LCLs (HG03371, 61 single cells profiled)

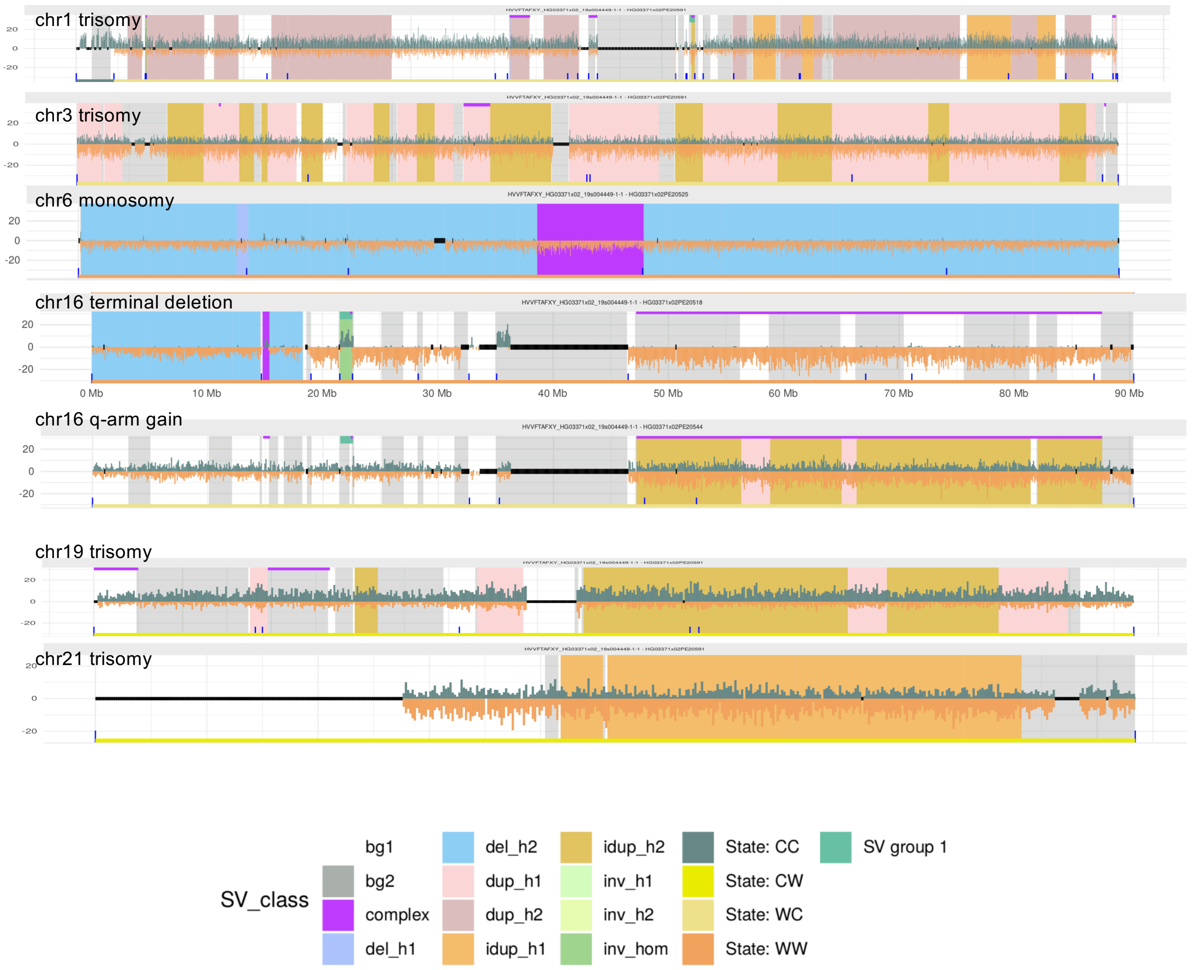

Single-cell SV events in LCLs (HG03486, 61 single cells profiled)

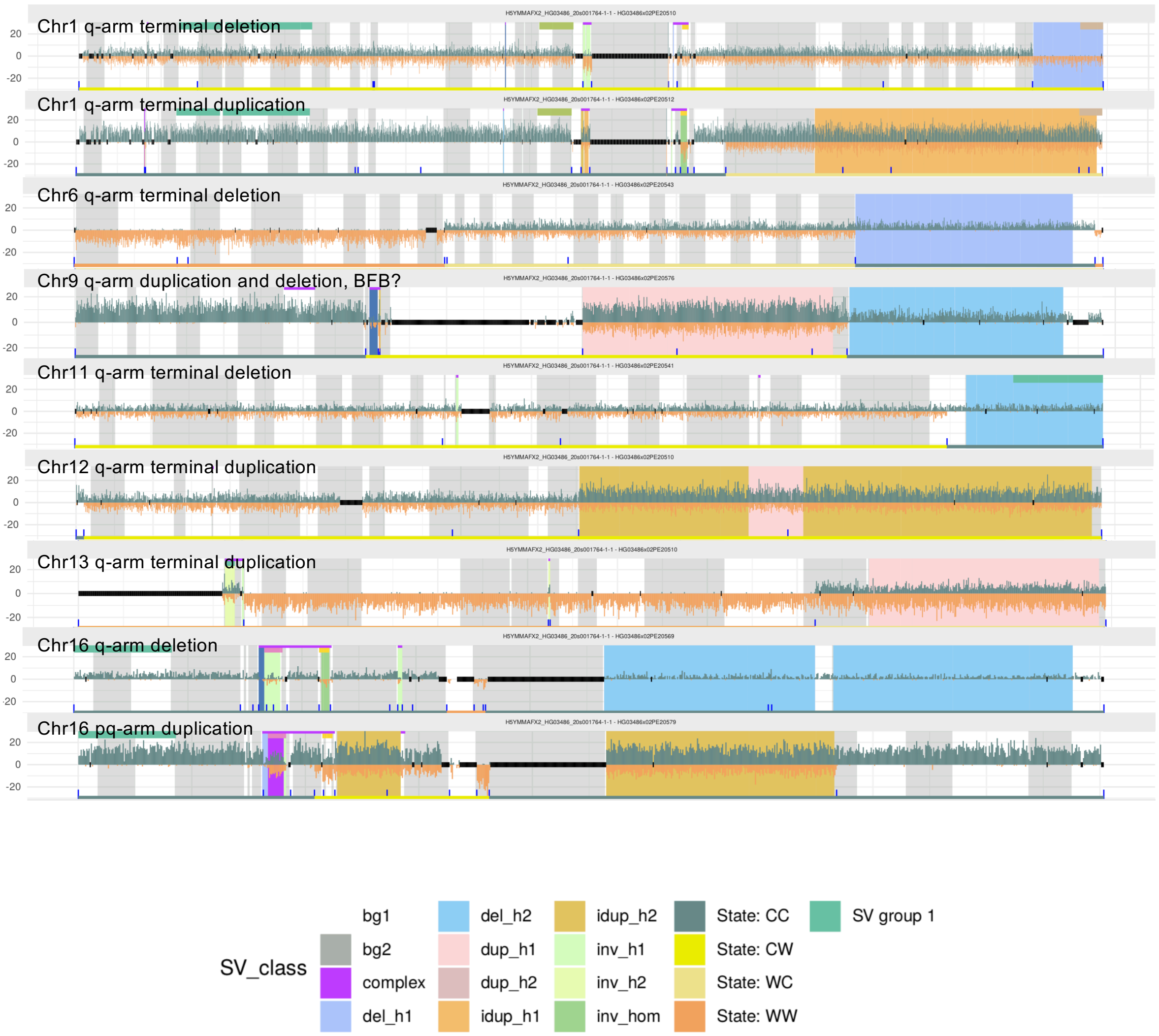

Single-cell SV events in LCLs (HG03683, 50 single cells profiled)

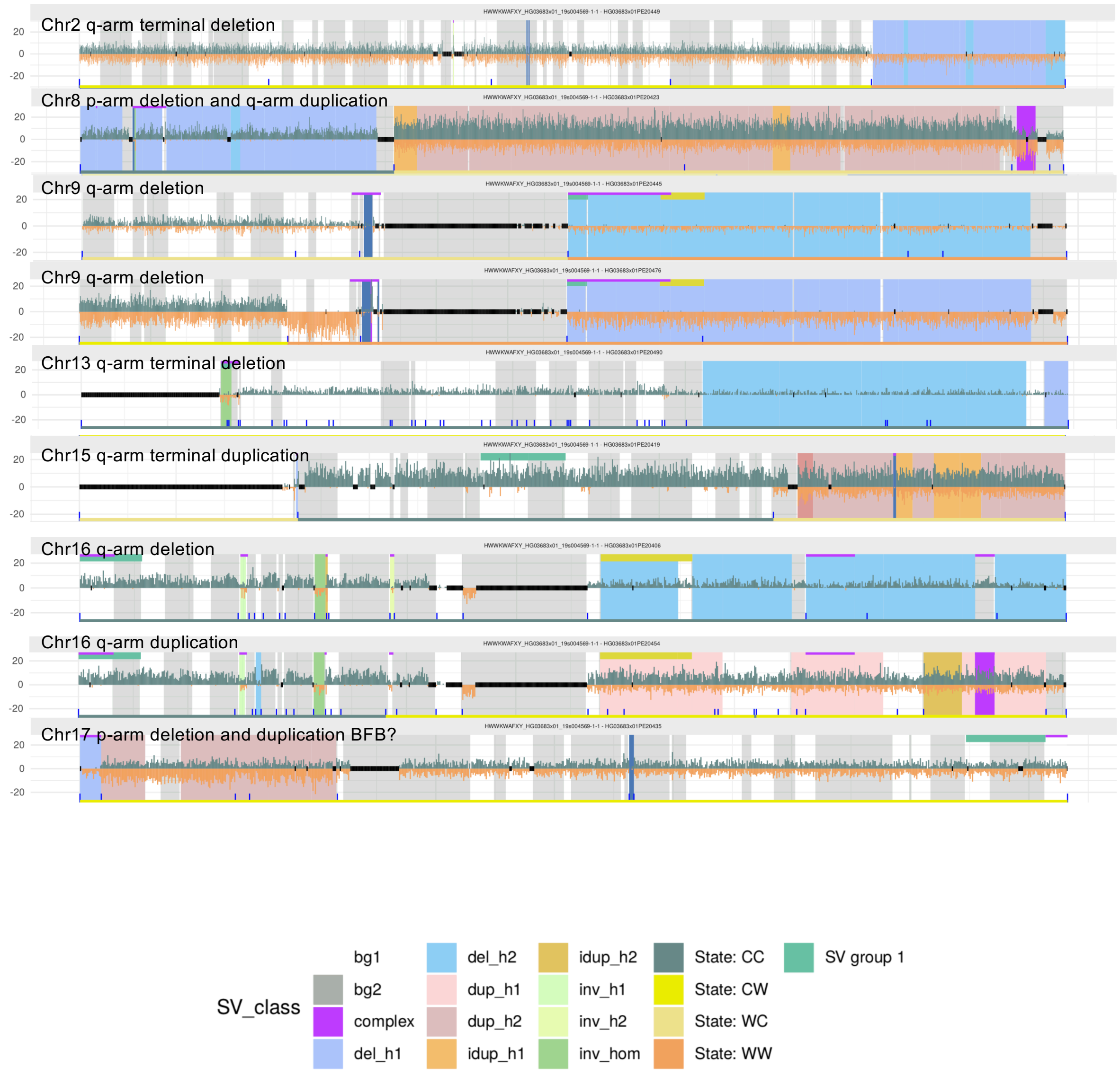

Single-cell SV events in LCLs (HG03732, 47 single cells profiled)

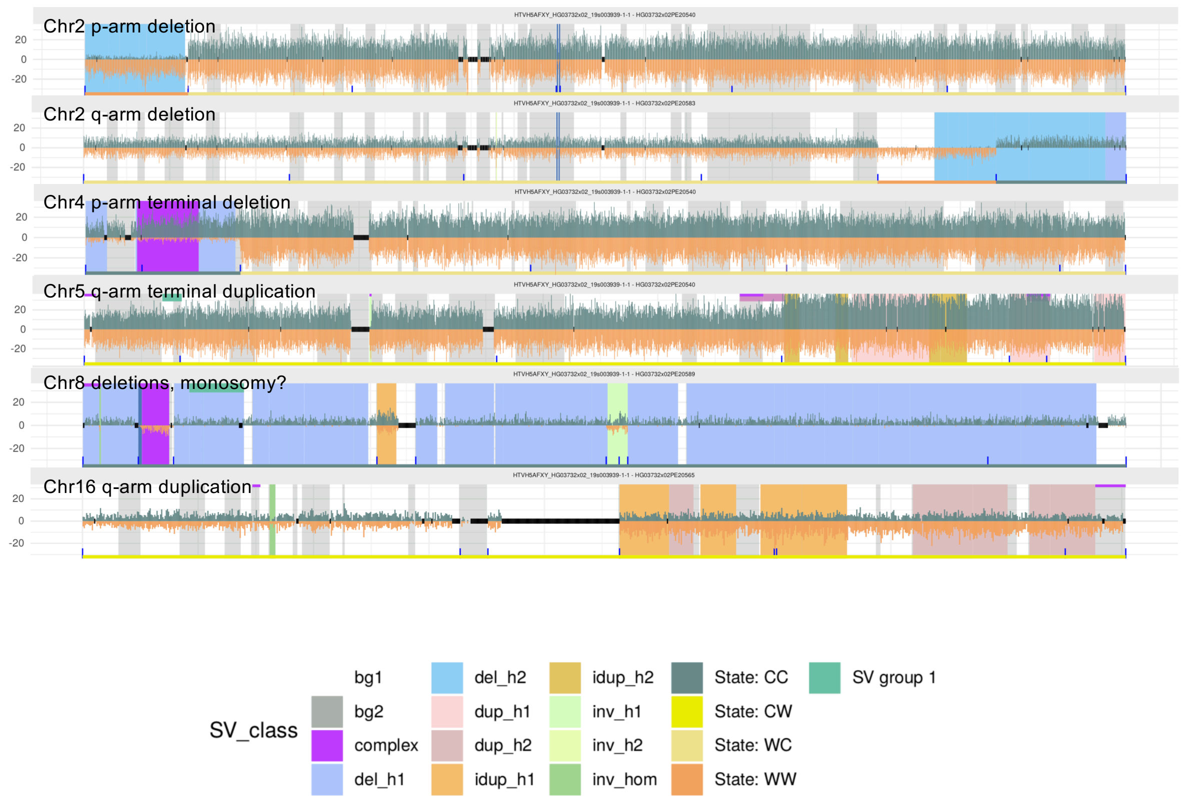

Supplement: Supplementary file 4 — Snapshots of somatic SV events in LCLs. [file 41587_2022_1551_MOESM4_ESM.pdf]
